# Botulinum Toxin Effects on Biochemical Biomarkers Related to Inflammation-Associated Head and Neck Chronic Conditions: A Systematic Review of Preclinical Research

**DOI:** 10.3390/toxins17080377

**Published:** 2025-07-29

**Authors:** Ines Novo Pereira, Giancarlo De la Torre Canales, Sara Durão, Rawand Shado, Ana Cristina Braga, André Mariz Almeida, Haidar Hassan, Ana Cristina Manso, Ricardo Faria-Almeida

**Affiliations:** 1 Faculty of Dental Medicine, University of Porto (FMDUP), Rua Dr. Manuel Pereira da Silva, 4200-393 Porto, Portugal; 2 Egas Moniz Center for Interdisciplinary Research, Egas Moniz School of Health & Science, 2829-511 Almada, Portugal; 3 Division of Oral Diagnostics and Rehabilitation, Department of Dental Medicine, Karolinska Institutet, SE-14104 Huddinge, Sweden; 4 Institute of Dentistry, Royal London Dental Hospital, Faculty of Medicine & Dentistry, Queen Mary University, Turner Street, London E1 2AD, UK; 5 School of Engineering (EEUM) ALGORITMI Research Centre, The Intelligent Systems Associate Laboratory (LASI), Campus de Gualtar, University of Minho, 4710-057 Braga, Portugal; 6 Centre for Cutaneous Research, Blizard Institute of Cell and Molecular Science, Faculty of Medicine & Dentistry, Queen Mary University of London (QMUL), 4 Newark Street, Whitechapel, London E1 2AT, UK; 7 Department of Dental Implantology, Rey Juan Carlos University (URJC), Av. de Atenas, S/N, 28922 Alcorcón, Spain; 8 LAQV/REQUIMTE, Periodontology and Oral Surgery Department, Faculty of Dental Medicine, University of Porto (FMDUP), Rua Dr. Manuel Pereira da Silva, 4200-393 Porto, Portugal

**Keywords:** biomarkers, botulinum toxin, inflammation, head and neck, chronic conditions, animal studies

## Abstract

Current research reported that the number of clinical studies found for botulinum toxin (BoNT) key effects on biochemical biomarkers in head and neck chronic conditions linked to inflammation was very low. There are no systematic reviews of animal studies on this topic, and hence our review aimed to evaluate the quality of the preclinical evidence. We searched PubMed, Scopus, and Web of Science databases, and registries up to 29 January 2024. There were 22 eligible records, and data were available for 11 randomised controlled trials. There were concerns about the risk of bias and great variations of data obtained regarding chronic conditions, which included mostly trigeminal neuralgia. The leading biomarkers were proinflammatory cytokines (IL-1β, TNF-α) and synaptosomal-associated protein-25 (SNAP25), followed by neuron activation marker c-Fos and calcitonin gene-related peptide (CGRP). Overall, data found that BoNT significantly altered the under/over-expression of biomarkers evoked by the investigated disease models and had no effect when the levels of these biomarkers were not changed by the induced chronic conditions in animals. However, there were some mixed results and exceptions, and the certainty evidence found was very low to low. Although the sample sizes detected significant effect size (*p* < 0.05), most studies are based on male inferior animals, which may limit the recommendations for clinical trials. This study is registered on PROSPERO (CRD42023432411).

## 1. Introduction

In recent years, botulinum toxin (BoNT) has been suggested as an alternative treatment to a few chronic conditions characterised by inflammatory states [1,2]. The unexpected discovery that the on-label cosmetic protocol for BoNT to ameliorate glabellar lines was beneficial in chronic migraine and has the potential to improve mood led to extensive research in the realm of headaches, pain conditions, and psychological illness [3,4]. Given the limitations of the few available pharmacotherapeutic options for the treatment of some of these chronic conditions, more attention has been paid to the promising long-lasting clinical effects and safety profile of BoNT [5].

The efficacy of BoNT therapy could not be unequivocally proven in some conditions or patient subpopulations [5]. Nevertheless, a trend for positive responses has been observed for many chronic conditions that have been linked to inflammation [6]. The lack of strong clinical evidence, to some extent, may be explained by methodological limitations of the studies being performed [7]. In addition, there is still fundamental unknown information on BoNT mechanisms that may preclude high-quality studies evaluating efficacy and immediate clinical translation [6].

At present, the mechanisms that mediate the non-motor effects of BoNT are not fully understood and constitute a field of active investigation [6]. Several factors related to BoNT mechanisms of action (e.g., protease longevity inside neurons and specificity for SNARE proteins, repeatability and reversibility of neuroparalysis, segmental actions in the sensory nucleus/spinal cord dorsal horn, specificity for hyperactive neurons, and selectivity for determined sensory neurons) may impact the therapeutic success of BoNT in certain clinical settings [6]. Hence, there are efforts taking place to uncover the potential effects of BoNT in the peripheral sensory nerves and central nervous system [1].

In parallel, the identification and application of biomarkers has gained increasing attention in the research and clinical fields [8,9]. Among the numerous advantages of biomarkers is their potential for evaluating the mechanistic actions of drugs through a precise measurement, providing less bias and an objective assessment [10]. In addition, a recent systematic review found limited studies in humans evaluating BoNT key effects on biochemical biomarkers, mostly calcitonin gene-related peptide (CGRP), and concluded that BoNT may influence some biomarkers found in head and neck chronic inflammatory conditions [7]. Therefore, deeper understanding of this topic, likely to broaden the horizon of BoNT research and clinical practice, is needed. Despite challenges and controversies, preclinical experimentation is thought to play a crucial role in research aiming to develop health care, providing unique insights into the search for new drug targets and biomarkers [11]. Moreover, exploratory animal studies may offer a broader range of possibilities to assess the drug’s pharmacokinetic and pharmacodynamic parameters [11].

In this direction, there has been growing awareness of the value of systematic reviews of animal studies to evaluate the quality of the preclinical evidence [12]. However, there are currently no systematic reviews of animal studies summarizing the key effects of BoNT and the relationship with biochemical biomarkers in head and neck chronic conditions characterised by inflammatory states. Understanding the effects of BoNT on the biomarkers that have been used in preclinical studies may offer an important step forward in future research, by encouraging a more consistent approach for measuring the therapeutic effects of BoNT. Moreover, preclinical research should focus on preferential targeting of BoNTs to relevant pathways for improving chronic inflammatory conditions, which may allow a clearer insight into the neurobiological mechanisms involved, as well as expedite the development of new strategies (e.g., novel engineered BoNT) with the potential to improve the safety and clinical efficacy. Therefore, it is important to scrutinise the validity of preclinical evidence in this emerging field.

## 2. Results

A total of 2049 records resulted from searching the databases and registers. After the duplicates were removed, 1519 remained for title and abstract review, including 142 records identified by manual screening of references. Following the full-text review of 111 documents, 22 articles were included [13,14,15,16,17,18,19,20,21,22,23,24,25,26,27,28,29,30,31,32,33,34]. The full Rayyan report is displayed in Supplementary File S1. A full list of citations that were excluded is available upon request.

**Figure 1:**
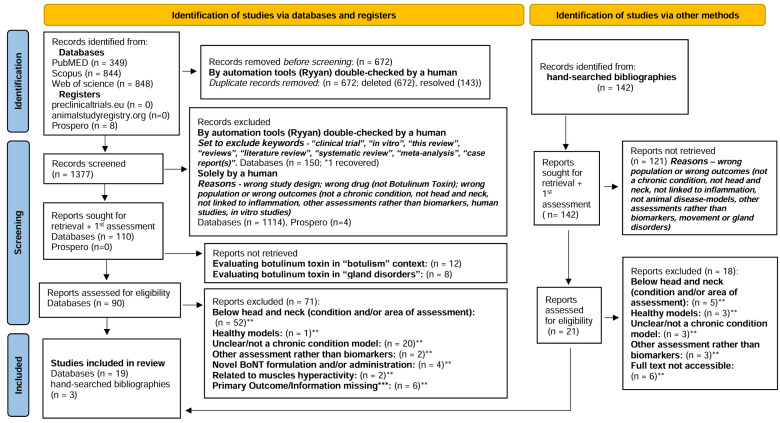
PRISMA 2020 flow diagram for new systematic reviews which included searches of databases, registers, and other sources [41]. * Articles Recovered for further assessment [18] ** Articles requiring adjudication regarding eligibility; *** Protocol for missing information (contact 1st attempt 28 February 2024; 2nd attempt 11 March 2024; 3rd attempt 26 March 2024) [35,36,37,38,39,40].

We did not include in our review 89 records requiring adjudication regarding eligibility, and the reasons for exclusion were listed and are also available upon request. Of these, we have tried to contact the authors of five studies for more information regarding the location of chronic lesions and/or BoNT intervention [35,36,37,38,39], and one study using a model of hypertrophic scarring that did not report whether BoNT was administered into the wound or an established hypertrophic scar [40]. Only one author replied to confirm that the “rosacea-like inflammation” model was established below the head and neck [36], and hence all six studies were excluded (Supplementary File S2, Table S1). The selection process is illustrated in the PRISMA Flow Diagram (Figure 1) [41].

The Supplementary File S3, Table S2 provides full methodological detail regarding the included records. Table 1 shows the summary of the characteristics of the 22 included studies. These were published between 2009 and 2023. Sample sizes ranged from 12 to 525, with nine studies not providing a clear study sample [15,18,21,23,24,26,29,31,32]. Only inferior animals were used for the experiments and there were variations in terms of species, weight, and age. Among the 22 studies, male animals represented the majority, only three studies used female animals [19,27,30], and one study did not specify this variable [25]. Studies also reported different timing and routes of BoNT administration, as well as variations in models for induction of diseases in animals. Overall, nine studies focused on disease models of trigeminal neuralgia [14,17,20,23,26,28,29,31,32], four studies explored scenarios of temporomandibular joint (TMJ) arthritis such as TMJ osteoarthritis (*n* = 1) [13], and TMJ rheumatoid arthritis (*n* = 3) [15,16,22], three records described experiments on depression [18,21,24], including one in major depressive disorder (MDD) [24], two articles reported on hypertrophic scar settings [25,30], while migraine [19], atopic dermatitis [27], anxiety [34], and chronic dry skin itch conditions were each discussed in one article [33]. The most commonly reported animal species were Sprague Dawley rats (*n* = 8) and Wister rats (*n* = 5). The specific instant in time for the measurement of the biomarker used in each study ranged from 30 min to 60 days following the administration of BoNT. Biological sampling locations included rostral dorsal skin, serum (retro orbital plexus), ear scar tissues, (periarticular) TMJ tissues, trigeminal ganglia (TG), TG sensory neurons, TG mandibular V3 division and boundary area, trigeminal (sub)nucleus caudalis (TNC), jugular plasma and medulla oblongata containing TNC, brainstem Vc region (caudal subnucleus of the spinal trigeminal nucleus), substantia nigra pars compacta, ventrolateral periaqueductal gray, hippocampus, hypothalamus, prefrontal cortex, amygdala, dura mater, cranial dura plasma protein complexes, dorsal root ganglia, medullary dorsal horn, and cerebrospinal fluid sampling.

### 2.1. Risk of Bias

**Figure 2:**
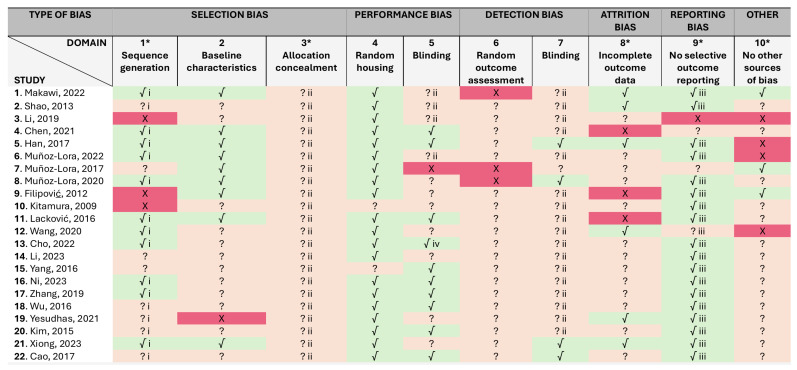
SYRCLE’s tool for assessing risk of bias. * Items in agreement with the items in the Cochrane Risk of Bias tool. Key: √, yes; X, no. ?, unclear. i, the investigators did not describe the method used for a random component in the sequence generation process. ii, it is unlikely that the outcome or the outcome measurement was influenced by this domain, given the objective measurements. iii, the study protocol was not available, but it was clear that the published report included all expected outcomes (i.e., comparing methods and results section). iv, single blind. A “yes” judgement indicates a low risk of bias; a “no” judgment indicates high risk of bias; the judgment will be “unclear” if insufficient details have been reported to assess the risk of bias properly. It is not recommend calculating a summary score for each individual study when using this tool [13,14,15,16,17,18,19,20,21,22,23,24,25,26,27,28,29,30,31,32,33,34].

A summary of the risk of bias assessments is presented in Figure 2, while justifications for assessments were recorded in the SYRCLE’s risk of bias tool, which are available on Supplementary File S4, Table S3. Overall, there were concerns about the risk of bias for all studies in at least one of the ten domains. However, 20 studies out of 22 were judged low risk for performance bias (4th domain), given that all measures used to house the animals randomly within the animal room were described. Although none of the animal studies registered the protocols in a central, readily available database, 18 studies were considered free of selective outcome reporting (9th domain), once it was clear that all the expected outcomes were included. Half of the studies (*n* = 11) reported a random approach, although the authors did not describe the random component in the sequence generation process. In addition, only four studies demonstrated that blinding of the outcome assessor was secured (7th domain), and no study provided enough detail to assess selection bias (3rd domain), i.e., whereas allocation to the different groups was sufficiently concealed. These may lead to overoptimistic conclusions regarding the treatment effects. However, given that objective measurements (biomarkers levels) were evaluated, we judged that it was probably unlikely that the outcome was influenced by these two domains. That said, we found that the major methodological weakness across the included studies lies in the inadequate reporting of whether or not animals were selected at random for outcome assessment and/or at different time points, which may facilitate the prediction of the allocation of the animals and influence the results or the direction of the magnitude of the effect. For example, due to circadian rhythms in some biological processes, certain biomarker levels may vary during the day and depend on the timing of measurements. Beyond these, there were further issues that raised concerns about the possibility of other types of bias, which included design-specific risks of bias (e.g., lack of control groups and short follow-up), the limitations of some animal models to adequately replicate the clinical disorder, and the male over-representation, skewing our comprehension of disease processes and precluding the successful translation to clinical trials. It was noteworthy that only 3 out of 22 included studies reported no specific sources of funding, but just 3 were funded by the pharmaceutical industry without disclosing the conflicts of interest [22,26,31].

### 2.2. Results of Individual Studies

To facilitate the interpretation of the relevance of the biomarker to each reported condition and the administration of BoNT, Table 2 displays data across the studies for specific biomarkers according to the frequency, each targeted condition and specific biological sampling employed. It also provides a summary of the potential mechanisms and biological rationale, as well as information as to whether the biomarkers can be considered for clinical trials. Table 2 shows the biomarkers that have been reported in more than one study, while those reported in single experiments are illustrated in Supplementary File S5, Table S4.

#### 2.2.1. Biomarkers

The leading biomarker with six studies were proinflammatory cytokine—interleukin 1 beta (IL-1β) [13,14,15,16,17,18], followed by tumor necrotic factor alfa (TNF-α) and synaptosomal-associated protein-25 (SNAP25) with five studies each [14,15,16,17,18,20,21,22,23,24]. Neuron activation marker c-Fos and calcitonin gene-related peptide (CGRP) were reported in four studies [14,15,19,20,22,24,26], while substance P (SP) [15,19], IL-6 [14,17], microglial purinergic CX3 chemokine receptor 1 (CX3CR1) [16,18], dural protein extravasation [20,28], transforming growth factor beta (TGF-β1) [25,30], collagen-related proteins (I, III) [25,30], α-smooth muscle actin (α-SMA) and myosin II proteins [25,30], and transient receptor potential ankyrin 1 (TRPA1) were explored in two studies each [23,33]. Finally, data exists for single experiments with microglia marker—ionized calcium-binding adaptor molecule 1 (IBA-1) [29], chemokine subclass fractalkine/CX3CL1 [18], marker for GABAergic neurons—inhibitory presynaptic marker vesicular GABA transporter (VGAT) [18], inhibitory synaptic markers VGAT co-localised with Gephyrin [18], inhibitory postsynaptic marker Gephyrin [18], matrix metallopeptidase 13 (MMP-13) [13], neuromodulator brain-derived neurotrophic factor (BDNF) [21], neurotransmitter and glutamate receptors N-methyl-D-aspartate receptor subunits [21], neurotransmitter serotonin 5-hydroxytryptamine [21], phosphorylated extracellular signal-regulated kinase (p-ERK) [21], cAMP response element binding protein (p-CREB) [21], toll-like receptors generally expressed in immune and glial cells to regulate innate and adaptive immunity, microglia marker CD11b [14], macrophage or microglia marker F4/80 [14], IL-4 [27], mast cell count [27], immunoglobulin E (IgE) [27], astroglia marker—glial fibrillary acidic protein (GFAP) [22], neurotransmitter glutamate [15], microglial purinergic receptor P2X7 [16], microglia-neuron modulators cathepsin S/fractalkine [16], isolectin B4-binding (IB4) (+) and IB4 (−) neurons/FM4-64 membrane-uptake marker [29], inflammatory cells (lymphocyte, monocyte, neutrophil, and plasma cells) [20], apoptosis rates of fibroblasts [25], transcriptional regulator of hypoxia-induced cellular responses—hypoxia-inducible factor (HIF-1α) [17], tyrosine hydroxylase [21], complement and microglia activation—C3, C1q, C3aR [18], lysosomal/microglia marker CD68 co-localised with IBA-1 [18], synaptic marker—excitatory postsynaptic density-95 (PSD95) [18], marker for glutamatergic neurons—excitatory presynaptic marker vesicular glutamate transporter 2 (VGlut2) [23], VGlut2 co-localised with PSD95 and dendritic spines from CA1 pyramidal neurons with C3 and C1q [18], voltage-gated sodium channels Nav isoforms [31], neuronal injury marker—activating transcription factor 3 (ATF3) [31], transient receptor potential melastatin (TRPM3, TRPM8), protein expression of transient receptor potential vanilloid (TRPV1, TRPV2, TRPV4) [23,32], and antioxidants enzymes [34].

#### 2.2.2. Botulinum Toxin Key Effect

Table 3 illustrates the summary of the findings regarding BoNT key effects on each biomarker reported in more than one of the included studies. Supplementary File S6, Table S5 shows the information for the biomarkers evaluated in only one study, and Supplementary File S7, Table S6 provides a synopsis of the methodological details and outcomes of the included studies.

Overall, regardless of methodological and characteristic variations, all the six studies evaluating IL-1β, in the context of peripheral- and neuro-inflammation, reported a significant decrease of the mRNA and/or protein expression of the biomarker after BoNT administration, when compared to vehicle administration and/or control disease models. Similar settings and results were observed for TNF-α, with only one out of five studies showing that BoNT had no effect in the peripheral release of this cytokine from the TMJ periarticular tissues and TG in a model of persistent inflammatory hypernociception (PIH) induced by rheumatoid arthritis [15].

For SNAP25 expression, mostly evaluating the toxin central effects and transport extended beyond the peripheral administration site, there were mixed results after BoNT exposure in the investigated conditions; one study reported no alterations in the hippocampus of an induced model of depression (experimental or control groups) [21], which differed from another study in trigeminal neuropathy settings, revealing a significant increase in brainstem Vc region (caudal subnucleus of the spinal trigeminal nucleus) when compared to control [23]; two studies observed cleaved SNAP25 (clSNAP25) only in the ipsilateral treated side of cranial dura and TNC, while the contralateral nontreated side was devoid of this major part of the N-ethylmaleimide-sensitive-factor attachment receptor (SNARE) complex [20,22]. A fifth study in MDD induced by chronic restrain stress showed a maximum and persistent expression of clSNAP25 in hindbrain sections, ipsilaterally to injected areas, with higher BoNT dosages [24].

The four studies evaluating neuronal activation after BoNT injection and potential central pathways involved in sensory processing, through expression of marker c-Fos in models of PIH, MDD, and trigeminal neuralgy [induced by either infraorbital nerve constriction (IoNC) or N-methyl-D-aspartate receptor (NMDA)], all reported a significant decrease in both ipsilateral and contralateral of ventrolateral periaqueductal gray, medullary dorsal horn, and TNC.

Two studies observed that the upregulation of the neuropeptide CGRP levels induced by TMD inflammatory pain and migraine models were significantly suppressed in jugular plasma and medulla oblongata containing TNC, as well as cranial dura, respectively [19,20]. In addition, another experiment reported that BoNT significantly inhibited the peripheral release of CGRP in TG and periarticular TMJ tissues induced by PIH in arthritis [15]. In contrast, the same author observed that neither the PIH model nor BoNT injection influenced the CGRP expression in TNC [22], while another experiment showed a significant increase of the biomarker in TNC evoked by a TMD inflammatory pain model, which was not altered following BoNT administration [20]. This study also revealed that the disease model, before or after BoNT, showed no significant changes in the concentration of CGRP in TG and cerebrospinal fluid sampling. Two of the studies evaluating CGRP after BoNT administration also reported on another neuropeptide (SP), having showed a significant reduction in the levels of upregulated SP in both PNS and CNS [15,19].

Two studies in trigeminal neuralgia, induced by either IoNC or compression of the trigeminal nerve root, demonstrated significant ipsilateral reduction of the upregulated proinflammatory cytokine IL-6 after BoNT treatment when compared to vehicle saline, in TG and TNC [14,17]. The CX3CR1 protein level did not differ in a trigeminal neuralgia model before or after BoNT treatment [16], but it was reported that the mRNA expression of this chemokine receptor selectively expressed in microglia was upregulated in scenarios of depression induced by chronic administration of reserpine in a Parkinson disease model, which was significantly decreased in the hippocampus in the experimental group using BoNT [18]. This suggested that the toxin may alleviate microglial activation via interaction with receptors on microglia. Two other studies in trigeminal pain induced by IoNC or Complete Freund’s Adjuvant (CFA) observed a significant reduction in induced dural protein extravasation [20,28]; however, the bilateral effect (ipsilateral and contralateral side) after BoNT was controversial. Collagen-related proteins (I,III), TGF-β1, α-SMA, and myosin II proteins were only measured in two studies inducing hypertrophic scars, and both supported that BoNT injection into tissues from the ears of rabbits remarkably reduced the expression of these biomarkers. TRPA1 protein expression was also found to be increased in models of trigeminal neuropathy induced by IoNC and chronic dry skin itch evoked by acetone–diethylether–water (AEW), which were significantly inhibited following BoNT administration.

The majority of data for single experiments found that BoNT significantly altered the biomarkers’ under/over-expression evoked by the investigated disease models and had no effect when the levels of these biomarkers were not changed by the induced chronic conditions in animals. There were a few exceptions: (1) when the toxin influenced the expression of the biomarkers that showed no significant differences between sham and disease model groups (e.g., TLR1, TLR4, TLR8); (2) when BoNT had no effect on the levels of biomarkers that were down/upregulated by the disease model (e.g., IgE, Nav 1.6, 1.8, ATF3, TRPM8); (3) when the effect of the toxin on the altered levels of the biomarkers was unclear (e.g., NR2A, TLR11).

### 2.3. Results of Synthesis

Biomarkers reported in three or more studies providing sample sizes were selected for inclusion in the meta-analysis, resulting in three eligible biomarkers: IL-1β, TNF-α, and CGRP.

All quantitative syntheses were conducted in Python 3.10 (PyCharm IDE) with statsmodels (0.14.5), SciPy (1.16.0), NumPy (2.3.1), Pandas (2.3.1), and Plotly (6.2.0). For each experiment, we calculated Hedges’ g—the standardised mean difference adjusted for small-sample bias—and its sampling variance. We prespecified an inverse-variance fixed-effect meta-analysis in which each study was weighted by the reciprocal of its variance; the pooled effect size, its standard error and 95% confidence interval were derived from these weights. Statistical heterogeneity was assessed with Cochran’s Q and summarised as I^2^. Small-study effects were investigated by visually inspecting funnel plots of effect size versus standard error and by Egger’s regression test, which regresses the standardised effect on study precision and interprets a non-zero intercept as evidence of asymmetry.

**Figure 3:**
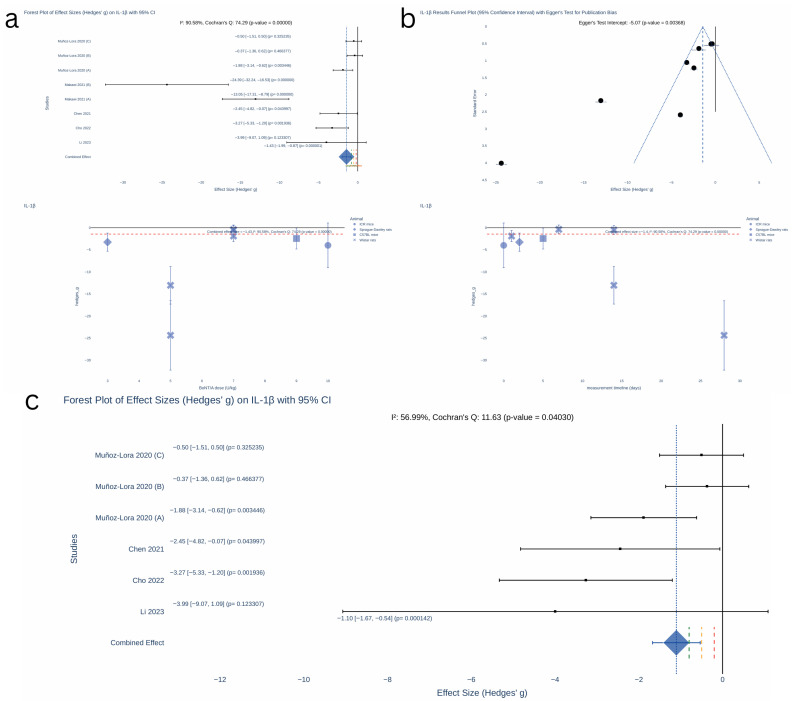
Meta-analysis IL-1β [13,14,16,17,18]. (**a**) Forest plot of effect sizes (Hedges’ g) on IL-1β with 95% CI. (**b**) IL-1β results funnel plot (95% CI) with Egger’s test for publication bias. (**c**) Forest plot of effect sizes (Hedges’ g) on IL-1β with 95% CI (excluding study with relatively high heterogeneity [13]). KEY: red—small effect size threshold (0.2); yellow—moderate effect size threshold (0.5); green—large effect size threshold; blue—combine effect size.

The meta-analysis using Hedges’ g revealed a significant effect of BoNT on IL-1β levels (Figure 3), with a pooled effect size of −1.43 (95% confidence interval (CI): [−1.99, −0.87], *p* < 0.00001). However, large heterogeneity was observed (Cochran’s Q = 74.29, *p* < 0.000001; I^2^ = 90.58%) and significant publication bias was detected (Egger’s test intercept = −5.07, *p* = 0.00368).

After excluding the study contributing to high heterogeneity, the effect size decreased to −1.1 (95% CI: [−1.67, −0.54], *p* < 0.001). While heterogeneity remained significant (Cochran’s Q = 11.63, *p* = 0.04030), it was reduced to a moderate level (I^2^ = 56.99%).

There was no individual-level data available to assess the impact of the timing of measurements on effect size. However, visual inspection suggests a trend toward a reduced magnitude of effect when IL-1β levels were measured at later time points. Similarly, no clear correlation between effect size and dose was evident based on the scatterplot analysis.

**Figure 4:**
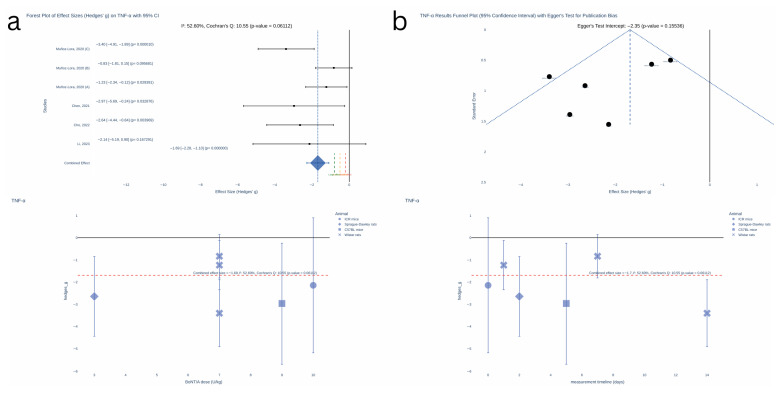
Meta-analysis TNF-α [14,16,17,18]. (**a**) Forest plot of effect sizes (Hedges’ g) on TNF-α with 95% CI. (**b**) TNF-α results funnel plot (95% CI) with Egger’s test for publication bias. KEY: red—small effect size threshold (0.2); yellow—moderate effect size threshold (0.5); green—large effect size threshold; blue—combine effect size.

The meta-analysis revealed a significant effect of BoNT on TNF-α levels (Figure 4), with a pooled effect size of −1.69 (95% CI: [−2.28, −1.10], *p* < 0.000001) with non-significant heterogeneity observed at moderate level (Cochran’s Q = 10.55, *p* = 0.06112; I^2^ = 52.60%) and non-significant publication bias (Egger’s test intercept = −2.35, *p* = 0.15536).

Similarly, no clear correlation between effect size and dose or measurement time was evident based on the scatterplot analysis.

The CGRP analysis was excluded entirely due to significant and substantial heterogeneity (Cochran’s Q = 44.76, *p* < 0.000001; I^2^ = 91.06%) and the presence of significant publication bias (Egger’s test intercept = −6.05, *p* < 0.0001).

#### Sample Size for Future Studies

Given the large heterogeneity observed in the meta-analysis results, we adopted a cautious approach to interpreting the effect size estimates. Conservative effect size values of −1 for IL-1β and −1.5 for TNF-α were used, instead of the original estimates of −1.1 and −1.69, respectively. A one-tailed test was applied, as the evidence strongly supported a directional hypothesis indicating a negative effect size (i.e., BoNT reduces IL-1β and TNF-α levels compared to the control group).

$$\begin{array}{c} n=\frac{2\times {\left(Z_{\alpha /2}+Z_{\beta }\right)}^{2}}{g^{2}} \end{array}$$

Using these conservative effect size estimates, power 80%, and significance level 5% (one-tailed), the required sample size calculations yielded *n* = 26 for IL-1β (13 per group) and *n* = 12 for TNF-α (6 per group).

### 2.4. Certainty of Evidence

**Figure 5:**
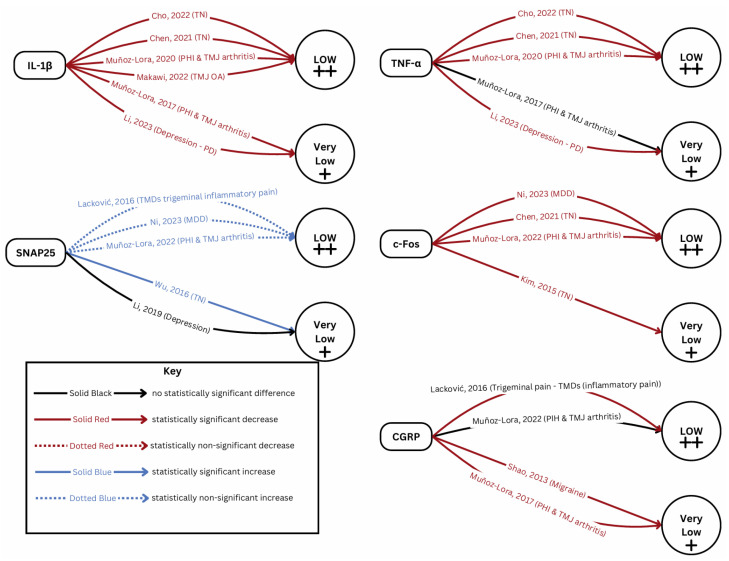
GRADE assessment [13,14,15,16,17,18,19,20,21,22,23,24,26].

Figure 5 provides a snapshot of the GRADE assessment results. The summary of findings tables show the certainty of evidence assessments for the biomarkers reported more than once (Table 3) and single experiments (Supplementary File S6, Table S5). Supplementary File S8 provides the explanations for the judgements. In short, very low to low-certainty evidence was found for the effects of BoNT on the investigated biomarkers in preclinical research.

## 3. Discussion

From this review, it seems that BoNT leads to a significant suppression of the severity of many induced disease models by targeting specific biological markers. However, while animal intervention studies still play an important role in human health care research, our findings seem to be in line with current evidence on common pitfalls of animal experiments. For example, Bahadoran et al. (2020) found that in animal studies, diseases are frequently induced with unclear concordance to human conditions [11]. More findings that were also consistent with our results include the great variation in animal characteristics and species studied, diversity of experimental designs, several models for induction of diseases, as well as differences in doses of drugs, timing, and delivery method protocols. Furthermore, the use of inferior models, with smaller dimensions, precluded the application of recommended protocols for BoNT use in humans and approved conditions such as chronic migraine [19,20]. In line with our study, Bahadoran et al. (2020) also did not find that it the registration of animal studies and to include or adequately report experimental details is yet common practice, including random allocation of animals and/or blinded assessment of the outcomes [11]. We add that there is also a lack of information, whereas allocation to the different groups was reasonably concealed.

These shortcomings may result in a lack of internal and external validity, as well as in challenges to conducting meta-analysis and extrapolating animal models to human clinical conditions. Nevertheless, as previously outlined, the authors conducted a systematic review of human studies on the same topic prior to commencing this study [7]. We found that CGRP was the most commonly examined biomarker in human trials on BoNT effects on inflammation-associated chronic conditions. Other biomarkers used in clinical studies included different immune cell classes, as well as several important players in inflammatory processes, serotonin, oxidative stress biomarkers, SNAP25, and SP. These appear to be largely in line with the biomarkers reported in the animal studies from this review. However, while for clinical research the most-studied scenario was the prevention of chronic migraine, with only one study focusing on trigeminal neuralgia, in preclinical research we observed the opposite. Additionally, although we adapted the eligibility criteria from the systematic review on clinical research, we found within the current study that twelve animal experiments explored other conditions that also fit the inclusion criteria namely, TMJ arthritis such as TMJ osteoarthritis and PHI in TMJ rheumatoid arthritis, anxiety, depression and MDD, hypertrophic scars, atopic dermatitis, and chronic dry skin itch conditions.

In addition, findings from this review suggest that several other biomarkers may be implicated in the pathogenesis of the studied chronic conditions, and hence these were considered important to monitor the progression of certain diseases linked to inflammation and the effects of BoNT intervention. Overall, data found that BoNT significantly altered the biomarkers’ under/over-expression evoked by the investigated disease models (e.g., IL-1β, IL-6, c-Fos, TRPA1, dural protein extravasation, collagen (I,III), TGF-β1, α-SMA, and myosin II proteins) and had no effect when the levels of these biomarkers were not changed by the induced chronic conditions in animals (e.g., CX3CR1).

Meta-analysis results also revealed a significant effect of BoNT on TNF-α levels. However, it is important to note that the evidence contained in our meta-analysis was based on quantitative studies providing sample sizes, which resulted in the exclusion of one study showing that BoNT had no significant effect on TNF-α levels in the peri-articular tissues from TMJ and TG. These results suggest that following the peripheral administration of BoNT, the negative effect on the levels of proinflammatory cytokines may contribute to improve the investigated conditions (trigeminal neuralgia with neuropathic pain and anxiety-like behaviours, depression in Parkinson’s disease and PIH and TMJ rheumatoid arthritis), but the evidence appear to be more consistent for the cytokine changes in the CNS rather than at the injection site or sensory ganglia.

There were also mixed results after BoNT administration in the investigated conditions for SNAP25, and CGRP. The distribution of cleaved SNAP25 confirmed the axonal transport of BoNT into the CNS after peripheral injection. However, this fuels the debate about whether BoNT can be transported to higher structures, like the hippocampus, or register endoproteolytic activity within the TNC contralateral non-injected side, suggesting the relevance of transcytosis involving second-order central synapses for the antinociceptive effect of BoNT. With regards to CGRP, the results showed that trigeminal pain induced in different animal species (e.g., Sprague Dawley and Wistar rats) and using different techniques may not result in a consistent significant increase of the neuropeptide in TG or TNC. Moreover, BoNT may not consistently block overexpression of CGRP in the TNC under all conditions, which may suggest that different stimuli may activate relevant pathways less susceptible to BoNT’s inhibitory effects.

There were a few other exceptions among single experiments: (1) when the toxin influenced the expression of the biomarkers that showed no significant differences between sham and disease model groups; (2) when BoNT had no effect on the levels of biomarkers that were down/upregulated by the disease model; (3) when the effect of the toxin on the altered levels of the biomarkers was unclear.

The heterogeneity observed across all analyses can result from several factors, including variations in chronic conditions, biological sampling, use of different models for induction of diseases and animals for the experiments (species, weight, sex, and age), inconsistencies in the timing of biomarker measurements, different doses administered and the methods or purpose for measuring the biomarkers. In addition, to highlight that we should exercise caution when interpreting these findings, given that the GRADE assessment showed very low to low certainty of evidence for the reported BoNT significant effects on each assessed biomarker. Moreover, Eager’s test and funnel plots showed significant evidence of publication bias for IL-1β and CGRP but not for TNF-α.

### 3.1. Limitations

One of the primary limitations was the high heterogeneity of data from several animal experiments subjected to bias. Most studies favoured male animals over female or failed to disclose the sex of the animals investigated, underestimating sex-related physiologic differences confounding experimental results owning to distinct disease predisposition, prognosis, and response to BoNT (e.g., effect of sex hormones on nociceptive thresholds and biomarker levels). In contrast, with the advancements in clinical research reporting and designs, the gender bias was prevalent. Considering the important sex differences in varied aspects of brain functioning, data obtained exclusively from male animals limits the possibility of female-specific therapeutics advancing and translation. The evident paradox is that women are more vulnerable to the highly prevalent neurologic and psychiatric disorders reported in the included studies. Besides the susceptibility to develop certain conditions, sex differences extend to the presentation, the course of the illness and/or treatment effectiveness. Similarly, when reported, there were differences in age among the animals studied. Considering that the disease phenotype and response to therapies can be affected by the age, this contributes to increased heterogeneity of the studies. Another important source of bias was the variations in study design and methods. For example, we cannot exclude the overestimation of treatment effect, which may result from the lack of information on randomization, allocation concealment, or blinded outcomes assessment. Moreover, although studies showed significant results, indicating a sufficient sample size, they are based on male inferior animals, which limits the recommendations for clinical trials. Specifically, there was a preponderance of male rodents, and although these have been widely used in preclinical research, whether rats and mice are reliable models to study chronic inflammation remains controversial. Bearing in mind that publication bias in the systematic reviews of animal studies may severely affect its validity, we have attempted to conduct a comprehensive and systematic search. For that, we have defined and prespecified a research question focusing on inflammation-associated chronic conditions, and we set the eligibility criteria to exclude studies in disorders related to muscles and glands hyperactivity. However, the pathophysiology of many conditions to which BoNT treatment has been proposed is complex and we may have excluded diseases of interest and able to amplify the results. For example, we have excluded movement disorders of the nervous system such as Parkinson’s disease. However, recent studies have suggested that this progressive neurodegenerative disease is most likely the result of inflammatory pathways malfunction linked to genetic vulnerability and immune deregulation caused by ageing and the activation of glia from neuronal insult [42]. Thus, a possible effective treatment of Parkinson’s disease through BoNT anti-neuroinflammatory properties rather than only its actions at the typical associated motor symptoms of tremors, bradykinesia, and rigidity via transient paralysis of the treated muscles is hinted at [43]. Moreover, we have narrowed the searches to head and neck conditions in an attempt to limit the heterogeneity of chronic conditions and biological sampling across studies to enhance interpretability and obtain a meaningful summary effect. However, we may have excluded relevant disease models (e.g., psoriasis) illustrated in the Supplementary File S2, Table S1. Finally, most of the included studies did not present results in tabular or numerical formats but instead relied on graphical plots. To extract data from these plots, ImageJ software (1.54k 15 September 2024) was used to estimate numerical values. While we acknowledge that the extracted data may not be perfectly precise, we believe the impact of this limitation on accuracy is negligible due to the reliability of ImageJ.

### 3.2. Future Directions

There are a few important questions worth further exploration. For example, considering the results from this review and the findings of a recent clinical trial showing the anti-inflammatory effects of BoNT in participants suffering from chronic bilateral occipital headaches [44], it would be important to clarify the potential of BoNT to regulate the expression of proinflammatory cytokines and peripheral inflammation at the injection site, as well as the contribution of such effects to improve the condition.

Overall, the exact mechanisms underlying BoNT effects that appear to be independent from motor function remain to be determined. Exploring these challenges may reveal critical aspects about how the toxin functions and lead to the discovery of new scientific perspectives on its molecular processes and systemic events. Greater knowledge on how BoNT acts at multiple levels could help with promoting new strategies to tackle many disorders.

We suggest conducting more high-quality preclinical studies using evidence-based disease models to assess biological plausibility, regardless of whether the result is negative or positive. We recommend considering important biological variables such as age and sex, as well as better reporting, providing greater transparency in the animal experiments (e.g., making use of recommended tools for quality assessment, following available reporting guidelines, registration of studies, and data accessibility). This may be conducive to improving the translational success rate and benefit humans.

## 4. Conclusions

This study demonstrated that ongoing animal interventional studies have introduced various potential reliable disease models and evidence-based biochemical biomarkers that may contribute to the pathophysiology of many inflammation-associated long-term conditions. Moreover, the results provide some new theoretical bases for treating many chronic conditions with BoNT. Despite a few exceptions, generally, data found that BoNT significantly altered the biomarkers’ under/over-expression and had no effect when the levels of these biomarkers were not changed by the induced chronic conditions linked to inflammation in animals. Finally, the meta-analysis revealed a significant negative effect of BoNT on TNF-α levels in the sensory ganglia and CNS, with neither significant heterogeneity nor publication bias.

## 5. Materials and Methods

The methodology used previously by our group for the systematic review of clinical trials was adapted for animal intervention studies [7]. We followed the Preferred Reporting Items for Systematic Reviews and Meta-Analyses (PRISMA) guidelines for reporting this review (see Supplementary File S9 for PRISMA 2020 checklist) [41]. The PICO framework was used as follows:

(P)opulation: animals—disease model of chronic conditions in the head and neck, linked to inflammation (traditional, neurogenic inflammation, neuroinflammation); (I)ntervention: botulinum toxin (BoNT); (C)omparisons: according to each specific chronic condition and between the biochemical biomarkers and unit of measurement that have been investigated; (O)utcomes: change (increase, decrease) or no change in biomarkers level.

### 5.1. Inclusion and Exclusion Criteria

All published studies with, or without, a control group were included, regardless of the sample size. We excluded all systematic reviews and meta-analysis, review articles, conference abstracts, editorials, and letters. There were no language restrictions, nor were studies excluded on the basis of the date or the journal of publication.

To be included, the studies had to follow the eligibility criteria: (1) use BoNT (any preparation, dose, delivery method, and administration site), (2) be conducted on animals and without species, age, and sex restrictions, (3) involve chronic conditions that have been related to inflammation, and/or neurogenic inflammation, and/or neuroinflammation, (4) restricted to the head and neck, (5) include biochemical biomarkers to assess the outcomes.

In attempts to limit the heterogeneity of chronic conditions, disease models, and biological sampling across studies, to enhance interpretability and obtain a meaningful summary effect, we focused on head and neck inflammation-associated chronic conditions such as migraine, orofacial pain, arthritis, trigeminal neuralgia, herpetic neuralgia, myofascial pain, temporomandibular joint pain, cancer, periodontitis, anxiety, depression, chronic stress, traumatic brain injury, ocular pain, dermatitis, rosacea, psoriasis, keloids or hypertrophic scars, cephalalgia, and hair loss. The models for the induction of disease should have had evidence of validity and reliability, and studies were excluded on this basis.

The studies were excluded as follows: in silico disease models, in vitro and human studies, evaluating BoNT in healthy models, or on induced acute conditions, or when the chronicity status was unclear, studies focusing on muscles and glands hyperactivity, with targets or assessments below head and neck, when considering combined approaches or other therapies rather than BoNT, studies assessing BoNT effects without biochemical biomarkers, or when these assessments were not reported after BoNT intervention.

### 5.2. Search—Sources and Strategy

We conducted electronic searches up to 29 January 2024 and within the PubMed, Web of Science, and Scopus databases. In addition, we performed searches in the animalstudyregistry.org “https://search.animalstudyregistry.org/” (accessed on 1 and 29 January 2024), preclinicaltrials.eu “ https://preclinicaltrials.eu/database” (accessed on 1 and 29 January 2024), and the International Prospective Register of Systematic Reviews PROSPERO “www.crd.york.ac.uk/prospero/” (accessed on 1 and 29 January). We also handsearched reference lists of included and excluded studies to locate additional studies. The Supplementary File S10 provides the full search strategies followed by two reviewers (I.N.P., S.D.).

### 5.3. Selection Process

The titles and abstracts of all papers retrieved were reviewed, independently and blinded, by two reviewers (I.N.P., S.D.). When consensus in the initial screening could not be attained, a third reviewer (G.D.C.) made the final judgement. Then, two reviewers (I.N.P., S.D.) independently screened the full text of the possibly relevant articles initially identified. When necessary, consensus was reached on inclusion or exclusion by discussion and/or by consulting the third reviewer (G.D.C.). The titles and abstracts retrieved by the searches were imported to a software platform (Rayyan). Duplicate records identified were removed, employing the Rayyan automatic de-duplication option, and after being manually reviewed (see Supplementary File S1 for full Rayyanreport). Google Translate was used to decide potential eligibility when non-English language articles were found.

### 5.4. Data Collection

We developed a standardised data-extraction table, which two reviewers (I.N.P., S.D.) tried out on four randomly selected studies. Two reviewers worked independently to extract data. Any conflicts were resolved by a third or fourth reviewer.

When information regarding the primary objective was incomplete, we attempted to contact the corresponding authors as predefined within the protocol (by web e-mail and a maximum of three attempts, at 10-day intervals).

We collected data on the first author’s name, year of publication, study design, funding sources, and/or funder involvement. We also extracted information regarding the characteristics of included studies and results as follows: population specifics including sample size, species, sex, and age demographics, disease modelled characteristics (induction method, when reported), any information relating to the BoNT administration (anatomical site of intervention, mode of delivery, dose, and preparation), and which biochemical biomarkers have been evaluated (baseline and outcome assessments), with unit and follow-up timing specifications, as well as biological sampling or specimens.

If data were available, we collected outcome event rates for each comparison group (dichotomous outcomes) and mean changes, or differences in final assessments against initial measurements, with any associated statistics (continuous outcomes). Where trialists reported other outcome measures of intervention effectiveness, we also extracted relevant data (e.g., aggravation, improvement, or no change in condition), and any other important aspects within the context of each study. In our analysis, we systematically documented “Not Reported” (NR) when a specific data point was missing. In addition, if trialists measured an outcome at multiple sites or subclasses, we extracted information relating to the site or subclass where the effect was highest. Finally, where data were presented in graphs, they were measured using the ImageJ software.

### 5.5. Study Characteristics, Risk of Bias, and Certainty Assessment

To evaluate the full methodology for all the included studies and assess the risk of bias, we designed the question list taken from the Systematic Review Centre for Laboratory animal Experimentation (SYRCLE’s) risk of bias tool for animal studies [45]. Two researchers independently applied the tool to each study and, at each stage, recorded the rationale for article appraisals. With regard to the assessment of the risk of bias, each domain was judged “low”, “unclear”, or “high”. Any conflicts in judgements or justifications for the risk of bias were resolved by discussion or with adjudication of a third and fourth reviewer if necessary. A summary “Risk of bias” judgement table was created.

To assess the certainty of the body of evidence for each reported biomarker and BoNT key effect, we followed the five Grading of Recommendations Assessment, Development, and Evaluation (GRADE) rating approach [46]. The certainty of evidence was judged as very low, low, moderate, or high. We have explained and recorded, in the summary of findings tables, all the decisions to up- or down-grade the certainty of studies. It was noteworthy that it is not recommended to calculate an overall risk of bias score for each individual study when using SYRCLE’s tool [47]. Therefore, for the GRADE domain 1 (risk of bias), we decreased the level of certainty one level for studies rated as having a high risk of bias in at least one domain, or unclear if there was a lack of concealment of randomization and/or lack of blinding. We downgraded the studies rated high risk of bias due to lack of concealment of randomization or lack of blinding by two levels.

### 5.6. Effect Measures and Synthesis Methods

Following the prespecified protocol [48], the evidence was synthesised narratively, and different tables were designed to provide a visual overview, making it possible to combine data across studies and to try to decide on the eligibility for conducting a meta-analysis. Studies that reported biomarker results without providing sample sizes were excluded from the quantitative analysis.

We planned to assess potential sources of effect heterogeneity as follows: (1) At least more than two studies would be necessary per biomarker to build a forest plot. The studies would be combined using a random effects forest plot. (2) The standardized mean difference, standard deviation, and the 95% confidence interval to be calculated for each biomarker reported in the included studies. The effect size to be calculated using Hedge’s g with a value of 0.2, 0.5, and 0.8 considered small, medium, and large, respectively. (3) The heterogeneity to be calculated using the Cochran’s Q test for significance and I^2^ test for magnitude, with a value of 25%, 50%, and 75% considered low, moderate, and high, respectively.

### 5.7. Registration and Protocol

The protocol of this systematic review was registered in the international prospective register of systematic reviews (PROSPERO) database (registration number: CRD42023432411) [48]. Deviations from the published protocol are as follows: (1) Inclusion of “systematic review” in the title to follow PRISMA 2020 guidelines. (2) We added to the search strategy other sources such as trials and systematic reviews registries. (3) Given the poor definition of chronicity in the available disease models, the eligibility criteria was updated, and we excluded studies when it was unclear if they were related to acute or chronic conditions. (4) We have excluded studies evaluating combined therapies, except when data were available for BoNT key effects only. (5) We have excluded disease models (or assessments) reported below head and neck. (6) To assess the quality of evidence for each pre-specified outcome, we considered the GRADE approach. (7) Given the number of studies presenting measurements only in graphs, we have attempted to extract data with ImageJ software. (8) We have obtained the collaboration of an additional author.

## Figures and Tables

**Table 1:** Included studies—characteristics.

Author	Sample	Group/Sample	Biological Sampling	Chronic Condition	Species	Age	Sex	Weight	Follow-Up
Makawi, 2022 [13]	*n* = 42	4/(*n* = 14) × 3 + (*n* = 3)	TMJ tissues	TMJ osteoarthritis monosodium iodoacetate	Wister albino rats	3–4 months	Male	(180–200 g)	2, 4 weeks
Chen, 2021 [14]	*n* = 48	4/(*n* = 12) × 2 + (*n* = 18) + (*n* = 6)	TNC	TN IoNC and anxiety-like behaviors	C57BL/6 mice	6–8 weeks	Male	(20 g)	5 days
Muñoz-Lora, 2017 [15]	?	5/?	Peri-articular tissues from TMJ and TG	PIH systemic immunization—mBSA/PBS+CFA and TMJ rheumatoid arthritis mBSA + formalin (0.5%)	Wistar rats	?	Male	(250–500 g)	24 h or 14 days
Muñoz-Lora, 2020 [16]	*n* = 40	5/(*n* = 8) × 5	Trigeminal subnucleus caudalis	PIH systemic immunization—mBSA/PBS+CFA and TMJ rheumatoid arthritis mBSA + formalin (0.5%)	Wistar rats	?	Male	(300–400 g)	24 h, 7 or 14 days
Cho, 2022 [17]	*n* = 236	5/(*n* = 6) × 5	TG	TN compression of the trigeminal nerve root	Sprague Dawley rats	?	Male	(250–280 g)	2 days
Li, 2023 [18]	?	4/(*n* = 3, 5–6) (*n* = 3) protein, %, fluorescence intensity, volume (*n* = 5–6) mRNA	Brain—Substantia nigra pars compacta and hippocampus	Depression reserpine chronic administration in Parkinson’s disease	Parkinson disease model-ICR mice	6–8 weeks	Male	(30 g)	?
Shao, 2013 [19]	*n* = 32	4/(*n* = 8) × 4	Jugular plasma and medulla oblongata—containing caudal trigeminal nucleus	Migraine NTG	Sprague Dawley rats	?	Female	(250–300 g)	24 h
Lacković, 2016 [20]	*n* = 105	3/(*n* = 6) × 3—CGRP 5/(*n* = 5–9) × 5—dura 3/(*n* = 5–8) × 3—dura 3/(*n* = 5—cell profiles (*n* = 4)—SNAP-25	Dura mater, TNC, TG, Cerebrospinal fluid sampling	Trigeminal pain—temporomandibular disorders (inflammatory pain) CFA	Wistar rats	3–3.5 months	Male	(300–350 g)	4 days
Li, 2019 [21]	?	5/(*n* = 6)—(naive) protein 4/(*n* = 5–6)—(naive) mRNA 7/(*n* = 6) —Tx protein 6/(*n* = 6) —Tx mRNA 3/(*n* = 6–7)—naive 6/(*n* = 6–7)—Tx	Hippocampus, hypothalamus, prefrontal cortex, amygdala (Brain)	Depression SRS	ICR mice	6–8 weeks	Male	(20–25 g)	1, 7, 14 days
Muñoz-Lora, 2022 [22]	*n* = 40	6/(*n* = 5)—c-fos 8/(*n* = 5)—CGRP, GFAP ?/(*n* = 3)—SNAP25	TNC	PIH systemic immunization—mBSA/PBS+CFA and TMJ rheumatoid arthritis mBSA + formalin (0.5%)	Sprague Dawley rats	6–8 weeks	Male	(300–400 g)	day 14
Wu, 2016 [23]	?	4/(*n* = 6)—SNAP-25 7/(*n* = 6)—TRPs	Brainstem Vc region (caudal subnucleus of the spinal trigeminal nucleus)	TN IoNC	Sprague Dawley rats	?	Male	(220–300 g)	7 days
Ni, 2023 [24]	?	6/(*n* = 4 cells from 3 mice) × 3; (*n* = 4 or 5 or 6 brain sections from 3 mice) × 3—SNAP25 3/(*n* = 3 mice)—c-fos	Brain	Major depressive disorder chronic restraint stress	pathogen-free C57BL/6J mice	8 weeks	Male	(25 g)	24 h after day 23 to 27
Wang, 2020 [25]	*n* = 18	4/(*n* = 36) ears 6 wound/per 36 ears (*n* = 216) wounds	Scar tissue (ear)	Hypertrophic scar lesion/wound	New Zealand big-ear albino rabbits	? (mature)	?	(2.5~3.5 kg)	day 28 and day 60
Kim, 2015 [26]	?	4/(*n* = 5) × 4	medullary dorsal horn	Trigeminal nociception (orofacial inflammatory pain) formalin; (chronic pain/inflammation); CFA; NMDA	Sprague Dawley rats	?	Male	(230–280 g)	?
Han, 2017 [27]	*n* = 42	6/(*n* = 6) × 4, (*n* = 9) × 2	Rostral dorsal Skin and Serum—retro orbital plexus	Atopic Dermatitis NC/Nga + contact sensitizer (TNCB)	NC/Nga mice	6 weeks	Female	?	day 14 after 1st challenge
Filipović, 2012 [28]	*n* = 200	4/(*n* = 20)	Cranial dura Plasma protein complexes	TN IoNC (with/without formalin)	Wistar rats	?	Male	(300–350 g)	3 days
Kitamura 2009 [29]	?	4/(*n* = 11), (*n* = 9), (*n* = 8) × 2—IB4+ 4/(*n* = 10), (*n* = 9), (*n* = 8) × 2—IB4−	TG sensory neurons	TN IoNC	Sprague Dawley rats	? (adult)	Male	(200–250 g)	11 days
Xiong, 2023 [30]	*n* = 24	4/(*n* = 6) × 4	Ear tissue—scar-related	Hypertrophic scar lesion/wound	New Zealand white rabbits	6 months	Female	(3.0~3.3 kg)	5 weeks
Yang, 2016 [31]	?	3/(*n* = 5) × 3	nerve-injured TG (mandibular (V3) division & boundary area)	Trigeminal neuropathic pain malpositioned dental implants	Sprague Dawley rats	?	Male	(220 and 240 g)	6 days
Zhang, 2019 [32]	?	4/(*n* = ?)	trigeminal spinal subnucleus caudalis	TN IoNC	Sprague Dawley rats	? (adult)	Male	(200–250 g)	7 days
Cao, 2017 [33]	*n* = 525	6?/(*n* = 6–7) per group?	Dorsal root ganglia	Chronic dry skin itch models AEW	CD1 (ICR) mice	6–8 weeks	Male	?	30 min, 1, 3, 7, 14 days
Yesudhas 2021 [34]	*n* = 12	2/(*n* = 6) × 2	Hippocampus (brain) tissues—total protein isolates	Anxiety and ageing	ageing model-BALB/c mice	7–8 months	Male	?	30 days

Key: ?, missing information.

**Table 2:** Biomarkers in preclinical research. Botulinum Toxin (BoNT) effects in head and neck (H&N) Chronic Inflammatory State (CIS).

Biomarkers	CIS	Author Year	Biological Sampling	Biomarker vs. BoNT	Biomarker vs. CIS	H&N Clinical Trials?
6 STUDIES						
IL-1β PRO-INFLAMMATORY CYTOKINE (ASSOCIATED TO PERIPHERAL INFLAMMATION AND NEUROINFLAMMATION)	TN	Chen, 2021 [14]	TNC	Peripherally administered BoNT induces modulation of neuronal and non-neuronal components in the nociceptive pathways. It may inhibit the overexpression of microglia-derived pro-inflammatory factors (e.g., IL-1β) and neuroinflammation.	Interplay of immune–neuronal–non-neuronal cells is crucial for the development and maintenance of chronic pain states, characterized by sensitization of the CNS. Microglia cells are essential in central sensitization and plasticity, stimulating the neuronal release of inflammatory mediators in response to injury. Neuroinflammation may be involved in the pathophysiology of TN and depression in PD. IL-1β is a mediator of inflammation with a key role in neuroinflammation and inflammatory diseases (e.g, important marker to monitor the progression of OA and TMJ arthritis, once IL-1β increased production leads to cartilage and connective tissue degradation).	√
	PHI and TMJ arthritis	Muñoz-Lora, 2020 [16]				
	Depression—PD	Li, 2023 [18]	Hippocampus			
	TMJ OA	Makawi, 2022 [13]	TMJ tissues	Peripherally administered BoNT may decrease peripheral pro-inflammatory cell infiltrate (at the injection site and sensory ganglia), potentially: 1. BoNT is taken up by sensory nerve terminals inhibiting peripheral release of neuropeptides, neurotransmitters, and transcription factors that reduce the inflammatory chemotaxis—neurogenic inflammation; 2. altered activation of glia in sensory ganglia via direct regulation of glia or mediated by regulation of sensory neurons through the prevention of neuropeptides and neurotransmitters release, 3. direct effect on immune cells at the injection site—anti-inflammatory effects.		
	PIH and TMJ arthritis	Muñoz-Lora, 2017 [15]	Peri-articular tissues from TMJ and TG			
	TN	Cho, 2022 [17]	TG			
5 STUDIES						
TNF-α PRO-INFLAMMATORY CYTOKINE (ASSOCIATED TO PERIPHERAL INFLAMMATION AND NEUROINFLAMMATION)	TN	Chen, 2021 [14]	TNC	Peripherally delivered BoNT is transsynaptically or transcytotically transported from primary afferents to glia within the CNS or postsynaptic neurons and induce central changes like reduced activation of microglia and microglia-derived pro-inflammatory factors (e.g., TNF-α) and neuroinflammation.	TNF-α mediates inflammatory diseases and plays a key role in neuroinflammation and neuropathic pain processes. It can activate other cytokines (e.g., IL-1). Neurotrophic factors and neuroinflammation are involved in the pathophysiology of depression in PD. TNF-α has been associated with cognition, depression, and disability. TNF-α high levels in the synovial fluid of TMJs has been related to TMJ disorders, joint inflammation, pain, and connective tissue destruction.	√
	PHI and TMJ arthritis	Muñoz-Lora, 2020 [16]				
	Depression—PD	Li, 2023 [18]	Hippocampus			
	PIH and TMJ arthritis	Muñoz-Lora, 2017 [15]	Peri-articular tissues from TMJ and TG	BoNT may decrease peripheral pro-inflammatory cell infiltrate in the periphery, which may reduce local neurogenic inflammation.		
	TN	Cho, 2022 [17]	TG			
SNAP25 PROTEIN/COMPONENT OF THE SNARE COMPLEX Cleaved SNAP-25—MARKER OF BoNT PROTEOLYTIC ACTIVITY AND PRESENCE OF ACTIVE BoNT (ASSOCIATED TO NEUROPLASTICITY—synaptic plasticity and neurotransmission)	Depression	Li, 2019 [21]	Hippocampus	BoNT cleaves SNAP25, blocking the release of neurotransmitters and neuropeptides with effects in the PNS and CNS. Central effects of BoNT may be induced by sensory input from the PNS, retrograde axonal transport to upper sensory regions (sensory ganglia and central terminals) or direct injection into the CNS (may have receptors and targets for BoNT). Changes in the CNS (e.g., reduced phosphorylation of glutamate receptors in second-order neurons, decreased microglia-activation, contralateral localization, and cortical reorganization), may be related to alterations in primary afferents or directly mediated by transsynaptic, transcytosis, or bloodstream transport. BoNT antinociceptive activity on the PIH may be related to central effects. In depression, one of the prevailing theories include modulation of neuroinflammation.	SNAP25—essential in cellular processes: exocytosis and neurotransmitter release, secretory vesicle extravasation, synaptic messaging, ion channel opening (mostly calcium channels), intercellular signalling, and has also been involved in promoting normal vesicle fusion and coordinating lysosomal trafficking. SNAP25 may be involved in regulating short-term plasticity at synapses. Low SNAP25 levels have been linked with depression and may be implicated in the pathophysiology of MDD. TMJ rheumatoid arthritis is a chronic inflammatory condition associated with a variety of systemic and local proinflammatory mediators (e.g., neurotransmitters). The related inflammation can cause pain, swelling, and limited movement in the TMJ.	√
	PIH and TMJ arthritis	Muñoz-Lora, 2022 [22]	TNC			
	TMDs trigeminal inflammatory pain	Lacković, 2016 [20]	Cranial dura			
	MDD	Ni, 2023 [24]	Brain—hindbrain sections			
	TN	Wu, 2016 [23]	Brainstem Vc region (caudal subnucleus of the spinal trigeminal nucleus)			
4 STUDIES						
c-Fos CELL ACTIVATION MARKER (mostly used for NEURONAL ACTIVITY, but can also be expressed in GLIA CELLS)	TN	Chen, 2021 [14]	TNC	Following BoNT administration, c-Fos expression may represent the activation of central pathways. BoNT is retrogradely transported to upper regions and reduces the expression of ion channels or cytokines from sensory ganglia, neuropeptides and neurotransmitter release from primary afferent central terminals, which may result in reduced central sensitization in the dorsal horn of the spinal cord or TNuc.	In the context of TMJ rheumatoid arthritis, TN, MDD, c-Fos expression has been used as a correlate of neuronal activity to map central pathways involved in sensory processing, including nociception, or to confirm whether trigeminal neurons participate in antinociception.	Used in animals (mostly postmortem) and cultured human cells
	PHI and TMJ arthritis	Muñoz-Lora, 2022 [22]				
	MDD	Ni, 2023 [24]	Brain—hindbrain sections (vlPAG)			
	TN	Kim, 2015 [26]	Medullary dorsal horn			
CGRP NEUROPEPTIDE (CAN ACT AS EXCITATORY NEUROTRANSMITTER)	Migraine	Shao, 2013 [19]	Jugular plasma and medulla oblongata—containing caudal trigeminal nucleus	BoNT may block secretion of CGRP and other neuropeptides from nerve terminals: 1. peripheral terminals of primary afferents at the injection site, 2. central terminals of primary afferents, likely owning to their release-dependence on the SNARE complex. The effect of BoNT may involve the suppression of neuropeptide release, which has a central role in neurogenic inflammation and neuroinflammation-associated conditions.	CGRP—regulatory neuropeptide is strongly involved in the pathology of migraines and deemed a key mediator of trigeminal sensitization. In trigeminal neuralgia, high levels of CGRP suggest a link between CGRP and the condition. Many other conditions (e.g., chronic pain, neuropathic disorders, musculoskeletal pain) depend on the release of excitatory neurotransmitters. Higher release of CGRP is involved in inflammatory conditions.	√
	PIH and TMJ arthritis	Muñoz-Lora, 2022 [22]	TNC			
	PIH and TMJ arthritis	Muñoz-Lora, 2017 [15]	Peri-articular tissues (TMJ) & TG			
	Trigeminal inflammatory pain—TMDs	Lacković, 2016 [20]	Dura mater, TNC, TG, CSF			
2 STUDIES						
SP NEUROPEPTIDE AND NEUROTRANSMITTER	Migraine	Shao, 2013 [19]	Jugular plasma and medulla oblongata—containing caudal trigeminal nucleus	BoNT may block the release of SP and other neuropeptides in both CNS and PNS, likely owing to their release-dependence on the SNARE complex. The effect of BoNT may involve the inhibition of the neuropeptide and neurotransmitter release, being essential in neurogenic inflammation and neuroinflammation-associated conditions.	SP is synthesized in the dorsal root ganglia sensory neurons and transported both centrally and peripherally, playing a pivotal role in pain neurotransmission. Higher release of SP is involved in inflammatory conditions.	√
	PIH and TMJ arthritis	Muñoz-Lora, 2017 [15]	Peri-articular tissues (TMJ) & TG			
IL-6 PRO- and ANTI-INFLAMMATORY CYTOKINE (ASSOCIATED TO PERIPHERAL INFLAMMATION AND NEUROINFLAMMATION)	TN	Cho, 2022 [17]	TG	BoNT may decrease peripheral pro-inflammatory cell infiltrate (potentially by inhibiting peripheral neuropeptides, neurotransmitters, and transcription factors that reduce the inflammatory chemotaxis or anti-inflammatory effect on site).	IL-6 has a crucial role in neuropathic pain development. Glia activation has been implicated in the development of central sensitization, which contributes to the pathogenesis of neuropathic pain. Activated microglia by peripheral nerve injury induces proliferation and activation of microglia in the CNS, and can release pro-inflammatory mediators (e.g., IL 6), which contributes to the development and continuity of neuropathic pain.	√
		Chen, 2021 [14]	TNC	Microglia activation in the CNS may be suppressed by BoNT, through inhibiting the overexpression of microglia-derived pro-inflammatory factors and neuroinflammation.		
CX3CR1 CHEMOKINE RECEPTOR (NEUROPLASTICITY-RELATED)	PIH and TMJ arthritis	Muñoz-Lora, 2020 [16]	TNC	BoNT may have effects on microglia-activated pathways through the reduction of microglia modulators, leading to the reduction of pro-inflammatory cytokines, thus inhibiting microglia-mediated neuroinflammation. BoNT undergoes binding to nerve terminals, thus cleaving SNAREs. By interacting with SNAP25, BoNT blocks the release of neurotransmitters and neuropeptides by vesicle-regulated exocytosis, which may affect neuronal functions as well as alleviate the activation of microglia (potentially via interaction with receptors on microglia).	CX3CR1 is selectively expressed in microglia. The receptor is involved in processes that stimulate the phosphorylation of diverse microglial intra-cellular mechanisms to prompt cell adhesion, migration, and release of pro-inflammatory cytokines, and hence triggering sensory neurons and running pain signals to upper centres. Microglia activation in the hippocampus was reported in depression in PD.	In vitro and model of human-into-mice grafts. Reports of phase 1 trials targeting CX3CR1-blockers in cancer pain and anti- CX3CR1 nanobody in kidney disease.
	Depression—PD	Li, 2023 [18]	Hippocampus			
Dural Protein Extravasation INDICATOR OF NEUROGENIC INFLAMMATION AND TRIGEMINAL ACTIVATION	TN	Filipović, 2012 [28]	Cranial dura Plasma protein complexes	Dural protein extravasation is a relevant aspect of trigeminal pain and can be influenced by the central and peripheral actions of BoNT. BoNT antinociception effect seems to rely on axonal transport through sensory neurons beyond the ganglion, very probably into the CNS.	Dural plasma protein extravasation is often associated with migraine, other headache and pain conditions in the trigeminal region. Plasma protein extravasation in cranial dura is a helpful marker of trigeminal activation, commonly used in preclinical testing of migraine medication.	√ Often employed to explore migraine pathophysiology, but not in BoNT context
	Trigeminal inflammatory pain—TMDs	Lacković, 2016 [20]	Cranial dura tissue, brainstem (TNC)			
Collagen COL- I AND COL- III—INDICATORS OF COLLAGEN DEPOSITION	HS	Xiong, 2023 [30]	Scar tissue (ear)	BoNT improves the appearance of hypertrophic scars (HSs) possibly by inhibiting their growth, throughly interfering with the cell cycle and regulating the TGFβ signalling pathways (e.g., TGF- β1/Smad, ERK, and JNK). BoNT may improve HSs by inhibiting the expression of α-SMA and myosin II, which may block the contraction of fibroblasts.	TGF-β1 high expression is among the key factors precipitating the formation of HS. TGF-β1 may activate several cellular pathways to participate in scar formation. It is a relevant regulator of inflammation, tissue repair and fibrosis. α-SMA is essential in the conversion of fibroblasts into myofibroblasts. Thus, α-SMA high expression suggests enhanced fibroblast fibrosis and overproduction of collagen. The atypical rise in collagen content in the extracellular matrix will determine the characteristics of HS, which is mostly associated with increased expression of COL- I.	Experiments on cultured cells derived from humans with the condition
		Wang, 2020 [25]				
α-SMAS and Myosin II Proteins PRO-FIBROTIC AND COLLAGEN-RELATED PROTEINS—MARKER FOR FIBROBLASTS-MYOFIBROBLASTS TRANSFORMATION		Xiong, 2023 [30]				
		Wang, 2020 [25]				
TGF-β1 PRO-FIBROSIS CYTOKINE (PERIPHERAL INFLAMMATION-RELATED)		Xiong, 2023 [30]				
		Wang, 2020 [25]				
TRPA-1 NOCICEPTORS NONSELECTIVE CATION CHANNEL PROTEINS (REGULATION OF ION CHANNELS)	TN	Wu, 2016 [23]	Brainstem Vc region (caudal subnucleus of the spinal trigeminal nucleus)	Peripherally applied BoNT has potentially peripheral and central antinociceptive effects. BoNT plays a key role in the modulation of expression of certain receptors or ion channels in nociceptor cell membranes, potentially reducing the excitability of pain-transmitting nerve cells.	TRPs have been implicated in the pathogenesis of pain sensation production and hyperalgesia, and are involved in the perception of pain, inflammation, and detecting noxious stimuli. Neuronal excitation, release of inflammatory neuropeptides, and resulting pain hypersensitivity, require TRPA1 channels. These are activated by the liberation of inflammatory agents from neuronal/non-neuronal cells (central and peripheral glial cells—e.g., Schwann cell TRPA1) around injury.	TRPA1 antagonists have been developed and tested in phase -I and -II clinical trials for diseases with prominent pain, but not in BoNT context.
	Chronic dry skin itch	Cao, 2017 [33]	Dorsal root ganglia		TRPA1—key regulator of neuropeptide release and neurogenic inflammation.	

LEGEND: √, biomarkers have been considered for clinical trials; CIS, chronic inflammatory state; BoNT, botulinum toxin; PNS, peripheral nervous system; CNS, central nervous system; HS, hypertrophic scar; TG, trigeminal ganglia; PD, Parkinson Disease; TNC, trigeminal nucleus caudalis; TMD, temporomandibular disorder; TMJ, temporomandibular joint; OA, osteoarthritis; TN, trigeminal neuralgia; PIH, persistent immunogenic hypersensitivity; TNF-α, tumor necrotic factor-α; IL, interleukin; CGRP, calcitonin gene related peptide; SP, substance P; SNAP-25, synaptosomal-associated protein-25; Vc, caudal subnucleus of the spinal trigeminal nucleus; c-Fos, neuron activation marker; CX3CR1, CX3 chemokine receptor 1; COL-I, collagen 1-related proteins; COL-III, collagen 3-related proteins; α-SMA, α-smooth muscle actin; TRPA1, transient receptor potential ankyrin 1; TGF-β1, transforming growth factor beta; vlPAG, ventrolateral periaqueductal gray; CSF, cerebrospinal fluid sampling.

**Table 3:** Biomarkers in preclinical research on botulinum toxin effects in chronic inflammatory state. Summary of outcomes. GRADE consistency assessment.

Biomarker (CIS)		Author Year	Unit Measure	BoNT Key Effect (Outcome)	Summary	Overall GRADE
Biological Sampling						
6 STUDIES						
IL-1β	TNC					
	TN	Chen, 2021 [14]	(mRNA expression)	(*n* = 12) TN vs. (*n* = 12) sham: (B)—ip.l t6 = 8.633, (↑) *p* = 0.0001 (*n* = 12) TN+BoNT (0.18 U) vs. (*n* = 18) TN+vehicle: (T1) ip.l, t6 = 10.39, (↓) *p* < 0.0001	1 RCT (*n* = 48), mean ± SEM (T1) 5 days after BoNT (↓)	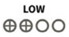
	PHI and TMJ arthritis	Muñoz-Lora, 2020 [16]	(pg/mL tissue)	(*n* = 8) PHI vs. sham: (B)—(↑) *p* < 0.05—NR (*n* = 8) PHI+BoNT (7 U/kg) vs. PHI+vehicle: (T1) (↓) *p* < 0.05—NR (this effect was reversed 7 days after Tx) (*n* = 8) PHI+BoNT (7 U/kg) vs. sham: (T2, T3) (↑) *p* < 0.05—NR	1 RCT (*n* = 40) mean ± SD (T1) 24 h after BoNT (↓) (T2) 7 days after BoNT (NS) (T3) 14 days after BoNT (NS)	
	TMJ tissues					
	TMJ OA	Makawi, 2022 [13]	(pg/mL tissue; mRNA; protein expression)	(*n* = 3) sham vs. (n = 2/group) OA: (T1, T2) 72.2 h(m), 2.6(SD) vs. 321.3 d(m), 4.4(SD) (*n* = 12) OA+BoNT (5 U/kg): (T1) ipl. (↓) *p* < 0.001, 205.3f(m), 10.6(SD); cl. 405.3b(m), 17.2(SD) (*n* = 12) OA+BoNT (5 U/kg): (T2) ipl. (↓) *p* < 0.001, 113.6E(m), 14.7(SD); cl. 464.6C(m), 12.0(SD)	1RCT (*n* = 42), mean (m), SD (T1) 2 weeks after BoNT (↓) (T2) 4 weeks after BoNT (↓)	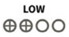
	Peri-articular tissues from TMJ and TG					
	PIH and TMJ arthritis	Muñoz-Lora, 2017 [15]	(pg/mL)	PHI: (B) (vs. sham (↑) *p* < 0.05; vs. PHI+vehicle NS *p* > 0.05)—NR PIH+BoNT (7 U/Kg) vs. PHI/PHI+vehicle: (T1, T2) (↓) *p* < 0.05—NR	1 ? (*n* = ?) ? (T1) 24 h after BoNT (↓) (T2) 14 days after BoNT (↓)	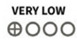
	TG					
	TN	Cho, 2022 [17]	(pg/mL tissue)	(*n* = 6) TN vs. (*n* = 6) sham or (*n* = 6) naive: (B) ip.l/cl. (↑) *p* < 0.05—NR (*n* = 6) TN+BoNT (3 U) vs. (*n* = 6) TN+vehicle: (T1) (↓) (*p* < 0.05)—NR	1 RCT (*n* = 236) mean ± SEM (T1) 2 days after BoNT (↓)	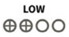
	Hippocampus					
	Depression—PD	Li, 2023 [18]	(mRNA; protein expression)	(*n* = 8)? reserpine vs. sham: (B) (↑)—NR (*n* = 8)? reserpine+BoNT: (T1) (↓) protein (F(3,8) = 14.50, *p* = 0.0013); mRNA (F(3,16) = 7.872, *p* = 0.0019)	1 ? (*n* = ?) mean ± SEM (T1) ? (↓)	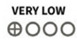
5 STUDIES						
TNF-α	TNC					
	TN	Chen, 2021 [14]	(mRNA; protein expression)	(*n* = 12) TN vs. (*n* = 12) sham: (B)—ip.l t6 = 8.990, (↑) *p* = 0.0001 (*n* = 12) TN+BoNT (0.18 U) vs. (*n* = 18) TN+vehicle: (T1) ip.l, t6 = 9.878, (↓) *p* < 0.0001	1 RCT (*n* = 48), mean ± SEM (T1) 5 days after BoNT (↓)	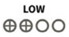
	PHI and TMJ arthritis	Muñoz-Lora, 2020 [16]	(pg/mL tissue)	(*n* = 8) PHI vs. sham: (B)— (↑) *p* < 0.05—NR (*n* = 8) PHI+BoNT (7 U/kg) vs. PHI+vehicle: (T1) NS *p* > 0.05—NR (*n* = 8) PHI+BoNT (7 U/kg) vs. PHI+vehicle: (T2) NS *p* > 0.05—NR (*n* = 8) PHI+BoNT (7 U/kg) vs. PHI+vehicle: (T3) (↓) *p* < 0.05—NR	1 RCT (*n* = 40) mean ± SD (T1) 24 h after BoNT (NS) (T2) 7 days after BoNT (NS) (T3) 14 days after BoNT (↓)	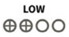
	Peri-articular tissues from TMJ and TG					
	PIH and TMJ arthritis	Muñoz-Lora, 2017 [15]	(pg/mL)	PHI vs. sham: (B) (↑) *p* < 0.05—NR PHI+vehicle vs. sham: (B) NS *p* > 0.05—NR PIH+BoNT (7 U/Kg) vs. PHI/PHI+vehicle: (T1, T2) NS *p* > 0.05—NR	1 ? (*n* = ?) ? (T1) 24 h after BoNT (NS) (T2) 14 days after BoNT (NS)	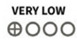
	TG					
	TN	Cho, 2022 [17]	(pg/mL tissue)	(*n* = 6) TN vs. (*n* = 6) sham or (*n* = 6) naive: (B) ip.l/cl. (↑) *p* < 0.05—NR (*n* = 6) TN+BoNT (3 U) vs. (*n* = 6) TN+vehicle: (T1) (↓) (*p* < 0.05)—NR	1 RCT (*n* = 236) mean ± SEM (T1) 2 days after BoNT (↓)	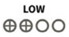
	Hippocampus					
	Depression—PD	Li, 2023 [18]	mRNA; protein expression	(*n* = 8)? reserpine vs. sham: (B) (↑)—NR (*n* = 8)? reserpine+BoNT: (T1) (↓) protein (F(3,8) = 23.78, *p* = 0.0002); mRNA (F(3,16) = 11.79, *p* = 0.0003)	1 ? (*n* = ?) mean ± SEM (T1) ? (↓)	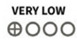
SNAP25	Hippocampus					
	Depression	Li, 2019 [21]	protein expression	naïve vs. SRS vs. (*n* = 6) naïve+BoNT vs. (*n* = 6) SRS+BoNT: expression of SNAP25 in the hippocampus was not altered—NR	1 nRT (*n* = ?) mean ± SEM (T1) 1 h, 1, 3, 7 days (SRS) 16, 18, 22, 29 days (BoNT) (NS)	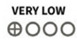
	TNC					
	PIH and TMJ arthritis	Muñoz-Lora, 2022 [22]	(cSNAP-25 staining/positive fibres)	(*n* = 10) PHI vs. sham: (B) (+) ipl.—NR (*n* = 10) PHI+BoNT (OnaBoNT.7 U/kg) vs. PHI+vehicle: (T1) (+) ipl.—NR (*n* = 10) PHI+BoNT (OnaBoNT.7 U/kg) vs. PHI+vehicle: (T1) NS cl.—NR (*n* = 10) PHI+BoNT (AboBoNT.14 U/kg) vs. PHI+vehicle: (T1) NS ipl./cl.—NR	1 RCT (*n* = 40) mean ± SEM (T1) 14 days after OnaBoNT (7 U) (+) ipl.	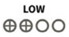
	Cranial dura					
	TMDs trigeminal inflammatory pain	Lacković, 2016 [20]	(presence clSNAP25—fibers containing SNAP-25 co-expressed for CGRP.	(*n* = 4) CFA+BoNT vs. (*n* = ?) CFA+saline: (T1) (+) ipl; (−) cl.l.—NR	1 RCT (*n* = 105) mean ± SEM (T1) 4 days after BoNT (+) ipl.	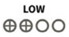
	Brain—hindbrain sections					
	MDD	Ni, 2023 [24]	(positive signal) (% positive signal area)	(*n* = 4 cells/3 mice/subgroup) CRS+BoNT (3 U, 10 U, 30 U): (T1) IFN ipl. (+) (Quantificational analysis among three different dosages: (F(2,9) = 99.68, *p* < 0.0001) (*n* = 4 cells/3 mice/subgroup) CRS+BoNT (30 U): (T1) FN ipl. (+) vs. CRS+BoNT (3 U, 10 U) FN (−) (*n* = 4 cells/3 mice/subgroup) CRS+BoNT (3 U, 10 U, 30 U): (T1) Pr5, Sp5 (−) (*n* = 4 cells/3 mice/subgroup) CRS+BoNT (10 U): (T1) Quantificational analysis of % (cl)SNAP25_197_-positive signal areas among groups of 3 time points: F(2,12) = 4.33, *p* = 0.0384) CRS+BoNT (*n* = 4 brain sections/3 mice) T1 vs. (*n* = 5 brain sections/3 mice) T2: *p* = 0.8069; T1 vs. (*n* = 6 brain sections/3 mice) T3: *p* = 0.4491; T2 vs. T3: *p* = 0.0382	1 RCT (*n* = ?) mean ± SEM (T1) 10 days after BoNT (+) IFN ipl. (T2) 4 weeks after BoNT (+) IFN ipl. (T3) 7 weeks after BoNT (+) IFN ipl.	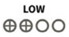
	Brainstem Vc region (caudal subnucleus of the spinal trigeminal nucleus)					
	TN	Wu, 2016 [23]	(by β-actin)	(*n* = 6) TN+saline+Peripheral BoNT vs. (*n* = 6) TN+saline+saline: (T1) (↑) (*p* < 0.05)	1 ? (*n* = ?) mean ± SD (T1) 7 days after BoNT (↑)	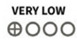
4 STUDIES						
	TNC					
c-Fos	TN	Chen, 2021 [14]	(mRNA; protein expression)	(*n* = 12) TN vs. sham: (B)—ip.l t6 = 4.707, (↑) *p* = 0.0033; cl. t6 = 4.022, (↑) *p* = 0.0069 (*n* = 12) TN+BoNT (0.18 U) vs. (*n* = 18) TN+vehicle: (T1) ip.l, t6 = 5862, (↓) *p* = 0.0011; cl. t6 = 8.647, (↓) *p* = 0.0001	1 RCT (*n* = 48), mean ± SEM (T1) 5 days after BoNT (↓)	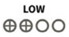
	PHI and TMJ arthritis	Muñoz-Lora, 2022. [22]	levels of c-Fos-positive nuclei	(*n* = 10) PHI vs. sham: (B) (+) ipl. and cl.—NR (*n* = 10) PHI+BoNT (OnaBoNT.7 U/kg) vs. PHI+vehicle: (T1) ipl. (↓) *p* < 0.001; cl. (↓) *p* < 0.01—NR (*n* = 10) PHI+BoNT (AboBoNT.14 U/kg) vs. PHI+vehicle: (T1) ipl. and cl. (↓) *p* < 0.01—NR	1 RCT (*n* = 40) mean ± SEM (T1) 14 days after OnaBoNT (7 U, 14 U) (↓) ipl & cl.	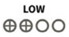
	Brain—hindbrain sections (Ventrolateral periaqueductal gray)					
	MDD	Ni, 2023 [24]	(positive signal/neurons) (% positive signal area)	(*n* = 3) CRS/CRS+saline vs. (*n* = 3) Naïve+Saline: (B) (↑)/*p* = 0.0274—NR (*n* = 3) CRS+BoNT (10 U/Kg) vs. CRS+Saline: (T1) (↓)F (2, 16) = 4.967, *p* = 0.0210	1 RCT (*n* = ?) mean ± SEM (T1) 24 h after day 23 to 27 (↓)	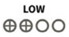
	Medullary dorsal horn					
	TN	Kim, 2015 [26]	(expression—number of neurons—Scale bar, 100 µm)	(*n* = 5) NMDA vs. (*n* = 5) sham: (B) (↑) in the superficial lamina I & II—NR (*n* = 5) ipl. BoNT + NMDA s.c. (3 U/Kg) and i.c. (1 U/Kg) vs. (*n* = 5) vehicle + NMDA: (T1) (↓) *p* < 0.05—NR	1 ? (*n* = ?) means ± SEM (T1) ? (↓)	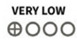
CGRP	Jugular plasma and medulla oblongata—containing caudal trigeminal nucleus					
	Migraine	Shao, 2013 [19]	(pg/mL)	(*n* = 8) NTG vs. (*n* = 8) sham, (a) jugular plasma/(b) oblongata: (B) (↑) 1.8-fold, (a) *p* < 0.01/(b) *p* < 0.05 (*n* = 8) NTG+ BoNT 5 U and 10 U/kg vs. (*n* = 8) NTG + vehicle: (T1) (↓) *p* < 0.01 (a) and (b)	1 ? (*n* = 32) means ± SEM (T1) 24 h after BoNT (↓)	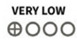
	TNC					
	PIH and TMJ arthritis	Muñoz-Lora, 2022 [22]	(pg/mL) area (µm^2^)	(*n* = 10) PHI vs. sham: (B) (NS) ipl. and cl.—NR (*n* = 10) PHI+BoNT (OnaBoNT.7 U/kg and AboBoNT.14 U/kg) vs. PHI+vehicle: (T1) (NS) F_3,16_ (ip.l.) = 0.3819; F_3,16_ (c.l.) = 1.267	1 RCT (*n* = 40) mean ± SEM (T1) 14 days after BoNT (7 U/14 U) (NS)	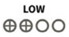
	Peri-articular tissues (TMJ) and TG					
	PIH and TMJ arthritis	Muñoz-Lora, 2017 [15]	(ng/mL)	PHI/PHI+vehicle vs. sham: (B) (↑) *p* < 0.05—NR PIH+BoNT (7 U/Kg) vs. PHI/PHI+vehicle: (T1, T2) (↓) *p* < 0.05—NR	1 ? (*n* = ?) ? (T1) 24 h after BoNT (↓) (T2) 14 days after BoNT (↓)	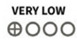
	Dura mater, TNC, TG, CSF					
	Trigeminal inflammatory pain—TMDs	Lacković, 2016 [20]	Concentration (fmol mg^−1^ or fmol mL^−1^)	(*n*/group—6) CFA+saline vs. saline (sham) (a) cranial dura, (b) TNC ipl, (c) TG ipl, (d) CSF: (B) (a) (↑) *p* < 0.01, (b) (↑) *p* < 0.05, (c) and (d) (NS)—NR (*n*/group—6) CFA+BoNT vs. CFA+saline: (T1) (a) (↓) *p* < 0.01, (b) and (c) and (d) (NS)—NR	1 RCT (*n* = 105) mean ± SEM (T1) 4-days after BoNT (↓) cranial dura	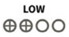
2 STUDIES						
SP	Jugular plasma and medulla oblongata—containing caudal trigeminal nucleus					
	Migraine	Shao, 2013 [19]	(pg/mL)	(*n* = 8) NTG vs. (*n* = 8) sham, (a) jugular plasma/(b) oblongata: (B) (↑) (a) 2.14-fold, *p* < 0.01; (b) 3.14-fold, *p* < 0.05 (*n* = 8) NTG+ BoNT 5 U and 10 U/kg vs. (*n* = 8) NTG + vehicle: (T1) (↓) (a) *p* < 0.05; (b) *p* < 0.01	1 ? (*n* = 32) means ± SEM (T1) 24 h after BoNT (↓)	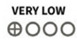
	Peri-articular tissues (TMJ) & TG					
	PIH and TMJ arthritis	Muñoz-Lora, 2017 [15]	(ng/mL)	PHI/PHI+vehicle vs. sham: (B) (↑) *p* < 0.05—NR PIH+BoNT (7 U/Kg) vs. PHI/PHI+vehicle: (T1, T2) (↓) *p* < 0.05—NR	1 ? (*n* = ?) ? (T1) 24 h after BoNT (↓) (T2) 14 days after BoNT (↓)	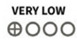
IL-6	TG					
	TN	Cho, 2022 [17]	pg/mLtissue	(*n* = 6) TN vs. (*n* = 6) sham or (*n* = 6) naive: (B) ip.l/cl. (↑) *p* < 0.05—NR (*n* = 6) TN+BoNT (3 U) vs. (*n* = 6) TN+vehicle: (T1) (↓) (*p* < 0.05)—NR	1 RCT (*n* = 236) mean ± SEM (T1) 2 days after BoNT (↓)	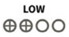
	TNC					
	TN	Chen, 2021 [14]	(mRNA; protein expression)	(*n* = 12) TN vs. (*n* = 12) sham: (B)—ip.l t6 = 5.629, (↑) *p* = 0.0013 (*n* = 12) TN+BoNT (0.18 U) vs. (*n* = 18) TN+vehicle: (T1) ip.l, t6 = 4.657, (↓) *p* = 0.0035	1 RCT (*n* = 48), mean ± SEM (T1) 5 days after BoNT (↓)	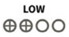
CX3CR1	TNC					
	PIH and TMJ arthritis	Muñoz-Lora, 2020 [16]	(OD, protein level)	(*n* = 8) PHI vs. sham: (B)—(NS) *p* > 0.05—NR (*n* = 8) PHI+BoNT (7 U/kg) vs. PHI+vehicle: (T1) NS *p* > 0.05—NR (*n* = 8) PHI+BoNT (7 U/kg) vs. PHI+vehicle: (T2) NS *p* > 0.05—NR (*n* = 8) PHI+BoNT (7 U/kg) vs. PHI+vehicle: (T3) NS *p* > 0.05—NR	1 RCT (*n* = 40) mean ± SD (T1) 24 h after BoNT (NS) (T2) 7 days after BoNT (NS) (T3) 14 days after BoNT (NS)	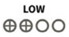
	Hippocampus					
	Depression—PD	Li, 2023 [18]	(mRNA expression levels)	reserpine vs. sham: (B) (↑)—NR reserpine+BoNT vs. reserpine: (T1) (↓) (F(3,20) = 5.718, *p* = 0.0054)	1 ? (*n* = ?) mean ± SEM (T1) ? (↓)	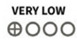
Dural Protein Extravasation	Cranial dura Plasma protein complexes					
	TN	Filipović, 2012 [28]	(ng of Evans blue per mg of dural tissue)	(*n* = 20) TN+saline vs. (*n* = 20) sham: (B) ipl. and cl. (↑) *p* < 0.01—NR (*n* = 20) TN+BoNT vs. (*n* = 20) TN+saline: (T1) (↓) bilateral *p* < 0.01—NR	1 nRT (*n* = 200) mean ± SEM (T1) 3 days after BoNT (↓) ipl. and cl.	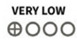
	Cranial dura tissue, brainstem (TNC)					
	Trigeminal inflammatory pain—TMDs	Lacković, 2016 [20]	ng (mg tissue)^−1^	(*n*/group 5–9) CFA+saline vs. saline (sham): (B) ipl. and cl. (↑) *p* < 0.001—NR (*n*/group—6) CFA+BoNT vs. CFA+saline: (T1) (↓) ipl. *p* < 0.001; NS cl.—NR	1 RCT (*n* = 105) mean ± SEM (T1) 4 days after BoNT (↓) ipl.	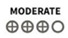
Collagen	Scar tissue (ear)					
	HS	Xiong, 2023 [30]	protein concentration and expression	(*n* = 6) HS+BoNT (2 U) vs. (*n* = 6) HS control: (T1) (↓) COL-I, *p* < 0.001; COL-III, *p* < 0.01—NR	1 RCT (*n* = 24) mean ± SD (T1) 5 weeks after BoNT (↓)	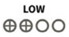
		Wang, 2020 [25]		(*n* = 12) HS vs. healthy skin: (B) (↑) COL-I, *p* < 0.01; COL-III, *p* < 0.05—NR (*n* = 12) HS+BoNT (a) 0.5 U, 1 U, 1.5 U/(b) 2 U: (T1) (↓) COL-I, (a) *p* < 0.01/(b) *p* < 0.05 (vs. HS); NS (vs. sham)—NR (*n* = 12) HS+BoNT (0.5 U, 1 U, 1.5 U, 2 U): (T1) (↓) COL-III, *p* < 0.05 (vs. HS); NS (vs. sham)—NR	1 RCT (*n* = 18) mean ± SD (T1) 28 days after BoNT (↓)	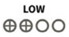
α-SMAS and Myosin II Proteins		Xiong, 2023 [30]	proteins, optical density (OD)	(*n* = 6) HS+BoNT (2 U) vs. (*n* = 6) HS control: (T1) (↓) *p* < 0.001—NR	1 RCT (*n* = 24) mean ± SD (T1) 5 weeks after BoNT (↓)	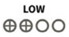
		Wang, 2020 [25]		(*n* = 12) HS vs. healthy skin: (B) (↑) α-SMA, *p* < 0.05; myosin II, *p* < 0.01—NR (*n* = 12) HS+BoNT (a) 0.5 U/(b) 1 U, 1.5 U, 2 U (vs. HS): (T1) α-SMA (a) NS/(b) (↓) *p* < 0.05—NR (*n* = 12) HS+BoNT (0.5 U, 1 U, 1.5 U, 2 U) vs. HS: (T1) (↓) myosin II, *p* < 0.01—NR	1 RCT (*n* = 18) mean ± SD (T1) 28 days after BoNT (↓)	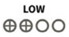
TGF-β1		Xiong, 2023 [30]	Protein concentration and expression	(*n* = 6) HS+BoNT (2 U) vs. (*n* = 6) HS control: (T1) (↓) *p* < 0.001—NR	1 RCT (*n* = 24) mean ± SD (T1) 5 weeks after BoNT (↓)	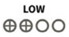
		Wang, 2020 [25]		(*n* = 12) HS vs. healthy skin: (B) (↑) *p* < 0.01—NR (*n* = 12) HS+BoNT (2 U) right ear: (T1) (↓) *p* < 0.01 (vs. HS); NS (vs. sham)—NR (*n* = 12) HS+BoNT (2 U) right ear: (T1) NS vs. HS+BoNT (0.5 U, 1 U, 1.5 U)—NR	1 RCT (*n* = 18) mean ± SD (T1) 28 days after BoNT (↓)	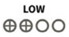
TRPA-1	Brainstem Vc region (caudal subnucleus of the spinal trigeminal nucleus)					
	TN	Wu, 2016 [23]	protein expression	(*n* = 6) TN+saline vs. (*n* = 6) sham+saline+saline: (B) (↑) (*p* < 0.05)—NR (*n* = 6) TN+saline+Peripheral BoNT (3 U/10 U) vs. (*n* = 6) TN+saline+saline: (T1) (↓) (*p* < 0.05)—NR	1 ? (*n* = ?) mean ± SD (T1) 7 days after BoNT (↓)	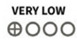
	Dorsal root ganglia					
	TN	Cao, 2017 [33]	protein expression	AEW vs. sham: (T1) (↑) t(10) = 5.414, *p* = 0.0003 AEW vs. sham: (T2) (↑) t(6) = 5.860, *p* = 0.00011 BoNT (0.1 U) vs. saline: (T1) (↓) t(8) = 4.571, *p* = 0.0018 BoNT (0.1 U) vs. saline: (T2) (↓) t(7) = 3.169, *p* = 0.0157	1 ? (*n* = 525) mean ± SD (T1) 3 days BoNT (↓) (T2) 7 days after BoNT (↓)	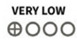

LEGEND: RCT, randomised controlled trial; nRT, non-randomised trial; CIS, chronic inflammatory state; BoNT, botulinum toxin; (↑), statistically significantly higher/increased; (↓), statistically significantly lower/decreased; (−), decrease but not significantly; (B), baseline; SEM, standard error of the mean; NS, no statistically significant difference; NR, not reported; ip.l., ipsilateral; c.l., contralateral; s.c., subcutaneously; i.c., intracisternally; Tx, treatment; SD, standard deviation; HS, hypertrophic scar; TG, trigeminal ganglia; PD, Parkinson Disease; TMD, temporomandibular disorder; TMJ, temporomandibular joint; OA, osteoarthritis; TN, trigeminal neuralgia; PIH, persistent immunogenic hypersensitivity; TNF-α, tumor necrotic factor-α; IL, interleukin; CGRP, calcitonin gene related peptide; SP, substance P; (cl)SNAP-25, (cleaved) synaptosomal-associated protein-25; c-Fos, neuron activation marker; CX3CR1, CX3 chemokine receptor 1; COL-I, collagen 1-related proteins; COL-III, collagen 3-related proteins; α-SMA, α- smooth muscle actin; TRPA-1, transient receptor potential ankyrin 1; Vc, caudal subnucleus of the spinal trigeminal nucleus; AEW, acetone–diethylether–water; TGF-β1, transforming growth factor beta; SRS; spatial restraint stress; CRS; chronic restraint stress; CFA, Complete Freund’s Adjuvant; NTG, nitroglycerin; CSF, cerebrospinal fluid sampling. GRADE certainty ratings—KEY: The true effect is probably markedly different from the estimated effect (VERY LOW); The true effect might be markedly different from the estimated effect (LOW); The true effect is likely close to the estimated effect (MODERATE); Confidence that the true effect is like the estimated effect (HIGH).

## Data Availability

The original contributions presented in this study are included in the article/Supplementary Materials. Further inquiries can be directed to the corresponding author. For example, the full list of citations that were excluded and recorded in the Rayyan platform or the listed reasons for inclusion versus exclusion studies requiring adjudication regarding eligibility are available upon request.

## Supplementary Materials

The following supporting information can be downloaded at: https://www.mdpi.com/article/10.3390/toxins17080377/s1, File S1: Rayyan report; File S2, Table S1: Biomarkers in Preclinical Research on Botulinum Toxin effects on Chronic Inflammatory State. Listed reasons for contacting the corresponding author as per protocol [35,36,37,38,39,40]; File S3, Table S2: Summary of findings [13,14,15,16,17,18,19,20,21,22,23,24,25,26,27,28,29,30,31,32,33,34]; File S4, Table S3: Risk of bias assessment—SYRCLE’s tool [13,14,15,16,17,18,19,20,21,22,23,24,25,26,27,28,29,30,31,32,33,34]; File S5, Table S4: Summary of findings for the biomarkers reported in only one of the included studies [13,14,15,16,17,18,20,21,22,23,25,27,29,31,32,34]; File S6, Table S5: GRADE assessment for biomarkers reported in single experiments [13,14,15,16,17,18,20,21,22,23,25,27,29,31,32,34]; File S7, Table S6: Synopsis of outcomes [13,14,15,16,17,18,19,20,21,22,23,24,25,26,27,28,29,30,31,32,33,34]; File S8: GRADE consistency assessment [13,14,15,16,17,18,19,20,21,22,23,24,25,26,27,28,29,30,31,32,33,34]; File S9 PRISMA 2020 checklist; File S10: Table S7 reviewer 1 (I.N.P.) search strategy; File S10 Table S8 reviewer 2 (S.D.) search strategy.

## Abbreviations

The following abbreviations are used in this manuscript:

BoNT	Botulinum toxin
SNAP25	Synaptosomal-associated protein-25
CGRP	Calcitonin gene related peptide
PRISMA	Preferred Reporting Items for Systematic Reviews and Meta-Analyses
SYRCLE	Systematic Review Centre for Laboratory animal Experimentation
GRADE	Grading of Recommendations Assessment, Development and Evaluation
CI	Confidence interval
TMJ	Temporomandibular joint
MDD	Major depressive disorder
TG	Trigeminal ganglia
TNC	Trigeminal nucleus caudalis
SNpc	Substantia nigra pars compacta
IL-1β	Interleukin 1 beta
TNF-α	Tumor necrotic factor alfa
SP	Substance P
CX3CR1	Microglial purinergic CX3 chemokine receptor 1
TGF-β1	Transforming growth factor beta
α-SMA	α- smooth muscle actin
TRPA	Transient receptor potential ankyrin
IBA-1	Ionized calcium-binding adaptor molecule 1
VGAT	Inhibitory presynaptic marker vesicular GABA transporter
MMP-13	Matrix metallopeptidase 13
BDNF	Brain derived neurotrophic factor
p-ERK	Phosphorylated extracellular signal-regulated kinase
p-CREB	cAMP response element binding protein
IgE	Immunoglobulin E
GFAP	Astroglia marker—glial fibrillary acidic protein
IB4	Isolectin B4-binding
HIF-1α	Hypoxia-inducible factor
PSD95	Postsynaptic density-95
VGlut2	Presynaptic marker vesicular glutamate transporter 2
ATF3	Activating transcription factor 3
TRPM	Transient receptor potential melastatin
TRPV	Protein expression of transient receptor potential vanilloid
PIH	Persistent inflammatory hypernociception
clSNAP25	Cleaved synaptosomal-associated protein-25
SNARE	N-ethylmaleimide-sensitive-factor attachment receptor
IoNC	Infraorbital nerve constriction
CFA	Complete Freund’s Adjuvant
AEW	Acetone–diethylether–water

## References

[1] Hajj R., Haddad C. (2021). The Anti-Nociceptive/Anti-Inflammatory Actions of Botulinum Toxin A for the Treatment of Chronic Pain: A Literature Review. Curr. Res. Dent..

[2] Geoghegan L., Rodrigues R., Harrison C.J., Rodrigues J.N. (2022). The Use of Botulinum Toxin in the Management of Hidradenitis Suppurativa: A Systematic Review. Plast. Reconstr. Surg. Glob. Open.

[3] Drugs@FDA: FDA-Approved Drugs. https://www.accessdata.fda.gov/drugsatfda_docs/label/2011/103000s5236lbl.pdf.

[4] Li Y., Liu T., Luo W. (2021). Botulinum Neurotoxin Therapy for Depression: Therapeutic Mechanisms and Future Perspective. Front. Psychiatry.

[5] Finnerup N.B., Attal N., Haroutounian S., McNicol E., Baron R., Dworkin R.H., Gilron I., Haanpää M., Hansson P., Jensen T.S. (2015). Pharmacotherapy for Neuropathic Pain in Adults: A Systematic Review and Meta-Analysis. Lancet Neurol..

[6] Matak I., Bölcskei K., Bach-Rojecky L., Helyes Z. (2019). Mechanisms of Botulinum Toxin Type A Action on Pain. Toxins.

[7] Novo Pereira I., Durão S., Hassan H., Braga A.C., Mariz Almeida A., Manso A.C., Faria-Almeida R., De la Torre Canales G. (2025). Botulinum Toxin Effects on Biochemical Biomarkers Related to Inflammation-Associated Head and Neck Chronic Conditions: A Systematic Review of Clinical Research. J. Neural. Transm..

[8] FDA-NIH Biomarker Working Group (2016). BEST (Biomarkers, EndpointS, and Other Tools) Resource.

[9] Smith S., Dworkin R., Turk D., Baron R., Polydefkis M., Tracey I., Borsook D., Edwards R., Harris R., Wager T. (2017). The Potential Role of Sensory Testing, Skin Biopsy, and Functional Brain Imaging as Biomarkers in Chronic Pain Clinical Trials: IMMPACT Considerations. J. Pain.

[10] Bodaghi A., Fattahi N., Ramazani A. (2023). Biomarkers: Promising and Valuable Tools towards Diagnosis, Prognosis and Treatment of Covid-19 and Other Diseases. Heliyon.

[11] Bahadoran Z., Mirmiran P., Kashfi K., Ghasemi A. (2020). Importance of Systematic Reviews and Meta-Analyses of Animal Studies: Challenges for Animal-to-Human Translation. J. Am. Assoc. Lab. Anim. Sci..

[12] Sandercock P., Roberts I. (2002). Systematic Reviews of Animal Experiments. Lancet.

[13] Makawi D., Korany N.S., Taha N.S., Abbass M.M.S. (2022). Regenerative Potential of Botox Combined and Uncombined with Platelet-Rich Plasma in Treating Induced Osteoarthritis of Temporomandibular Joint in Albino Rats. Egypt. J. Histol..

[14] Chen W.-J., Niu J.-Q., Chen Y.-T., Deng W.-J., Xu Y.-Y., Liu J., Luo W.-F., Liu T. (2021). Unilateral Facial Injection of Botulinum Neurotoxin A Attenuates Bilateral Trigeminal Neuropathic Pain and Anxiety-like Behaviors through Inhibition of TLR2-Mediated Neuroinflammation in Mice. J. Headache Pain.

[15] Lora V.R.M.M., Clemente-Napimoga J.T., Abdalla H.B., Macedo C.G., de la Torre Canales G., Barbosa C.M.R. (2017). Botulinum Toxin Type A Reduces Inflammatory Hypernociception Induced by Arthritis in the Temporomadibular Joint of Rats. Toxicon.

[16] Manuel Muñoz-Lora V.R., Abdalla H.B., Del Bel Cury A.A., Clemente-Napimoga J.T. (2020). Modulatory Effect of Botulinum Toxin Type A on the Microglial P2X7/CatS/FKN Activated-Pathway in Antigen-Induced Arthritis of the Temporomandibular Joint of Rats. Toxicon.

[17] Cho J.H., Son J.Y., Ju J.S., Kim Y.M., Ahn D.K. (2022). Cellular Mechanisms Mediating the Antinociceptive Effect of Botulinum Toxin A in a Rodent Model of Trigeminal Irritation by a Foreign Body. J. Pain.

[18] Li Y., Yin Q., Li Q., Huo A.-R., Shen T.-T., Cao J.-Q., Liu C.-F., Liu T., Luo W.-F., Cong Q.-F. (2023). Botulinum Neurotoxin A Ameliorates Depressive-like Behavior in a Reserpine-Induced Parkinson’s Disease Mouse Model via Suppressing Hippocampal Microglial Engulfment and Neuroinflammation. Acta Pharmacol. Sin..

[19] Shao Y.-F., Zhang Y., Zhao P., Yan W.-J., Kong X.-P., Fan L.-L., Hou Y.-P. (2013). Botulinum Toxin Type a Therapy in Migraine: Preclinical and Clinical Trials. Iran. Red Crescent Med. J..

[20] Lacković Z., Filipović B., Matak I., Helyes Z. (2016). Activity of Botulinum Toxin Type A in Cranial Dura: Implications for Treatment of Migraine and Other Headaches. Br. J. Pharmacol..

[21] Li Y., Liu J., Liu X., Su C.-J., Zhang Q.-L., Wang Z.-H., Cao L.-F., Guo X.-Y., Huang Y., Luo W. (2019). Antidepressant-Like Action of Single Facial Injection of Botulinum Neurotoxin A Is Associated with Augmented 5-HT Levels and BDNF/ERK/CREB Pathways in Mouse Brain. Neurosci. Bull..

[22] Muñoz-Lora V.R.M., Dugonjić Okroša A., Matak I., Del Bel Cury A.A., Kalinichev M., Lacković Z. (2022). Antinociceptive Actions of Botulinum Toxin A1 on Immunogenic Hypersensitivity in Temporomandibular Joint of Rats. Toxins.

[23] Wu C., Xie N., Lian Y., Xu H., Chen C., Zheng Y., Chen Y., Zhang H. (2016). Central Antinociceptive Activity of Peripherally Applied Botulinum Toxin Type A in Lab Rat Model of Trigeminal Neuralgia. Springerplus.

[24] Ni L., Chen H., Xu X., Sun D., Cai H., Wang L., Tang Q., Hao Y., Cao S., Hu X. (2023). Neurocircuitry Underlying the Antidepressant Effect of Retrograde Facial Botulinum Toxin in Mice. Cell Biosci..

[25] Wang J., Liu D., Li X., Li X.-J. (2020). BTXA Could Induce Fibroblast Apoptosis and Inhibit the Expression of α-SMA and Myosin II in Scar Tissue of Rabbit Ears. Biotechnol. Bioproc E.

[26] Kim H.-J., Lee G.-W., Kim M.-J., Yang K.-Y., Kim S.-T., Bae Y.-C., Ahn D.-K. (2015). Antinociceptive Effects of Transcytosed Botulinum Neurotoxin Type A on Trigeminal Nociception in Rats. Korean J. Physiol. Pharmacol..

[27] Han S.B., Kim H., Cho S.H., Chung J.H., Kim H.S. (2017). Protective Effect of Botulinum Toxin Type A Against Atopic Dermatitis-Like Skin Lesions in NC/Nga Mice. Dermatol. Surg..

[28] Filipović B., Matak I., Bach-Rojecky L., Lacković Z. (2012). Central Action of Peripherally Applied Botulinum Toxin Type A on Pain and Dural Protein Extravasation in Rat Model of Trigeminal Neuropathy. PLoS ONE.

[29] Kitamura Y., Matsuka Y., Spigelman I., Ishihara Y., Yamamoto Y., Sonoyama W., Kamioka H., Yamashiro T., Kuboki T., Oguma K. (2009). Botulinum Toxin Type a (150 kDa) Decreases Exaggerated Neurotransmitter Release from Trigeminal Ganglion Neurons and Relieves Neuropathy Behaviors Induced by Infraorbital Nerve Constriction. Neuroscience.

[30] Xiong J., Li X., Xu G., Wang Y., Wen H. (2023). Effectiveness of Fractional Carbon Dioxide Laser Combined with Botulinum Toxin Type A in a Rabbit Ear Model with the Underlying Mechanism. J. Cosmet. Dermatol..

[31] Yang K.Y., Kim M.J., Ju J.S., Park S.K., Lee C.G., Kim S.T., Bae Y.C., Ahn D.K. (2016). Antinociceptive Effects of Botulinum Toxin Type A on Trigeminal Neuropathic Pain. J. Dent. Res..

[32] Zhang Y., Su Q., Lian Y., Chen Y. (2019). Botulinum Toxin Type A Reduces the Expression of Transient Receptor Potential Melastatin 3 and Transient Receptor Potential Vanilloid Type 4 in the Trigeminal Subnucleus Caudalis of a Rat Model of Trigeminal Neuralgia. Neuroreport.

[33] Cao L.-F., Si M., Huang Y., Chen L.-H., Peng X.-Y., Qin Y.-Q., Liu T.-T., Zhou Y., Liu T., Luo W.-F. (2017). Long-Term Anti-Itch Effect of Botulinum Neurotoxin A Is Associated with Downregulation of TRPV1 and TRPA1 in the Dorsal Root Ganglia in Mice. Neuroreport.

[34] Yesudhas A., Radhakrishnan R.K., Sukesh A., Ravichandran S., Manickam N., Kandasamy M. (2021). BOTOX^®^ Counteracts the Innate Anxiety-Related Behaviours in Correlation with Increased Activities of Key Antioxidant Enzymes in the Hippocampus of Ageing Experimental Mice. Biochem. Biophys. Res. Commun..

[35] Baral H., Sekiguchi A., Uchiyama A., Nisaa Amalia S., Yamazaki S., Inoue Y., Yokoyama Y., Ogino S., Torii R., Hosoi M. (2021). Inhibition of Skin Fibrosis in Systemic Sclerosis by Botulinum Toxin B via the Suppression of Oxidative Stress. J. Dermatol..

[36] Choi J.E., Werbel T., Wang Z., Wu C.C., Yaksh T.L., Di Nardo A. (2019). Botulinum Toxin Blocks Mast Cells and Prevents Rosacea like Inflammation. J. Dermatol. Sci..

[37] Ward N.L., Kavlick K.D., Diaconu D., Dawes S.M., Michaels K.A., Gilbert E. (2012). Botulinum Neurotoxin A Decreases Infiltrating Cutaneous Lymphocytes and Improves Acanthosis in the KC-Tie2 Mouse Model. J. Investig. Dermatol..

[38] Li X., Ye Y., Wang L., Zhou W., Chu X., Li T. (2022). Botulinum Toxin Type a Combined with Transcranial Direct Current Stimulation Reverses the Chronic Pain Induced by Osteoarthritis in Rats. Toxicon.

[39] Amalia S.N., Uchiyama A., Baral H., Inoue Y., Yamazaki S., Fujiwara C., Sekiguchi A., Yokoyama Y., Ogino S., Torii R. (2021). Suppression of Neuropeptide by Botulinum Toxin Improves Imiquimod-Induced Psoriasis-like Dermatitis via the Regulation of Neuroimmune System. J. Dermatol. Sci..

[40] Liu D.-Q., Li X.-J., Weng X.-J. (2017). Effect of BTXA on Inhibiting Hypertrophic Scar Formation in a Rabbit Ear Model. Aesthetic Plast. Surg..

[41] Page M.J., McKenzie J.E., Bossuyt P.M., Boutron I., Hoffmann T.C., Mulrow C.D., Shamseer L., Tetzlaff J.M., Akl E.A., Brennan S.E. (2021). The PRISMA 2020 Statement: An Updated Guideline for Reporting Systematic Reviews. BMJ.

[42] Cutrona C., Marchet F., Costanzo M., De Bartolo M.I., Leodori G., Ferrazzano G., Conte A., Fabbrini G., Berardelli A., Belvisi D. (2023). Exploring the Central Mechanisms of Botulinum Toxin in Parkinson’s Disease: A Systematic Review from Animal Models to Human Evidence. Toxins.

[43] Ham H.J., Yeo I.J., Jeon S.H., Lim J.H., Yoo S.S., Son D.J., Jang S.-S., Lee H., Shin S.-J., Han S.B. (2022). Botulinum Toxin A Ameliorates Neuroinflammation in the MPTP and 6-OHDA-Induced Parkinson’s Disease Models. Biomol. Ther..

[44] Gfrerer L., Xu W., Austen W., Ashina S., Melo-Carrillo A., Longhi M.S., Adams A.M., Houle T., Brin M.F., Burstein R. (2022). OnabotulinumtoxinA Alters Inflammatory Gene Expression and Immune Cells in Chronic Headache Patients. Brain.

[45] Hooijmans C.R., Rovers M.M., de Vries R.B.M., Leenaars M., Ritskes-Hoitinga M., Langendam M.W. (2014). SYRCLE’s Risk of Bias Tool for Animal Studies. BMC Med. Res. Methodol..

[46] GRADE Book. https://book.gradepro.org/.

[47] Wei D., Tang K., Wang Q., Estill J., Yao L., Wang X., Chen Y., Yang K. (2016). The Use of GRADE Approach in Systematic Reviews of Animal Studies. J. Evid. Based Med..

[48] Pereira I.N., Durão S., De la Torre Canales G., Braga A.C., Almeida A.M., Hassan H., Manso A.C., Almeida R.F. Biomarkers in Preclinical Research on Botulinum Toxin Effects on Inflammation-Associated Chronic Conditions. PROSPERO 2023 CRD42023432411. https://www.crd.york.ac.uk/prospero/display_record.php?ID=CRD42023432411.

