# Supplementary material for: Botulinum Toxin Effects on Biochemical Biomarkers Related to Inflammation-Associated Head and Neck Chronic Conditions: A Systematic Review of Preclinical Research
Source: Toxins (Basel). 2025 Jul 29;17(8):377. doi: 10.3390/toxins17080377 (PMC12390450; doi:10.3390/toxins17080377)
Supplement: Supplementary file 1 [file toxins-17-00377-s001.zip › SR2. file S1. Rayyan report.pdf]

File S1. Rayyan REPORT

Possible Duplicates: Unresolved **0**; Deleted **672**; Not duplicates **2**; Resolved **143**

Inclusion decisions: Undecided **1373/0**; Maybe **1373/61**; Included **1373/19**; Excluded **1373/1293**

Search methods:

Uploaded References [pubmed-neurotoxin-set(6).txt]: 114

Uploaded References [pubmed-neurotoxin-set(7).txt]: 117

Uploaded References [savedrecs(1).bib]: 764

Uploaded References [scopus.ris]: 578

Uploaded References [pubmed-neurotoxin-set(7).txt]: 118

Uploaded References [savedrecs(1).bib]: 84

Uploaded References [scopus.ris]: 266

Keywords for include: randomized: 113, placebo: 112; compared with: 108, trial: 84, placebo controlled: 74, double blind: 66, randomly: 39, controlled trial: 27, randomly assigned: 20, controlled study: 19, randomised: 18, assigned to: 15, randomized controlled trial: 10, single blind: 8, parallel group: 7, crossover: 7, double blinded: 6, cross over: 5, randomised controlled trial: 4, control groups: 3, RCT: 2, controlled design: 1, parallel groups: 1, randomly allocated: 0, double marked: 0, doubleblinded: 0, single masked: 0, doubleblind: 0, CCT: 0

Keywords for exclude: rats: 175, rat: 159, cells: 142, mice: 138, trials: 88, mouse: 78, animal: 60, animals: 52, in vitro: 36, case report: 34, this review: 30, clinical trial: 29, cohort: 27, prevalence: 26, murine: 26, observational: 19, retrospectively: 18, retrospective study: 17, survey: 17, reviews: 15, toxin substrate 1: 14, rodent: 14, systematic review: 13, rabbit: 13, rabbits: 10, case reports: 9, longitudinal: 9, regression analysis: 8, equine: 8, randomized controlled trials: 7; literature review: 7, meta-analysis: 7, case control: 5, cross-sectional: 4, rodents: 4, canine: 4, retrospective cohort: 2, regression analyses: 2, healthy controls: 2, non-randomized: 2, age-matched: 2, cadavers: 2, porcine: 2, fish: 2, soil: 2, randomised controlled trials: 1, sensitivity and specificity: 1, nonrandomized: 1, single arm: 1, transgenic: 1, cadaveric: 1, healthy control: 0, non-randomised: 0, nonrandomised: 0, broilers: 0, purebred: 0, beagles: 0, broiler: 0, cadaver: 0, piglets: 0, beagle: 0

Labels: not evaluating BoNT: 659, clinical study: 466, not assessing biomarkers: 392, review article: 243, target not head and neck: 139, target not chronic disease model: 133, head and neck?: 69, in vitro study: 33, chronic condition?: 29, effect primarily on muscle contraction: 29, combined therapy?: 25, special issue: 15, modified BoNT: 14, evaluating botulism: 12, effect primarily on glands contraction: 8, bio functional measures: 8, healthy models: 8, pretreatment with BoNT?: 5, contact corresponding author: 4, biomarker?: 4, novel engineered botulinum molecule: 3, animal model?: 3, full text accessible?: 2, prevention (wound healing) rather than treatment of HS: 2; linked to inflammation: 2

Exclusion reasons: wrong study design: 732, wrong drug: 655, wrong outcome: 612, wrong population: 4

Main language: eng: 109, jpn: 1

Publication types: Journal Article: 1370, Research Support, Non-U.S. Gov't: 49, Review: 18, Research Support, N.I.H., Extramural: 12, Comparative Study: 7, Research Support, U.S. Gov't, P.H.S.: 5, Research Support, U.S. Gov't, Non-P.H.S.: 3, Evaluation Study: 2, INPR: 1, Randomized Controlled Trial: 1, Editorial: 1, Systematic Review: 1, Introductory Journal Article: 1, Letter: 1

Journal: HEADACHE: 53, TOXINS: 30, JOURNAL OF HEADACHE AND PAIN: 18, REVISTA DE NEUROLOGIA: 15, CEPHALALGIA: 12, Pain: 9, EUROPEAN JOURNAL OF NEUROLOGY: 9, LARYNGOSCOPE: 8, Neuroscience: 8, Scientific Reports: 8, PLASTIC AND RECONSTRUCTIVE SURGERY: 8, PAIN: 8, NEUROLOGY: 7, JOURNAL OF NEUROLOGY: 7, JOURNAL OF NEURAL TRANSMISSION: 7, NEUROLOGICAL SCIENCES: 7, MEDICINE: 7, International Journal of Dentistry and Oral Science: 7, European Chemical Bulletin: 7, Toxins: 7, TOXICON: 7, PAIN MEDICINE: 7, NERVENHEILKUNDE: 6, PLOS ONE: 6, Journal of Neuroscience: 6, CLINICAL NEUROLOGY AND NEUROSURGERY: 6, SCHMERZ: 6, AKTUELLE NEUROLOGIE: 6, PLoS ONE: 6, Toxicon : official journal of the International Society on Toxinology: 6

Authors: Zhang, Y.: 15, o: 11, Dodick, David W.: 11, Silberstein, Stephen D.: 11, Wang, Y.: 10, Brin, Mitchell F.: 10, Lacković, Z.: 9, Wang, L.: 9, Wang, Z.: 9, S: 8, Lacković Z: 7, Fern: 7, Bach-Rojecky, L.: 7, Wang, X.: 7, Liu, Y.: 7, Lipton, Richard B.: 7, Zhang, J.: 7, Mathew, Ninan T.: 6, Tassorelli, Cristina: 6, Li, X.: 6, Aurora, Sheena K.: 6, Zhang, Z.: 6, Zhang, X.: 6, Matak, I.: 6, Li, J.: 6, Janis, Jeffrey E.: 6, DeGryse, Ronald E.: 5, Blumenfeld, Andrew M.: 5, Ahmed, Fayyaz: 5, Mahowald, Maren L.: 5, Matak I: 5, Martelletti, Paolo: 5, Huang, L.: 5, Wang, J.: 5, Diener, Hans-Christoph: 5, Li, L.: 5, Yang, J.: 5, Xu, H.: 5, Sun, Y.: 5, Luvisetto, Siro: 4, Blumenfeld, Andrew: 4, Hu, X.: 4, Gaul, Charly: 4, G: 4, Ogino, S.: 4, Jabbari, B: 4, Laskawi, Rainer: 4, Schwartz, M: 4, Uchiyama, A.: 4, ra: 4, Aoki, K. Roger: 4, Birklein, F.: 4, Pavone, Flaminia: 4, Silberstein, SD: 4, Marinelli, S.: 4, Yokoyama, Y.: 4, Gfrerer, Lisa: 4, Ma, L.: 4, Irimia, Pablo: 4, Turkel, Catherine C.: 4, Luvisetto, S.: 4, Kalinichev, M.: 4, Zhang, S.: 4, Yang, Y.: 4, er: 4, Liu, H.: 4, Motegi, S.-I.: 4, Pavone, F.: 4, Liu, Z.: 4, Chen, J.: 4, Pascual, Julio: 4, Evers, S.: 4, Chen, Yuan: 4, Lian, Yajun: 4, Straube, Andreas: 4, Durham, P.L.: 4, Guntinas-Lichius, Orl: 4, ro: 4, Guerrero, Angel L.: 4, Blitzer, Andrew: 4, Rizzatti-Barbosa, Celia Marisa: 3, Akai, R.: 3, Kaye, Alan D.: 3, Blumenfeld, A: 3, Kim, Seong-Taek: 3, Ishikawa, O.: 3, Jankovic, Joseph: 3, Yu, J.: 3, Torii, R.: 3, Zhang, C.: 3, re: 3, Yaksh, T.L.: 3, Martinez-Pias, Enrique: 3, Li, T.: 3, Burstein, Rami: 3, Bach-Rojecky L: 3, Wang, G.: 3, Chen, C.: 3, Sekiguchi, A.: 3, Delussi, Marianna: 3

Year: 2024: 6, 2023: 116, 2022: 118, 2021: 107, 2020: 96, 2019: 96, 2018: 72, 2017: 78, 2016: 62, 2015: 69, 2014: 64, 2013: 56, 2012: 53, 2011: 45, 2010: 38, 2009: 45, 2008: 38, 2007: 30, 2006: 34, 2005: 19, 2004: 28, 2003: 13, 2002: 15, 2001: 13, 2000: 13, 1999: 13, 1998: 8, 1997: 9, 1996: 6, 1995: 1, 1994: 2, 1993: 2, 1992: 1; 1991: 1, 1985: 2, 1984: 1, 1983: 1, 1977: 1, 1974: 1
