# Supplementary material for: Botulinum Toxin Effects on Biochemical Biomarkers Related to Inflammation-Associated Head and Neck Chronic Conditions: A Systematic Review of Preclinical Research
Source: Toxins (Basel). 2025 Jul 29;17(8):377. doi: 10.3390/toxins17080377 (PMC12390450; doi:10.3390/toxins17080377)
Supplement: Supplementary file 1 [file toxins-17-00377-s001.zip › SR2. file S10. Search Table S7 and S8.pdf]

File S10. Table S7 – Reviewer 1 (IP) search strategy

Terms used in the research.

Database	Search Format
PUBMED	(((neurotoxin) OR (neuromodulator)) AND ((inflammation) OR (inflammatory state) OR (chronic orofacial pain) OR (orofacial neuropathic pain) OR (chronic migraine) OR (chronic arthritis) OR (herpetic neuralgia) OR (trigeminal neuralgia) OR (temporomandibular joint pain) OR (head and neck myofascial pain) OR (facial hypertrophic scars) OR (facial keloids) OR (periodontitis) OR (mood disorders) OR (anxiety) OR (chronic stress) OR (depression) OR (rosacea) OR (psoriasis) OR (dermatitis) OR (cephalalgia) OR (alopecia)) AND ((neuroinflammation) OR (neurogenic inflammation) OR (neuropeptides) OR (substance P) OR (calcitonin gene relative peptide) OR (glutamate) OR (anti-inflammatories (pain mediators) OR (inflammation mediators) OR (cytokines) OR (chemokines) OR (interleukin – 6) OR (interleukin – 10) OR (interleukin – 1 beta) OR (tumor necrosis factor alfa) OR (tumor necrosis factor weak inducer of apoptosis) OR (C-reactive protein) OR (monocyte chemoattractant protein-1) OR (brain-derived neurotrophic factor) OR (nerve growth factor) OR (astroglia markers) OR (microglia markers) OR (glial fibrillary acidic protein) OR (5-hydroxytryptamine) OR (ionized calcium-binding adaptor molecule 1) OR (antioxidant stress) OR (reactive oxidative stress) OR (reactive oxygen species) OR (secondary messengers) OR (markers) OR (biomarkers))) AND (botulinum toxin)<br><br>*A)* ***Filters****: Other animals.* ***117 selected items*** Sent On: Mon Jan 01 19:35:36 2024<br><br>*B)* ***Filters****: Full text, Other animals.* ***114 selected items*** Sent On: Mon Jan 01 19:37:39 2024
SCOPUS	**botulinum** AND **toxin** AND **chronic** AND **inflammation** AND (EXCLUDE (SUBJAREA , "AGRI") OR EXCLUDE (SUBJAREA , "ENVI") OR EXCLUDE (SUBJAREA , "COMP") OR EXCLUDE (SUBJAREA , "PHYS") OR EXCLUDE (SUBJAREA , "SOCI") OR EXCLUDE (SUBJAREA , "EART") OR EXCLUDE (SUBJAREA , "MATH") OR EXCLUDE (SUBJAREA , "ARTS") OR EXCLUDE (SUBJAREA , "BUSI") OR EXCLUDE (SUBJAREA , "ECON") OR EXCLUDE (SUBJAREA , "DECI")) AND (EXCLUDE (DOCTYPE , "re") OR EXCLUDE (DOCTYPE , "ch") OR EXCLUDE (DOCTYPE , "bk") OR EXCLUDE (DOCTYPE , "cp") OR EXCLUDE (DOCTYPE , "sh") OR EXCLUDE (DOCTYPE , "ed") OR EXCLUDE (DOCTYPE , "no") OR EXCLUDE (DOCTYPE , "le") OR EXCLUDE (DOCTYPE , "tb")) AND (EXCLUDE (EXACTSRCTITLE , "Neurourology And Urodynamics") OR EXCLUDE (EXACTSRCTITLE , "Journal Of Urology") OR EXCLUDE (EXACTSRCTITLE , "Urology") OR EXCLUDE (EXACTSRCTITLE , "European Urology") OR EXCLUDE (

Database	Search Format
	EXACTSRCTITLE , "Physiological Reviews") OR EXCLUDE (EXACTSRCTITLE , "International Urology And Nephrology") OR EXCLUDE (EXACTSRCTITLE , "Luts Lower Urinary Tract Symptoms") OR EXCLUDE (EXACTSRCTITLE , "International Urogynecology Journal") OR EXCLUDE (EXACTSRCTITLE , "Journal Of Foot And Ankle Surgery") OR EXCLUDE (EXACTSRCTITLE , "Korean Journal Of Urology") OR EXCLUDE (EXACTSRCTITLE , "World Journal Of Urology") OR EXCLUDE (EXACTSRCTITLE , "Current Urology Reports") OR EXCLUDE (EXACTSRCTITLE , "Heart Rhythm") OR EXCLUDE (EXACTSRCTITLE , "Gastrointestinal Endoscopy") OR EXCLUDE (EXACTSRCTITLE , "Japanese Journal Of Clinical Urology") OR EXCLUDE (EXACTSRCTITLE , "Urological Science") OR EXCLUDE (EXACTSRCTITLE , "Aktuelle Urologie") OR EXCLUDE (EXACTSRCTITLE , "Journal Of Pediatric Urology")) AND (EXCLUDE (EXACTKEYWORD , "Human") OR EXCLUDE (EXACTKEYWORD , "Humans") OR EXCLUDE (EXACTKEYWORD , "Unclassified Drug") OR EXCLUDE (EXACTKEYWORD , "Clinical Article") OR EXCLUDE (EXACTKEYWORD , "Human Cell") OR EXCLUDE (EXACTKEYWORD , "In Vitro Study") OR EXCLUDE (EXACTKEYWORD , "Urinary Bladder") OR EXCLUDE (EXACTKEYWORD , "Bladder") OR EXCLUDE (EXACTKEYWORD , "Clinical Trial") OR EXCLUDE (EXACTKEYWORD , "Lidocaine") OR EXCLUDE (EXACTKEYWORD , "Overactive Bladder") OR EXCLUDE (EXACTKEYWORD , "Interstitial Cystitis") OR EXCLUDE (EXACTKEYWORD , "Clinical Feature") OR EXCLUDE (EXACTKEYWORD , "Spinal Cord Injury") OR EXCLUDE (EXACTKEYWORD , "Urothelium") OR EXCLUDE (EXACTKEYWORD , "Cystitis") Number of results : **578** On: Mon Jan 01 21:39:23 2024
WEB OF SCIENCE	(ALL=**(botulinum toxin))** AND ALL=**(chronic)** and Review Article or Meeting Abstract or Proceeding Paper or Letter or Editorial Material or Early Access or Note or Correction or Book Chapters or Data Paper or News Item or Retracted Publication (**Exclude – Document Types)** and 1.142 Urology or 1.95 Gastrointestinal & Esophageal Diseases or 1.129 Back Pain or 1.128 Fertility, Endometriosis & Hysterectomy or 1.255 Musculoskeletal Disorders or 1.233 Pelvic & Renal Disorders or 1.199 Lung Cancer **(Exclude – Citation Topics Meso)** and Pediatrics or Urology Nephrology or Energy Fuels or Computer Science or Legal Medicine or Linguistics or Medical Informatics or Substance Abuse or Telecommunications or Obstetrics Gynecology or Respiratory System **(Exclude – Research Areas)** and Cardiac Cardiovascular Systems or Genetics Heredity or Gastroenterology Hepatology or Environmental Sciences or Materials Science Biomaterials **(Exclude – Web of Science Categories)** and 1.82 Gait & Posture or 10.240 Music or 1.94 Cardiac Arrhythmia or 2.211 Mass Spectrometry or 2.176 Drug Delivery Chemistry or 3.45 Soil Science or 3.60 Herbicides, Pesticides & Ground Poisoning or 1.194 Tuberculosis & Leprosy or 1.168 Vascular,

Database	Search Format
	Cardiac & Thoracic Surgery or 1.137 Sleep Science & Circadian Systems or 1.134 Trauma & Emergency Surgery or 1.105 Strokes or 3.2 Marine Biology or 1.102 Stem Cell Research or 4.289 Biophotonics & Electromagnetic Field Safety or 1.195 Neuroendocrine & Intestinal Disorders or 1.315 Laser Surgery, Therapy & Protection or 1.273 Health Literacy & Telemedicine **(Exclude – Citation Topics Meso)** and 1.34.982 Achilles Tendon or 1.247.2375 Hyperhidrosis or 1.253.1927 Compartment Syndrome or 1.34.1657 Elbow or 1.43.1203 Acupuncture or 1.43.1905 Complex Regional Pain Syndrome or 1.5.894 Nicotine or 3.16.314 Essential Oil or 1.81.2290 Vasectomy or 1.173.1322 Breast Reconstruction or 1.253.1100 Carpal Tunnel Syndrome or 1.34.485 Shoulder or 1.216.1172 Inguinal Hernia (Exclude – Citation Topics Micro) and MOVEMENT DISORDERS **(Exclude – Publication Titles) 764 *selected items (764 articles; 392 review articles)*** Sent On: Mon Jan 01 20:45:35 2024

Website/Register	Search Format
preclinicaltrials.eu<br>animalstudyregistry.org	Intervention/treatment = (“**Botulinum toxin**”)<br>Number of results : **0** On: Mon Jan 01 22:37:24 2024
PROSPERO	**4** records found for **botulinum toxin;** Source of the review: **Animal:DB** On: Mon Jan 01 22:02:19 2024

Table S8 – Reviewer 2 (SD) search strategy

Terms used in the research.

Database	Search Format
PUBMED	(((neurotoxin) OR (neuromodulator)) AND ((inflammation) OR (inflammatory state) OR (chronic orofacial pain) OR (orofacial neuropathic pain) OR (chronic migraine) OR (chronic arthritis) OR (herpetic neuralgia) OR (trigeminal neuralgia) OR (temporomandibular joint pain) OR (head and neck myofascial pain) OR (facial hypertrophic scars) OR (facial keloids) OR (periodontitis) OR (mood disorders) OR (anxiety) OR (chronic stress) OR (depression) OR (rosacea) OR (psoriasis) OR (dermatitis) OR (cephalalgia) OR (alopecia)) AND ((neuroinflammation) OR (neurogenic inflammation) OR (neuropeptides) OR (substance P) OR (calcitonin gene relative peptide) OR (glutamate) OR (anti-inflammatories (pain mediators) OR (inflammation mediators) OR (cytokines) OR (chemokines) OR (interleukin – 6) OR (interleukin – 10) OR (interleukin – 1 beta) OR (tumor necrosis factor alfa) OR (tumor necrosis factor weak inducer of apoptosis) OR (C-reactive protein) OR (monocyte chemoattractant

Database	Search Format
	protein-1) OR (brain-derived neurotrophic factor) OR (nerve growth factor) OR (astroglia markers) OR (microglia markers) OR (glial fibrillary acidic protein) OR (5-hydroxytryptamine) OR (ionized calcium-binding adaptor molecule 1) OR (antioxidant stress) OR (reactive oxidative stress) OR (reactive oxygen species) OR (secondary messengers) OR (markers) OR (biomarkers))) AND (botulinum toxin)<br><br>***Filters**: Other animals.* ***118 selected items*** Sent On: Mon Jan 29 14:23:33 2024
SCOPUS	**botulinum** AND **toxin** AND **chronic** AND **inflammation** AND (EXCLUDE (DOCTYPE , "re") OR EXCLUDE (DOCTYPE , "cp") OR EXCLUDE (DOCTYPE , "sh") OR EXCLUDE (DOCTYPE , "ed") OR EXCLUDE (DOCTYPE , "no") OR EXCLUDE (DOCTYPE , "le") OR EXCLUDE (DOCTYPE , "tb") OR EXCLUDE (DOCTYPE , "er")) AND (LIMIT-TO (EXACTKEYWORD , "Animal Experiment") OR EXCLUDE (EXACTKEYWORD , "Animal Cell") OR EXCLUDE (EXACTKEYWORD , "Humans") OR EXCLUDE (EXACTKEYWORD , "Urinary Bladder") OR EXCLUDE (EXACTKEYWORD , "In Vitro Study") OR EXCLUDE (EXACTKEYWORD , "Human Cell") OR EXCLUDE (EXACTKEYWORD , "Urothelium") OR EXCLUDE (EXACTKEYWORD , "Overactive Bladder") OR EXCLUDE (EXACTKEYWORD , "Bladder Function") OR EXCLUDE (EXACTKEYWORD , "Cystitis")) AND (EXCLUDE (SUBJAREA , "PHYS") OR EXCLUDE (SUBJAREA , "COMP") OR EXCLUDE (SUBJAREA , "AGRI") OR EXCLUDE (SUBJAREA , "ENVI") OR EXCLUDE (SUBJAREA , "ENGI") OR EXCLUDE (SUBJAREA , "HEAL") OR EXCLUDE (SUBJAREA , "CENG") OR EXCLUDE (SUBJAREA , "MATE") OR EXCLUDE (SUBJAREA , "CHEM") OR EXCLUDE (SUBJAREA , "NURS")) AND (EXCLUDE (EXACTSRCTITLE , "Journal Of Urology") OR EXCLUDE (EXACTSRCTITLE , "American Journal Of Physiology Renal Physiology") OR EXCLUDE (EXACTSRCTITLE , "European Urology") OR EXCLUDE (EXACTSRCTITLE , "Prostate") OR EXCLUDE (EXACTSRCTITLE , "Urology") OR EXCLUDE (EXACTSRCTITLE , "BMC Urology") OR EXCLUDE (EXACTSRCTITLE , "Heart Rhythm") OR EXCLUDE (EXACTSRCTITLE , "Korean Journal Of Urology") OR EXCLUDE (EXACTSRCTITLE , "Luts Lower Urinary Tract Symptoms") OR EXCLUDE (EXACTSRCTITLE , "American Journal Of Physiology Heart And Circulatory Physiology") OR EXCLUDE (EXACTSRCTITLE , "American Journal Of Physiology Lung Cellular And Molecular Physiology") OR EXCLUDE (EXACTSRCTITLE , "American Journal Of Respiratory And Critical Care Medicine") OR EXCLUDE (EXACTSRCTITLE , "Neurourology And Urodynamics") OR EXCLUDE (EXACTSRCTITLE , "American Journal Of Physiology Gastrointestinal And Liver Physiology") OR EXCLUDE (EXACTSRCTITLE , "Cell") OR

Database	Search Format
	EXCLUDE (EXACTSRCTITLE , "Cell And Bioscience") OR EXCLUDE (EXACTSRCTITLE , "BMC Nephrology") OR EXCLUDE (EXACTSRCTITLE , "British Journal Of Nutrition") OR EXCLUDE (EXACTSRCTITLE , "Autonomic Neuroscience Basic And Clinical") OR EXCLUDE (EXACTSRCTITLE , "International Urology And Nephrology") OR EXCLUDE (EXACTSRCTITLE , "Journal Of Cosmetic Dermatology") OR EXCLUDE (EXACTSRCTITLE , "Advances In Radiation Oncology") OR EXCLUDE (EXACTSRCTITLE , "American Journal Of Respiratory Cell And Molecular Biology") OR EXCLUDE (EXACTSRCTITLE , "Antibiotics") OR EXCLUDE (EXACTSRCTITLE , "Cells") OR EXCLUDE (EXACTSRCTITLE , "Cardiovascular Diabetology") OR EXCLUDE (EXACTSRCTITLE , "Evidence Based Complementary And Alternative Medicine") OR EXCLUDE (EXACTSRCTITLE , "Lasers In Medical Science") OR EXCLUDE (EXACTSRCTITLE , "ACS Chemical Neuroscience") (Number of results: **266** On: Mon Jan 29 12:16:27 2024
WEB OF SCIENCE	**botulinum toxin** (All Fields) and **chronic** (All Fields) and **inflammation** (All Fields) and Retracted Publication or Editorial Material or Proceeding Paper **(Exclude – Document Types)** and Urology Nephrology or Gastroenterology Hepatology or Respiratory System or Pediatrics or Critical Care Medicine or Biochemistry Molecular Biology or Food Science Technology or Medicine General Internal **(Exclude – Web of Science Categories) 84** ***selected items (84 articles; 33 review articles)*** Sent On: Mon Jan 29 20:58:05 2024
**Website/Register**	**Search Format**
preclinicaltrials.eu<br>animalstudyregistry.org	Intervention/treatment = (“**Botulinum toxin**”)<br>Number of results : **0** On: Mon Jan 29 18:46:19 2024
PROSPERO	**4** records found for **botulinum toxin;** Source of the review: **Animal:DB** On: Mon Jan 29 19:02:34 2024
