# Supplementary material for: Botulinum Toxin Effects on Biochemical Biomarkers Related to Inflammation-Associated Head and Neck Chronic Conditions: A Systematic Review of Preclinical Research
Source: Toxins (Basel). 2025 Jul 29;17(8):377. doi: 10.3390/toxins17080377 (PMC12390450; doi:10.3390/toxins17080377)
Supplement: Supplementary file 1 [file toxins-17-00377-s001.zip › SR2. file S2 Table S1. Included Vs Excluded (requiring adjudication). docx.pdf]

File S2 Table S1: Biomarkers in Preclinical Research on Botulinum Toxin effects on Chronic Inflammatory State. Listed reasons for contacting the corresponding author as per protocol

REFERENCE	STUDY	CONDITION	BIOMARKER	INCLUSION/EXCLUSION
**CONTACT CORRESPONDING AUTHOR AS PER PROTOCOL**				
Baral, 2021 [35]	Preclinical animal study	Skin Fibrosis in Systemic Sclerosis	**Fibrotic skin**<br>**fibrosis markers -** α-SMA$^+$ myofibroblasts, protein CTGF<br>**Inflammatory cells and cytokines** - CD3$^+$ T cells and CD68$^+$ macrophages<br>**Apoptosis cells and related marker –** keratinocytes, vascular endothelial cells, fibroblasts, caspase-3<br>**Oxidative stress associated factors** - NOX2, HO-1, Trx2	√ (?) head and neck<br>"Unknown location for skin bleomycin-treated area/ BoNT injection"<br>"Scleroderma, or systemic sclerosis (SSc), is an immune-mediated chronic disorder with a systemic involvement characterized by small vessel alterations and progressive fibrosis of the skin and internal organs, such as lungs, gastrointestinal tract, and heart"<br>https://doi.org/10.3390/biomedicines10010163<br>**CONTACT Corresponding author as per protocol:** Sei-ichiro Motegi **Email:** **smotegi@gunma-u.ac.jp**
Choi, 2019 [36]	Preclinical animal + in vitro study	Rosacea-like inflammation	**Animal study:**<br>**Skin lesions**<br>**Rosacea biomarkers** – Cma1, KLK5, Mmp9, Trpv2<br>**Mast cell degranulation**<br><br>**In vitro study:**<br>**Skin lesions**<br>**BoNTA receptor -** Sv2<br>**SNARE proteins -** SNAP-25, VAMP2<br>**Mast cell degranulation**	Not head and neck<br>"rosácea-like inflammation" model was established by intradermally injecting LL-37" **unknown location** (**image seems to be from dorsum**)"<br><br>"Rosacea is a chronic inflammatory skin disorder which is characterized by facial flushing, telangiectasia and inflammatory papules and pustules on the central area of the face"<br><br>**CONTACT Corresponding author as per protocol:** Anna Di Nardo **Email:** adinardo@ucsd.edu<br>Confirmation of injections/assessments into the back.
Ward, 2012 [37]	Preclinical animal study	Psoriasis	**Skin**<br>**Inflammatory cells** - CD4$^+$ T cells, CD11c$^+$ DCs, CD8$^+$ T cells, F4/80$^+$ macrophages	(?) head and neck<br>"Unknown location for skin lesions area" but "BoNT and saline injected into **dorsal skin**"<br>"well-established *mouse model* of psoriasis, the *KC-Tie2* mouse"<br>**CONTACT Corresponding author as per protocol: Dr. Nicole L. Ward**<br>**Email:** nicole.ward@case.edu
Li, 2022 [38]	Preclinical animal study	chronic pain induced by osteoarthritis	**midbrain periaqueductal gray (PAG)**<br>expression level of N-methyl-D-aspartate receptor-2B (NMDAR2B)	? combined treatment? Head and neck? Unknown location for "BoNT Intraarticular injection"<br>"five groups: Sham group, MIA group (disease model), MIA + BoNT/A + tDCS group (transcranial direct current stimulation), **MIA + BoNT/A +**

				**StDCS group** (sham- transcranial direct current stimulation), as well as MIA + NS (saline) + tDCS group"<br>**CONTACT Corresponding author as per protocol: Dr. Tieshan Li Email: tieshanli@qdu.edu.cn**
Amalia, 2021 [39]	Preclinical animal study	Psoriasis	**Lesional skin**<br>**Nerve fibres** - PGP9.5^{+}<br>**Neuropeptides** - SP, CGRP<br>**Neuropeptide inhibitor** - CGRP antagonist ($CGRP_{8-37}$)<br>**Cytokines** – IL-17A/F<br>**Inflammatory cells** - CD4^{+} T cells, CD11c^{+} dendritic cells	√ (?) head and neck<br>"Unknown location for skin lesions area/ BoNT injection"<br><br>"Psoriasis is a systemic, immune-mediated disorder, characterized by inflammatory skin and joint manifestations."<br>https://doi.org/10.1111/j.1468-3083.2011.04410.x<br><br>**CONTACT Corresponding author as per protocol: Sei-ichiro Motegi Email: smotegi@gunma-u.ac.jp**
Liu, 2017 [40]	Preclinical animal study	Hypertrophic scar model	**Scar tissue**<br>**Scar proliferation** - hypertrophic index<br>**Angiogenesis** - CD31,<br>**Fibroblast proliferation** - Ki67, and TGF-β1 expression	? unknown timing for BoNT administration – wound or established hypertrophic scar?<br>**CONTACT Corresponding author as per protocol: Xiao-jing Li**<br>**Email: ay_lxj@yahoo.com**
