# Supplementary material for: Botulinum Toxin Effects on Biochemical Biomarkers Related to Inflammation-Associated Head and Neck Chronic Conditions: A Systematic Review of Preclinical Research
Source: Toxins (Basel). 2025 Jul 29;17(8):377. doi: 10.3390/toxins17080377 (PMC12390450; doi:10.3390/toxins17080377)
Supplement: Supplementary file 1 [file toxins-17-00377-s001.zip › SR2. file S3. Table S2. Summary of findings.pdf]

File S3. Table S2: Biomarkers in Pre-clinical Research on Botulinum Toxin effects on Chronic Inflammatory State. Summary of findings.

PRE-CLINICAL STUDIES (ANIMAL)				
**Author, Year**	**Study Design Population LOE**	**Condition CIS**	**Biological sampling Biomarker**	**BoNTA key effect Change in biomarker**
**INFLAMMATION-NEUROGENIC INFLAMMATION-NEUROINFLAMMATION**				
Makawi, 2022 [13]	(n=42) male adult Wister albino rats, age: 3-4 months (180 - 200g)<br>Induced TMJ osteoarthritis (OA) (4weeks period - a period equivalent to about 3 -4 years in humans) by injection of 2mg monosodium iodoacetate in both sides of TMJ.<br>(n=3) control group (no OA) right and left TMJs (6 specimens).<br><br>Follow-up: 2 weeks (histological, biochemically), 4 weeks post-treatment (radiographically, biochemically)<br><br>4 weeks following OA induction, rats were randomly divided – intraarticular 0.05ml injections weekly - left side control (saline 50μL), right side experimental Tx group:<br>G1. (n=14) Botox (5 IU/kg diluted in 50 μL saline),<br>G2. (n=14) 50μL PRP<br>G3. (n=14) BoNT + PRP<br>*(n=2, each group sacrificed for confirmation of osteoarthritis - histological, x-ray, PCR+ELISA (IL-1β) analysis.).	Temporomandibular joint (TMJ) osteoarthritis	**TMJ tissues**<br>matrix metalloproteinase-13 (MMP-13) and Interleukin 1 beta (IL-1β) levels (pg/ml tissue)<br><br>by PCR-ELISA<br><br>Other assessments: histological analysis, bone area percentages and joint space (mm) between head of condyle and temporal bone (CBCT)	• The treated sides showed statistically significantly ↑ mean bone area %, in addition to ↓ statistically significantly mean IL-1β and MMP13 levels than the untreated sides (p<0.001).<br>• Botox-treated samples showed a statistically significant lower mean bone area % than the other two treated groups (p<0.001).<br>• The treated sides recorded a ↑ mean joint space than the untreated sides that is statistically significant only in the Botox group (p<0.001).<br><br>BoNT GROUP:<br>• mean, SD values of ANOVA test for comparison between bone area %, IL-1β and MMP13 levels of the different groups:<br>at 2 weeks post-treatment:<br>- Normal: Bone area % (mean - 82.4a, SD – 2.9); IL-1β (mean - 72.2h, SD - 2.6); MMP13 (mean - 1.26E, SD - 0.10).<br>- Osteoarthritis: (mean - 41.5e, SD – 2.9); IL-1β (mean - 321.3d, SD – 4.4); MMP13 (mean - 3.55B, SD - 0.25).<br>- Botox (Treated side): Bone area % (mean - 63.7c, SD - 4.8); IL-1β (mean - 205.3f, SD - 10.6); MMP13 (mean - 2.24C, SD - 0.13).<br>- Botox (Untreated side): Bone area % (mean - 50.6d, SD - 2); IL-1β (mean - 405.3b, SD - 17.2); MMP13 (mean - 4.16A, SD - 0.75).<br>at 4 weeks post-treatment:<br>- Normal: Joint space (mm) (mean - 0.43A, SD – 0.09); IL-1β (mean - 72.2G, SD - 2.6); MMP13 (mean - 1.26G, SD - 0.10).<br>- Osteoarthritis: (mean - 0.14C, SD – 0.01); IL-1β (mean - 321.3D, SD – 4.4); MMP13 (mean - 3.55E, SD - 0.25).<br>- Botox (Treated side): Joint space (mm) (mean - 0.28B, SD – 0.03); IL-1β (mean - 113.6E, SD – 14.7); MMP13 (mean - 1.61F, SD - 0.06).<br>- Botox (Untreated side): Joint space (mm) (mean - 0.15C, SD – 0.02); IL-1β (mean - 464.6C, SD – 12.0); MMP13 (mean - 6.17B, SD - 0.05).
	LIMITATIONS: This study was approved by Institutional Animal Care and Use Committee (IACUC) in Cairo University (CUIII-F7417). The authors certify that there was no specific funding for the research and publication of this article and threre are no conflicts of interest. There is currently no consensus model for OA that naturally reflects human disease.			

	**https://doi.org/10.1016/j.joca.2018.09.016.** There is still not a clear "gold standard" for choosing the best animal model for OA. Currently, the most utilized type of chemical induction is MIA, which has an inhibitory activity of glyceraldehyde-3-phosphate dehydrogenase glycolysis and induces the death of chondrocytes **https://doi.org/10.3390/biology12020283**. One of the most important pro-inflammatory cytokines that contribute to the pathophysiology of osteoarthritis is IL-1β. IL-1β is considered an important biochemical marker to monitor the progression of osteoarthritis and efficacy of the intervention, since an increased production in IL-1β results in cartilage degradation. MMP-13 is considered one of the most common enzymes studied for cartilage degradation, due to its capacity in cleaving collagen type II which predominates in the articular cartilage.			
Shao, 2013 [19]	(n=32) adult female Sprague-Dawley rats (250-300g) age: unknown- migraine induced by nitroglycerin (NTG) – frontal and temporal subcutaneous injection.<br>Follow-up: 24h after vehicle or BoNT administration (2 ml blood withdrawn and medulla oblongata containing caudal trigeminal nucleus).<br>Subcutaneous (frontal and temporal) injection 2h after NTG.<br>G1. (n=8) NTG + vehicle<br>G2. NTG + BoNT<br>G2a.(n=8) 5U/kg or G2b. (n=8) 10U/kg<br>G3. (n=8) Control	Migraine	**Jugular plasma and medulla oblongata - containing caudal trigeminal nucleus**<br>CGRP, SP (pg/mL)<br><br>CGRP- and SP-like immunoreactivity (CGRP-LI and SP-LI) determined by radioimmunoassay.	• Values presented as means ± SEM. Quantitative data analysed by one-way ANOVA. For binomial qualitative data, comparisons between groups (chi-square).<br>• NTG induced marked ↑ of CGRP-LI levels in jugular plasma (1.8-fold, P<0.01) and oblongata (1.8-fold, P<0.05) and SP-LI levels in jugular plasma (2.14-fold, P<0.01) and oblongata (3.14-fold, P<0.01), both compared with control.<br>• Local BoNT injection (2h after NTG) suppressed NTG-induced CGRP-LI in jugular plasma (P<0.01) and oblongata (P<0.01), and SP-LI levels in jugular plasma (P<0.05) and oblongata (P<0.01), both compared with vehicle.<br>• The inhibitory effect of (G2b) BoNT 10 U/kg on both CGRP-LI and SP-L levels was not different from (G2a) BoNT 5 U/kg (P>0.05).<br>• The evidence that BoNT ↓ NTG-induced CGRP-LI and SP-LI levels in trigeminovascular system suggests that BoNT attenuates migraine by suppression of neuropeptide release. This may occur via a SNAP-mediated mechanism, similar to the blockade of acetylcholine release in motor neurons.
	LIMITATIONS: The model of migraine induced by NTG, a nitric oxide (NO) donor, has been widely used in animals or humans. NTG-induced migraine attack appears to be a right model for assessing changes in vasoactive neuropeptides as CGRP and SP which are likely to occur in a spontaneous crisis. Peptide levels obtained in vessel oblongata are so close to the trigeminovascular system that could be considered as reliable predictors of migraine attack. Neuropeptides (CGRP) and substance P (SP) convey nociceptive impulses to the brain and at the same time may co-release CGRP and SP from the peripheral endings, thus evoking a variety of effects collectively known as neurogenic inflammation. All the protocols followed the guidelines of the International Association for the Study of Pain and the European Communities Council (86/609/ ECC) and were approved by the Institutional Animal Care and Use Committees of Gansu Province Medical Animal Center and Lanzhou University. This work was supported by Scientific and Technique Support Project of Gansu Province and Medical Subject Fund of Lanzhou University.			
Li, 2019 [21]	(n=?, 4 mice housed by each cage) male ICR mice (6–8 weeks, 20g–25g). Mouse model of depression induced by spatial restraint stress (SRS).<br>Follow-up: 1,7,14 days<br>BoNT 0.18U single facial intramuscular injection (3 points at each cheek)<br>Imipramine, 10mg/kg, fluoxetine 10 mg/kg injected intraperitoneally daily.<br><br>G1. Control (naïve)<br>G2. Spatial restraint stress (SRS) model<br>G3. G1 + BoNT<br>G4. G2 + BoNT	Depression<br><br>(chronic mild stress depression model)	**Hippocampus, hypothalamus, prefrontal cortex, amígdala (Brain)**<br>**Neurotrophic factors** – BDNF (mRNA (RT-PCR), Protein expression (Western blotting analysis))<br>**Synaptosomal-associated protein 25** (SNAP25), protein expression<br>**N-methyl-D-aspartate receptor (NMDAR) subunits** - NR1, NR2A and NR2B<br>**Precursor for neurotransmitter (serotonin)** – 5-HT (ng/g)	• All data are presented as mean ± SEM. Differences between two groups were determined with Student's t-test. One-way ANOVA with the Bonferroni post-test was used for multiple group comparisons.<br>**•Naïve mice (G1):**<br>- (G3) BoNT Tx Improves Depressive-Like Behaviours in Naïve Mice (n = 5–10/group).<br>- (G3) BoNT Tx significantly ↑ 5-HT Levels in the Hypothalamus of Naive Mice (F(2,15) = 3.811, P = 0.0459) (n = 6–7), but not in the hippocampus and prefrontal cortex. These data indicated that ↑ 5-HT levels in the hypothalamus may be involved in the anti-depressive activity of BoNT in naıve mice, although the mechanisms underlying this up-regulation remain to be determined.<br>- (G3) protein expression of BDNF in the hippocampus did not change after BoNT Tx in naïve mice (n = 6).<br>- (G3) mRNA expression of BDNF was not altered in the hippocampus, hypothalamus, and prefrontal cortex after BoNT Tx (n = 5-6). Thus, ↓ expression of the NMDAR subunit NR2A in the hippocampus is likely involved in the anti-depressive effects of BoNT Tx in naïve mice.

			(using HPLC)<br>**Neuroprotective and growth promoters** -<br>p-ERK, p-CREB<br>measured with HPLC analysis, RT-PCR, western blotting<br><br>Behavioural assessments: forced swimming test, tail suspension test, sucrose preference test, body weight, rotarod test (motor function), open field test (locomotory activity)	- (G3) expression of NR1 and NR2B in the hippocampus did not change, but the expression of NR2A in the hippocampus was significantly ↓ from 1h to 1 day after BoNT Tx (F(4, 25) = 11.74, P\0.0001)(n = 6).<br>- (G3) expression of SNAP25 in the hippocampus was not altered after BoNT Tx, suggesting that peripherally-administered BoNT may be not able to reach the hippocampus (n = 6).<br>**•SRS mice (G2) - display robust depression-like behaviors, 5-HT levels were significantly ↓ in the hippocampus (t = 3.383, P = 0.0277) and hypothalamus (t = 6.596, P = 0.0027), but not in the prefrontal cortex, protein expression of BDNF in the hippocampus was ↓ (t = 3.500, P = 0.0057), mRNA expression of BDNF was ↓ in the hippocampus (t = 4.117, P = 0.0146) and amygdala (t = 4.129, P = 0.0145)** and **did not change in the prefrontal cortex and hypothalamus, expression of phosphorylated ERK (p-ERK) and p-CREB was suppressed in the hippocampus (t = 5.350, P = 0.0003; t = 4.034, P = 0.0024) - compared with control (G1), NMDAR subunits NR1 (t = 5.102, P = 0.0005) and NR2B (t = 4.529, P = 0.0011) were ↓, while NR2A did not change in the hippocampus in SRS-treated mice vs. controls (G1) (t = 0.8309, P = 0.4254):**<br>- (G4) BoNT Tx Improves Depressive-Like Behaviors in Mice Undergoing SRS (BoNT has significant antidepressant-like effects in SRS mice).<br>- (G4) BoNT Tx significantly ↑ 5-HT Levels in the Brain of SRS Mice (n = 6–7/group) - hippocampus (F(4, 10) = 21.46, P <0.0001), hypothalamus (F(4, 10) = 12.29, P = 0.0007), and prefrontal cortex (F(4, 10) = 7.101, P = 0.0056). Thus, these data indicated that ↑ 5-HT levels in distinct brain regions may be involved in the antidepressant-like effects of BoNT therapy in SRS mice.<br>- (G4) BoNT Tx Causes Up-Regulation of BDNF Expression in the Brain of SRS Mice: BoNT significantly ↑ protein expression of BDNF (F(5, 30) = 10.37, P\0.0001) (n = 6).<br>- (G4) BoNT Tx did not alter the expression of SNAP25 in control and SRS mice (n=6).<br>- (G4) BoNT Tx remarkably ↑ expression of NR1 (F(5, 30) = 15.50, P<0.0001) and NR2B (F(5, 30) = 4.758, P = 0.0026) in the hippocampus in SRS mice (n=6). These results indicated distinct NMDAR subunits, NR1 and NR2B, are involved in the antidepressant like effects of BoNT Tx.<br>- (G4) BoNT Tx significantly ↑ mRNA expression of BDNF in the hippocampus during 1–7 days post-injection of BoNT (F(4, 10) = 8.700, P = 0.0027) and during 1–14 days post-injection in the amygdala (F(4, 10) = 7.423, P = 0.0048) (n=6).<br>- (G4) BoNT Tx transiently ↑ mRNA expression of BDNF also in the brain regions (prefrontal cortex and hypothalamus) in SRS mice F(4, 10) = 7.727, P = 0.0042; F(4, 10) = 6.459, P = 0.0078). Data suggested that up-regulation of BDNF expression in the brain may contribute to the antidepressant-like effects of BoNT Tx.<br>- (G4) BoNT Tx ↑ expression of p-ERK and p-CREB one day after BoNT Txt (F(5, 30) = 5.810, P = 0.0007; F(5, 30) = 8.619, P\0.0001). Data suggested that transient up-regulation of p-ERK and p-CREB may be involved in the antidepressant-like effects of BoNT Tx.<br>**Overall:**

				•G3 and G4 significantly ↑ the 5-HT levels in several brain regions, including the hippocampus and hypothalamus. (G3: HPLC. *P <0.05 *vs* G1; G4: HPLC. **P <0.01, ***P <0.001 *vs* G1; $^{\#}P$ <0.05, $^{\#\#}P$ <0.01, $^{\#\#\#}P$ <0.001 *vs* G2).<br>•Overall, these results demonstrated that BoNT has anti-depressant-like activity in mice, and this is associated with ↑ 5-HT levels and the activation of BDNF/ERK/CREB pathways in the hippocampus.<br>•Motor coordination and locomotor activity was not altered by BoNT, indicating that BoNT has anti-depressive effects in native mice without affecting motor function.
	LIMITATIONS: No placebo used. Repeated SRS causes depression-like behaviors in rodents, and it has been widely used as a preclinical model of depression. Reports have shown that reduced 5-HT levels in the brain contribute to the pathogenesis of depression in both animal models and patients. Studies have pointed out that a decreased BDNF level is involved in the pathogenesis of depression and antidepressants increase the production of BDNF in the hippocampus. A role of N-methyl-D-aspartate receptor (NMDAR) subunits, such as NR1, NR2A and NR2B, has been implicated in the pathogenesis of depression. The intracellular ERK-CREB pathway has been demonstrated to play important roles in neurotrophin signaling and neurogenesis, which are involved in the pathogenesis of depression. All animal experiments were performed according to the National Institutes of Health Guide for the Care and Use of Laboratory Animals. All animal care and experimental procedures were approved by the Institutional Animal Care and Use Committee of Soochow University. The work was supported by grants from the National Natural Science Foundation of China, and the Natural Science Foundation of Jiangsu Province, China, Jiangsu Key Laboratory of Neuropsychiatric Diseases, the Second Affiliated Hospital of Soochow University Preponderant Clinic Discipline Group Project Funding, the Postgraduate Research and Practice Innovation Program of Jiangsu Province, China, Suzhou Science and Technology For People's Livelihood, the Postgraduate Research and Practice Innovation Program of Jiangsu Province, China.			
Chen, 2021 [14]	(n=48) Male C57BL/6 mice 6-8 week-old, 20g (modified TN model established by CCI of the distal infraorbital nerve surgery) + TLR2 knockout mice (Tlr2−/− ; stock#005846)<br>Follow-up: 5 day after BoNT/19-day after CCI (western blotting, RT-PCR, immunofluorescence)<br>2-weeks after CCI - BoNT (0.18U) subcutaneous unilateral peripheral (facial) injection into the whisker pad on the ipsilateral side.<br><br>G1. Sham G (n=12)<br>G2. CCI+Vehicle-treated TN (n=18)<br>G3. CCI+BoNT (n=12) 2-weeks after BoNT-analgesic effect worn off (lasted about 9-days), a second injection performed.<br>G4. Also tested the pain behaviours of TN in CCI+*Tlr2*$^{-/-}$ (n=6) - to investigate the role of TLR2 on the development of persistent pain in the CCI mouse model.	Trigeminal Neuralgia (TN) induced by IoNC - neuropathic pain and anxiety-like behaviors	**Trigeminal nucleus caudalis (TNC)**<br>mRNA or protein expression levels<br>**toll-like receptors (TLRs) –** TLR1, TLR2, TLR5, TLR4, TLR8, TLR11, MyD88<br>**glia activation markers (microglia marker) -** CD11b, F4/80 (macrophage or microglia marker), IBA-1<br>**neuron activation marker-** c-Fos<br>**proinflammatory factors -** IL-1β, TNF-α, IL-6<br>(tested by RT-qPCR, immunofluorescence, Western blotting).<br><br>Other assessments: Bilateral mechanical pain (von Frey test), Hypersensitivity (open field), Anxiety-like behaviour (elevated	•**CCI surgery**: **Ipsilateral side TNC** - significantly ↑ the expression of TLR2 and TLR5, MyD88, F4/80, CD11b, c-Fos, IL-1β, TNF-α, IL-6 and IBA-1. Also, a significantly ↓ in the mRNA expression TLR11 (sham vs. CCI+vehicle, TLR2: t6 = 6.307, P = 0.0007; TLR5: t6 = 7.584, P = 0.0003; TLR11: t6 = 10.8, P < 0.0001; F480: t6 = 2.965, P = 0.0351; CD11b: t6 = 0.3945, P = 0.0076; c-Fos: t6 = 4.707, P = 0.0033; IL-1β: t6 = 8.633, P = 0.0001; TNF-α: t6 = 8.990, P = 0.0001; IL-6: t6 = 5.629, P = 0.0013; IBA-1: t4 = 2.949, P = 0.0420; MyD88: t6 = 2.718, P = 0.0347); **Contralateral side TNC** - CCI surgery and sham groups showed no significant change in the mRNA expression of TLRs, mRNA expression of c-Fos was significantly ↑ in the CCI group compared with sham G (sham vs. CCI: t6 = 4.022, P = 0.0069), there was no statistically significant difference in the expression of proinflammatory factors compared with sham G and IBA-1 expression (IBA-1, ipsilateral TNC vs. contralateral side of CCI: t4 = 4.332, P = 0.0123); expression of MyD88 did not change (sham vs. CCI, t6 = 0.9386, P = 0.3842)<br>•Data was analyzed using Graph Prism 6 (Graph Pad, La Jolla, CA). Shapiro-Wilk test was used to test normality of the data. All values were presented as mean ± SEM. Unpaired Student's t-test was used to compare two groups. Two-Way Repeated Measures ANOVA with post-hoc Bonferroni test was performed for multiple comparisons. Data from four mice were used for statistical analysis – at least for RT-qPCR, and Western blotting.<br>•TN was successfully established – unilateral ligation of the distal infraorbital nerve caused bilateral mechanical pain hypersensitivity in the whisker pad lasting for at least 5 weeks.<br>•G3 significantly attenuated CCI-induced bilateral mechanical pain hypersensitivity (appeared after 1h, lasted 9 days) and anxiety-like behaviours (but not depression-like behavior) induced by

	Randomly assigned, blinded to investigator (group of mice during testing was blinded to the experimenter of the behavioral test).		plus-maze testing), and (depressive-like behaviour) forced swimming test	CCI surgery in mice, which was associated with ↓ microglia activation, ↓ expression of TLR2, and ↓ expression of several proinflammatory mediators in the TNC.<br>•(G3) **Facial injection of BoNT down-regulated the expression of toll-like receptors (TLRs) in the TNC of CCI mice**:<br>-(G3) showed: **Ipsilateral side TNC** - a significant ↓ in the mRNA expression of TLR1, TLR2, TLR4, TLR5 and TLR8, compared with (G2) (CCI + vehicle vs. CCI + BoNT, TLR1: $t6 = 3.393$, $P = 0.0146$; TLR2: $t6 = 6.058$, $P = 0.0009$; TLR4: $t6 = 8.721$, $P = 0.0001$; TLR5: $t6 = 6.897$, $P = 0.0005$; TLR8: $t6 = 5.766$, $P = 0.0012$). **Contralateral side TNC** - expression TLR1, TLR4 and TLR8 were significantly ↓ on day 5 after BoNT injection compared with vehicle-treated (G2) (TLR1: $t6 = 6.614$, $P = 0.0006$; TLR4: $t6 = 6.963$, $P = 0.0004$; TLR8: $t6 = 2.7$, $P = 0.0356$). suggested that changes of TLRs expression in the TNC were involved in the pathogenesis of TN and BoNT Tx significantly down-regulated the mRNA expression of TLRs, particularly TLR2 and TLR5.<br>•(G3) **inhibitory effects of BoNT Tx on the mRNA expression of glia activation markers and proinflammatory mediators in the TNC of the CCI mice:**<br>-(G3) – **Ipsilateral side**: BoNT Tx significantly ↓ markers (F4/80, CD11b, c-Fos) on day 5 after BoNT injection (CCI + vehicle vs. CCI + BoNT, F4/80: $t6 = 4.799$, $P = 0.003$; CD11b: $t6 = 4.994$, $P = 0.0025$; c-Fos: $t6 = 5862$, $P = 0.0011$) and mRNA expression of proinflammatory factors (CCI + vehicle vs. CCI + BoNT IL-1β: $t6 = 10.39$, $P < 0.0001$; TNF-α: $t6 = 9.878$, $P < 0.0001$; IL-6: $t6 = 4.657$, $P = 0.0035$). Also, MyD88 was significantly ↓ (CCI + vehicle vs. CCI + BoNT, $t6 = 2.654$, $P = 0.0378$). **Contralateral side**: BoNT significantly ↓ the upregulation of mRNA expression of c-Fos compared with that of vehicle-treated group (G2) (CCI + vehicle vs. CCI + BoNT, $t6 = 8.647$, $P = 0.0001$). suggested that BoNT injection inhibited microglia activation and upregulation of proinflammatory factors and significantly ↓ bilateral activation of neurons in the TNC CCI-induced, which may be associated with analgesic effects of BoNT on mirror image pain. Also, MyD88 did not change in contralateral side (CCI vs. CCI + BoNT/A, $t6 = 0.5799$, $P = 0.5831$)<br>•(G3) **inhibitory effects of BoNT Tx on upregulation of the expression of TLR2 and IBA-1 in the ipsilateral side of CCI**:<br>- The upregulation of TLR2 and IBA-1 was significantly suppressed on day 5 after BoNT injection in CCI. These results suggested that microglia activation was inhibited and co-localization of IBA-1 and TLR2 was ↓ after BoNT injection in the TNC on the surgical side.<br>•(G3) **BoNT ↓ the upregulation of MyD88 expression after CCI and TLR2 deficiency also ↓ MyD88 expression in the TNC and alleviated bilateral mechanical pain hypersensitivity:**<br>- **Ipsilateral side** - upregulation of the expression of MyD88 was significantly ↓ on day 5 after BoNT injection (CCI + vehicle vs. CCI + BoNT, $t6 = 2.654$, $P = 0.0378$). **Contralateral side** - expression of MyD88 did not change (CCI vs. CCI + BoNT, $t6 = 0.5799$, $P = 0.5831$).<br>• On day 14 after CCI, the expression of MyD88 in the bilateral TNC of Tlr2−/− mice was significantly ↓ compared with that of wild-type mice (ipsilateral: $t4 = 2.923$, $P = 0.0431$; contralateral: $t4 = 3.438$; $P = 0.0138$) data indicated that MyD88 as downstream molecule of

				TLR signalling is involved in the pathogenesis of TN and BoNT injection could ↓ expression level of MyD88 in the ipsilateral TNC.<br>•Second facial injection of BoNT produced similar analgesic effect in the ipsilateral side of CCI surgery in mice and was also evident on the contralateral side (mirror image pain).<br>•BoNT attenuated anxiety-like behaviours and the analgesic effects may be associated with the inhibition of TLR2-mediated neuroinflammation in the TNC (G4: TLR2 deficiency alleviates bilateral mechanical pain hypersensitivity in the CCI mice).<br>•Peripheral administration of BoNT was able to suppress microglia-mediated neuroinflammation in the central nerve system.
	LIMITATIONS: only male mice (unbalanced sample); there are several other TN animal models; flaccid paralysis of the facial muscles at the injection site (potential side effects). TLR2 gene expression is known to be used as a reliable marker of activated microglia in vivo and studies have also suggested that TLR2 is involved in the pathogenesis of neuropathic pain models. All procedures were approved by the Animal Care and Use Committee of Soochow University. The number of mice used in each experiment are made available. This animal study followed the ARRIVE guidelines. This work was supported by Suzhou science and technology plan key technology application research project (SS2019060), and the National Natural Science Foundation of China (81671270; 81870874).			
Han, 2017 [27]	(n=42) NC/Nga Mice (mouse model for atopic dermatitis) + contact sensitizer (TNCB)<br>Follow up: day-14 (histology) after 1st challenge<br>BoNT (Botox) - single intradermal injections on the rostral back on the day of TNCB sensitization.<br>Test area limited to 1.5x1.5 cm)<br><br>G1. (n=6) Untreated (control)<br>G2. (n=6) 2-Chloro-1,3,5-trinitrobenzene [TNCB] alone<br>G3. (n=9) TNCB + BoNT 30 U/kg<br>G4. (n=9) TNCB + BoNT 60 U/kg<br>G5. (n=6) TNCB + vehicle [0.9% saline]<br>G6. (n=6) TNCB + 0.03% tacrolimus<br>*outcome assessor masked to study purpose and hypothesis, and not involved in Tx administration and assignment.	Atopic Dermatitis	**Rostral dorsal Skin + Serum**<br>**Mast Cell count** (per 5 high power fields)<br>**Cytokine** – IL-4 mRNA + protein expression (ng/mL).<br>(via RT-PCR)<br>**Total IgE** (serum – retro orbital plexus) (ng/mL).<br><br>Other assessments: skin thickness, transepidermal water loss (TEWL), skin severity scores, histological/ laboratory tests.	•TNCB, compared to control: marked epidermal thickening, mRNA and protein expression of IL-4 significantly ↑, IgE levels markedly ↑ – on day-14.<br>•BoNT significantly ↓ skin thickness and TEWL in the TNCB-applied skin.<br>•Data expressed as the mean ± SEM. The clinical severity scores, acanthosis and mast cell infiltration, were ↓ in G3 and G4. The **mast cell number** (per 5 high power fields) was significantly ↓ in G3 (28.1±4.70 vs. (G1) p=0.004, vs. (G2) p=0.000), G4 (17.7±2.69 vs. (G1) p=1.000, vs. (G2) p=0.000), G6 (55.2±10.3 vs. (G1) p=0.000, vs. (G2) p=0.000) compared to G2 (85.3±7.55 vs. (G1) p=0.000) and G5 (76.5±8.64 vs. (G1) p=0.000, vs. (G2) p=0.366). Control (G1) = 13.4±3.65 vs. (G2) p=0.000.<br>•BoNT (G3 and G4) and G6 significantly ↓ TNCB-induced increase in **IL-4 mRNA and protein** expression in mice (ng/mL). In **G1** (mRNA:0.90±0.36 vs. (G2) p=0.000; protein:1.00±0.84ng/mL vs. (G2) p=0.000), **G2** (mRNA:174±44.9 vs. (G1) p=0.000; protein expression:69.3±13.4ng/mL vs. (G1) p=0.000), **G3** (mRNA:53.0±17.6 vs. (G1) p=0.004, vs. (G2) p=0.000; protein expression (20.2±4.84ng/mL vs. (G1) p=0.44, vs. (G2) p=0.000), **G4** (mRNA:44.3±7.38 vs. (G1) p=0.028, vs. (G2) p=0.000; protein expression: 17.4±5.72ng/mL vs. (G1) p=0.148, vs. (G2) p=0.000), **G6** (mRNA: 84.9±20.2 vs. (G1) p=0.000, vs. (G2) p=0.000; protein expression: 49.0±16.4ng/mL vs. (G1) p=0.000, vs. (G2) p=0.37 compared to **G5** (mRNA:161±27.4 vs. (G1) p=0.000, vs. (G2) p=1.000; protein expression: 67.6±16.6ng/mL vs. (G1) p=0.000, vs. (G2) p=1.000)(p<0.01)<br>•BoNT effect on serum **IgE** level was not significant (ng/mL). At day-14, were significantly ↑ in G2 and G5 compared to G1 (G2:103±27.4 vs. (G1) p=0.000; G5: 112±46.5ng/mL vs. (G1) p=0.000, vs. (G2) p=1.000; vs G1: 2.93±1.76ng/mL)(p<0.01). G3, G4 and G6 failed to supress TNCB-induced IgE serum levels - G3 and G4 (G3: 68.7±13.5 vs. (G1) p=0.011, vs. (G2) p=0.765; G4: 66.5±29.7ng/mL vs. (G1) p=0.15, vs. (G2) p=0.573) and G6 (118±49.4ng/mL vs. (G1) p=0.000, vs. (G2) p=1.000)
	LIMITATIONS: NC/Nga mice have been considered one of the most valuable mouse models representing human AD. However, the low incidence of AD-like lesions, late onset of the disease, and poor reproducibility are disadvantages. The authors adopted contact sensitizers to the NC/Nga mice model. Data limited to 14 days, further studies need to identify the duration of effects of BoNT. The exact			

	dose should be confirmed in future studies. Small sample size. Supported by a grant of the Korean Healthcare technology R&D project, Ministry of Health & Welfare, Republic of Korea. The authors have indicated no significant interest with commercial supporters. Diffusion of BoNT is largely dependent on its volume and concentration, thus, the test area was limited to 1.5x1.5cm.			
Muñoz-Lora, 2022 [22]	(n=40) arthritic model – male Sprague–Dawley rats (300–400g, 6–8 weeks Induced persistent immunogenic hypersensitivity-PIH (systemic immunization mBSA/PBS+CFA (day-0,-7,-14) and TMJ intraarticular injection (day-21,-28,-35) - methylated bovine serum albumin (mBSA) + low-dose formalin (0.5%)<br>Follow-up: day-13 (pre-formalin), day-14 (post formalin)/day-56 (behavioural assessment), day-57 (behavioural assessment, sacrifice, biochemical assessment).<br><br>*randomly assigned into 4 groups:<br>G1. (n=10) control (non-induced PIH) + saline (no systemic immunization, TMJ intraarticular injection mBSA (day-0,-7,-14) + TMJ intraarticular injection saline (day-21,-28,-35,-42)<br><br>Unilateral injection into left TMJ at day-42:<br>G2. (n=10) induced (PIH) + Vehicle (saline - NaCl 0.9%)<br>G3. (n=10) PIH+AboBoNT (14U/Kg)<br>G4. (n=10) PIH+OnaBoNT (7U/Kg)	Arthritis - Immunogenic Hypersensitivity - Temporomandibular joint (TMJ) rheumatoid arthritis	**Trigeminal nucleus caudalis (TNC)**<br>**SNARE proteins** - SNAP-25<br>**Glial markers (astroglial) -** GFAP<br>**Neuron activation marker**- c-Fos<br>**Neuropeptide** – CGRP<br>Central effects assessed by immunohistochemical analysis.<br><br>Other assessments - measurement of spontaneous nociception by examining facial grimacing related to pain (RGS), evoked pain by examining facial mechanical allodynia over the skin covering the stimulated TMJ (Frey filaments) – behavioural assessments pre-formalin (day-13) + post-formalin (day-14)	•**Pre-formalin** - Lack of evoked and spontaneous allodynic responses, painful facial expression (score = 0) and a low number of behavioral nocifensive responses in all groups;<br>**Post-formalin** - bilateral mechanical allodynia over the area of TMJ, ↑ values of RGS, a significant ↑ in the nocifensive responses (facial grimacing, head flinching, facial rubbing), ↑ levels of c-Fos-positive nuclei in both ipsilateral and contralateral TNC, ipsilateral ↑ in astrocytic marker glial fibrillary acidic protein (GFAP) on induced animals compared to noninduced.<br>•Quantitative data were presented as the mean ± standard error of the mean (SEM):<br>•Both G3 and G4 significantly ↓ the formalin-induced spontaneous pain-related behaviours (nocifensive response), mechanical evoked TMJ bilateral allodynia, ↓ RGS score (at later time points (9–14 and 18–23 min) of the hypernociceptive rats.<br>•Effects were associated with the central occurrence of **cSNAP-25** (cSNAP-25 on the contralateral (nontreated) side was not observed, only in the ipsilateral trigeminal sensory regions (TNC) (10 sections per animal (n=3/Tx group).<br>•Effects were associated with significant ↓ of c-Fos (neuronal activation) and GFAP upregulation in both ipsilateral and contralateral TNC, 14 days after Tx. **GFAP** (analysis was performed on 5 randomly selected slices per animal (n=5 animals/group) Mean ± SEM; *** $p < 0.001$ and * = $p < 0.05$ vs. noninduced + saline and +++ = $p < 0.001$ and + = $p < 0.05$ vs. induced + saline ($F_{3,16}$ (GFAP area) = 73.14; $F_{3,16}$ (GFAP gray value) = 23.21; one-way ANOVA followed by Tukey's multiple comparisons test); **c-Fos** (c-Fos-expressing neuronal profiles (5 sections/animal, n = 5 animals/Tx group) Mean ± SEM; +++ = $p < 0.001$ and ++ = $p < 0.01$ vs. induced + saline ($F_{5,24}$ = 20.1, one-way ANOVA followed by Tukey's post hoc test).<br>•The levels of CGRP expression were not ↑ in the hypernociceptive rats or altered by G3 or G4. **CGRP** (analysis was performed on 5 randomly selected slices per animal (n=5 animals/group) Mean ± SEM; ($F_{3,16}$ (ipsilateral) = 0.3819; $F_{3,16}$ (contralateral) = 1.267; one-way ANOVA).<br>•BoNT antinociceptive activity on the immunogenic hypersensitivity is associated with the toxin axonal transport to trigeminal sensory areas and reduction of neuronal and glial activation in central nociceptive regions, alongside peripheral action.<br>•↓ of neuronal and astrocyte activation suggest the toxin's central actions.
	LIMITATIONS: There are many animal models of RA-related pain, including TMJ-RA, that have been proposed and even though this model resembles human TMJ-RA it has a monoarthritic (single joint) presentation, since it is induced locally inside the mBSA-stimulated joint. Also, this arthritic model produces a delayed-type hypersensitivity (viz., type IV hypersensitivity) and is characterized by central sensitization due to immune and neuronal cells activation, leading to the local release of a variety pronociceptive factors. This fact increases the validity of the model to study the progression of pain on chronic inflammatory conditions. Experiment is restricted to the trigeminal region, so differences in the mechanism of action of BoNT applied to other extracranial joints (e.g., knee and ankle) and actions at the spinal levels cannot be excluded. Additionally, during the period after BoNT-A treatment, the aboBoNT- or onaBoNT-treated rats did not exhibit a significantly slower weight gain compared to the saline-treated rat groups (p = 0.070, one-way ANOVA, results not shown). This excludes possible systemic effects or a local muscular BoNT action (e.g., mastication and feeding) interfering with the animal weight gain. This study was funded by Ipsen. Mikhail Kalinichev is an employee of Ipsen Innovation, France.			
Muñoz-Lora, 2017 [15]	(n=?) Male Wistar rats (250–500g) arthritis-induced persistent inflammatory hypernociception (PIH) in the left TMJ	Arthritis – persistent inflammatory hypernociception in	**Peri-articular tissues from TMJ and Trigeminal ganglia**	•BoNT (G1,G2,G3) ↓ the persistent inflammatory hypernociception induced by arthritis in the TMJ of rats without differences among groups. Established the dose of the BoNT at 7 U/Kg (G2) for the next experiments.

	(methylated bovine serum albumin (mBSA)/phosphate-buffered saline (PBS) + Freund's complete adjuvant (CFA) (immunization + booster after 7-, and 14-days – systemic injection into the back) + mBSA intraarticular injection into TMJ (7-,14-,21.days after last immunization/21-,28-,35-days of experience) + intraarticular injection of low dose of formalin (0.5%) 7-days after the last challenge/day-35 in immunized rats (day-43 of experience).<br>Follow-up: 24h or 14 days after BoNT injection intra-TMJ injection<br><br>G0. mBSA intraarticular injection into TMJ (Control, non-immunised)<br>At day-42/7days after last challenge of TMJ induction, intra-TMJ injection:<br>G1. PIH+BoNT 3.5U<br>G2. PIH+BoNT 7U<br>G3. PIH+BoNT 14U<br>G4. PIH+Saline (NaCl-0.9%)<br>G5. PIH	temporomandibular joint (TMJ)	**Neurotransmitters** – CGRP (ng/mL), SP (ng/mL), glutamate (nmol)<br>**Cytokine** - IL-1β, TNF-α (pg/mL)<br><br>using histopathological immunofluorescent staining - . ELISA and Western Blot analyses.<br>Other assessments – behavioural nociceptive tests	•BoNT (G2) significantly ↓ the peripheral release of SP and CGRP; and the pro-inflammatory cytokine IL-1β ($P < 0.05$; ANOVA: Tukey's test).<br>•BoNT had no effect in the peripheral release of glutamate and the cytokine TNF-α ($P > 0.05$; ANOVA: Tukey's test).<br>•Intra-articular injection of BoNT ↓ the albumin-induced arthritis persistent hypernociception in TMJ of rats by peripheral inhibition of neuropeptides release and certain pro-inflammatory cytokines.<br>•The present study showed no ↓ of TNF-α quantity, suggesting no anti-inflammatory effects of BoNT; thus, reduction of IL1-β quantity after BoNT-A application may be due to the inhibition of neurotransmitters SP and CGRP, which in turn ↓ the inflammatory chemotaxis and consequently ↓ the release of this cytokine.
	LIMITATIONS: study was approved by the Ethics Committee in Animals Research of the State University of Campinas, following the National Council for Control of Animal Experimentation (CONCEA) and the International Association for the Study of Pain (IASP) guidelines. Different circumstances experiment and control group ? "After 24 h or 14 days of BoNT treatment, an intra-articular injection of formalin (0,5%) was administered. Immediately after the formalin injection, the behavioral nociceptive response was evaluated. After behavioral evaluation, animals were terminally anesthetized and trigeminal ganglia were removed and stored".			
Muñoz-Lora, 2020 [16]	(n=40) male Wistar rats (300–400 g)<br>Model of antigen-induced arthritis by systemic injection into the back of CFA and mBSA diluted in PBS (booster at 7-, 14-days) + mBSA intraarticular injection into TMJ for 3 week (day-21,-28,-35 of experiment).<br>Follow-up: 24h, 7-, **or** 14-days (n=8/group):<br>G1. non-immunized (control group) – injected with mBSA/PBS + intraarticular mBSA into TMJ.	Arthritis temporomandibular joint (TMJ)	**Trigeminal subnucleus caudalis microglia-activated pathway/ protein level:**<br>**microglial purinergic** - P2X7 receptor and CX3 chemokine receptor 1 (CX3CR1) (OD) (by Western blot)<br>**microglial-neuron modulators** - Cathepsin S (CatS)/Fractalkine (FKN) (pg/mL)<br>**pro-inflammatory cytokines** - TNF-α, IL-1β (pg/mL)	•(G2) antigen-induced arthritis in the TMJ significantly ↑ the protein levels of P2X7, CatS, FKN, TNF-α and IL-1β in the trigeminal subnucleus caudalis ($P < 0.05$), in comparison to (G1).<br>•Data were analysed using one-way analysis of variance (ANOVA) with post hoc contrasts using the Tukey's test. Data are presented in figures as mean ± standard deviation (SD).<br>•BoNT intra-TMJ injection significantly ↓ protein levels of P2X7 in 24h and it was maintained all time points tested ($P < 0.05$).<br>•CX3CR1 protein level did not differ among groups ($P > 0.05$).<br>•BoNT significantly ↓ protein levels of CatS, FKN, and TNF-α 14 days after treatment (FKN was ↓ after 7-,14-days, and TNF-α was not affected 24h or 7 days after BoNT).<br>•IL-1β was significantly ↓ just 24 h after the BoNT intra-TMJ treatment, this effect was reversed 7 days after treatment.

	G2. immunised (TMJ arthritis)<br>(7-days after last immunisation, treated with unilateral intra-TMJ injection/ ipsilateral to immunisation):<br>G3.immunised + 3 different BoNT(7U/kg) groups (according to follow-up timing)<br>G4. Immunised + vehicle saline (0.9% NaCl; 20 µl)		(by enzyme-linked immunosorbent assay ELISA)	•intra-TMJ injection of BoNT may promote a central effect by reducing the P2X7/CatS/FKN microglia-activated pathway in the trigeminal subnucleus caudalis.<br>•This effect is related with a decrease of microglia modulators CatS and FKN, leading to the reduction of proinflammatory cytokines IL-1β and TNF-α. However, further experiments should be conducted in order to assess if these effects are accomplished in a direct or indirect manner.
	LIMITATIONS: Persistent antigen-induced arthritis in the TMJ, which uses (mBSA) as antigen, are useful to assess the immunomodulatory mechanisms of different drugs. However, there are other mechanisms involved in the pathogenesis of the model that may also be related to BoNT activity. This model is considered a delayed-type hypersensitivity model, with mBSA as the antigen. It is characterized by the recruitment of CD4+ T cells, which provide peripheral inflammatory mediators including diverse pro-inflammatory cytokines (e.g. TNF-α, IL-1β, IL-12, IL-18), and initiate sensitization of the CNS by activation of microglial cells. Recruitment and activation of resident microglia is in part mediated by the P2X7/CatS/FKN mechanism. The study was approved by the Ethics Committee in Animals Research of the State University of Campinas (CEUA/UNICAMP #4587- 1/2017), and followed the guidelines from the National Council for Control of Animal Experimentation (CONCEA), ARRIVES guidelines and the International Association for the Study of Pain			
Filipović, 2012 [28]	(n=200) male Wistar rats (300–350g) infraorbital nerve constriction (IoNC) (experimental model of trigeminal neuropathy)<br>2 types of trigeminal pain (neuropathic and inflammatory).<br>1. IoNC nerve injury - Bilateral mechanical allodynia (responsiveness to 0.16–2 g on both sides, 14 days after IoNC) - 70% of the IoNC operated animals.<br>2. Orofacial 2.5 % formalin-induced pain (injected unilaterally subcutaneously into vibrissal pad, 3-days prior testing in animals pretreated with saline/BoNT)<br><br>Follow-up: bilateral mechanical allodynia - 3, 6, 20 and 30-days following BoNT single unilateral injection (day 0);<br>Bilateral assessment of dural neurogenic inflammation plasma dural extravasation – 3-days after BoNT/17-days post-IoNC (without(1)/with(2) formalin)<br><br>1. (n=20/ 4 groups):	Trigeminal neuropathy induced by infraorbital nerve constriction (IoNC)	**Plasma protein complexes**<br>**Dural Protein Extravasation**<br>(ng of Evans blue per mg of dural tissue) - measured as colorimetric absorbance of Evans blue.<br><br>Other measurements: allodynia tested by von Frey filaments; to test the role of axonal transport of BoNT in sensory neurons for its effects on bilateral neuropathic pain and dural extravasation – axonal transport blocker Colchicine (2µl) injected into trigeminal ganglion; behavioural test - rubbing time.	•Results were presented as mean ± S.E.M and analyzed by ANOVA followed by the Newman-Keuls post hoc test for between-group differences. In the time-course experiment ANOVA was employed for repeated measurements followed by Tukey's test.<br>•Unilateral injections in 1. and 2. produced bilateral dural extravasation.<br>•Single unilateral BoNT injection (G1a or G1b) significantly ↓ dural extravasation (IoNC-induced), as well as allodynia (2 and 3 days following the BoNT and lasting more than 2 weeks), bilaterally.<br>•BoNT ↓ pain and dural extravasation (formalin-induced).<br>•Effects of BoNT on pain and dural extravasation in IoNC model were dependent on axonal transport through sensory neurons, as evidenced by colchicine injections (5 mM, 2 µl) into the trigeminal ganglion completely preventing BoNT effects.<br>•The lasting effect of a unilateral injection of BoNT in experimental animals suggests BoNT might have a long-term beneficial effect in craniofacial pain associated with dural neurogenic inflammation. Bilateral effects of BoNT and dependence on retrograde axonal transport suggest a central site of its action.

	G1. BoNT (3.5U/Kg) single unilateral injection 14 days after IoNC, subcutaneously into vibrissal pad (G1a.ipsilateral or G3b.contralateral).<br>G2. Saline (control)<br>G3. Sham operated - exposing infraorbital nerve without placing silk ligatures around the nerve.<br><br>2. (n=8/ 3groups):<br>G4. BoNT (G1) + formalin<br>G5. Saline (G2) + formalin<br>G6. Saline (control – G2)<br>To assess bilateral dural extravasation, dural tissue from 4 animals was pooled in one sample (left and right side separately)			
	LIMITATIONS: Doses based on the doses used in other pain models. The experiments were conducted according to the National Institute of Health Guide for the Care and Use of Laboratory Animals (Publication No. 85-23, revised 1996) and approved by the Ethical Committee of the University of Zagreb, School of Medicine. Infraorbital nerve constriction injury (IoNC) accompanied by hyperalgesia and allodynia is used as a model of trigeminal neuropathy in rats.			
Kitamura, 2009 [29]<br><br>*also in vitro study	(n=?) adult male Sprague–Dawley rats (200–250g)<br>unilateral infraorbital nerve constriction (IoNC) TN model.<br>Follow-up: post-operative (IoNC induction) day-14/ 11-days after BoNT/saline injection.<br><br>(day-3 after IoNC) single peripheral injection of BoNT (100pg in 0.1ml of sterile saline) in the center of the whisker pad.<br><br>G1.All rats were injected intradermally with saline on the contralateral side (sham).<br>On the ipsilateral side facial skin:<br>G2. IoNC + saline<br>G3. IoNC + BoNT	Trigeminal neuropathy induced by infraorbital nerve constriction (IoNC)	**Trigeminal ganglion (TRG) sensory neurons - IB4 (+) and IB4 (-) neurons** - acutely isolated from the side of the injury<br><br>By membrane-uptake marker (N-(3-triethylammoniumpropyl)-4-(6-(4-(diethylamino)phenyl)hexatrienyl) pyridinium dibromide (FM4-64) (by confocal microscopy fluorescence intensity ratio)<br><br>Other assessments: sensory testing, neuropathy behaviours (head withdrawal thresholds (HWTs)), dissociation of TRG neurons, FM4-64 dye staining, confocal imaging,	•Results are presented as group means ± SEM. Differences in group means were evaluated by RM ANOVA on ranks or t-test.<br>•(in vitro) BoNT pre-treatment (10 ng/60 µl) for 3h of acutely dissociated TRG neurons from naïve rats significantly ↓ the rate of FM4-64 release (induced by a depolarizing stimulus - KCl), from somata, compared to untreated neurons. This effect of BoNT was significantly higher ($P<0.05$, RM ANOVA on ranks) in IB4 (-) neurons compared to IB4 (+) neurons.<br>•**unilateral IoNC in rats produces long-lasting behaviours of neuropathy which are concomitant with ↑ transmitter release from somata of TRG neurons acutely isolated from the side of the injury** - IoNC speeds up the onset and rate of FM4-64 dye release from somata of TRG neurons:<br>IB4 (+) and IB4 (-) neurons ipsilateral to IoNC exhibited a profoundly faster onset of KCl-evoked vesicular release of FM4-64 compared to neurons isolated from contralateral TRG. The decay time constant (r) of FM4-64 signal during KCl application was 26.4±8.1s for IB4 (+) and 11.1±2.6s for IB4 (-) TRG neurons acutely dissociated from contralateral sham surgery. IoNC significantly ↓ the decay (r) in IB4 (+) (3.1±1.0 s) and IB4 (-) (4.2±0.9 s) neurons, without affecting the onset time of KCl-induced vesicular release. In addition, the initial rate of KCl-induced FM4-64 release was faster in IB4 (+) neurons ipsilateral to IoNC, while IB4 (-) neurons ipsilateral to IoNC showed significantly larger maximal release of FM4-64 compared to neurons from control rats. Intensity ratio: IB4(+) Ipsi IoNC (n=8), Contra-sham (n=11); IB4(-) Ipsi IoNC (n=8), Contra-sham (n=10).

				•Intradermal unilateral ipsilateral injection of BoNT (in the area of infraorbital branch of the trigeminal nerve innervation) alleviated IoNC- induced neuropathy behaviours (significantly ↑ the head withdrawal thresholds (HWTs) compared to saline-injected IoNC rats). HWTs: contra-sham (G1) n=12, saline (G2) n=6, BoNT (G3) n=6.<br>•BoNT ↓ the rate and magnitude of exaggerated FM4-64 dye release from somata in TRG neurons from these rats after IoNC. The KCl-induced decrease in the FM4-64 signal was greatly attenuated in both IB4 (+) and IB4 (-) neurons from IoNC rats treated with peripheral BoNT injection. Intensity ratio: IB4(+) IoNC + BoNT (n=9), IoNC + saline (n=8); IB4(-) IoNC + BoNT (n=9), IoNC + saline (n=8).<br>•Also, the onset of KCl - induced FM4-64 release was significantly slower in both IB4 (+) and IB4 (-) TRG neurons from the BoNT-treated IoNC side.<br>•(in vivo) peripheral injection BoNT in neuropathic rats ↓ neuropathic pain behaviors by ↓ exaggerated neurotransmitter vesicular release from TRG sensory neurons (in both IB4 (+) and IB4 (−) neurons)
	LIMITATIONS: study were performed in accordance to specifications of an animal protocol approved by Okayama University (OKU-2007137). All experiments were conformed to relevant National Institutes of Health guidelines on the ethical use of animals. This study was supported by a grant from the Ministry of Education, Science and Culture of Japan (No. 18390512), Ryobi Teien Memorial Foundation and Japanese Association for Dental Science. The study did not study the spread of BoNT/A in the brainstem, hence, it could not confirm the demonstrated ability of retrogradely transported BoNT/A to undergo transcytosis.			
Lacković, 2016 [20]	(n=105) male Wistar rats (300–350g; 3–3.5 months old) model of pain and inflammation induced by CFA injection into left TMJ.<br>*Methylene blue was injected into a few animals to confirm successful targeting of the TMJ.<br>Randomly allocated to different experimental treatments<br>Follow-up: 24h/1-day after CFA/4-days after BoNT (G2) or 2h after G3* (behavioural assessment*, DNI plasma extravasation*, and RIA for CGRP), ? (histology and immunohistochemistry of dura mater)<br><br>G0. Saline control (sham)<br>G1. CFA + Saline (0.9% NaCl)<br>G2. CFA + BoNT single injections into left TMJ (3-days prior CFA):<br>G2a. Intra-articular (5Ukg^{-1},20 µL)	Trigeminal pain - model temporomandibular disorders (inflammatory pain) induced by complete Freund's adjuvant injection into temporomandibular joint (TMJ)	**Cranial dura tissue, brainstem (TNC)**<br>**Dural neurogenic inflammation (DNI) marker**-plasma protein extravasation (ng (mg tissue)$^{-1}$) (assessed by Evans blue dye, ipsilateral and contralateral)<br>**Dura mater, ipsilateral TNC, ipsilateral Trigeminal ganglion (TG), CSF**<br>**Neuropeptide** – CGRP (fmol mg^{-1} or fmol mL^{-1}) (by RIA)<br>**Cranial dura enzymatic activity** – SNAP-25 (assessed by immunohistochemistry)<br>**Inflammatory cells** – lymphocyte, monocyte, neutrophile, plasma cells (by Giemsa-staining - number of Giemsa positive profiles)	•CFA-evoked TMJ inflammation was accompanied by inflammatory changes in the cranial dura (plasma protein extravasation significantly ↑ bilaterally in CFA-injected vs. G0, in ipsilateral dura was double that contralateral side ($P < 0.001$, t-test for dependent samples) and elevated number of inflammatory cell infiltration, which were not present in G0, ($P < 0,001$)) and significantly ↑ levels of CGRP I dura mater and ipsilateral caudal trigeminal nuclei.<br>•Following peripheral toxin injection, cleaved SNAP-25, the product of BoNT enzymic activity, was colocalized with CGRP-expressing dural afferents.<br>•BoNT prevented CFA-evoked dural inflammation and CGRP peptide increase in cranial dura.<br>•Results are presented as means ± SEM and analysed by one-way ANOVA followed by the Newman–Keuls post hoc test. $P < 0.05$ was considered significant<br>•G2a, G2b, and G3 ↓ **bilateral mechanical allodynia evoked by CFA** (P<0.001). The differences between the BoNT (G2) and sumatriptan (G3) were not significant. (n/group = 5–9), *P < 0.05, ** P < 0.01, ***P < 0.001, significantly different from G0; +++ P < 0.001, significantly different from G1.<br>•G2a, G2b, and G3 ↓ **DNI evoked by CFA** (P<0.001). In the contralateral side, none of the Tx affected the DNI. (n/group = 5–9). *P < 0.05, ***P < 0.001, significantly different from G0; ++P < 0.01; +++P < 0.001, significantly different from G1.<br>•G2c - four injections outside TMJ, as observed with the single BoNT injection into the TMJ (G2a and G2b), prevented both bilateral allodynia and the CFA-evoked plasma protein extravasation (n/group = 5–8), *P < 0.05, **P < 0.01, ***P < 0.001, significantly different from G0; + P < 0.05, significantly different from G1; +++P < 0.001, significantly different from G1.

	G2b. Intraganglionic – left trigeminal ganglion through infraorbital foramen ($2U kg^{-1}$,2µL volume)<br>G2c. Facial injections at four sites outside TMJ - total dose of $5U kg^{-1}$ divided in four equal doses ($1.25 U kg^{-1}$ per site):<br>(i) bilaterally into the rat forehead above the orbital arch,<br>(ii) bilaterally into the whisker pad.<br>G3. (n=?) CFA + sumatriptan ($175µgkg^{-1}$) 24h after CFA		Other assessments: behavioural testing (mechanical allodynia) by using von Frey monofilaments, Investigation of the effect of the axonal transport inhibitor, colchicine (7-days after G2a and G2b), on antinociceptive activity and appearance of cleaved SNAP-25 in dura mater following BoNT injection.	•BoNT prevented **dural tissue infiltration with inflammatory cells induced by TMJ inflammation.** (n/group = 5. *P < 0.05, significantly different from saline control; ***P < 0.001, significantly different from G0; +++P < 0.001, significantly different from G1.<br>•BoNT inhibited the upregulation of **CGRP levels in dura induced by TMJ inflammation**. The effect of BoNT on CGRP expression in trigeminal nuclei was not significant. CGRP concentration was not significantly altered in trigeminal ganglion and CSF. (n/group = 6. *P < 0.05, significantly different from G0; **P < 0.01, significantly different from G0; ++P < 0.01, significantly different from G1).<br>•After BoNT peripheral application (G2a, G2b, G2c), **cleaved SNAP-25 colocalized with CGRP** was present in the injected-side of intracranial dural nerve endings. Contralateral dura was devoid of cleaved SNAP-25, ruling out possible systemic BoNT diffusion. (n/group = 6. *P < 0.05, significantly different from saline control; **P < 0.01, significantly different from saline control; ++P < 0.01, significantly different from saline + CFA).<br>•Injection of the axonal transport blocker colchicine into the trigeminal ganglion prevented the formation of cleaved SNAP-25 in dura.<br>•Pericranially injected BoNT was taken up by local sensory nerve endings, axonally transported to the trigeminal ganglion and transcytosed to dural afferents. Colocalization of cleaved SNAP-25 and the migraine mediator CGRP in dura suggests that BoNT may prevent DNI by suppressing transmission by CGRP. This might explain the effects of BoNT in TMJ inflammation and in migraine and some other headaches.<br>•BoNT injections at four sites were effective in preventing CFA-evoked pain and DNI similarly to the single BoNT injection into the TMJ, suggesting that the effects of BoNT on allodynia and DNI are not primarily mediated by its direct peripheral effect on CFA-stimulated neurons.
	LIMITATIONS: Plasma protein extravasation in cranial dura is a useful marker of trigeminal activation, often employed in preclinical screening of antimigraine drugs. It was only hypothesized that TMJ pain might provide a suitable model to study trigeminal activation leading to DNI, as well as the mechanism of BoNT action in the trigeminovascular system, assumed to be involved in migraine and other headaches. All animal care and experimental procedures were in accordance with the 2010/63/EU Directive on the protection of animals used for scientific purposes and the recommendations of International Association for the Study of Pain and were approved by the Ethical Committee of University of Zagreb School of Medicine. The experimental procedures used in the work described in this article were as humane as possible. All animal studies are described in compliance with the ARRIVE guidelines for reporting experiments. Five animals per group were examined for histological study of the cranial dural tissue. Some results for Cleaved SNAP-25 colocalizes with CGRP-expressing afferents of the dura mater after BoNT/A peripheral treatment are not shown. The work was supported by grants from Croatian Ministry of Science, Education and Sport (no. 108-1080003-0001 awarded to Z. L.), Croatian National Science Foundation (no. O-1259- 2015 awarded to Z. L.), Hungarian National Brain Research Program (SROP4.2.2.A-11/1/KONV-2012-0024 and the KTIA NAP_13-1-2013-0001 awarded to Z. H.) and National Brain Research Program B (Chronic Pain Research Group); KTIA_NAP_13–2014-0022; awarded to Z. L., 888819).			
Wang, 2020 [25]	(n=18) healthy, mature New Zealand big-ear albino rabbits (no sex restriction) 2.5~3.5 kg.<br>*6 wounds on each ear, at least 1 cm apart, 18 rabbits with a total of 216 wound.	Hypertrophic scar	**Scar tissue (ear)**<br>Apoptosis rates of fibroblasts (%).<br>Collagen I, III, and TGF-β1 expression (by Western blot analysis)<br>Expression of α-SMA and myosin II proteins (by Immunohistochemistry)	•Groups comparison using Student's t-test. The data are expressed as mean ± S.D. $P \leq 0.05$ is considered as statistically significant.<br>•The **thickness of dermis** at scar site (G3) 2.43 ± 0.47mm, 1.78 ± 0.37mm in G1 (0.5 IU), 0.78 ± 0.51mm in G1 (2.0 IU), 0.64 ± 0.49mm in G2, and 0.34 ± 0.17mm in normal skin (G4). BoNT could significantly ↓ the thickness of dermis in scar area of rabbit ear ($P < 0.01$) with dose dependent, which had similar effect with hormone.<br>•BoNT ↓ **cell proliferation in the dermis**, which was related to the effects of BoNT on the expression of collagen. The **apoptosis rates of fibroblasts** were ↑ in the scars with the Tx of

	Follow-up: 60 days after injection (scar tissue harvested), day-28 after the injection of BoNT (apoptotic effect)<br><br>At the day 28, when the scar has appeared, the Tx regimen started.<br>36 rabbits ears were randomly divided into 4 detection groups:<br>(Tx groups, directly inject the drug into the lesion/scar)<br>G1. (n=12, right ear) treated with BoNT (0.5U, 1.0U, 1.5U, 2.0U)<br>G2. (n=12, left ear) same dose of hormone-TAC<br>G3. (n=12) scar group + phosphate-buffered saline (PBS) - no Tx<br>G4. Healthy skin (control) – no Tx		Other assessments: Thickness of dermis (mm)	BoNT than PBS and the rates in normal skin were much ↓ ($P < 0.01$). BoNT ↑ the apoptosis rates of fibroblasts with dose dependent.<br>•**collagen type I and III** - expression levels ↓ significantly in G1 and G2 ($P < 0.01$), while a large number of deposition was revealed in the PBS control group (G3). There was significant difference on the expression of collagen I between G2 and G1 (2.0 IU) ($P < 0.05$). On the contrary, there was no significant difference in the expression of collagen III between G2 and medium, high-dose BoNT (1.5 IU, 2.0 IU). In addition, the inhibition of collagen I was increasingly obvious with the dose of BoNT increased ($P < 0.05$).<br>•high-dose BoNT (2.0 IU) and G2 could significantly ↓ the **expression of TGF-β1** compared with the PBS group. However, there were no significant changes between BoNT and G2. In addition, there were no significant differences between high-dose and low-dose of BoNT.<br>•expression of **α-SMA and myosin II proteins** were significantly ↓ in G1 and G2 compared with G4. With the dose of BoNT increasing, the expression of proteins ↓. Moreover, the changes were significant between low-dose and high-dose BoNT on the expression of myosin II proteins ($P < 0.05$). However, there were no significant changes with high concentrations of BoNT (1.5, 2.0 IU) and G2.
	LIMITATIONS: All animal experiments were conducted according to Principles of Laboratory Animal Care (National Society for Medical Research). This study was approved by the Animal Ethics Committee of Anhui Medical University (LLSC20170427). This study was funded by Anhui Science and Technology Research Project (1604a0802078). Study was performed based on the limited scar tissue of rabbits. The results may not represent the scar in human. there is no animal weight comparison in this study, which may interfere the formation of hypertrophic scar tissue during 4 weeks' feeding. The specific regulation mechanism of BoNT on the above protein has not been clear, the action time of drug is short and concentration point selection is not enough, the suitable concentration range for BoNT has not been clearly studied.			
Cho, 2022 [17]	(n=236) male Sprague-Dawley rats (250−280 g) - rodent model of TN produced by compression of the trigeminal nerve root (TNR)<br><br>Follow-up:<br>Sampling from TG –7-days after compression of TNR/ 2 days after single BoNT injection (postoperative day-7).<br>Mechanical allodynia – 2-days before TNR, day- 3, 5, 6, 7, 10, 12, 14, 17, 21, 24, 30, 40, 55, and 70 after TNR. (effects of single or double/repeated Tx BoNT (1 or 3 U/kg) at postoperative days 5 and 12, respectively).<br>G0. Model of TN<br>G1. TN + Single subcutaneous injection BoNT (3 U/kg) postoperative day-5.	Trigeminal neuralgia – induced by compression of the trigeminal nerve root (TNR)	**Trigeminal ganglion (TG) transcriptional regulator of hypoxia-induced cellular responses**: hypoxia-inducible factor (HIF-1α) protein expression (GAPDH was used as a loading control)<br>(by western blotting)<br>**Cytokine levels**: IL-1β, IL-6, and TNF-α concentrations (pg/ml)<br>(by ELISA)<br><br>Other assessments: Evaluation of Mechanical Allodynia (changes in the air-puff thresholds) and up-regulate IL-1β, IL-6, and TNF-α concentrations in the TG after injection of PX-12, a HIF-1α	•Data were reported using mean ± SEM. Data obtained from the western blots and ELISA procedure were analyzed by using Student's t-test for 2 group comparisons, and one way ANOVA subsequently using the Holm-Sidak post hoc analysis for multi-group comparisons:<br>• Postoperative day-7 – Model (G0) in comparison to sham group(G2) or naïve rats(G3):<br>- produced a significant mechanical allodynia (significant ↓ of ipsilateral air-puff thresholds ($F [1, 19] = 269.56$, $P < .05$), observed on POD 3, maintained until POD 55, and returned to its preoperative values on POD 70.<br>- ↑ the expression of (HIF)-1α and cytokines levels including interleukin (IL)-1β, IL-6, and (TNF)-α in the trigeminal ganglion (TG) (significantly ↑ IL-1β, IL-6, TNF-α levels, and HIF-1a expression in the ipsilateral TG compared with G2/sham ($P < .05$), but did not affect HIF-1α expression in the contralateral (data not shown).<br>• Single or double Tx with a high BoNT (3 U/kg) led to significantly prolonged antinociceptive effects.<br>• Single BoNT Tx (3 U/kg) significantly ↑ the air-puff thresholds compared with the vehicle-treated G4 ($F [2, 18] = 51.60$, $P < .05$); double BoNT Tx (3 U/kg), also significantly ↑ the air-puff thresholds in comparison to G4 ($F [2, 18] = 276.85$; $P < .05$).

	G2. Sham group<br>G3. Naïve rats<br>G4. TN + vehicle Tx<br>(n = 6/group) to measure cytokine concentration; (n = 6/group) for western blot analysis.<br>All animal groups underwent a single-blind randomization carried out by one of the researchers.		inhibitor (to elucidate the participation of the HIF-1α associated cytokine pathway in the development of TN)	• 1st BoNT Tx on POD-5 produced anti-allodynic effects similar to the single treatment, the 2nd Tx on POD-12 produced anti-allodynic effects for 9 subsequent days. Repeated subcutaneous injections of a low dose BoNT (1 U/kg) did not affect the air-puff thresholds.<br>• day-2 after single Tx with BoNT (3 U/kg) significantly suppressed the upregulation of HIF-1α expression and IL-1β, IL-6, and TNF-α concentrations in the TG. $P<0.05$, BoNT-treated G1 compared with vehicle-treated G4.<br>• Intraganglionic injection of PX-12, compared to vehicle (100% DMSO), led to significant anti-allodynic effects and ↓ the IL-1β, IL-6, and TNF-α levels in the (TG $F[1, 12] = 251.8$; $P < .05$).<br>•These findings indicate that:<br>- the antinociceptive effect of BoNT is mediated by HIF-1α associated cytokines modulation in the TG and it is therefore a potentially relevant Tx strategy for TN. Results suggest that repeat BoNT Tx will effectively inhibit and that the BoNT-induced antinociceptive effects are transmitted through the primary afferent fibers and not through a systemic mode of action because anti-allodynic effects were not produced following a subcutaneous BoNT injection into the left hind leg (data not shown).<br>- participation of HIF-1α in the development of TN and that upregulated HIF-1α expression in the TG plays a critical role in the development of pain.
	LIMITATIONS: This model described a significant long-term nociceptive pain behaviors in this animal model, including mechanical allodynia and hyperalgesia caused by compression of the TNR. Generally, hypoxia, ischemia, and inflammatory conditions increase the HIF-1α level, which produces proinflammatory cytokines such as interleukin (IL)-1b or (TNF)-α that will maintain the inflammatory processes. A recent study has shown the involvement of HIF-1α in chronic pain. In that report, a HIF-1α blockade produced analgesic effects in a complex regional pain syndrome animal model. However, no evidence is currently available regarding a possible role of HIF-1a in the development of TN. The role of cytokines in the development of neuropathic pain is well known. All procedures and animal experiments followed experimental protocols (pain grade E) approved by the Institutional Animal Care and Use Committee of the School of Dentistry, Kyungpook National University. All evaluations with animal were carried out in accordance with the ethical principles recommended by the International Association for the Study of Pain (IASP). In this study, only male SD rats were utilized to exclude the effects of sex hormones on nociceptive thresholds because pain threshold and tolerance have been found to be substantially smaller in female than male. Sex hormones play a key role in contributing to gender differences.			
Li, 2023 [18]<br><br>*also in vitro	(n=) Parkinson disease adult mouse model (30g, 6–8 weeks old) male ICR mice chronically administered reserpine (mice were fed a reserpine solution (prepared with drinking water, 3 μg/mL) daily for 10 weeks) – behavioural phenotypes of depression and neurochemical changes in the substantia nigra pars compacta (SNpc) and striatum.<br><br>Follow-up:?<br><br>From the 10th week, BoNT (10U/kg−1 ·d−1) injected into the cheek once daily for 3 consecutive days.	Depression (induced by reserpine chronic administration) in Parkinson´s disease established model.	**Brain-Substantia nigra pars compacta (SNpc) and hippocampus**<br>Dopamine levels (ng/g), tyrosine hydroxylase (TH)<br>(by Western blotting and immunohistochemistry)<br>**Proinflammatory cytokine** (TNF-α and IL-1β) protein and mRNA levels (by western blotting and RT-PCR, respectively).<br>**Complement expression and microglia activation –** C3, C1q, and complement receptor C3aR -	• All data are presented as the mean ± SEM. Two-way ANOVA followed by Bonferroni's multiple comparison test and one-way ANOVA with Tukey's post hoc test were used. $*P < 0.05$, $**P < 0.01$, $***P < 0.001$, versus Con group. $\# P < 0.05$, $\#\#P < 0.01$, $\#\#\#P < 0.001$.).<br>•(G1) reserpine model (compared with control G2) - dopamine and TH level was significantly ↓ in both the substantia nigra and striatum, ↑ complement C3 and C1q protein expression in the hippocampus, significantly ↑ mRNA expression of complement receptor C3aR, significantly ↑ TNF-α and IL-1β protein and mRNA, mRNA expression of CX3CR1 was significantly ↑ in the RSP group. However, no remarkable changes were observed in the mRNA expression of CX3CL1 among the different groups. excitatory VGlut2 synaptic density was ↓ in the reserpine-induced PD depression model, while BoNT could reverse glutamatergic synapse loss in the hippocampal regions.<br>•BoNT significantly ameliorated **depressive-like behaviours,** and although it reversed the reserpine-induced decrease in **TH protein levels**, it did not improve significantly TH activity in SNpc of reserpine-treated mice. (Immunoblotting analysis of the expression of TH in the SNpc: Quantification of TH protein expression normalized to GAPDH in reserpine-treated mice with

	G1. Reserpine-treated mice<br>G2. Control mice<br>G3. Reserpine-treated mice + BoNT<br>G4. Control mice + BoNT<br><br>Mice received intraperitoneal injections of pramipexole and duloxetine (10 mg/kg) for two consecutive weeks starting at the 10th week of reserpine treatment - effect of BoNT on reserpine-induced depressive symptoms in PD mice (behavioural tests) was compared with that of other common medications, including pramipexole and duloxetine.		signalling receptor expressed by microglia (protein and mRNA expressions)<br><br>**Microglial activation and engulfment of synapses -** Immunostaining of the presynaptic marker VGlut2 (excitatory presynaptic vesicle proteins), lysosomal marker CD68, and microglial marker* in Ionized calcium binding adaptor molecule 1 (Iba1).<br>**mRNA expression of CX3CR1 signalling, fractalkine CX3CL1, synapse and spine loss-** colocalized puncta of VGlut2 and PSD95 and dendritic spines from CA1 pyramidal neurons, complement C3 and C1q protein expression,<br><br>*Microglia are the resident immune cells in the central nervous system.<br><br>Other assessments: Behavioural test (body weight, Rotarod test, Pole climbing test, Open field test, Forced swimming test, Tail suspension test, Sucrose preference test), In vitro cell culture-CCK-8 assay (mouse BV2 microglial cell line supplemented with reserpine in the presence or absence of BoNT for 24h)	BoNT Tx (G1) (n = 3 mice for each group, F (3, 8) = 4.316, P = 0.0436). Quantification of TH-positive neurons relative to the Control (G2) in the SNpc (n = 3 mice for each group, F (3, 8) = 3.767, P = 0.0593).<br>•Substantia nigra and striatal dopamine concentrations were detected by HPLC (for substantia nigra dopamine, t = 3.005, df = 6, P = 0.0239. For striatal dopamine, t = 2.884, df = 6, P = 0.0279). Striatal dopamine concentrations were also determined by ELISA (t = 3.614, df = 6, P = 0.0112).<br>•BoNT ameliorated the reserpine-induced activation of the **classical complement pathway** in vivo and in vitro. ((n = 6 images from 3 mice for each group. For C3, F (3, 20) = 5.604, P = 0.0059).<br>•BoNT reversed reserpine-induced **complement and microglia activation in the hippocampal CA1 region:** significantly ↓ protein expression of complement C3 and C1q, the mRNA expression: of complement C3 was found to have a consistent change compared to that of protein alterations, but no significant difference was found in the complement C1q among the different groups. mRNA expression of C3aR was significantly ↓ in G3 (Quantification of complement C3 and C1q protein expression normalized to Tubulin (n = 3 mice for each group, For C3, F (3, 8) = 34.09, P < 0.0001. For C1q, F (3, 8) = 29.37, P = 0.0001). Complement C3, C1q, and C3aR mRNA expression levels were determined by qRT–PCR (n = 5–6 mice for each group, For C3, F (3, 20) = 8.045, P = 0.0010. For C1q, F (3, 20) = 1.978, P = 0.1498. For C3aR, F (3, 16) = 4.338, P = 0.0204).<br>•**BoNT suppressed microglial activation in vivo and in vitro.** Quantification of % of CD68+ lysosome volume in Iba1+ microglia volume (n = 6 images from 3 mice/each group. F (3, 20) = 27.82, P < 0.0001). Chemokine receptor CX3CR1 (e) and fractalkine CX3CL1 (f) mRNA expression levels in the hippocampal samples were measured by qRT–PCR (n = 5–6 mice for each group. For CX3CR1, F (3, 20) = 5.718, P = 0.0054). For CX3CL1, F (3, 16) = 2.236, (P = 0.1235).<br>•BoNT significantly attenuated the **microglial engulfment of presynaptic synapses, thus ameliorating the apparent synapse and spine loss in the hippocampus** in the reserpine-treated mice. (Quantification of individual and colocalized excitatory pre- and postsynaptic markers (c) and inhibitory pre- and postsynaptic markers (d) (n = 6 images from 3 mice for each group. For VGlut2, F (3, 20) = 7.739, P = 0.0013. For PSD95, F (3, 20) = 7.603, P = 0.0014. For VGlut2 + PSD95, F (3, 20) = 9.593, P = 0.0004. For VGAT, F (3, 20) = 1.191, P = 0.3384. For Gephyrin, F (3, 20) = 0.2680, P = 0.8476. For VGAT + Gephyrin, F (3, 20) = 2.016, P = 0.1440). e Representative images of Golgi-stained dendrite spines of pyramidal neurons in the hippocampal CA1 regions. Scale bar = 5 μm. f Quantification of the dendrite spine density of pyramidal neurons in the hippocampal CA1 regions (n = 12 images from 3 mice for each group. F (3, 44) = 27.26, P < 0.0001).<br>(Quantification of the percentage of engulfed VGlut2+ synaptic volume in Iba1+ microglial volume (n = 6 images from 3 mice for each group. F (3, 20) = 25.95, P < 0.0001).

				•BoNT suppressed **microglia-mediated expression of pro-inflammatory cytokines** TNF-α and IL-1β in reserpine-treated mice. (BoNT ameliorated microglia-mediated neuroinflammation in vivo and in vitro. a, b Hippocampal TNF-α (a) and IL-1β (b) protein concentrations were measured by ELISA (n = 3 mice for each group. For TNF-α, F (3, 8) = 23.78, P = 0.0002. For IL-1β, F (3, 8) = 14.50, P = 0.0013). c, d Hippocampal TNF-α (c) and IL-1β (d) mRNA expression was determined by qRT–PCR (n = 5 mice for each group. For TNF-α, F (3, 16) = 11.79, P = 0.0003. For IL-1β, F (3, 16) = 7.872, P = 0.0019).<br>•(IN VITRO) BoNT (0.1 U/mL) ameliorated reserpine-induced complement and microglia activation in mouse BV2 microglial cells in vitro.<br>•BoNT may ameliorate depressive-like behaviour in a reserpine-induced PD mouse model through reversing the synapse loss mediated by classical complement induced-microglial engulfment as well as alleviating microglia-mediated proinflammatory responses.<br>•BoNT ameliorates reserpine-induced depressive behaviours.<br>•Complement activation is involved in the reserpine-induced mouse model.<br>•BoNT reverses spine loss and reduced synapse density, inhibits complement activation and microglial engulfment, alleviates reserpine-induced neuroinflammation.
	LIMITATIONS: The reserpine-induced model has been widely applied to investigate the role of the monoamine system in the regulation of motor and psychiatric disorders, including PD and depression. All animal experiments were conducted according to the National Institutes of Health Laboratory Animal Care and Use Guidelines. All animal operations and experimental procedures were approved by the Animal Ethics Committee of Soochow University. This work was supported by grants from the National Natural Science Foundation of China (32200778), Natural Science Foundation of Jiangsu Province (BK20220494), Suzhou Medical and Health Technology Innovation Project (SKY2022107), startup funding [NH21500221], [NH21500122], and research funding by the Clinical Research Center of Neurological Disease in The Second Affiliated Hospital of Soochow University [ND2022A04] to Qifei Cong; the Suzhou Science and Technology Plan Key Technology Application Research Project [SS2019060], the National Natural Science Foundation of China [No. 81671270], the Suzhou Clinical Research Center of Neurological Disease [SZZX201503], and the Natural Science Foundation of Jiangsu Province of China [BK2011294] to Wei-feng Luo; and the Priority Academic Program Development of Jiangsu Higher Education Institutes (PAPD) to Chun-feng Liu.			
Yang, 2016 [31]	(n=) male Sprague-Dawley rat (220 and 240g) model of trigeminal neuropathic pain produced by malpositioned dental implants following extraction of 2nd molar- inducing injury to the inferior alveolar nerve VS Sham Group (had only extraction without implant placement).<br><br>Follow-up: 3, 7, 12 days after surgery (mechanical allodynia); 3days before surgery and 3, 5, 7, 9, 11, 14, 18, 21, 25, 32, 39, 46, and 53-days after (behavioural changes); POD9/6-days after BoNT (3U/Kg) (expression ATF3, Nav 1.3, 1.6, 1.7, 1.8)<br>*In some animals, antinociceptive effects were evaluated after repeated low doses	Trigeminal neuropathic pain produced by malpositioned dental implants	**nerve-injured trigeminal ganglion (mandibular (V3) division and boundary area) ipsilateral**<br>voltage-gated sodium channels (Navs) - Nav isoform 1.3, 1.6, 1.7, 1.8 expression;<br>**marker of neuronal injury** - expression of ATF3<br><br>Other assessments: Mechanical allodynia, air-puff threshold, aggressiveness, and biting	•The Western blotting data were analyzed by 1-way analysis of variance, followed by Holm-Sidak post hoc analysis. Results are mean ± SEM at each time point tested.<br>•Subcutaneous injections BoNT (1 or 3 U/kg) on POD 3 significantly attenuated mechanical allodynia (F3,28 = 28.390, P < 0.001) , although 0.3 U/kg of BoNT did not affect the air-puff threshold compared to G2.<br>•A single injection with 1U/kg of BoNT, the antiallodynic effects persisted up to POD 18, while BoNT (3 U/kg) produced prolonged antiallodynic effects over the entire experimental period.<br>•Tx with BoNT on postoperative days 7 and 12, when pain had already been established, also produced prolonged antiallodynic effects (F3,28 =26.434, P < 0.001).<br>•Double Tx BoNT (1U/kg, but not 0.3U/Kg) produced prolonged, more antiallodynic effects as compared with single treatments (F1,14 = 39.373, P < 0.001).<br><br>•The malpositioned dental implant produced significant ↑ the expression of Nav1.6, Nav1.7, and Nav1.8, but nerve injury did not affect the expression of Nav1.3 in the trigeminal ganglion.<br>•Subcutaneous administration of BoNT (3 U/kg) significantly inhibited the upregulation of only Nav isoform 1.7 (Nav1.7) expression in the mandibular (V3) division (boundary area) of the trigeminal ganglion in the nerve-injured animals (F2,12 = 9.176, P < 0.05).

	of BoNT (0.3 or 1 U/kg) on POD 3 and 4, respectively<br><br>Day-3 pot-operative, (n = 5 per group) subcutaneous injection into the most sensitive area of facial region:<br>G1. BoNT (G1a. 0.3U/Kg, G1b. 1U/Kg, G1c. 3U/kg).<br>G2. Saline- treated			•The malpositioned dental implant ↑ the level of ATF3-positive cells in the trigeminal ganglion on POD 9, but this was not affected by the subcutaneous injection of 3 U/kg of BoNT – suggesting that BoNT did not alleviate injury of the inferior alveolar nerve produced by a malpositioned dental implant
	LIMITATIONS: Navs play a critical role in modulating the excitability of most neurons, including nociceptive sensory signalling. All procedures involving the use of animals were approved by the Institutional Care and Use Committee of the School of Dentistry, Kyungpook National University, and were carried out in accordance with the ethical guidelines of the International Association for the Study of Pain for the investigation of experimental pain in conscious animals. s supported by the National Research Foundation of Korea (funded by the Ministry of Science, ICT and Future Planning; 2008-0062282 and 2012M3A9B6055414) and by Hugel, Inc.			
Ni, 2023 [24]	(n=) pathogen-free C57BL/6J male mice (8 weeks of age, 25g) subjected to chronic restraint stress (CRS) - induced depression and drug administration<br><br>CRS - placed into 50-ml conical tubes with holes for air fow 2–3h/ day for 21 consecutive days.<br><br>Follow-up: After the 21-day restraint stress terminated: day-22 (FST), day-23-27 (SPT) - behavioural tests. 24h after behavioral tests, mice were sacrificed for tissue section preparation.<br><br>Mice were randomly divided into three groups for later measurement at three different time points. Each group was then randomly divided into 5 subgroups for specific treatment.<br><br>pre-injecting into the unilateral whisker intrinsic musculature (WIM) with 3 dosages at 3 different time points prior to the end of the restraint period:<br>i) 6 weeks before to the restrain end/ 3weeks before the restrain start,	major depressive disorder (MDD)	**Brain**<br>**hindbrain sections**<br>1 - BoNT-cleaved SNAP25 (**cl.SNAP25197**) – to explore characteristics of retrotranssynaptic action and dose- or time-dependence of BoNT-mediated cleavage.<br>**subcellular spatial localization**<br>2- **SNAP25** was co-immunostained with choline acetyltransferase (**ChAT**, a marker of cholinergic neurons) and postsynaptic density-95 (**PSD95**, a dendrite marker representing the post-synaptic membrane) – to address whether BoNT utilized transsynaptic activity to reach the synaptic terminals of second-order projecting neurons.<br>5 - to evaluate whether the activity of wFMNs-projecting vlPAG neurons is altered following CRS or BoNT - **expression of c-Fos**.	• Pre-injection of high dose of 30 U/kg BoNT at three time points, while mice in the 10 U/kg subgroups pre-injected BoNT 3 weeks and 3 days, and 3U/kg subgroup pre-administered BoNT 3 weeks prior to the end of restraint robustly exhibited ameliorated depressive-like behavior in CRS mice.<br>• BoNT undergoes retrograde cell-to-cell transport from facial injection loci to the second-order neuron wFMNs-projecting synaptic boutons could directly modulate depression by inhibiting the excitatory neurons in the vlPAG with output to the wFMNs.<br>• BoNT-cl.SNAP25197 was detectable only in one side of the lFN, a subnucleus encompassing upstream synaptic terminals without interneurons, but not in the trigeminal sensory nuclear complex, adjacent regions, or premotor nucleus, parallels the concept of BoNT retrograde transcytosis via neural connection rather than systemic diffusion.<br>• the majority of c-Fos-positive neurons in the vlPAG of CRS mice inhibited by BoNT facial injection were CaMKII-positive, whereas the overlap between GABAergic and c-Fos-positive neurons was little affected, indicating that BoNT displays an affinity for excitatory neurons in the CNS and that BoNT influences depressive-like behavior, at least by initially acting on whisker motor neurons, followed by entering the premotor nucleus to influence its function.<br><br>Data were analysed using one-way ANOVA followed by Bonferroni' or Dunnett's multiple comparisons test to analyse the data from the subgroups. Two-tailed Student's t-test was used for two-group comparisons with normally distributed data. Results are presented as means±SEM<br>• **BoNT transsynaptic enters second-order projecting neuron boutons after axonal retrograde transports to wFMNs soma**:<br>**1** - BoNT axonal retrograde transported to the soma of (wFMNs) and subsequent transcytosis to synaptic terminals of second-order neurons induced central effects.<br>**1** - quantification of cl.SNAP25197 revealed that BoNT activity was dose-dependent, with a maximum positive signal observed in the 30 U/kg subgroup that decreased with attenuated BoNT dosages 10 days after the drug injection: quantificational analysis of BoNT-cleaved

	ii) 3weeks before to the end of restrain/ 1-day before restrain start,<br>iii) 3days before 21-day restraint stress end.<br><br>G1. CRS+BoNT (i) n=13; ii) n=15; iii) n=9) each group - G1a. 3U/kg, G1b. 10U/Kg, G1c. 30U/Kg)<br>G2. (i) n=13; ii) n=15; iii) n=9) CRS+sterile saline (5µl)<br>G3. (i) n=10; ii) n=10; iii) n=10) Naïve+sterile saline (5µl) (restricted to the food and water with free moving).		5 – **c-Fos** + excitatory (Ca2+/calmodulin-dependent protein kinase type II (**CaMKII)**), inhibitory (glutamate decarboxylase 1 (**GAD67**))<br><br>Other assessments:<br>**unilateral WIM under condition of trigeminal ganglion infraorbital nerve transection**<br>monosynaptic retrograde tracer CTB-488 injected alone or CTB-488-mixed BoNT to label the wFMNs (whisker-innervating facial motoneurons). Co-immunostaining for NeuN (a neuronal marker).<br>**innervating the lFN**<br>To identify the terminal types of aferent axons projecting to wFMNs, focus on three distinct types synapses expressing acetylcholine vesicular transporter (vAChT), serotonin transporter (SerT), or vesicular glutamate transporter 2 (vGluT2) - to identify the terminal types of afferent axons projecting to wFMNs. (Co-immunostaining of these specifc synaptic markers in brain sections from CTB-488-mixed BoNT/A-treated mice)<br><br>5.dopaminergic (tyrosine hydroxylase (TH)), and serotonergic (tryptophan hydroxylase 2 (TpH2)) neuronal markers - to identify the neuron types in the vlPAG that send afferent synapses to the wFMNs.	SNAP25197 among subgroups of 3 different BoNT dosages. n=4 cells from 3 mice/subgroup, respectively. $F (2, 9)=99.68$, $P<0.0001$. Bonferroni's multiple comparisons: 3 U/kg vs. 10 U/kg: $P<0.0001$, 3 U/kg vs. 30 U/kg: $P<0.0001$, 10 U/kg vs. 30 U/kg: $P=0.0169$.<br>**1** - cl.SNAP25197 was detected 10 days following BoNT injection and persisted in the lFN for at least 7 weeks following BoNT injection at 10 U/kg: quantificational analysis of the % of BoNT-cleaved SNAP25197-positive signal areas among groups of 3 time points post-BoNT (10 U/kg) pre-injection, indicating that cl.SNAP25197 was detected at 10 days, with a highest expression at 4 weeks, and existed persistently to at least 7 weeks post-BoNT WIM injection. n=4 brain sections from 3 mice in the group of 10 days, n=5 brain sections from 3 mice in the group of 4 weeks, and n=6 brain sections from 3 mice in the group of 7 weeks, respectively. $F (2, 12)=4.33$, $P=0.0384$. The P-value of Bonferroni's multiple comparisons: 10 Days vs. 4 Weeks: $P=0.8069$, 10 Days vs. 7 Weeks: $P=0.4491$, 4 Weeks vs. 7 Weeks: $P=0.0382$.<br>**2** - SNAP25 surrounded ChAT and PSD95 but did not co-localize with either ChAT or PSD95. SNAP25 was thus considered to specifically localize in the plasma membrane of the axons and was acted upon by BoNT at this site, as shown through transsynaptic tracing from the cholinergic somas of wFMNs (Scale bar, 50 µm. *P<0.05, ***<0.0001,n.s non-significance).<br>**3** - After 10 days, CTB-488 signals were detected only in the ipsilateral lFN of the injection side, but not in the trigeminal sensory nuclear complex. Co-immunostaining for NeuN (a neuronal marker) together with CTB-488 and ChAT confirmed that the wFMNs were all cholinergic: all NeuN-positive cells expressing ChAT in the FN (n=6 brain sections from 3 mice). n.s., non-signifcance; ACh, acetyl cholinergic pathway.<br>**3** - Measurement of the NeuN positive cell size of co-labelled neurons between the CTB-488-mixed BoNT and CTB-488-alone groups revealed no change in wFMNs soma size following retrograde BoNT: Comparison of the soma size of efected neurons on lFN between the groups of single CTB-488 and CTB-488-mixed BoNT injection. n=3 mice per group, unpaired two-tailed student's t-test, $t=0.9621$, $df=14$, $P=0.3523$. The neuron soma area in each group was normalized to the group of mice injected with single CTB-488.<br>**4** - Co-immunostaining of these specific synaptic markers in brain sections from CTB-488-mixed BoNT-treated mice revealed that these types of fibres all sent projections to wFMNs, illustrating large excitatory neurons of the premotor nucleus.<br><br>• **BoNT inhibits the overexcitation of excitatory neurons in the vlPAG induced by CRS**<br>**5** - c-Fos expression was significantly ↑ in the vlPAG of CRS mice that received saline, BoNT whisker pad injection significantly ↓ the number of c-Fos-positive neurons: Quantifcational analysis of c-Fos numbers among subgroups of naïve mice, saline and BoNT pre-injected CRS mice. n=3 mice per subgroup, One-way ANOVA test and Dunnett's multiple comparisons test comparing each subgroup with the subgroup of CRS mice pre-treated with saline, $F (2, 16)=4.967$, $P=0.0210$. The P-value of Dunnett's multiple comparisons: Naïve+Saline vs. CRS+Saline: $P=0.0274$, CRS+Saline vs. CRS+BoNT: $P=0.0321$.

			•Other assessments (IN VITRO) **unilateral WIM of animals with a transected infraorbital nerve of the trigeminal ganglion** retrograde polytranssynaptic pseudorabies virus (PRV) tracer. EGFP-conjugated PRV (PRV-EGFP) - to rule out interference from afferent sensory regions and investigate the circuitry upstream of wFMNs.<br><br>wFMNs-projecting vlPAG - potential region that might modulate the antidepressant function of retrograde BoNT. vlPAG terminals in the lFN – to ascertain the anatomical synaptic connectivity of vlPAG input to wFMNs.<br><br>c-Fos brain mapping, neuroanatomical tracing, and specific chemogenetic manipulation to evaluate the contribution of the wFMNs-projecting vlPAG neurons to retrograde effects<br><br>Other assessments: behavioural tests (forced swimming test (FST) - despair, sucrose preference test (SPT) – anhedonia, and open field test (OFT) - locomotor ability.	**5** - immunofluorescence revealed that nearly 50% of the activated c-Fos-positive neurons in CRS mice were colocalized with CaMKII-positive neurons, whereas almost 20% were Gad67 positive. The number of c-Fos and CaMKII double-positive neurons was strikingly ↓ in the vlPAG of BoNT-treated CRS mice: Proportion of c-Fos and CaMKII double-labelled neurons in c-Fos-labelled neurons. n=3 mice per group, Dunnett's multiple comparisons test comparing each subgroup with the subgroup of CRS mice who were pre-treated with saline, $F(2, 16)=15.39$, $P=0.0002$. The P-value of Dunnett's multiple comparisons: Naïve+Saline vs. CRS+Saline: $P=0.0002$, CRS+Saline vs. CRS+BoNT: $P=0.0013$.<br><br>Other assessments:<br>• Anatomical neural connectivity of the ipsilateral vlPAG– wFMNs–WIM<br>• Inhibition of vlPAG–wFMNs excitatory neurons of CRS mice mimics facial BoNT antidepressant effects.<br>• wFMNs-projecting vlPAG excitatory neuron activation neutralizes the BoNT antidepressant function.
	LIMITATION: chronic restraint stress (CRS) represents a traditional model extensively used in depressive or anxiolytic studies. Short time window to explore the specific efficiency time of the BoNT. Results obtained from rodents are not generalizable to human feelings and expression. This work was supported by National Natural Science Foundation of China (No. 32071022) and Zhejiang Provincial Natural Science Foundation.			
Zhang, 2019 [32]	(n=) Adult male Sprague–Dawley rats (200–250g)	Trigeminal neuralgia - chronic	**trigeminal spinal subnucleus caudalis**	• All quantitative data were presented as mean±SEM. Because of heterogeneity of variance, statistical significance was evaluated using Kruskal-Wallis and Wilcoxon rank sum tests.

	Rat model of trigeminal neuralgia (ION-CCI)<br>Follow-up: 7-days after BoNT injection (western blots and immunohistochemical staining – harvested tissues)<br><br>Subcutaneously injection into the whisker pad tissue on the operation side, 14 days after ION-CCI:<br><br>Randomly divided into 4 groups:<br>G1. Control + saline (sham; identical operation, but the nerve was not ligated)<br>G2. ION-CCI model + saline<br>G3. ION-CCI+BoNT (3U)<br>G4. ION-CCI+BoNT (10U)	constriction injury to the infraorbital nerve (ION-CCI)	protein expression of transient receptor potential vanilloid type 4 (TRPV4), transient receptor potential melastatin 3 (TRPM3) (optical densities) (by western blots, immunohistochemistry).<br><br>Other assessments: pain threshold (Von Frey hairs)	• The pain thresholds of rats ↓ to a minimum (14 days after ION-CCI).<br>• Compared with ION-CCI group, the pain thresholds of the 3U and 10U groups were significantly ↑ 4 days after the subcutaneous injection of BoNT ($P<0.05$)<br>• The expression of TRPM3 and TRPV4 in ION-CCI was significantly ↑ than that in the control group ($P<0.05$).<br>• The expression TRPM3 and TRPV4 was significantly ↓ in both 3U and 10U group compared with ION-CCI group ($P<0.05$), without significant differences between groups ($P>0.05$).<br>• Overexpression of TRPM3 and TRPV4 can jointly mediate the occurrence of mechanical hyperalgesia in TN and analgesic effects of BoNT may be related to the inhibition of TRPM3 and TRPV4 expression.<br>• Results suggest that BoNT might be transported from the peripheral to the central nervous system by retrograde axonal transport, degrade SNAP-25, block exocytosis, and reduce the protein expression of TRPM3 and TRPV4.
	LIMITATIONS: Control Groups using TRPM3-specific and TRPV4-specific antagonists were not included. Such control groups can be used to test whether mechanical hyperalgesia in rats is reversible. This study was supported by National Nature Science Foundation of China and the Youth Fund of the First Affiliated Hospital of Zhengzhou University.			
Wu, 2016 [23]	(n=?) adult male Sprague-Dawley rats ION-CCI model (220–300g).<br><br>Follow-up: 14-days after ION-CCI (antinociceptive effect G2 vs. G3), 4-,8-,20-days after BoNT (pain thresholds), 7 days after BoNT/21 after ION-CCI (motor coordination, western blot).<br><br>(n=6/group) Subcutaneously into the whisker pad tissue (ipsilaterally to the nerve injury) 14 days after the ION-CCI:<br>G1. ION-CCI+saline+Peripheral BoNT (G1a.3U/Kg, G1b.10 U/kg)<br>G2. Control (ION-CCI+saline+saline)<br>G3. Sham+saline+saline (identical except that the ION was not ligated)<br><br>Colchicine or normal saline (2 µl) was injected into the trigeminal ganglion (ipsilaterally to the nerve injury) of	Trigeminal neuralgia - constriction injury of the infraorbital nerve (ION-CCI)	**Brainstem Vc region (caudal subnucleus of the spinal trigeminal nucleus)**<br>TRPs (transient receptor potential ankyrin 1 (TRPA1), transient receptor potential vanilloid type 1 (TRPV1), transient receptor potential vanilloid type 2 (TRPV2) and transient receptor potential vanilloid melastatin 8 (TRPM8)), and cSNAP-25 (western blot)<br><br>Other assessments: Antinociceptive effect/pain threshold/allodynia was tested by Von Frey filaments, motor coordination ability by Rota-rod test. Colchicine effects.	• All data is expressed as mean ± SD. The statistical significance was assessed using One-way ANOVA and the New-man-Keuls test.<br>• Peripheral application of BoNT (3, 10 U/kg) significantly ↑ the pain threshold of ION-CCI.<br>• 4-days after the subcutaneous injection with BoNT, pain thresholds of each BoNT dose group significantly ↑ compared to the control group ($P < 0.05$).<br>• This antinociceptive effects reached a maximum level at 8 days and remained significantly ↑ until 20 days. The 10 U/kg group had better antinociceptive effects than the 3 U/kg group; however, the difference between them was not statistically significant ($P > 0.05$).<br>• Rota-rod test showed that BoNT administration at doses tested (3U: 255.7 ± 8.5; 10U: 278.6 ± 11.4) did not significantly affect rat motor coordination (compared to control – 3U:268.2 ± 13.8; 10U: 271.8 ± 16.6) $P>0.05$) – BoNT injection into facial trigeminal nerve region did not cause systemic effects in rats even at high doses (10 U/kg), suggesting that BoNT exerts specific antinociceptive function in the CNS without affecting its other functions.<br>• 7-days after BoNT (10U/Kg), the level of (cSNAP-25) in Vc in BoNT G1 significantly ↑ compared to control group ($P <0.05$).<br>• Peripheral application of BoNT (10 U/kg) affected brainstem Vc, which could be blocked by the axonal transport blocker colchicine injection into the trigeminal ganglion - resulted in failure to ↑ the level of cSNAP-25 and in disappearance of the antinociceptive effects of BoNT (10 U/kg) ($P > 0.05$).<br>• In the Vc region of ION-CCI rats, significantly ↑ the expression levels of TRPA1 (day-14 and increased until day-28), TRPV1 (day-14 and increased until day-28), TRPV2 (day-7 and increased until day-28) and TRPM8 (day-7, maximum at day-14, remained increased until day-

	anesthetized rat, 12h before BoNT/saline (ION-CCI+colchicine+saline/BoNT)			28) whereas peripheral application of BoNT significantly ↓ the high expression of TRPA1 and TRPV1 (at day-7 after BoNT in a dose-related manner (P<0.05)), TRPV2 (at day-7, 10U/Kg rather than 3U/Kg), but not TRPM8 (P>0.05) at 7 days after BoNT injection.<br>• The finding of this study suggest that peripherally applied BoNT can produce antinociceptive effects in ION-CCI model. The underlying mechanisms may be BoNT acts on the Vc via axonal transport (colchicine test), ↓ the high expression of TRPA1, TRPV1 and TRPV2, and ↓ central sensitization.<br>• Differences in antinociceptive effects between different doses of BoNT in ION-CCI model of TN were not statistically significant.
	LIMITATIONS: ION-CCI model reproduces important aspects of TN, including signs of abnormal spontaneous pain-related behaviour and mechanical allodynia. cSNAP-25 can be used as a reliable marker of the BoNT diffusion and local action. Vc is the primary relay for orofacial pain and temperature sensations and the site for processing sensory information and plays an important role in the mechanism of TN pathogenesis. It is currently recognized that TRPA1, TRPV1, TRPV2 and TRPM8 play an important role in the pathogenesis of pain sensation production and hyperalgesia and are involved in the perception of pain induced by chemical, temperature or mechanical stimuli. This work was supported by a grant from National Natural Science Foundation of China (Nos. U1404809, 81571260) and the Youth Innovation Fund of the First Affiliated Hospital of the Zhengzhou University.			
Yesudhas, 2021 [34]	(n=12) ageing male BALB/c mice 7-8 months-old. experimental ageing model?<br><br>Follow-up: 30 days after Tx (1st behavioural tests and then animals were sacrificed - hippocampi of brains were dissected out)<br><br>(1U/Kg body weight) single and mild dose intramuscular injection in the thigh:<br>G1. BoNT (n=6)<br>G2. Control-Sterile saline (n=6)	Anxiety and ageing	**Hippocampus (brain) tissues – total protein isolates**<br>**Key antioxidants enzymes (neuroprotection)** – SOD, Catalase, GSH, GPx (U/mg protein)<br>(biochemical analysis)<br><br>Other assessments:<br>1. behavioural tests - open field test (seconds), elevated plus maze (seconds), and light-dark box test (seconds).	•One-way ANOVA was used to assess the statistical significance followed by Tukey's post hoc test. Student t-test was applied for the difference in the mean enzymatic activities of hippocampal antioxidants between control and BoNT.<br>•G1 exhibited better performance in all behavioural tests:<br>Open field (Outer zone: G2= 273 ± 19 vs G1= 237 ± 12: Inner zones: G2 = 27 ± 19 vs G1 = 56 ± 17).<br>Elevated Plus maze (Closed arms: G2 = 249 ± 15 vs G1 = 132 ± 12; Open arms: G2= 38 ± 18 vs G1 = 123 ± 12).<br>Light-dark box test (time spent in light compartment: G2 = 70 ± 31 vs G1 = 106 ± 29; dark compartment: G2 = 230 ± 32 vs G1 = 194 ± 30).<br>•Compared to G2, G1 ↑ the activities of antioxidant enzymes in the hippocampus of ageing experimental animals - biochemical analysis:<br>SOD (G2 = 0.63 ± 0.08 vs G1 = 0.76 ± 0.03),<br>Catalase (G2 = 27.5 ± 3.3 vs G1 = 34.5 ± 2.4),<br>GSH (G2 = 0.29 ± 0.008 vs G1= 0.38 ± 0.05),<br>GPx (G2 = 18.4 ± 2.3 vs G1 = 21.2 ± 1.3)<br>•BoNT prevents level of innate anxiety- related symptoms and increased level of key antioxidant enzymes in the hippocampus of experimental ageing mice (validated its anxiolytic efficacy).<br>•Study suggested that BoNT may be considered as an anxiolytic medication and an agent of neuroprotective measure in the brain.
	LIMITATIONS: Cholinergic crisis and oxidative stress in the hippocampus of the brain have been known to induce anxiety disorders upon ageing. scientific evidence suggests that individuals with adult-onset neurological and mood disorders including anxiety have an increased level of Ach. The vulnerability of the hippocampus to cellular oxidative stress has been linked to the development of anxiety. All experiments were conducted in accordance with the approval of the Institutional Animal Ethics Committee (IAEC) (Ref No: BDU/IAEC/P27/2018, August 07, 2018), under the regulation of the Committee for the Purpose of Control and Supervision of Experiments on Animals (CPCSEA), India. Supported by a research grant (SERB-EEQ/ 2016/000639) from the Science and Engineering Research Board (SERB). M.K. has been supported by the Faculty Recharge Programme, University Grants Commission (UGC-FRP), New Delhi, India. MK would like to acknowledge financial assistance from an Early Career Research Award from SERB (SERB-ECR/2016/000741). AY was supported as JRF in the project grant- SERB-EEQ/2016/ 000639. R.K.R. was supported as JRF from			

	the project grant-SERBECR/2016/000741. MK acknowledges RUSA 2.0, Biological Sciences, Bharathidasan University for the financial assistance, and UGC-SAP and DST-FIST for the infrastructure of the Department of Animal Science, Bharathidasan University.			
Kim, 2015 [26]	(n=) male Sprague-Dawley rats (230-280g)<br>Injected subcutaneously into the vibrissa pad: (40µl 3%) formalin (to assess the inflammatory nociceptive response) or (40µl) CFA (to induce chronic inflammation and to evaluate heat hypersensitivity). Also, microinjection of NMDA (0.5 µg/7 µl) (to evaluate orofacial nociceptive behavioural responses and changes in c-Fos expression to examine trigeminal neurons response)<br><br>Follow-up: (n=8 per group) nociceptive responses recorded for 45min after formalin; (n=8 per group) thermal hyperalgesia (1, 3, 5, 7, 9, 11, 13, 15, and 18 days after CFA); (n=5 per group) c-fos expression after NMDA (?)<br><br>Injection of BoNT, 3 days before formalin, CFA, or NMDA:<br>(1 or 3U/kg, 30µl) **subcutaneously** into the vibrissa pad (peripheral effects);<br>(0.3 or 1U/kg, 10µl) was injected **intracisternally** (central effects).<br><br>G1. (G1a - formalin; G1b - CFA; G1c – NMDA)<br>G2. Vehicle + (G1a or G1b or G1c)<br>G3. Peripheral BoNT (1U/Kg) + (G1a or G1b or G1c)<br>G4. Peripheral BoNT (3U/Kg) + (G1a or G1b or G1c)<br>G5. Central BoNT (0.3U/Kg) + (G1a or G1b or G1c)	Trigeminal nociception - orofacial inflammatory pain models – formalin-induced; complete Freund's adjuvant (CFA)- chronic pain/inflammation; excitatory amino acids – **N-methyl-D-aspartate receptor (NMDA)** (by activating spinal or trigeminal neurons).	**medullary dorsal horn**<br>*c-Fos* expression (number of c-fos immunoreactive neurons)<br><br>Other assessments: orofacial nociceptive behavioural responses - intrathecal or intracisternal induced nociceptive behaviour	• All data are presented as mean±standard error of the mean (SEM).<br>• **Effects of BoNT on the formalin-induced nociceptive behaviour (number of rubbing)**: BoNT subcutaneous injection (3U/Kg, but not lower dose 1U/Kg and vehicle) and intracisternal administration (0.3 and 1U/Kg, but not vehicle) significantly attenuated the formalin-induced nociceptive behaviour in the second phase. $^{*}p<0.05$ vehicle *vs*. BoNT group, n=8 per group.<br>• **Effects of BoNT on CFA-induced thermal hypersensitivity (head withdrawal latency)**: Both subcutaneous and intracisternal injection of BoNT, but not vehicle, attenuated the head withdrawal latency induced by CFA injection. Intracisternal administration (0.3 or 1U/Kg) blocked thermal hyperalgesia $^{*}p<0.05$ vehicle *vs*. BoNT group, n=8 per group.<br>• **Effects of BoNT on the NMDA-induced nociceptive behaviour (number of rubbing):** Intracisternal administration of NMDA evoked a significant nociceptive behaviour by the activation of trigeminal neurons. Both subcutaneous and intracisternal (significantly) attenuated the number of rubbing induced by the intracisternal injection of NMDA. $^{*}p<0.05$ vehicle *vs*. BoNT group, n=8 per group.<br>• **Effects of BoNT on *c-Fos* expression in the medullary dorsal horn**: (A) *c-Fos* immunoreactive neurons in a naïve animal. (B) Intracisternal administration of 0.5 µg NMDA ↑ the number of *c-Fos* immunoreactive neurons in the superficial lamina I and II in the medullary dorsal horn. (C) Subcutaneous administration of BoNT (3 U/kg) ↓ the number of *c-Fos* immunoreactive neurons. (D) Intracisternal injection of BoNT (1 U/kg) ↓ the number of *c-Fos* immunoreactive neurons. (E) The histogram shows the number of *c-Fos* immunoreactive neurons in the ipsilateral medullary dorsal horn. $^{*}p<0.05$ vehicle vs. BoNT group, n=5 per group. Scale bar, 100 µm.<br><br>•peripheral and central administration of BoNT attenuated the formalin-induced nociceptive behaviour and CFA-induced thermal hyperalgesia in the orofacial region. The trigeminal NMDA receptor-mediated nociceptive behaviour was also attenuated by the peripheral and central application of BoNT.<br>•immunohistochemical staining revealed that subcutaneous or intracisternal administration of BoNT downregulated c-Fos expression in the medullary dorsal horn - suggesting that the central antinociceptive effects of BoNT are mediated by transcytosed BoNT or direct inhibition of the trigeminal neurons when the peripheral or central administration of BoNT.

	G6. Central BoNT (1U/Kg) + (G1a or G1b or G1c)			
	LIMITATIONS: The features of formalin-induced inflammatory pain model are inconsistent with those of TN. The long-lasting effects (>30 days) of the CFA assay comprise its main advantage, and it is considered one of the few assays that allow the investigation of the “chronic” phase of pain (https://doi.org/10.3389/fpain.2023.1150749). Preclinical evidence supports the contribution of the N-methyl-D-aspartate receptors (NMDARs, a subclass of ionotropic glutamate receptors) to the trigeminal nociceptive signal processing pathway under various pathological conditions (https://doi.org/10.3389/fncel.2022.999509). In addition, BoNT pretreatment method is not a good clinical simulation of BoNT treatment for TN. supported by the National Research Foundation of Korea (NRF) and funded by the Ministry of Science, ICT & Future Planning (2008-0062282 and 2012M3A9B6055414) and by Hugel Inc.			
Xiong, 2023 [30]	(n=24) New Zealand white female rabbits (age: 6 months, 3.0 ~ 3.3 kg)<br>HS model in rabbit ears.<br>Follow-up: 5 weeks after 1st Tx / 9-weeks after the start of experiment.<br><br>4 weeks postoperatively, rabbits were randomly divided:<br>G1 (n=6) HS+control+same volume of normal saline.<br>G2 (n = 6) HS+ fractional CO2 laser (repeated after 2 weeks)<br>G3 (n = 6) HS+BoNT (2U) injection into HS<br>G4 (n = 6) HS+BoNT + laser	Hypertrophic scar	**Ear tissue - scar-related** protein levels and expression (**TGF-β1, α-SMA, COL-I, COL-III**) (average optical density - immunohistochemistry; protein concentration - western blotting)<br><br>Other assessments:<br>scar thickness, scar elevation index (SEI) (hematoxylin and eosin staining). collagen content and alignment (Masson's trichrome staining), fibroblast proliferation	• Data presented as mean ± standard deviation (SD). Tukey–Kramer and analysis of variance tests using GraphPad Prism 8.0.2 Software (Version X, USA) were used for multiple comparisons and determining p-values among the four groups.<br>• Hypertrophic scars (scar thickness) were ↓ in all Tx groups compared with the control group.<br>• Consistently, western blot and immunohistochemistry analysis results revealed, TGF-β1, α-SMA, and COL-I expression levels were significantly ↓ in the scar tissues of each Tx group ($p < 0.001$) compared with control group.<br>• Compared with the control group, COL-III ↑ after fractional CO 2 laser Tx ($p < 0.001$), whereas it ↓ in the BoNT group ($p < 0.01$), and the effect was more evident in the combination group ($p < 0.001$).<br>• The SEI (the ratio of the distance from the highest point of a scar to the ear cartilage to the distance from the normal skin to the ear cartilage) ↓ significantly in the fractional CO2 laser and BoNT Tx groups than in the control group; however, the effect of the combined therapy was better ($p < 0.001$).<br>• Fractional CO2 laser Tx or BoNT injection inhibited fibroblast proliferation and restored cell polarity.<br>• The content and deposition of collagen fibers were significantly ↓ in the fractional CO2 laser and BoNT Tx groups than in the control group, with a flatter and neat arrangement. The effect of the combined Tx was significant ($p < 0.001$).<br>• The combination group had ↓ scar thickness, SEI, and expression of scar-related proteins in HSs, with an appearance similar to that of normal rabbit ear skin. Furthermore, the fibroblast content and collagen deposition ↓ significantly in the combination group ($p < 0.001$).<br>•western blotting and immunohistochemical analysis results revealed that Tx with fractional CO2 laser or BoNT alone ↓ the expression of TGF-β1 and α-SMA and ↓ collagen deposition. However, fractional CO2 laser + BoNT injection ↑ these effects. Abnormal proliferation of fibroblasts is the most crucial factor leading to HS formation. H&E staining of HS tissues obtained from rabbit ears revealed massive proliferation and disorganized arrangement of fibroblasts in HSs in the control group. H&E staining in each Tx group revealed that the fractional CO2 laser and BoNT Tx effectively ↓ fibroblast proliferation; however, the combined treatment produced better outcomes. SEI is an essential indicator of scar proliferation. Notably, fractional CO2 laser + BoNT Tx can ↓ SEI and cause HSs to be more similar to normal skin.
	LIMITATIONS: there is a significant difference between rabbit ears and human skin; therefore, the rabbit ear model cannot completely represent human HS. Supported by a grant from the Science and Technology Research Project GJJ180005, Jiangxi Provincial Department of Education. TGF-β1 is a crucial pro-fibrosis cytokine, and its high expression is one of the main factors leading to HS			

	formation. TGF-β1 is a significant regulator of tissue repair, inflammation, and fibrosis and can activate multiple cellular pathways, such as the TGF-β1/Smad, ERK, and MAPK pathways, to participate in scar formation. α-SMA is a vital factor in the transformation of fibroblasts into myofibroblasts, and its high expression indicates enhanced fibroblast fibrosis and collagen synthesis. The abnormal ↑ in collagen content in fibroblast ECM is responsible for the appearance and texture of HS and is mainly caused by ↑ expression of COL-I. Because of persistent tension, HSs in these areas are more likely to recur after Tx. BoNT acts on motor nerve endings and can induce chemodenervation by inhibiting acetylcholine binding to receptors. Striated muscle will be functionally denervated for approximately 6 months after injection. BoNT has been used more frequently in recent years to treat HS, owing to its ability to ↓ muscle tension. Furthermore, some studies have reported that BoNT improves the appearance of HSs and inhibits their growth by affecting the cell cycle and regulating the TGFβ signalling pathway. BoNT can also inhibit fibroblast proliferation and differentiation and ECM deposition.			
Cao, 2017 [33]	(n=525) male CD1 (ICR) mice 6-8 weeks old.<br>Follow-up: western blotting/animals sacrificed – 30min, 1, 3, 7, 14-days after BoNT / after behaviour test of chronic model (AEW Tx twice daily for 7 days). (for mRNA and protein levels in DRG; single injection into the neck - BoNT 0.1U)<br><br>Sham Group vs. chronic model (treated with water only)<br>Saline VS BoNT injected in 3 ways:<br>i) itch model (acute and chronic) – single intradermal injection at neck (0.03, 0.1, 0.3, 1U) – for chronic: 1 day before AEW.<br>ii) formalin-induced pain model – subcutaneously injection into hindpaw (0.1U/mice)<br>iii) compound 48/80-induced itch model – repeatedly injected into neck (0.01, 0.03, 0.1U)	Dry skin itch models: acute (histaminic-dependent: compound 48/80-induced; non-histaminic-dependent: chloroquine-induced) and **chronic (induced by acetone-diethylether-water (AEW))**	**dorsal root ganglia (DRG) - transcriptional and translational levels** expression (mRNA, **protein (western blotting)**) of the transient receptor potential cation channel, subfamily V, member 1 (TRPV1), and the **transient receptor potential cation channel, subfamily A, member 1 (TRPA1)**<br><br>Other assessments: behavioural test (scratching behaviour), rota-rod test (motor function)	• Data presented as mean ± standard deviation (SD). An unpaired Student´s t-test was used for two-groups comparisons. One-way analysis of variance with the Bonferroni' post-test for multiple comparisons. Two-way repeated-measures analysis of variance was also used to analyse the data at multiple time points.<br>• Pretreatment of intradermal BoNT exerted long-term inhibitory effects on compound 48/80-induced and chloroquine-induced acute itch following BoNT for 1, 3, 7, and 14 days before acute itch induction, but not on day 21,or after single BoNT (0.03, 0,1, 1U) 30min before compound 48/80, compared with pre-Tx with saline group.<br>• Pre-Tx with BoNT (0.1U) inhibit formalin-induced pain in both phase 1 and 2 – for a longer period than anti-itch activity (significantly ↓ the time of linking and flinching, phase 1: max effect after 3-days and lasted at least 21-days or Phase 2: from 30min to 7-days compared to control).<br>• rota-test: BoNT at higher doses (0.3 and 1.0U), but not lower dose (0.1U) significantly ↓ fall latency, suggesting impairment of motor function.<br>• A single injection of BoNT (0.1U) on acute itch mice significantly ↓ expression of the TRPV1 and TRPA1 at both transcriptional and translational levels in the DRG in mice, and this effect lasted for at least 7-days (mRNA expression - for TRPV1: F (5,17)=11.95, P<0.0001; For TRPA1: F(5,17)=20,29, P<0.0001); (protein expression - for TRPV1: F (5,15)=9,437, P=0.0003; For TRPA1: F(5,13)=5,104, P=0.0083).<br>• Repeated BoNT (3 times) on acute itch mice: significantly ↓ compound 48/80-induced scratching compared with saline, and protein expression of TRPA1 in cervical DRG: F(2,6)=78,40, P<0.0001).<br><br>• Pretreatment of BoNT also attenuated dry **skin-induced chronic itch induced by acetone-diethylether-water Tx** and abolished the **upregulation of TRPA1** in the DRG:<br>- AEW Tx caused intense scratching in mice and significantly ↑ TRPA1 for 3-days compared with sham group: [t(10)=5,414, P=0.0003] and 7 days [t(6)=5,860, P=0.0011]. Data not shown, but reported that expression level of TRPV1 in the DRG was not altered.<br>• BoNT (0.1U) significantly supressed the development of chronic itch on days 5 and 7 [F times(4,56)=40.4, P<0.0001; F group(1,14)=21.32, P=0.0004; F time x group(4,56)=7.622, P<0.0001] – anti-pruritic activity under chronic itch conditions.<br>• BoNT significantly ↓ **protein expression TRPA1** for 3 days [t(8)=4.571, P=0.0018] and 7 days [t(7)=3.169, P=0.0157] compared with saline (n=6-7 mice).

				• It was suggested that downregulation of the expression of TRPA1 and TRPV1 in the DRG may contribute toward the long-term anti-itch effects of a single injection of BoNT in mice and BoNT Tx may serve as an alternative strategy for anti-itch therapy.
	LIMITATIONS: Primary sensory neurons in DRG are responsible for detecting pruritogenic stimuli through their peripheral terminals in the skin and transducing itch signals to the spinal cord through their central terminals. Transient receptor potential (TRP) channels play pivotal roles in multiple somatosensations, such as thermal and mechanical sensation, pain, and itch. It is generally accepted that TRPV1 is required for histamine-dependent itch, whereas TRPA1 is required for histamine-independent itch, such as chloroquine-induced itch, bile acids-induced cholestatic itch, and oxidative stress-induced itch. Previous work showed that upregulation of TRPA1 expression in DRG contributed toward the development of chronic itch related to dry skin. Expression level of TRPV1 in DRG in AEW model – data not shown. Supported by grants from the NSFC (National Natural Science Foundation of China), from the Nature Science Foundation of Jiangsu province, from Second Affiliated Hospital of Soochow University Preponderant Clinic Discipline Group Project Funding, and A Project Funded by the Priority Academic Program Development of Jiangsu Higher Education Institutions.			

LEGEND

CIS, chronic inflammatory state; **BoNT,** botulinum toxin; **(↑)**, statistically significantly higher/increased; **(↓),** statistically significantly lower/decreased; **SEM,** standard error of the mean; **NS**, no statistically significant difference; **NR**, not reported; **ip.l.**, ipsilateral; **c.l.**, contralateral; **s.c.**, subcutaneously; **i.c.**, intracisternally; **POD**, post operative day; **Tx**, treatment; **O.D,** optical density**; (mm)**, millimetres; **SD**, standard deviation; ANOVA; analysis of variance; **CBCT**, Cone Beam computer Tomography; **ELISA**, enzyme linked immunosorbent assay; **qRT–PCR**; Real-Time Quantitative Reverse Transcription PCR**; RGS**, facial grimacing related to pain; **FST**, forced swimming test; **SEI**, scar elevation index; **TEWL,** transepidermal water loss; **HS**, hypertrophic scar; **PRP**, platelet rich plasma; **TNR**; trigeminal nerve root; **CCI**, chronic constriction injury; **NeuN**, neuronal nuclei; **WIM**, whisker intrinsic musculature; **OFT**, open field test; **MMP-13**, matrix metallopeptidase; **TG**, trigeminal ganglia; ; **TNC**, trigeminal nucleus caudalis; **TMD**, temporomandibular disorder; **TMJ**, temporomandibular joint; **OA**, osteoarthritis; **TN**, trigeminal neuralgia; **PIH**, persistent immunogenic hypersensitivity; **TNF-α**, tumor necrotic factor-α; **IL**, interleukin; **ION-CCI/IoNC**, infraorbital nerve constriction; **SNpc**, substantia nigra pars compacta; **mBSA**, methylated bovine serum albumin; **CFA**, Complete Freund's Adjuvant; **PBS**, phosphate-buffered saline; **NTG**, nitroglycerin; **SP**, substance P; **CGRP**, calcitonin gene related peptide; **BDNF**, brain derived neurotrophic factor; **(cl)SNAP-25**, (cleaved) synaptosomal-associated protein-25; **ChAT**, choline acetyltransferase; **PSD95**, postsynaptic density-95; **NMDAR**, N-methyl-D-aspartate receptor; **5-HT**, 5-hydroxytryptamine; **SRS**; spatial restraint stress; **Vc**, caudal subnucleus of the spinal trigeminal nucleus**; p-ERK**, phosphorylated extracellular signal-regulated kinase; **p-CREB**, cAMP response element binding protein; **Iba-1**; ionized calcium-binding adaptor molecule 1; **TLRs** - toll-like receptors; **c-Fos**, neuron activation marker; **GFPA**, glial fibrillary acidic protein; **DNI**, dural neurogenic inflammation; **IgE**, immunoglobulin E; **CX3CR1,** CX3 chemokine receptor 1; **IB4**, isolectin B4-binding; **COL-I**, collagen 1-related proteins; **COL-II**, collagen 2-related proteins; **α-SMA**, α- smooth muscle actin; **HIF-1α**, hypoxia-inducible factor; **TH**, tyrosine hydroxylase, dopaminergic neuronal marker; **VGlut2,** vesicular glutamate transporter 2; **VGAT,** vesicular GABA transporter; **SOD**, superoxide dismutase; **GSH,** glutathione; **GPx,** glutathione peroxidase; **DRG**; dorsal root ganglia; **ATF3**, activating transcription factor 3; **TRPV4**, protein expression of transient receptor potential vanilloid type 4; **TRPM**, transient receptor potential melastatin; **TNCB**, 2-Chloro-1,3,5-trinitrobenzene; **TRPV,** transient receptor potential vanilloid type ; **TRPA1**, transient receptor potential ankyrin 1; **Vc**, caudal subnucleus of the spinal trigeminal nucleus; **AEW**, acetone-diethylether-water; **TGF-β1**, transforming growth factor beta; **vAChT**, acetylcholine vesicular transporter; **SerT**, serotonin transporter; **CTB-488**, monosynaptic retrograde tracer; **PRV-EGFP**, retrograde polytranssynaptic pseudorabies virus (PRV) tracer, EGFP-conjugated; **wFMNs**, whisker-innervating facial motoneurons; **CaMKII**, excitatory Ca^{2+}/calmodulin-dependent protein kinase type II; **GAD67**, inhibitory glutamate decarboxylase 1; **vlPAG,** ventrolateral periaqueductal grey; **TpH2**, tryptophan hydroxylase 2, serotoninergic neuronal marker; **GAPDH,** Glyceraldehyde 3-phosphate dehydrogenase
