# Supplementary material for: Botulinum Toxin Effects on Biochemical Biomarkers Related to Inflammation-Associated Head and Neck Chronic Conditions: A Systematic Review of Preclinical Research
Source: Toxins (Basel). 2025 Jul 29;17(8):377. doi: 10.3390/toxins17080377 (PMC12390450; doi:10.3390/toxins17080377)
Supplement: Supplementary file 1 [file toxins-17-00377-s001.zip › SR2. file S4. Table S3. RoB.pdf]

File S4. Table S3. risk of bias assessment - SYRCLE's tool

TYPE OF BIAS	SELECTION BIAS			PERFORMANCE BIAS		DETECTION BIAS		ATTRITION BIAS	REPORTING BIAS	OTHER
**DOMAIN / STUDY**	**1* Sequence generation**	**2 Baseline characteristics**	**3* Allocation concealment**	**4 Random housing**	**5 Blinding**	**6 Random outcome assessment**	**7 Blinding**	**8* Incomplete outcome data**	**9* No selective outcome reporting**	**10* No other sources of bias**
Makawi, 2022 [13]	√ i	√<br>•balanced demographics: male adult Wister albino rats, 3-4 months (180 - 200g).<br><br>•only male mice (uneven gender distribution).<br><br>•4 weeks after induction, n=2, each group sacrificed for confirmation of OA – histological, x-ray, PCR+ELISA (IL-1β) analysis.<br><br>•n=3, control group using the right and left TMJs to detect baseline levels before the induction of AO.<br><br>•Adequate timing of disease induction - before randomization of the intervention	? ii	√<br>All animals were housed in a sterile, controlled environment at a temperature of 28 to 35ºC and maintained throughout the experiments on a 12:12 h light-dark cycle (housing conditions are identical/ comparable).	? ii<br>•unclear if caregivers and/or investigators blinded from knowledge which intervention each animal received, but 3 different individuals performed statistical data, radiographic, and biochemical analysis.<br><br>•circumstance during the intervention were similar in treatment groups	X<br>•animals in control vs experimental group were sacrificed at different time points, thus unlikely animals were selected at random for outcome assessment.<br><br>•animals in experimental groups were sacrificed at the same time points.	? ii<br>•unclear if outcome assessors were blinded.<br><br>•outcome assessment methods were the same in control and treatment groups – objective measures.<br><br>•experimental groups - same timing for outcome assessments (2 weeks - histological, biochemically, 4 weeks post-treatment - radiograph, biochemically).	√<br>All animals included for analysis, balanced in numbers across intervention groups, with similar reasons for missing data across groups	√ iii	√<br>•the authors certify that there was no specific funding for the research and publication of this article.<br><br>•there is still not a clear "gold standard" for choosing the best animal model for OA

Shao, 2013 [19]	? i	?<br>•balanced demographics: adult female Sprague-Dawley rats (250-300g), unknown age.<br><br>•only female mice (uneven gender distribution).<br><br>•(n=8) served as control (before induction of disease).<br><br>•Most likely adequate timing of disease induction - before allocation of the intervention – but unclear.	? ii	√<br>Housed individually in cages. The animals were raised and maintained under standard laboratory conditions with a 12-hour light-dark cycle (light on 07:00-19:00 h). Food and water were offered ad libitum. (housing conditions are identical/ comparable). All the protocols followed the guidelines (86/609/ ECC)	? ii<br>•Unclear if caregivers and/or investigators blinded from knowledge which intervention each animal received during the experiment.<br><br>•circumstance during the intervention were similar in treatment groups.	?<br>•unclear, but likely that all animals (control and experimental groups) were randomly beheaded 24h after vehicle or BoNT injection for 2 ml blood withdrawn and medulla oblongata containing caudal trigeminal nucleus harvest and radioimmuno assay (RIA) – same time point	? ii<br>•unclear if outcome assessors were blinded.	√<br>•” Every effort was made to minimize the numbers and any suffering of the animals used” – unlikely, but unclear if more than the reported 32 animals were used.<br><br>•animals included for analysis, balanced in numbers across groups (n=8).	√iii	?<br>•the work was supported by Scientific and Technique Support Project of Gansu Province and Medical Subject Fund of Lanzhou Universit.
Li, 2019 [21]	x	?<br>•balanced demographics: male adult ICR mice (6 weeks–8 weeks, 20g–25g).<br><br>•only male mice (uneven gender distribution).<br><br>•unclear sample-size.<br><br>•Most likely adequate timing of disease induction - before	? ii	√<br>•all animals were maintained under a 12-h light/dark cycle with food and water ad libitum, and the room was kept at 22 ± 2 ◦C and 40%–60% humidity. All mouse experiments were performed between 09:00 and 16:00.	? ii<br>•unclear if caregivers and/or investigators blinded from knowledge which intervention each animal received during the experiment.	?<br>•unclear if animals were selected at random for outcome assessment and/or at different time points.	? ii<br>•unclear if outcome assessors were blinded.<br><br>•outcome assessment methods were the same in control and treatment	?<br>•unclear information regarding sample size. Thus, unclear if all animals were included in the analysis or if any missing	X<br>•exploratory study – outcomes described in methods (5-HT level, depressive-like behaviour tests) and results	X<br>•design-specific risks of bias: lack of placebo group.<br><br>•supported by grants from the National Natural Science Foundation of China, and the Natural Science

		allocation of the intervention – but unclear.		Four mice were housed in each cage. All animal experiments were performed according to the National Institutes of Health Guide for the Care and Use of Laboratory Animals.<br><br>•mice in the control group remained undisturbed in their home cages without access to water and food, like the SRS mice.	•circumstance during the intervention were similar in treatment groups and control - Instruments used to conduct experiment similar between experimental and control group.<br><br>•lack of placebo.		groups – objective measures.	outcome data.	section with differences (added BDNF protein and mRNA expression, expression of SNAP25, NMDAR subunits and ERK-CREB pathway).	Foundation of Jiangsu Province, China, Jiangsu Key Laboratory of Neuropsychiatric Diseases, the Second Affiliated Hospital of Soochow University Preponderant Clinic Discipline Group Project Funding, the Postgraduate Research and Practice Innovation Program of Jiangsu Province, China, Suzhou Science and Technology For People's Livelihood, the Postgraduate Research and Practice Innovation Program of Jiangsu Province, China.
Chen, 2021 [14]	√ i	√<br>•balanced demographics: male C57BL/6 mice 6-8 week-old, 20g.<br><br>•TLR2 knockout mice (Tlr2−/−; stock#005846), was further used to investigate the role of	? ii	√<br>•housed at 5 to 6 per cage, habituated to the colony room for a week before the experiments were performed, and kept under a 12 h light/12	√<br>•the group of mice during testing was blinded to the experimenter of the behavioral test.	?<br>•unclear if animals were selected at random for outcome assessment.	? ii<br>•unclear if outcome assessors were blinded.<br><br>•outcome assessment	X<br>•only data from four mice were used for statistical analysis –	?<br>•some outcomes have not been comprehensi vely discussed –	?<br>•supported by Suzhou science and technology plan key technology application research project,

		TLR2 on the development of persistent pain in the CCI mouse model.<br><br>•only male mice (uneven gender distribution).<br><br>•unbalanced sample size among groups.<br><br>•adequate timing of disease induction - before randomization of the intervention.		h night cycle. The rearing environment was maintained at a constant room temperature of 22 °C and 60%–80% humidity. In addition, water and food were freely available. All procedures were approved by the Animal Care and Use Committee of Soochow University. The number of mice used in each experiment are available. This animal study followed the ARRIVE guidelines.		•unclear, but likely the same time points for outcome assessment - 5 day after BoNT/19-day after CCI (western blotting, RT-PCR, immunofluorescence).	methods were the same in control and treatment groups – objective measures.	unclear reasons.	Gfap, TLR3, TLR6, TLR7, TLR9, TLR12, TLR13	and the National Natural Science Foundation of China.<br><br>•there are several other TN animal models.<br><br>•flaccid paralysis of the facial muscles at the injection site (potential side effects).
Han, 2017 [27]	√ i	√<br>•balanced baseline characteristics: female 6 weeks-old NC/Nga mice (mouse model for atopic dermatitis) + contact sensitizer (TNCB)<br><br>•unclear demographics (weight of the animals)<br><br>•unbalanced sample size among groups explained (in case severe side effects occur).	? ii	√<br>•mice were kept under a 12h light/12h dark cycle and maintained under conventional conditions at 24±1◦C and 50 ±10% humidity. Water and food were freely available. The animal care, handling, and experimental procedures were performed in	√<br>•Tx administrator and assignment not involved in outcome assessment.	?<br>•unclear if animals were selected at random for outcome assessment.<br><br>•unclear, but likely the same time points for outcome assessment - day-14 after 1st challenge.	√<br>•outcome assessor masked to study purpose and hypothesis, and not involved in Tx administration and assignment.	√<br>•all animals included for analysis.<br><br>•unbalanced in numbers across intervention groups (6 vs. 9) with reasons (in case severe side effects occur).	√ iii	X<br>•design-specific risks of bias: data limited to 14 days (further studies need to identify the duration of effects of BoNT as well as the exact dose), small sample size.<br><br>• NC/Nga mice have been associated to low incidence of AD-

		•adequate timing of disease induction - before randomization of the intervention		accordance with a protocol approved by the Animal Care and Use Committee, and all procedures followed the U.S. National Institutes of Health guidelines.						like lesions, late onset of the disease, and poor reproducibility are disadvantages. (thus, NC/Nga mice model was modified - contact sensitizers were adopted).<br><br>•supported by a grant of the Korean Healthcare technology R&D project, Ministry of Health & Welfare, Republic of Korea. The authors have indicated no significant interest with commercial supporters.
Muñoz-Lora, 2022 [22]	√ i	√<br>•balanced baseline characteristics: male Sprague–Dawley rats (300–400g, 6–8 weeks).<br><br>•only male mice (uneven gender distribution).<br><br>•balanced sample size among groups.<br><br>•adequate timing of disease induction - before	? ii	√<br>•animals were housed in plastic cages with a maximum of 3 animals per cage. Animals were kept in a room with a controlled constant temperature (22 ± 0.5 ◦C) and a 12h:12h light:dark cycle (lights on from 07:00 to 19:00).	? ii<br>•unclear if caregivers and/or investigators blinded from knowledge which intervention each animal received during the experiment.<br><br>•circumstance during the intervention were similar in	?<br>•unclear if animals were selected at random for outcome assessment.<br><br>•unclear, but likely the same time points for outcome assessment - day-13 (pre-	? ii<br>•unclear if outcome assessors were blinded.<br><br>•outcome assessment methods were the same in control and treatment groups – objective measures.	?<br>•”all efforts were made to reduce the number of animals”. Thus, unlikely, but unclear, if more than the reported 40 animals were used.	√ iii	X<br>•there are many animal models of RA-related pain, including TMJ-RA, that have been proposed. This model has a monoarthritic (single joint) presentation, since it is induced locally inside the mBSA-stimulated joint.

		randomization of the intervention.		Food and water were available ad libitum.<br><br>•animal procedures were approved by the Ethics Committee of the University of Zagreb and conducted according to the European Communities Council Directive and the International Association for the Study of Pain guidelines.<br><br>•all data was presented in accordance with the ARRIVE guidelines	treatment groups and control - Instruments used to conduct experiment similar between experimental and control group, timing of administrations.	formalin), day-14 (post formalin)/day -56 (behavioural assessment), day-57 (behavioural assessment, sacrifice, biochemical assessment).		•balanced in numbers across all groups (n=10).<br><br>•only data from 5 animal/group were used for statistical analysis – unclear reasons.		•funded by Ipsen- the author is an employee of Ipsen Innovation, France.
Muñoz-Lora, 2017 [15]	?	√<br>•balanced baseline characteristics: Male Wistar rats (250–500g).<br><br>•only male mice (uneven gender distribution).<br><br>•unclear demographics (age of the animals).<br><br>•unknown sample size.<br><br>•adequate timing of disease induction - before allocation of the intervention.	? ii	√<br>•animals housed in standard clear plastic cages with a maximum of four animals per cage and maintained in a temperature-controlled room (23 ± 1 ∘C) with a 12 h dark-light cycle (lights on at 06:00 a.m.) with free access to food and water.	X<br>•unclear, but not likely, that the caregivers and/or investigators were blinded from knowledge which intervention each animal received during the experiment.<br><br>•circumstance during the intervention were different in Tx	X<br>•animals in experimental group were sacrificed at different time points (24h or 14-days after BoNT treatment), thus unlikely animals were selected at random for outcome assessment.	?<br>•evaluations were made by a researcher blind to the group assignment.<br><br>•unclear outcome assessment methods in control and placebo group.	?<br>•unclear information regarding sample size. Thus, unclear if all animals were included in the analysis or if any missing outcome data.	?<br>•placebo/ saline treatment was not pre-specified in the protocol.<br><br>•sample sizes for each group were not specified.	√<br>•did not receive any specific grant from funding agencies in the public, commercial, or not-for-profit sectors. This study was supported by Sao Paulo Research Foundation and National Council for Scientific and

				•study was approved by the Ethics Committee in Animals Research of the State University of Campinas, following the National Council for Control of Animal Experimentation (CONCEA) and the International Association for the Study of Pain (IASP) guidelines.	groups and control - Instruments used to conduct experiment differ between experimental and control group, as well as timing of administrations/ assessments: "After 24 h or 14 days of BoNT Tx, an intra-articular injection of formalin (0,5%) was administered. Immediately after the formalin injection, the behavioral nociceptive response was evaluated. After behavioral evaluation, animals were terminally anesthetized and trigeminal ganglia were removed and stored".	•unclear assessment-time points for non-immunised or placebo protocols.				Technological Development.
Muñoz-Lora, 2020 [16]	√ i	√<br>•balanced baseline characteristics: Male Wistar rats (300–400g).<br><br>•only male mice (uneven gender distribution).	? ii	√<br>•housed in plastic cages with a maximum of 4 animals per cage at a controlled room temperature (23±1	?<br>•unclear, if the caregivers and/or investigators were blinded from knowledge which intervention each	X<br>•animals in experimental group were sacrificed at different time points (24h,	√<br>•all injections were applied by two experimenters that had no participation in	?<br>•"all efforts were made to reduce the number of animals that were	√ iii	?<br>•supported by grants from Brazilian governmental financial support by the Sao Paulo

		•unclear demographics (age of the animals)<br>•balanced sample size.<br>•adequate timing of disease induction - before allocation of the intervention		◦C) and a 12 h/12 h, light/dark cycle.<br><br>•study was approved by the Ethics Committee in Animals Research of the State University of Campinas and followed the guidelines from the National Council for Control of Animal Experimentation (CONCEA), ARRIVES guidelines and the International Association for the Study of Pain	animal received during the experiment, but all injections were applied by two experimenters that had no participation in the biochemical analysis.<br><br>•circumstance for assessments were different in treatment groups and control - timing of assessments: “Animals were deeply anesthetized and euthanized 24 h, 7 or 14 days after BoNT treatment, according to each group”	7-days or 14-days after BoNT treatment), thus unlikely animals were selected at random for outcome assessment.<br><br>•unclear assessment-time points for non-immunised or placebo protocols.	the biochemical analysis.<br><br>•unclear outcome assessment methods/ in control and placebo group.	randomly divided into 5 groups”. Thus, unlikely, but unclear, if more than the reported 40 animals were used.<br>•balanced in numbers across all groups (n=8)		Research Foundation as a Doctoral Fellowship and by the National Council for Scientific and Technological Development as a Doctoral Fellowship and as researcher productivity fellowship.<br><br>•this model is useful to assess immunomodulatory mechanisms. However, there are other mechanisms involved in the pathogenesis of the model that may also be related to BoNT activity.
Filipović, 2012 [28]	X	√<br>•balanced baseline characteristics: Male Wistar rats (300–350g).<br><br>•only male mice (uneven gender distribution).<br><br>•balanced sample size.	? ii	√<br>•animals were kept under constant 12h/12h light/dark cycle with unlimited access to food and water. The experiments were conducted according to the National Institute of	?<br>•unclear, if the caregivers and/or investigators were blinded from knowledge which intervention each animal received during the experiment.	?<br>•unclear if animals were selected at random for outcome assessment.<br><br>•same time points for outcome	? ii<br>•unclear if outcome assessors were blinded.<br><br>•outcome assessment methods were the same in control and	X<br>•balanced in numbers across all groups (n=20) and (n=8).<br><br>•only data from 8 animal/group	√ iii	√<br>•no financial support or conflict of interests declared.<br><br>•Infraorbital nerve constriction injury (IoNC) accompanied by hyperalgesia and

		•unclear demographics (age of the animals).<br><br>•adequate timing of disease induction - before allocation of the intervention.		Health Guide for the Care and Use of Laboratory Animals and approved by the Ethical Committee of the University of Zagreb, School of Medicine.		assessment - 3-days after BoNT/17-days post-IoNC (without/with formalin).	treatment groups – objective measures.	were used for assessment with formalin – unclear reasons.<br><br>•only data from 5 or 4 animal/group were used for statistical analysis – unclear reasons.		allodynia is used as a model of trigeminal neuropathy in rats.<br><br>•doses based on the doses used in other pain models.
Kitamura, 2009 [29]	X	?<br>•balanced baseline characteristics: adult male Sprague–Dawley rats (200–250g).<br><br>•only male mice (uneven gender distribution).<br><br>•unclear demographics (age of the animals).<br><br>•unclear sample size.<br><br>•Most likely adequate timing of disease induction - before allocation of the intervention – but unclear.	? ii	?<br>•study was performed in accordance to specifications of an animal protocol approved by Okayama University. All experiments were conformed to relevant National Institutes of Health guidelines on the ethical use of animals.	?<br>•unclear, if the caregivers and/or investigators were blinded from knowledge which intervention each animal received during the experiment.	?<br>•unclear if animals were selected at random for outcome assessment.<br><br>•same time points for outcome assessment - day-14 post-IoNC/ 11-days after BoNT/saline injection.	? ii<br>•unclear if outcome assessors were blinded.<br><br>•outcome assessment methods were the same in control and treatment groups – all experiments were performed within 1.5 h of tissue harvest - objective measures.	?<br>•” We minimized the number of animals used and their suffering”.<br><br>•unclear information regarding sample size. Thus, unclear if all animals were included in the analysis or if any missing outcome data.	√ iii	?<br>• supported by a grant from the Ministry of Education, Science and Culture of Japan, Ryobi Teien Memorial Foundation and Japanese Association for Dental Science.

								•unbalanced in numbers across all groups for each assessment – unclear reasons.		
Lacković, 2016 [20]	√ i	√<br>•balanced baseline characteristics: male Wistar rats (300–350g; 3–3.5 months old).<br><br>•only male mice (uneven gender distribution).<br><br>•balanced sample size.<br><br>•Methylene blue was injected into a few animals to confirm successful targeting of the TMJ in disease-model induction.<br><br>•Most likely adequate timing of disease induction - before allocation of the intervention – but unclear.	? ii	√<br>•Rats were kept under a constant 12 h/12 h light/dark cycle with free access to food and water.<br><br>•All animal care and experimental procedures were in accordance with the 2010/63/EU Directive on the protection of animals used for scientific purposes and the recommendations of International Association for the Study of Pain and were approved by the Ethical Committee of University of Zagreb School of Medicine. The experimental procedures used in the work described	√<br>•The experimenter conducting the behavioural testing was unaware of the treatments given to the animals.	?<br>•unclear if animals were selected at random for outcome assessment.<br><br>•different time points for outcome assessment - 24h/1-day after CFA/4-days after BoNT or 2h after sumatriptan* (behavioural assessment* , DNI plasma extravasation *, and RIA for CGRP), unclear (histology and immunohisto chemistry of dura mater)	? ii<br>•unclear if outcome assessors apart from behavioural testing were blinded.<br><br>•outcome assessment methods were the same in control and treatment groups – objective measures.	X<br>•some results for Cleaved SNAP-25 colocalizes with CGRP-expressing afferents of the dura mater after BoNT peripheral treatment are not shown.<br><br>•unclear information regarding sample size. Thus, unclear if all animals were included in the analysis or if any missing outcome data.	√ iii	?<br>•supported by grants from Croatian Ministry of Science, Education and Sport, Croatian National Science Foundation, Hungarian National Brain Research Program and National Brain Research Program B.<br><br>•The recommended protocol for BoNT application in chronic migraine consists of multiple injections to 31 head and neck sites. A similar protocol is difficult to replicate in rats because of the

				in this article were as humane as possible. All animal studies are described in compliance with the ARRIVE guidelines for reporting experiments.				•unbalanced in numbers across all groups for each assessment – unclear reasons.<br><br>•only data from 5 animal/group were examined for histological study of the cranial dural tissue – unclear reasons.		smaller cranial dimensions.
Wang, 2020 [25]	√ i	?<br>•balanced baseline characteristics: healthy, mature New Zealand big-ear albino rabbits (no sex restriction) 2.5~3.5 kg.<br><br>•there is no animal weight comparison in this study, which may interfere the formation of hypertrophic scar tissue during 4 weeks' feeding.<br><br>•unclear gender distribution.<br><br>•unclear demographics (age of the animals).	? ii	√<br>•All animals were approved by the animal care and use committee.<br><br>•The rabbits were reared in the cage separately under standard conditions at 25 ± 2o C and eat freely.<br><br>•All animal experiments were conducted according to Principles of	?<br>•unclear, if the caregivers and/or investigators were blinded from knowledge which intervention each animal received during the experiment.<br><br>•circumstance for assessments were the same in treatment groups and control - timing of assessments: 60	?<br>•unclear if animals were selected at random for outcome assessment.<br><br>•different time points for outcome assessment – 60-, 28-days after injection.	? ii<br>•unclear if outcome assessors were blinded.<br><br>•outcome assessment methods were the same in control and treatment groups – objective measures.	√<br>•all animals included for analysis.<br><br>•balanced in numbers across intervention groups.	? iii<br>•the specific regulation mechanism of BoNT on the protein has not been clear/ discussed	X<br>•design-specific risks of bias: the action time of drug is short and concentration point selection is not enough; the suitable concentration range for BoNT has not been clearly studied.<br><br>•This study was funded by Anhui Science and

		•balanced sample size.<br><br>•adequate timing of disease induction - before allocation of the intervention.		Laboratory Animal Care (National Society for Medical Research). This study was approved by the Animal Ethics Committee of Anhui Medical University.	days after injection (scar tissue harvested), day-28 after the injection of BoNT (apoptotic effect), but different site for injections: left and right ear according to groups/Tx					Technology Research Project.
Cho, 2022 [17]	√ i	?<br>•balanced baseline characteristics: male Sprague-Dawley rats (250−280 g).<br><br>•only male mice (uneven gender distribution) – reason: to exclude the effects of sex hormones on nociceptive thresholds because pain threshold and tolerance have been found to be substantially smaller in female than male. Sex hormones play a key role in contributing to gender differences.<br><br>•unclear demographics (age of the animals).<br><br>•unclear sample size among groups or groups definition.<br><br>•adequate timing of disease induction - before allocation of the intervention.	? ii	√<br>•rats were housed 3 to 4 per cage in a temperature-controlled room (23 ± 1°C) with 12/12-h dark/light cycle and had free access to food and water.<br><br>•all procedures and animal experiments followed experimental protocols (pain grade E) approved by the Institutional Animal Care and Use Committee of the School of Dentistry, Kyungpook National University. All evaluations with animal were carried out in accordance with the ethical principles	√ iv<br>•all animal groups underwent a single-blind randomization carried out by one of the researchers.	?<br>•unclear if animals were selected at random for outcome assessment.<br><br>•same time points for objective outcome assessment – 2-days after BoNT injection.	? ii<br>•unclear if outcome assessors were blinded.<br><br>•outcome assessment methods were the same in control and treatment groups – objective measures.	?<br>•unclear information regarding sample size among groups. Thus, unclear if all reported sample (n=236) animals were included in the analysis or if any missing outcome data.<br><br>•balanced in numbers across intervention groups (n=6/group) for objective outcomes.	√ iii	?<br>•no funding or conflicts information available.<br><br>•There are no current animal models of TN that adequately replicate the clinical disorder, because the exact underlying causes of TN are not known. Most used neuropathic animal model following constriction injury of infraorbital nerve. Although this animal model cannot perfectly replicate the clinical disorder, the main theory for the

				recommended by the International Association for the Study of Pain (IASP)				•model - HIF-1α expression compared with the sham group (data not shown)		pathophysiology of TN has been linked to the compression of the TNR.
Li, 2023 [18]	?	?<br>•balanced baseline characteristics: Adult (approximately 30 g, 6–8 weeks old) male ICR mice.<br><br>•all mice were weighed on the day before the behavioral tests. The reserpine treatment did not significantly change the body weight of the mice.<br><br>•several tests were conducted to confirm that chronic administration of reserpine could reproduce remarkable depression-like symptoms and pathological changes in the mouse model of Parkinson's disease.<br><br>•only male mice (uneven gender distribution) - many studies have found that antidepressant therapies in the female mouse models of depression are affected by the estrus cycle and estrogen.<br><br>•unclear sample size.	? ii	√<br>•animals were fed in an environment with a pressure ventilation system, an ambient temperature maintained at 22 ± 2 °C, and a relative humidity maintained at 40%–70%. Mice were housed in a 12-h/12-h light/dark cycle (8:00–20:00) and provided with standard mouse chow and water ad libitum.<br><br>•mice were fed a reserpine solution (prepared with drinking water, 3 μg/mL) daily for 10 weeks.<br><br>•All animal experiments were conducted according to the	?<br>•unclear, if the caregivers and/or investigators were blinded from knowledge which intervention each animal received during the experiment.<br><br>•unclear if circumstance for assessments were the same in treatment groups and control - timing of assessments unknown.	?<br>•unclear if animals were selected at random for outcome assessment.<br><br>•behavioural tests were mainly carried out in the experimental animal operating room from 9:00 a.m. to 16:00 p.m., and the time points were adjusted according to the experimental designs.<br><br>•same time points for objective	? ii<br>•unclear if outcome assessors were blinded.<br><br>•outcome assessment methods were the same in control and treatment groups – objective measures.	?<br>•unclear information regarding sample size among groups. Thus, unclear if all animals were included in the analysis or if any missing outcome data.<br><br>•only data from limited animal/group (varied between 3 to 5-6 animals/ group) were examined – unclear reasons.	√ iii	?<br>•reserpine-induced model has been widely applied to investigate the role of the monoamine system in the regulation of motor and psychiatric disorders, including PD and depression.<br><br>•synapse loss and deficits in synaptic plasticity associated with depression are broadly distributed throughout the central nervous system. Given the postmortem studies of structural alterations in the hippocampus, the study mainly focused on

		•unclear, but most likely adequate timing of disease induction - before allocation of the intervention.		National Institutes of Health Laboratory Animal Care and Use Guidelines. All animal operations and experimental procedures were approved by the Animal Ethics Committee of Soochow University		outcome assessment –				structural synaptic plasticity – limited brain areas to allow generalizability.<br><br>•supported by grants from the National Natural Science Foundation of China, Natural Science Foundation of Jiangsu Province, Suzhou Medical and Health Technology Innovation Project, startup funding and research funding by the Clinical Research Center of Neurological Disease in The Second Affiliated Hospital of Soochow University; the Suzhou Science and Technology Plan Key Technology Application Research Project, the National Natural Science	

											Foundation of China, the Suzhou Clinical Research Center of Neurological Disease and the Natural Science Foundation of Jiangsu Province of China; and the Priority Academic Program Development of Jiangsu Higher Education Institutes (PAPD)
Yang, 2016 [31]	?	?<br>•balanced baseline characteristics: male Sprague-Dawley rats (220 and 240g).<br><br>•only male mice (uneven gender distribution) excluded the effects of sex hormones on nociceptive thresholds - found to be substantially smaller in female than male. Sex hormones play a key role in contributing to gender differences.<br><br>•unclear demographics (age of the animals).<br><br>•unclear total sample size.	? ii	?<br>•all procedures involving the use of animals were approved by the Institutional Care and Use Committee of the School of Dentistry, Kyungpook National University, and were carried out in accordance with the ethical guidelines of the International Association for the Study of Pain for the investigation of experimental pain in conscious animals.	√<br>•all behavioural responses were measured in a blind fashion.<br><br>•circumstance for assessments were different in treatment groups - timing of assessments: in some animals, antinociceptive effects were evaluated after repeated administration of low doses of BoNT-A (0.3 or 1 U/kg) on PODs 3 and 4, respectively	?<br>•unclear if animals were selected at random for outcome assessment.<br><br>•same time points for objective outcome assessment – POD 9/ 6-days after BoNT (3U/Kg).	? ii<br>•unclear if outcome assessors (for biomarkers) were blinded.<br><br>•outcome assessment methods were the same in control and treatment groups – objective measures.	?<br>•unclear information regarding total sample size. Thus, unclear if all animals were included in the analysis (n=5 per group) or if any missing outcome data.<br><br>•unclear sample size for statistical analysis - n = 5 to 8	√ iii	?<br>•supported by the National Research Foundation of Korea (funded by the Ministry of Science, ICT and Future Planning) and by Hugel, Inc.

		•most likely, adequate timing of disease induction - before allocation of the intervention.						animals per group?<br><br>•injection of BoNT (3 U/kg) into the hind leg of rat model did not induce antiallodynic effects - data not shown.		
Ni, 2023 [24]	√ i	?<br>•balanced baseline characteristics: pathogen-free C57BL/6J male mice (8 weeks of age, 25g).<br><br>•only male mice (uneven gender distribution) - many studies have found that antidepressant therapies in the female mouse models of depression are affected by the estrus cycle and estrogen.<br><br>•unclear total sample size.<br><br>•unclear, but most likely adequate timing of disease induction - before allocation of the intervention.	? ii	√<br>•housed in standard transparent plastic cages (330×205×180 mm) with four mice per cage under a 12-h:12-h light–dark cycle with ad libitum access to food and water. Mice were habituated for 1 week before formal experiments.<br><br>•All mice were returned to their home cages for free moving after administration.	√<br>•experimenters were blinded to all subgroup allocation during the experiments.<br><br>•circumstance for assessments were different in treatment groups - timing of assessments: pre-injecting into the unilateral whisker intrinsic musculature (WIM) with 3 dosages at 3 different time points prior to the end of the restraint period	?<br>•unclear if animals were selected at random for outcome assessment.<br><br>•different time points for objective outcome assessment, but all mice were sacrificed for tissue section preparation 24h after behavioural tests.	? ii<br>•unclear if outcome assessors (for biomarkers) were blinded.<br><br>•outcome assessment methods were the same in control and treatment groups – objective measures.	?<br>•unclear information regarding total sample size. Thus, unclear if all animals were included in the analysis or if any missing outcome data.<br><br>•unbalanced and different sample sizes for statistical analysis according to each outcome.	√ iii	?<br>• This work was supported by National Natural Science Foundation of China and Zhejiang Provincial Natural Science Foundation.<br><br>•design-specific risks of bias: short time window to explore the specific efficiency time of the BoNT.<br><br>•chronic restraint stress (CRS) represents a traditional model extensively used in depressive or anxiolytic studies.

											•results obtained from rodents are not generalizable to human feelings and expression.
Zhang, 2019 [32]	√ i	?<br>•balanced baseline characteristics: adult male Sprague-Dawley rats (200 and 250g).<br><br>•only male mice (uneven gender distribution) excluded the effects of sex hormones on nociceptive thresholds - found to be substantially smaller in female than male. Sex hormones play a key role in contributing to gender differences.<br><br>•unclear demographics (age of the animals).<br><br>•unclear sample size.<br><br>•most likely, adequate timing of disease induction - before allocation of the intervention	? ii	√<br>•kept at room temperature (~22∘C), with a light cycle of 12h light/ 12h dark, and with adequate food and drinking water.<br><br>•all experimental procedures were approved by the Animal Experimental Ethics Committee of Zhengzhou University.	√<br>•the tester (mechanical pain threshold test) was blinded to the group allocation of rats.	?<br>•unclear if animals were selected at random for outcome assessment.<br><br>•same time points for objective outcome assessment, all mice were sacrificed for tissue section preparation – 7-days after Tx.	? ii<br>•unclear if outcome assessors (for biomarkers) were blinded.<br><br>•outcome assessment methods were the same in control and treatment groups – objective measures.	?<br>•unclear information regarding total sample size. Thus, unclear if all animals were included in the analysis or if any missing outcome data.	√ iii	?<br>•design-specific risks of bias: control groups using TRPM3-specific and TRPV4-specific antagonists to test whether mechanical hyperalgesia in rats is reversible were not included.<br><br>•supported by National Nature Science Foundation of China and the Youth Fund of the First Affiliated Hospital of Zhengzhou University.<br><br>•There are no current animal models of TN that adequately replicate the clinical disorder, because the exact underlying causes

											of TN are not known. Most used neuropathic animal model - following constriction injury of infraorbital nerve.
Wu, 2016 [23]	? i	?<br>•balanced baseline characteristics: adult male Sprague-Dawley rats ION-CCI model (220–300g).<br><br>•only male mice (uneven gender distribution) excluded the effects of sex hormones on nociceptive thresholds - found to be substantially smaller in female than male. Sex hormones play a key role in contributing to gender differences.<br><br>•unclear demographics (age of the animals).<br><br>•unclear sample size.<br><br>•most likely, adequate timing of disease induction - before allocation of the intervention	? ii	√<br>•all rats were housed in climate-controlled rooms on a 12/12 light/dark cycle with water and standardized rodent diet available ad libitum.<br><br>•The experimental procedures were approved by the Commission of Zhengzhou University for ethics of experiments on animals in accordance with international standards.	√<br>•testers were not informed as to which rats had CCI or sham surgery.	?<br>•unclear if animals were selected at random for outcome assessment.<br><br>•same time points for objective outcome assessment, all mice were sacrificed for tissue section preparation – “at the designated time”	? ii<br>•unclear if outcome assessors (for biomarkers) were blinded.<br><br>•outcome assessment methods were the same in control and treatment groups – objective measures.	?<br>•unclear information regarding total sample size. Thus, unclear if all animals were included in the analysis (n=6/group) or if any missing outcome data.	√ iii	?<br>•supported by a grant from National Natural Science Foundation of China and the Youth Innovation Fund of the First Affiliated Hospital of the Zhengzhou University.<br><br>•ION-CCI model is widely accepted as an appropriate model of trigeminal neuralgia - reproduces important aspects of TN, including signs of abnormal spontaneous pain-related behaviour and mechanical allodynia.
Yesudhas, 2021 [34]	? i	X	? ii	√<br>•mice were maintained in the	?<br>•unclear, if the caregivers and/or	?<br>•unclear if animals were	? ii<br>•unclear if outcome	√	√ iii	?<br>•supported by a research grant

		•balanced baseline characteristics: ageing male BALB/c mice 7-8 months-old<br><br>•only male mice (uneven gender distribution) - many studies have found that antidepressant therapies in the female mouse models of depression are affected by the estrus cycle and estrogen.<br><br>•balanced sample size.<br><br>•unclear, experimental ageing model? Innate anxiety? no disease induction before allocation of the intervention.		animal house facility of Bharathidasan University in the standard 12-h light/dark cycle with free access to food and water.<br><br>• all experiments were conducted in accordance with the approval of the Institutional Animal Ethics Committee (IAEC) under the regulation of the Committee for the Purpose of Control and Supervision of Experiments on Animals (CPCSEA), India.	investigators were blinded from knowledge which intervention each animal received during the experiment.<br><br>•circumstance for assessments were the same in treatment groups and control – timing for assessments - 30 days after Tx (1st behavioural tests and then animals were sacrificed - hippocampi of brains were dissected out)	selected at random for outcome assessment.<br><br>•same time points for objective outcome assessment, all mice were sacrificed for tissue section preparation – after behavioural tests	assessors (for biomarkers) were blinded.<br><br>•outcome assessment methods were the same in control and treatment groups – objective measures.	•all animals included for analysis.<br><br>•balanced in numbers across intervention groups.		from the Science and Engineering Research Board (SERB), the Faculty Recharge Programme, University Grants Commission (UGC-FRP), New Delhi, India. financial assistance from an Early Career Research Award from SERB, RUSA 2.0, Biological Sciences, Bharathidasan University, and UGC-SAP and DST-FIST for the infrastructure of the Department of Animal Science, Bharathidasan University.
Kim, 2015 [26]	? i	?<br>•balanced baseline characteristics: male Sprague-Dawley rats (230-280g).<br><br>•only male mice (uneven gender distribution) excluded the effects of sex hormones on nociceptive thresholds - found to be substantially smaller in female than male.	? ii	√<br>•rats were maintained at a constant temperature and under a standard 12 h/12 h light/dark cycle. Food and water were freely available.	√<br>•all experiments were performed in a blinded manner	?<br>•unclear if animals were selected at random for outcome assessment.<br><br>•unclear time points for objective outcome	? ii<br>•unclear if outcome assessors were blinded.<br><br>•outcome assessment methods were different among different	?<br>•unclear information regarding total sample size. Thus, unclear if all animals were included in the analysis (n=5 or 8 /group) or if	√ iii	?<br>•the features of formalin-induced inflammatory pain model are inconsistent with those of TN. In addition, BoNT pretreatment method is not a good clinical

		Sex hormones play a key role in contributing to gender differences.<br><br>•unclear demographics (age of the animals).<br><br>•unclear total sample size.<br><br>•unclear adequate timing of disease induction - before allocation of the intervention.		•all procedures including the use of animals were approved by the Institutional Care and Use Committee of the School of Dentistry, Kyungpook National University (Daegu, Korea). Experiments were carried out in accordance with the ethical guidelines for the investigation of experimental pain in conscious animals issued by the International Association for the Study of Pain (1982).		assessment (c-fos)	orofacial pain models – objective measure.	any missing outcome data.<br><br>•unbalanced and different sample sizes for statistical analysis according to each outcome.		simulation of BoNT treatment for TN.<br><br>• design-specific risks of bias: objective outcome – c-fos expression only assessed in 1 of 3 orofacial pain models (NMDA).<br><br>•supported by the National Research Foundation of Korea (NRF) and funded by the Ministry of Science, ICT & Future Planning and by Hugel Inc.
Xiong, 2023 [30]	√ i	√<br>•balanced baseline characteristics: New Zealand white female rabbits (age: 6 months, 3.0 ~ 3.3 kg)<br><br>•only female (uneven gender distribution)<br><br>•balanced sample size.<br><br>•adequate timing of disease induction - before allocation of the intervention.	? ii	√<br>•rabbits were housed in separate cages under standard conditions.<br><br>•study was approved by the Laboratory Animal Ethics Committee of the Nanchang University School of Medicine. The animal experimental procedures strictly followed the guidelines of the	?<br>•unclear, if the caregivers were blinded from knowledge which intervention each animal received during the experiment.<br><br>•circumstance for assessments were the same in treatment groups and control – timing for assessments, but	?<br>•unclear if animals were selected at random for outcome assessment.<br><br>•same time points for objective outcome assessment.	√<br>•histological experiments were performed by researchers blinded to the experimental procedures.	√<br>•all animals included for analysis.<br><br>•balanced in numbers across intervention groups.	√ iii	?<br>•there is a significant difference between rabbit ears and human skin; therefore, the rabbit ear model cannot completely represent human HS.<br><br>•supported by a grant from the Science and Technology Research Project,

				Guide for the Care and Use of Laboratory Animals published by the National Institutes of Health.	fractional CO2 laser and BoNT injection methods of administration differ.					Jiangxi Provincial Department of Education
Cao, 2017 [33]	? i	?<br>•balanced baseline characteristics: male CD1 (ICR) mice 6-8 weeks old.<br><br>•only male (uneven gender distribution)<br><br>•unclear if balanced sample size among groups.<br><br>•likely, but unclear if adequate timing of disease induction - before allocation of the intervention.	? ii	√<br>•all mice were maintained on room temperature (~22±2◦C) and humidity (60-80%), with a light cycle of 12h light/ 12h dark, and with enough food and water available ad libitum.<br><br>•study was approved by the Animal Use and Ethics Committee of the Soochow University. The animal experimental procedures strictly followed the guidelines of the IASP.	√<br>•animal experiments were conducted in a blinded manner with respect to drug Tx.<br><br>•behavioural studies were carried out by experimenter unaware of the Tx.<br><br>•animal experiments were conducted in a sound-attenuated cabin between 9:00am and 16:00pm.	?<br>•unclear if animals were selected at random for outcome assessment.<br><br>•different outcomes (objective TRPV1 levels) time points for outcome assessment according to type of model.<br><br>•cervical DRG´s were collected following injection BoNT (0.1U) at different time points.	√<br>•scratching behaviour was quantified in a blinded manner.	?<br>•unclear information regarding sample size among groups. Thus, unclear if all animals (n=525) were included in the analysis or if any missing outcome data.<br><br>•unbalanced and different sample sizes for statistical analysis according to each outcome.<br><br>•expression level of TRPV1 in DRG in AEW model – data not shown	√ iii	?<br>•supported by grants from the NSFC (National Natural Science Foundation of China), from the Nature Science Foundation of Jiangsu province, from Second Affiliated Hospital of Soochow University Preponderant Clinic Discipline Group Project Funding, and A Project Funded by the Priority Academic Program Development of Jiangsu Higher Education Institutions.

**Legend: BoNT**, botulinum toxin; **Tx**, treatment; **AO**, osteoarthritis; **TN**, trigeminal neuralgia; **PD**, Parkinson's disease; **WIM**, whisker intrinsic musculature; **IoNC/ION-CCI**, chronic constriction injury of the infraorbital nerve; **CRS**, chronic restraint stress; **POD**, postoperative day; **IASP**, International Association for the Study of Pain; **PAPD**, Priority Academic Program Development; **IAEC**, Institutional Animal Ethics Committee; **CPCSEA**, Committee for the Purpose of Control and Supervision of Experiments on Animals; **NSFC**, National Natural Science Foundation of China; **DNI**, dural neurogenic inflammation; **RIA**, radioimmunoassay; **TNR**, trigeminal nerve root; **CGRP**, calcitonin gene related peptide; **mBSA**, methylated bovine serum albumin; **CFA**, Complete Freund's Adjuvant; **HS**, hypertrophic scar; **CFA**, Complete Freund's Adjuvant; **BDNF**, brain derived neurotrophic factor; **p-ERK**, phosphorylated extracellular signal-regulated kinase; **p-CREB**, cAMP response element binding protein; **SRS**; spatial restraint stress; **NMDAR**, N-methyl-D-aspartate receptor; **RT-PCR**, reverse transcription-polymerase chain reaction; **TLRs** - toll-like receptors; **TNC**, trigeminal nucleus caudalis; **SNARE**, soluble N-ethylmaleimide-sensitive-factor attachment protein receptor; **SNAP-25**, synaptosomal-associated protein-25; **HIF-1α**, hypoxia-inducible factor; **TRPV4**, protein expression of transient receptor potential vanilloid type 4; **TRPM3**, transient receptor potential melastatin 3; **TRPV1,** transient receptor potential vanilloid type 1; **TRPA1**, transient receptor potential ankyrin 1; **DRG**, dorsal rot ganglia; **AEW**, induced by acetone-diethylether-water.
*Items in agreement with the items in the Cochrane Risk of Bias tool.
A "yes" judgement indicates a low risk of bias; a "no" judgment indicates high risk of bias; the judgment will be "unclear" if insufficient details have been reported to assess the risk of bias properly. It is not recommend calculating a summary score for each individual study when using this tool.
**Key: √**, yes; **X**, no. **?,** unclear. **i**, *the investigators did not describe the method used for a random component in the sequence generation process.* **ii**, *it is unlikely that the outcome or the outcome measurement was influenced by this domain, given the objective measurements.* **iii**, *the study protocol was not available, but it was clear that the published report included all expected outcomes (i.e. comparing methods and results section).* **iv**, *single blind.*
