# Supplementary material for: Botulinum Toxin Effects on Biochemical Biomarkers Related to Inflammation-Associated Head and Neck Chronic Conditions: A Systematic Review of Preclinical Research
Source: Toxins (Basel). 2025 Jul 29;17(8):377. doi: 10.3390/toxins17080377 (PMC12390450; doi:10.3390/toxins17080377)
Supplement: Supplementary file 1 [file toxins-17-00377-s001.zip › SR2. file S5. Table S4. Biomarkers_single experiments.pdf]

File S5. Table S4. Biomarkers in Preclinical Research (single experiments). Botulinum Toxin (BoNT) effects in Head & Neck (H&N) Chronic Inflammatory State (CIS)

Biomarkers	CIS	Author Year	Biological sampling	Biomarker vs. BoNT	Biomarker vs. CIS	H&N clinical trials ?
1 STUDY						
IBA-1<br>MICROGLIA MARKER (potential marker for monocyte/macrophage lineage)	TN	Chen, 2021 [14]	TNC	BoNT may supress the activation of microglia in the CNS.	Microglia (resident immune cells in the CNS) play a critical role in central sensitization and plasticity.	Post-mortem brain samples, not in BoNT context.
VGAT<br>PRESYNAPTIC VESICLE PROTEINS (INHIBITORY) - MARKER FOR GABAERGIC NEURONS<br>VGAT/ Gephyrin<br>Gephyrin<br>PSD95<br>SYNAPTIC MARKER<br>VGlut2/ PSD95<br>VGlut2/ IBA-1<br>(NEUROPLASTICITY-RELATED)<br>VGlut2<br>PRESYNAPTIC VESICLE PROTEINS (EXCITATORY) - MARKER FOR GLUTAMATERGIC NEURONS	Depression - PD	Li, 2023 [18]	Brain- SNpc & hippocampus	BoNT may inhibit the release of neurotransmitter, including glutamate and GABA (and their vesicular transporters), acting on PNS and CNS.<br>BoNT may attenuate the microglial engulfment of presynaptic synapses (improving the apparent synapse and spine loss in the hippocampus)	Potential activities of GABAergic neurons are involved in depression in PD.<br>Synaptic density of colocalized puncta of VGAT/Gephyrin at inhibitory synapses was used to test if synapse loss occurs in the hippocampal CA1 region (associated with hippocampal atrophy) in depression in PD, and with BoNT. (Gephyrin is a central GABAergic synapse organizer).<br>PSD95 primarily regulates the differentiation of excitatory synapses at the postsynaptic density.<br>Synaptic density of colocalized puncta of VGlut2/PSD95 at excitatory synapses was measured to test if synapse loss occurs in the hippocampal CA1 region (associated with hippocampal atrophy) in depression in PD, and with BoNT.<br>VGlut2/IBA-1 was used to test microglial engulfment of synapses in depression in PD, and with BoNT,<br>Potential activities of glutamatergic neurons are involved in depression in PD, indicating modulators of glutamate signalling as a novel potential drug.	* A chronic migraine clinical trial with BoNT evaluted genes of GABA system - GABRE, GABRQ, GABRA3, and genes related to glutamate homeostasis – MEF2D, LRP1, MTDH, EAAT2, GRIK3

TH<br>ENZYME RESPONSIBLE FOR DOPAMINE SYNTHESIS				TH was used to explore if BoNT impacts neurochemical features in PD	Reduced TH levels and TH+ neurons in both the striatum and SNpc have been associated with depression, suggesting a potential role for dopamine dysfunction.	√<br>Not in BoNT context.
C3<br>C1q<br>C3aR<br>COMPLEMENT PROTEINS AND THEIR REGULATORS				BoNT may suppress the activation of microglia in the CNS and have effects on complement-mediated microglial synaptic engulfment and microglia-mediated neuroinflammation.<br>BoNT may impact the activation of complement and microglia via interaction with receptors on microglia.	Interplay between microglia-complement-neurological disorders has been associated to neuroinflammation and complement-mediated microglial synaptic engulfment. Complement proteins and their regulators are involved in synapse elimination (neurodevelopment) and synapse loss (neurodegeneration process).	√<br>Peripheral plasma concentration in MDD, but not in BoNT context.
Fractalkine (CX3CL1)/ CX3CR1					CX3CL1/CX3CR1 complement pathway - implicated in microglia activation and microglia-mediated neuronal remodelling.	?<br>e.g., serum levels of patients with systemic sclerosis, but not in the context of BoNT-microglia-neuronal cross-talks
CD68/IBA-1<br>LYSOSOME/MICROGLIA MARKER					CD68 is a lysosomal-associated protein in microglia in the brain and is associated with phagocytic cells. CD68/IBA-1 was used to define microglial activation	Post-mortem brain samples, not in BoNT context
MMP-13<br>COLLAGENASE	TMJ OA	Makawi, 2022 [13]	TMJ tissues	The specific mechanism by which BoNT influences MMP13 expression is still under investigation	MMP-13 is considered one of the most common enzymes studied for cartilage degradation, due to its capacity in cleaving collagen type II (major component of articular cartilage)	√<br>TMJ disc samples, not in BoNT context.
BDNF<br>NEUROMODULATOR (NEUROPLASTICITY)	Depression	Li, 2019 [21]	Hippocampus, hypothalamus, prefrontal córtex,	BoNT effect in depression prevailing theories: facial and social feedback, structural or functional changes in the brain - upregulation of BDNF and monoamines, insula cortex alteration and modulation effects on neuroinflammation.	Neuroinflammation and neurotrophic factors may contribute to the pathophysiology of depression. BDNF level is low in animal models of depression and depressed patients, and antidepressants target the production of BDNF. BDNF plays a critical role in synaptic plasticity mainly via activation of NMDARs	√<br>Serum and urine levels in multiple sclerosis and neurogenic detrusor overactivity.

				amígdala (Brain)		and promotes CREB phosphorylation through ERK activation.	
NR1	NMDAR SUBUNITS<br><br>NEUROTRANSMITTER (GLUTAMATE) RECEPTORS				BoNT may inhibit the translocation of neurotransmitter receptors to the neural cell membrane.<br>The expression of the phosphorylated NR2B subunit is reduced by peripherally administered BoNT, possibly mediated by decreased glutamate release from presynaptic primary afferent terminals rather than the direct effects in postsynaptic neurons.	NMDAR binds glutamate subunits and has been implicated in the pathogenesis of depression (the distinct roles of these subunits are unclear and may depend on the animal models used. Phosphorylation of NMDARs has shown to cause long-term synaptic plasticity related to central sensitization.	√<br>e.g., in patients with schizophrenia, but not in the context of BoNT.
NR2A							?
NR2B							√<br>NR2B receptor antagonists TXT-0300 (to treat CNS diseases, mental and pain diseases, but not in BoNT context
5-HT<br>NEUROTRANSMITTER					BoNT blocks neurotransmitter release and leads to build-up of synaptic vesicles near the presynaptic membrane.	5-HT – important neurotransmitter in synaptic transmission. Serotoninergic dysfunctions or reduced 5-HT levels (brain) contribute to the pathogenesis of depression in animal models and patients	√
p-ERK	PROTEIN KINASE				BoNT may activate BDNF-ERK-CREB signalling pathways.	The intracellular ERK-CREB pathway is important in neurotrophins signalling and neurogenesis (involved in the pathogenesis of depression). BDNF-ERK-CREB plays a vital role in modulation of neuronal function, synaptic plasticity of neurons, cell survival, cell apoptosis, learning, and long-term potentiation.	?<br>*clinical trials ERK1/2 in advanced solid tumor, not in BoNT context nor ERK-CREB pathway.
p-CREB	TRANSCRIPTION FACTOR (NEUROPLASTICITY)						
TLR1	TOLL-LIKE RECEPTORS (TLRS) normally expressed in immune and glial cells to regulate innate and adaptive immunity	TN	Chen, 2021 [14]	TNC	BoNT may supress the activation of microglia after nerve injury in the CNS, inhibiting the overexpression of microglia-derived pro-inflammatory factors and neuroinflammation in the central nerve system (e.g., via suppression of TLR2/myD88 signalling and TLR2-mediated neuroinflammation).	Glia activation has been implicated in the development of central sensitization through microglial mediators (e.g., cytokines, PGE2 and BDNF), which regulate inhibitory synaptic transmission in spinal cord dorsal horn neurons through presynaptic, postsynaptic and/or extrasynaptic mechanisms, and contributes to the pathogenesis of neuropathic pain.	√<br>*clinical trials evaluated expression of inflammatory genes (TLR signalling and TLR regulatory)
TLR2<br>MICROGLIA MARKER							
TLR4							
TLR5							
TLR8							

TLR11	(NEUROINFLAMMATION & NEUROPLASTICITY-RELATED)					TLR2 may be involved in the pathogenesis of neuropathic pain models. It is a reliable marker of activated microglia in vivo.	
MyD88	DOWNSTREAM MOLECULE OF TLRS					CD11b detects morphological changes of microglia	X
CD11b	MICROGLIA MARKER						Cultured human microglia from discarded brain tissue
F4/80	MACROPHAGE OR MICROGLIA MARKER						X
IL-4		Atopic Dermatitis	Han, 2017 [27]	Rostral dorsal Skin	BoNT was reported to have antipruritic, anti-inflammatory, and antiallergic actions to treat histamine-induced itch, lichen simplex chronicus, allergic rhinitis, and psoriasis.<br>BoNT inhibits the release of ACh, neuropeptides, and VIP from nerve endings in response to stimuli, suppression in eosinophil infiltration and capillary dilation, improving pruritus and neurogenic inflammation.	Cross-talks between secreted factors, inflammatory cells, resident skin cells and neural networks has been implicated in atopic dermatitis and itch. IL-4 has key role in the inflammatory response, skin barrier dysfunction, and itch sensation in atopic dermatitis.<br>Mast cells can contribute to the symptoms of itching, redness, and inflammation by releasing various mediators.<br>ACh, neuropeptides and VIP mediates inflammation and itch in atopic dermatitis.	√<br>* IL-4 and IgE plasma levels were measured in patients with atopic dermatitis, but not in the context of BoNT
Mast cell							
IgE				Serum - retro orbital plexus			
GFAP<br>PROTEIN/ASTROCYTES MARKER & MARKER OF ASTROGLIOSIS		PIH & TMJ arthritis	Muñoz-Lora, 2022 [22]	TNC	BoNT may suppress the activation of microglia in the CNS and thus the levels of GFAP	GFAP levels in the cerebrospinal fluid (CSF) and blood have been proposed as biomarkers for certain neurological disorders. Astrocytes represent most glial cells with a central role in the persistence of pain.	√<br>Plasma GFAP in Alzheimer's, not BoNT context
Glutamate<br>NEUROTRANSMITTER		PIH & TMJ arthritis	Muñoz-Lora, 2017 [15]	Peri-articular tissues (TMJ) & TG	BoNT may block the release of neurotransmitters from peripheral C-fibre nerve endings and in TG (potentially in the central terminals of the peripheral sensory neurons - primary afferents)	Glutamate - considered one of the principal excitatory neurotransmitters involved in central (higher levels) and PNS. Within peripheral tissues is pro-nociceptive and implicated in the development and progression of pain.	√

P2X7	MICROGLIAL PURINERGIC RECEPTOR	PIH & TMJ arthritis	Muñoz-Lora, 2020 [16]	TNC	BoNT may have effects on microglia-activated pathways through the reduction of microglia modulators, leading to the reduction of pro-inflammatory cytokines.<br>Peripherally administered BoNT may directly influence microglial function by transcytotically transport to microglia or from the modulation of cytokines or purinergic signalling in primary afferents.	P2X7 receptor/Cathepsin S (CatS)/Fractalkine (FKN) microglia-activated pathway signals antigen-induced arthritis of the TMJ.<br>P2X7 - implicated in microglia-mediated neuronal remodelling. There is a relation between P2X7 receptor activation and CatS upregulation and cleavage of FKN from neurons that engages the CX3CR1 receptor back in microglia. These processes stimulate communications between glial cells and neurons.	?<br>In chronic migraine, report for genes which encode voltage dependent channels – P2RX7
Cathepsin S (CatS)	MICROGLIAL-NEURON MODULATORS						?<br>Current CatS inhibitors in clinical trials (mostly for autoimmune diseases cancer) face challenges
Fractalkine (FKN)							√<br>anti-FKN monoclonal antibodies for Reumathic arthritis, not in BoNT context.
IB4 (+) and IB4 (-) MARKER SUBSET OF SENSORY NEURONS / FM4-64 MEMBRANE-UPTAKE MARKER (VESICULAR TRANSMITTER RELEASE)		TN	Kitamura, 2009 [29]	TRG sensory neurons	Peripheral injection of BoNT decreases the exaggerated neurotransmitter release in peripheral sensory neurons (BoNT effects on the sensory ganglia).	Atypical neurotransmitter release and signalling within the trigeminal nerve pathway has been linked to TN.	Not directly translatable to human clinical settings.
Inflammatory cells: lymphocyte, monocyte, neutrophil, plasma cells (NEUROGENIC INFLAMMATION & NEUROINFLAMMATION)		Trigeminal pain - TMDs (inflammatory pain)	Lacković, 2016 [20]	Cranial dura tissue	BoNT may inhibit neurogenic inflammation by cleaving SNAP25 and blocking the release of neurotransmitters from peripheral C-fibre nerve endings and trigeminal ganglion. It may also play a role on modulation of the development of neuroinflammation by inhibiting the overexpression of microglia-derived pro-inflammatory factors.	Potentially, inflammatory process characterized by local neurogenic inflammation and neuroinflammation, in the pathophysiology of TN. Neurogenic inflammation is triggered by nerve activation and results in neuropeptide release and rapid plasma extravasation. Neuroinflammation is a localized inflammation in the peripheral and central nervous system, which results in the activation of glial cells in DRG, spinal cord, and brain.	√
Fibroblasts		HS	Wang, 2020 [25]	Scar Tissue (ear)	BoNT may improve hypertrophic scars by reducing muscle tensions and act on the biological behaviour of fibroblasts (e.g.,	The main cell to induce wound contraction and scar formation is the fibroblast.	Experiments on cultured cells-derived from

					induce fibroblast apoptosis, which can inhibit fibroblasts contraction).		humans with the condition
HIF-1α	TRANSCRIPTOR FACTOR - REGULATOR OF HYPOXIA-INDUCED CELLULAR RESPONSES (ASSOCIATED TO PERYPHERAL INFLAMMATION)	TN	Cho, 2022 [17]	TG	BoNT may regulate HIF-1α associated cytokine pathway in the TG (through the primary afferent fibers)	HIF-1α higher levels in hypoxia, ischemia, and inflammatory states. Originates proinflammatory cytokines that maintain the inflammatory processes. Potential role in the development of TN. It is crucial in vascular remodelling, erythropoiesis, angiogenesis, cell proliferation, and wound healing.	√ *Blood plasma levels of HIF-1α in systemic sclerosis, but not in BoNT context.
Nav 1.3<br>Nav 1.6<br>Nav 1.7<br>Nav 1.8	VOLTAGE-GATED SODIUM CHANNELS (Navs) α-SUBUNITS TRANSMEMBRANE PROTEIN COMPLEXES (REGULATION OF ION CHANNELS)	TN	Yang, 2016 [31]	TG (mandibular (V3) division and boundary area)	The underlying cellular mechanisms for BoNT antinociceptive effects may be mediated by the modulation of expression of certain peripheral Navs α-subunits levels in sensory ganglia, via primary afferent fibers of the trigeminal nerve rather than systemic action.	Navs α-subunits exhibit distinct expression patterns, electrophysiologic and pharmacologic properties. Tissue and nerve damage interferes with specific Nav α-subunits, which contribute to modulating the excitability of most neurons (e.g., nociceptive sensory signalling, underlying chronic pain conditions).	Experiments using induced pluripotent stem cells from human with hereditary neuropathic pain syndrome
ATF3	PROTEIN - MARKER OF NEURONAL INJURY (MOLECULAR MARKER FOR INJURED DORSAL ROOT GANGLION SENSORY NEURONS IN THE PNS)				BoNT may produce prolonged antinociceptive effects before and after pain has been established.	Nerve injury results in the development of neuropathic pain.	√ * Serum ATF3 levels after SCI or ischemic stroke, but not in the context of BoNT.
TRPM3<br>TRPV4	PERMEABLE NONSELECTIVE CATION CHANNEL (NEUROPLASTICITY)	TN	Zhang, 2019 [32]	Trigeminal spinal subnucleus caudalis	BoNT key mechanisms may include the modulation of expression of certain receptors or ion channels in nociceptor cell membranes. BoNT potential effects on sensory ganglia includes blockage of translocation of TRPV1 to the sensory neuronal surface in the ganglia. BoNT central effects involve retrograde axonal transport to the central nervous system from the periphery, potentially mediated	TRPs have been implicated in the perception of pain, inflammation and detecting noxious stimuli. TRPV1 localized in the central terminals of trigeminal afferents within the trigeminal nucleus caudalis has a role in perpetuating neuropathic pain. TRPs may contribute to neuronal excitability and potentially in neurological conditions.	√ In chronic migraine, genes of the TRP family – TRPV1, TRPV3, TRPM8
TRPV1	HEAT- AND CAPSAICIN-SENSITIVE ION CHANNEL	TN	Wu, 2016 [23]	Brainstem Vc region (caudal subnucleus of			
TRPV2	CALCIUM-PERMEABLE CATION CHANNEL						

TRPM8	COLD-ACTIVATED ION CHANNEL			the spinal TNuc)	by TRPV1-expressing sensory neurons (expressing central afferent terminals) or other neuronal pathways.		
SOD<br>Catalase<br>GSH<br>GPx	ANTIOXIDANT ENZYMES (NEUROPROTECTION & NEUROPLASTICITY-RELATED)	Anxiety & ageing	Yesudhas, 2021 [34]	Hippocampus (brain) tissues – total protein isolates	BoNT may have anxiolytic effects by estimating the enzymatic activities of key antioxidants properties (neuroprotection). Mechanisms underlying BoNT enzymatic effects are unclear.	Associations to anxiety: 1. Ageing and various neuropathogenic conditions associated with the abnormal discharge of neurotransmitters (increased levels of ACh have been associated with adult-onset neurological and mood disorders including anxiety). 2. The vulnerability of the hippocampus to cellular oxidative stress.<br>Hippocampal plasticity may contribute to the regulation of mood and key antioxidant enzymes in the hippocampus are important for neuroprotection by neutralizing harmful free radicals.	?<br>*Other oxidative stress markers were used in the plasma of patients with chronic migraine.

LEGEND: **CIS,** chronic inflammatory state; **BoNT,** botulinum toxin; **CNS,** central nervous system; **PNS,** peripheral nervous system**; MMP-13,** matrix metallopeptidase; **TG,** trigeminal ganglia; **PD,** Parkinson Disease; **TNC,** trigeminal nucleus caudalis; **TMD,** temporomandibular disorder; **TMJ,** temporomandibular joint; **OA,** osteoarthritis; **TN,** trigeminal neuralgia; **TNuc,** trigeminal nucleus; **PIH,** persistent immunogenic hypersensitivity; **IL,** interleukin; **SNpc,** substantia nigra pars compacta; **vlPAG,** ventrolateral periaqueductal gray; **BDNF,** brain derived neurotrophic factor; **(cl)SNAP-25,** (cleaved) synaptosomal-associated protein-25; **ChAT,** choline acetyltransferase; **PSD95,** postsynaptic density-95; **NMDAR**, N-methyl-D-aspartate receptor; **5-HT,** 5-hydroxytryptamine; **Vc,** caudal subnucleus of the spinal trigeminal nucleus; **p-ERK**, phosphorylated extracellular signal-regulated kinase; **p-CREB**, cAMP response element binding protein; **Iba-1**; ionized calcium-binding adaptor molecule 1; **Myd88,** myeloid differentiation factor; **TLRs** - toll-like receptors; **GFPA**, glial fibrillary acidic protein; **DNI**, dural neurogenic inflammation; **IgE**, immunoglobulin E; **CX3CR1,** CX3 chemokine receptor 1; **IB4**, isolectin B4-binding; **HIF-1α**, hypoxia-inducible factor; **TH**, tyrosine hydroxylase, dopaminergic neuronal marker; **VGlut2**, vesicular glutamate transporter 2; **VGAT**, vesicular GABA transporter; **SOD**, superoxide dismutase; **GSH**, glutathione; **GPx,** glutathione peroxidase; **DRG**; dorsal root ganglia; **ATF3**, activating transcription factor 3; **TRPV4**, protein expression of transient receptor potential vanilloid type 4; **TRPM**, transient receptor potential melastatin; **TRPV**, transient receptor potential vanilloid type; **Ach**, acetylcholine; **SCI,** spinal cord injury; **VIP,** vasoactive intestinal peptide.
