# Supplementary material for: Botulinum Toxin Effects on Biochemical Biomarkers Related to Inflammation-Associated Head and Neck Chronic Conditions: A Systematic Review of Preclinical Research
Source: Toxins (Basel). 2025 Jul 29;17(8):377. doi: 10.3390/toxins17080377 (PMC12390450; doi:10.3390/toxins17080377)
Supplement: Supplementary file 1 [file toxins-17-00377-s001.zip › SR2. file S6. Table S5. GRADE_single experiments.pdf]

File S6. Table S5. Biomarkers in Preclinical Research (Single experiments) on Botulinum Toxin effects in Chronic Inflammatory State. Summary of outcomes. GRADE consistency assessment.

Biomarker (CIS)	Biological sampling	Author Year	Unit measure	BoNT Key effect (Outcome)	Summary	Overall GRADE
**1 STUDY**						
				**TNC**		
IBA-1	TN	Chen, 2021 [14]	mRNA; protein expression	• (n=12) TN vs (n=12) sham: (B) - ip.l t4 = 2.949, (↑) P=0.0420; cl. t4 = 4.332, NS<br>• (n=12) TN+BoNT (0.18U) vs (n=18) TN+vehicle: (T1) ip.l, (↓) - NR	1 RCT (n=48), mean ± SEM<br>(T1) 5 days after BoNT (↓)	LOW ⊕⊕○○
				**Brain- SNpc & hippocampus**		
fractalkine/ CX3CL1	Depression - PD	Li, 2023 [18]	mRNA expression	• reserpine vs sham: (B) (X) no changes - NR<br>• reserpine+BoNT: (T1) (X) F (3, 16) = 2.236, P = 0.1235 in hippocampus	1 ? (n=?) mean ± SEM<br>(T1) ? (X)	VERY LOW ⊕○○○
VGAT			Scale bar=5 µm	• reserpine vs sham: (B) (NS) - NR<br>• reserpine+BoNT: (T1) (NS) F (3, 20) = 1.191, P=0.3384 hippocampal CA1	1 ? (n=?) mean ± SEM<br>(T1) ? (NS)	VERY LOW ⊕○○○
VGAT/ Gephyrin			synaptic density	• reserpine vs sham: (B) (NS) - NR<br>• reserpine+BoNT: (T1) (NS) F (3, 20) = 2.016, P=0.1440 hippocampal CA1	1 ? (n=?) mean ± SEM<br>(T1) ? (NS)	VERY LOW ⊕○○○
Gephyrin				• reserpine vs sham: (B) (NS) - NR<br>• reserpine+BoNT: (T1) (NS) F (3, 20) = 0.2680, P=0.8476	1 ? (n=?) mean ± SEM<br>(T1) ? (NS)	VERY LOW ⊕○○○
PSD95				• reserpine+BoNT: (T1) F (3, 20) = 7.603, P=0.0014	1 ? (n=?) mean ± SEM<br>(T1) ? (?)	VERY LOW ⊕○○○
VGlut2/ PSD95			Scale bar = 5 µm	• reserpine vs sham: (B) (↓) in hippocampus - NR<br>• reserpine+BoNT: (T1) (↑) F (3, 20) = 9.593, P=0.0004 in Hippocampal CA1 region	1 ? (n=?) mean ± SEM<br>(T1) ? (↑)	VERY LOW ⊕○○○
VGlut2/ IBA-1			Scale bar = 10 µm - %	• reserpine vs sham: (B) (↑) - NR<br>• reserpine+BoNT: (T1) (↓) F (3, 20) = 25.95, P<0.0001 in Hippocampal CA1 region	1 ? (n=?) mean ± SEM<br>(T1) ? (↓)	VERY LOW ⊕○○○
TH			protein levels	• reserpine vs sham: (B) (↓) F (3, 8) = 3.767, P = 0.0593 in SNpc & striatum<br>• reserpine+BoNT: (T1) (√) F (3, 8) = 4.316, P = 0.0436 in SNpc & striatum	1 ? (n=?) mean ± SEM<br>(T1) ? (√) NS	VERY LOW ⊕○○○
VGlut2			mRNA; protein expression	• reserpine vs sham: (B) (↓) in hippocampus - NR<br>• reserpine+BoNT: (T1) (↑) F (3, 20) = 7.739, P = 0.0013 in Hippocampal CA1 region	1 ? (n=?) mean ± SEM<br>(T1) ? (↑)	VERY LOW ⊕○○○
C3				• reserpine vs sham: (B) (↑) expression (a) protein, (b) mRNA & activation in hippocampus - NR<br>• reserpine+BoNT: (T1) (↓) (a) F (3, 8) = 34.09, P < 0.0001; (b) F (3, 20) = 8.045, P = 0.0010 in Hippocampal CA1 region; & activation P = 0.0059- NR	1 ? (n=?) mean ± SEM<br>(T1) ? (↓)	VERY LOW ⊕○○○
C1q				• reserpine vs sham: (B) expression (a) protein (↑), (b) mRNA (NS) & activation (↑) in hippocampus - NR<br>• reserpine+BoNT: (T1) (a) (↓) F (3, 8) = 29.37, P = 0.0001; (b) (NS) F (3, 20) = 1.978, P = 0.1498 in Hippocampal CA1 region; & activation (↓) P < 0.0001 - NR	1 ? (n=?) mean ± SEM<br>(T1) ? (↓) protein, (NS) mRNA	VERY LOW ⊕○○○

**C3aR**				• reserpine vs sham: (B) (↑) - NR<br>• reserpine+BoNT: (T1) (↓) F (3, 16) = 4.338, P = 0.0204	1 ? (n=?) mean ± SEM<br>(T1) ? (↓)	VERY LOW ⊕○○○
**CD68/IBA-1**			%	• reserpine vs sham: (B) (↑) in hippocampus - NR<br>• reserpine+BoNT: (T1) (↓) F (3, 20) = 27.82, P<0.0001, in hippocampus	1 ? (n=?) mean ± SEM<br>(T1) ? (↓)	VERY LOW ⊕○○○
				**TMJ tissues**		
**MMP-13**	**TMJ OA**	**Makawi, 2022 [13]**	protein expresson, pg/ml tissue	• (n=3) sham vs (n=2/group) OA: (T1, T2) 1.26E(m), 0.10(SD) vs 3.55B(m), 0.25(SD)<br>• (n=14) OA+BoNT (5U/kg): (T1) ipl. (↓) p<0.001, 2.24C(m), 0.13(SD); cl. 4.16A(m), 0.75(SD)<br>• (n=14) OA+BoNT (5U/kg): (T2) ipl. (↓) p<0.001, 1.61F(m), 0.06(SD); cl. 6.17B(m), 0.05(SD)	1RCT (n=42), mean (m), SD<br>(T1) 2weeks after BoNT (↓)<br>(T2) 4 weeks after BoNT (↓)	LOW ⊕⊕○○
				**Hippocampus, hypothalamus, prefrontal córtex, amígdala (Brain)**		
**BDNF**	**Depression**	**Li, 2019 [21]**	mRNA; protein expression	• (n=5-6) SRS vs naïve ((a) protein / (b) mRNA): (B) (a) (↓) (t = 3.500), P = 0.0057 in hippocampus / (b) (↓) (t = 4.117), P = 0.0146 in hippocampus; (t = 4.129), P = 0.0145 in amigdala; (x) in hypothalamus & prefrontal cortex.<br>• naïve+BoNT vs naïve: (T1) (a) / (b) (X) - NR<br>• (n=6) SRS+BoNT: (a) (↑) F(5, 30) = 10.37, P<0.0001 / (b) (↑) F(4, 10) = 8.700, P = 0.0027 in hippocampus; (↑) F(4, 10) = 7.423, P = 0.0048 in amigdala; (↑) transiently F(4, 10) = 7.727, P = 0.0042 in prefrontal cortex; (↑) transiently F(4, 10) = 6.459, P = 0.0078 in hypothalamus.	1 nRT (n=?) mean ± SEM<br>(B) 1h, 1-,3-,7-days<br>(T1) 16-,18-,22-, 29-days after BoNT (↑)	VERY LOW ⊕○○○
**NR1**			by tubulin	• SRS vs naive: (B) (↓) (t = 5.102), P = 0.0005 in hippocampus<br>• naïve+BoNT vs naïve: (T1) (X) - NR<br>• (n=6) SRS+BoNT: (T1) (↑) F(5, 30) = 15.50, P<0.0001 in hippocampus	1 nRT (n=?) mean ± SEM<br>(B) 1h, 1-,3-,7-days<br>(T1) 16-,18-,22-, 29-days after BoNT (↑)	VERY LOW ⊕○○○
**NR2A**				• (n=6) SRS vs naive: (B) (X) (t =0.8309), P=0.4254 in hippocampus<br>• naïve+BoNT vs naïve: (T1) (↓) F(4, 25) = 11.74<br>• (n=6) SRS+BoNT: NR	1 nRT (n=?) mean ± SEM<br>(B) 1h, 1-,3-,7-days<br>(T1)16-,18-,22-,29-days after BoNT (NR)	VERY LOW ⊕○○○
**NR2B**				• SRS vs naive: (B) (↓) (t = 4.529), P = 0.0011 in hippocampus<br>• naïve+BoNT vs naïve: (T1) (X) - NR<br>• (n=6) SRS+BoNT: (T1) (↑) F(5, 30) = 4.758, P = 0.0026 in hippocampus	1 nRT (n=?) mean ± SEM<br>(B) 1h, 1-,3-,7-days<br>(T1) 16-,18-,22-, 29-days after BoNT (↑)	VERY LOW ⊕○○○
**5-HT**			ng/g	• (n=6-7) SRS vs naive: (B) (↓) (t = 3.383), P = 0.0277 in hippocampus, (↓) (t = 6.596), P = 0.0027 in hypothalamus, (X) in prefrontal cortex – NR<br>• (n=6-7) naïve+BoNT vs naïve: (T1) F(2, 15) = 3.811, P = 0.0459 in hippocampus, (X) in hypothalamus & prefrontal cortex.<br>• (n=6-7) SRS+BoNT: (T1) (↑) F(4, 10) = 21.46, P < 0.0001 in hippocampus, F(4, 10) = 12.29, P = 0.0007 in hypothalamus, F(4, 10) = 7.101, P = 0.0056 in prefrontal cortex.	1 nRT (n=?) mean ± SEM<br>(B) 1day<br>(T1) 16-,18-,22-, 29-days after BoNT (↑)	VERY LOW ⊕○○○
**p-ERK**			HPLC analysis, RT-PCR, western blotting (GAPDH)	• SRS vs naive: (B) (↓) (t = 5.350), P = 0.0003 in hippocampus<br>• naïve+BoNT vs naïve: (T1) (X) - NR<br>• (n=6) SRS+BoNT: (T1) (↑) F(5, 30) = 5.810, P = 0.0007 in hippocampus	1 nRT (n=?) mean ± SEM<br>(B) 1-, 3-,7-days<br>(T1) 16-,18-,22-, 29-days after BoNT (↑)	VERY LOW ⊕○○○
**p-CREB**				• SRS vs naive: (B) (↓) (t = 4.034), P = 0.0024 in hippocampus<br>• naïve+BoNT vs naïve: (T1) (X) - NR<br>• (n=6) SRS+BoNT: (T1) (↑) F(5, 30) = 8.619, P<0.0001 in hippocampus	1 nRT (n=?) mean ± SEM<br>(B) 1-, 3-,7-days<br>(T1) 16-,18-,22-, 29-days after BoNT (↑)	VERY LOW ⊕○○○
				**TNC**		

**TLR1**	**TN**	**Chen, 2021 [14]**	mRNA; protein expression	• (n=12) TN vs (n=12) sham: (B) - ip.l (X); cl. (X) - NR<br>• (n=12) TN+BoNT (0.18U) vs (n=18) TN+vehicle: (T1) (↓) ipl. t6 = 3.393, P=0.0146; cl. t6 = 6.614, P=0.0006	1 RCT (n=48), mean ± SEM<br>(T1) 5 days after BoNT (↓) ipl. /cl.	LOW ⊕⊕○○
**TLR2**				• (n=12) TN vs (n=12) sham: (B) - ip.l t6 = 6.307, (↑) P=0.0007; cl. (X) - NR<br>• (n=12) TN+BoNT (0.18U) vs (n=18) TN+vehicle: (T1) ip.l, t6 = 6.058 (↓) P=0.0009	1 RCT (n=48), mean ± SEM<br>(T1) 5 days after BoNT (↓) ipl.	LOW ⊕⊕○○
**TLR4**				• (n=12) TN vs (n=12) sham: (B) - ip.l (X); cl. (X) - NR<br>• (n=12) TN+BoNT (0.18U) vs (n=18) TN+vehicle: (T1) (↓) ipl. t6 = 8.721, P=0.0001; cl. t6 = 6.963, P=0.0004	1 RCT (n=48), mean ± SEM<br>(T1) 5 days after BoNT (↓) ipl. /cl.	LOW ⊕⊕○○
**TLR5**				• (n=12) TN vs (n=12) sham: (B) - ip.l t6 = 7.584, (↑) P=0.0003; cl. (X) - NR<br>• (n=12) TN+BoNT (0.18U) vs (n=18) TN+vehicle: (T1) ip.l, t6 = 6.897 (↓) P=0.0005	1 RCT (n=48), mean ± SEM<br>(T1) 5 days after BoNT (↓) ipl.	LOW ⊕⊕○○
**TLR8**				• (n=12) TN vs (n=12) sham: (B) - ip.l (X); cl. (X) - NR<br>• (n=12) TN+BoNT (0.18U) vs (n=18) TN+vehicle: (T1) (↓) ip.l, t6=5.766, P=0.0012: cl. t6 = 2.7, P=0.0356	1 RCT (n=48), mean ± SEM<br>(T1) 5 days after BoNT (↓) ipl. /cl.	LOW ⊕⊕○○
**TLR11**				• (n=12) TN vs (n=12) sham: (B) - ip.l t6 = 10.8 (↓) P < 0.0001; cl. (X) - NR<br>• (n=12) TN+BoNT (0.18U) vs (n=18) TN+vehicle: (T1) - NR	1 RCT (n=48), mean ± SEM<br>(T1) 5 days after BoNT - NR	LOW ⊕⊕○○
**MyD88**				• (n=12) TN vs (n=12) sham: (B) - ip.l t6 = 2.718 (↑) P=0.0347; cl. t6 = 0.9386 (X) P=0.3842<br>• (n=12) TN+BoNT (0.18U) vs (n=18) TN+vehicle: (T1) ipl. (↓) t6 = 2.654, P=0.0378; cl. (X) t6 = 0.5799, P=0.5831	1 RCT (n=48), mean ± SEM<br>(T1) 5 days after BoNT (↓) ipl.	LOW ⊕⊕○○
**CD11b**				• (n=12) TN vs (n=12) sham: (B) - ip.l t6 = 0.3945 (↑) P=0.0076; cl. (X) - NR<br>• (n=12) TN+BoNT (0.18U) vs (n=18) TN+vehicle: (T1) ipl. (↓) t6 = 4.994, P=0.0025; cl. (X) - NR	1 RCT (n=48), mean ± SEM<br>(T1) 5 days after BoNT (↓) ipl.	LOW ⊕⊕○○
**F4/80**				• (n=12) TN vs (n=12) sham: (B) - ip.l t6 = 2.965 (↑) P=0.0351; cl. (X) - NR<br>• (n=12) TN+BoNT (0.18U) vs (n=18) TN+vehicle: (T1) ipl. t6 = 4.799, (↓) P = 0.003; cl. (X) - NR	1 RCT (n=48), mean ± SEM<br>(T1) 5 days after BoNT (↓) ipl.	LOW ⊕⊕○○
**Rostral dorsal Skin**						
**IL-4**	**Atopic Dermatitis**	**Han, 2017 [27]**	mRNA; protein expression (ng/mL)	• (n=6) sham vs (n=6) TNCB (a) mRNA / (b) protein: (B) (a) 0.90 ± 0.36, p=0.000 / (b) 1.00±0.84, p=0.000<br>• (n=6) TNBC vs (n=6) sham (a) mRNA / (b) protein: (T1) (↑) (a) 174±44.9, p=0.000 / (b) 69.3±13.4, p=0.000<br>• (n=9) TNCB + BoNT (30U) vs control /vs TNCB: (T1) (↓) (a) 53.0±17.6, p=0.004 / p=0.000; (b) 20.2±4.84, p=0.44 / p=0.000<br>• (n=9) TNCB + BoNT (60U) vs control /vs TNCB: (T1) (↓) (a) 44.3±7.38, p=0.028 / p=0.000; (b) 17.4±5.72, p=0.148 / p=0.000	1 RCT (n=48), mean ± SEM<br>(T1) 14 days after 1st challenge (↓)	LOW ⊕⊕○○
**Mast cell**			count 5 high power fields	• (n=6) sham vs (n=6) TNCB: (B) 13.4±3.65, p=0.000<br>• (n=6) TNBC vs (n=6) sham: (T1) (↑) 85.3 ± 7.55, p=0.000<br>• (n=9) TNCB + BoNT (30U) vs control /vs TNCB: (T1) (↓) 28.1 ± 4.70, p=0.004 / p=0.000<br>• (n=9) TNCB + BoNT (60U) vs control /vs TNCB: (T1) (↓)17.7±2.69, p=1.000 / p=0.000	1 RCT (n=48), mean ± SEM<br>(T1) 14 days after 1st challenge (↓)	LOW ⊕⊕○○
**Serum - retro orbital plexus**						
**IgE**			ng/mL	• (n=6) sham vs (n=6) TNCB: (B) 2.93±1.76, p=0.000<br>• (n=6) TNBC vs (n=6) sham: (T1) (↑) 103±27.4, p=0.000<br>• (n=9) TNCB + BoNT (30U) vs control /vs TNCB: (T1) (NS) 68.7 ± 13.5, p=0.011 / p=0.765<br>• (n=9) TNCB + BoNT (60U) vs control /vs TNCB: (T1) (NS) 66.5±29.7, p=0.15 / p=0.573	1 RCT (n=48), mean ± SEM<br>(T1) 14 days after 1st challenge	LOW ⊕⊕○○
**TNC**						
**GFAP**	**PIH & TMJ arthritis**	**Muñoz-Lora, 2022 [22]**	surface area (μm²); mean grey intensity	• (n=10) PHI vs sham (a) area / (b) gray value: (B) (↑) ipl. (a) P < 0.001 / (b) P < 0.05; cl. - NR<br>• (n=10) PHI+BoNT vs PHI+vehicle: (T1) (↓) ipl. OnaBoNT.7U/kg (a) / (b) P < 0.001; AboBoNT.14U/kg (a) P < 0.001 / (b) P < 0.05; cl. – NR	1 RCT (n=40) mean±SEM<br>(T1) 14 days after BoNT (7U/14U) (↓) ipl	LOW ⊕⊕○○

				• (n=10) ($F_{3,16}$ (GFAP area) = 73.14; $F_{3,16}$ (GFAP gray value) = 23.21		
	**Peri-articular tissues (TMJ) & TG**					
**Glutamate**	**PIH & TMJ arthritis**	**Muñoz-Lora, 2017 [15]**	(nmol)	• PHI / PHI+vehicle vs sham: (B) NS P > 0.05- NR<br>• PIH+BoNT (7U/Kg) vs PHI/PHI+vehicle: (T1, T2) NS P > 0.05 - NR	1 ? (n=?) ?<br>(T1) 24h after BoNT (NS)<br>(T2) 14 days after BoNT (NS)	VERY LOW ⊕○○○
**P2X7**	**TNC**					
	**PIH & TMJ arthritis**	**Muñoz-Lora, 2020 [16]**	OD, protein level	• (n=8) PHI vs sham: (B) - (↑) P < 0.05 - NR<br>• (n=8) PHI+BoNT (7U/kg) vs PHI+vehicle: (T1) (↓) P < 0.05 - NR<br>• (n=8) PHI+BoNT (7U/kg) vs PHI+vehicle: (T2) (↓) P < 0.05 - NR<br>• (n=8) PHI+BoNT (7U/kg) vs PHI+vehicle: (T3) (↓) P < 0.05 - NR	1 RCT (n=40) mean±SD<br>(T1) 24h after BoNT (↓)<br>(T2) 7 days after BoNT (↓)<br>(T3) 14 days after BoNT (↓)	LOW ⊕⊕○○
**cathepsin S/**			pg/mL	• (n=8) PHI vs sham: (B) - (↑) P < 0.05 - NR<br>• (n=8) PHI+BoNT (7U/kg) vs PHI+vehicle: (T1) NS P > 0.05 - NR<br>• (n=8) PHI+BoNT (7U/kg) vs PHI+vehicle: (T2) NS P > 0.05 - NR<br>• (n=8) PHI+BoNT (7U/kg) vs PHI+vehicle: (T3) (↓) P < 0.05 - NR	1 RCT (n=40) mean±SD<br>(T1) 24h after BoNT (NS)<br>(T2) 7 days after BoNT (NS)<br>(T3) 14 days after BoNT (↓)	LOW ⊕⊕○○
**fractalkine**				• (n=8) PHI vs sham: (B) - (↑) P < 0.05 - NR<br>• (n=8) PHI+BoNT (7U/kg) vs PHI+vehicle: (T1) NS P > 0.05 - NR<br>• (n=8) PHI+BoNT (7U/kg) vs PHI+vehicle: (T2) (↓) P < 0.05 - NR<br>• (n=8) PHI+BoNT (7U/kg) vs PHI+vehicle: (T3) (↓) P < 0.05 - NR	1 RCT (n=40) mean±SD<br>(T1) 24h after BoNT (NS)<br>(T2) 7 days after BoNT (↓)<br>(T3) 14 days after BoNT (↓)	LOW ⊕⊕○○
**Neurons**	**TRG sensory neurons**					
**IB4 (+)**<br>**IB4 (-)**	**TN**	**Kitamura 2009 [29]**	KCl-evoked vesicular release FM4-64; (r) decay time constant	• sham+saline vs TN (a) IB4+ / (b) IB4- : (B) (↑) vesicular release/ (↓) (r) - (a) (r) (n=11) 26.4±8.1s vs (n=8) 3.1±1.0s / (b) (r) (n=10) 11.1±2.6s vs (n=8) 4.2±0.9s<br>• TN+BoNT vs TN+saline ipl: (T1) (a)/ (b) (↓) KCL-evoked vesicular release & (↓) slower onset - NR	1 nRT (n=?) mean±SEM<br>(T1) 11-days after BoNT (↓) ipl.	VERY LOW ⊕○○○
**Inflammatory**	**Cranial dura tissue**					
**cells** lymphocyte, monocyte, neutrophile, plasma cells	**Trigeminal inflammatory pain - TMDs**	**Lacković, 2016 [20]**	number of Giemsa positive profiles -	• (n/group 5) CFA+saline vs saline (sham): (B) (↑) P < 0.001 - NR<br>• (n/group 5) CFA+BoNT vs CFA+saline: (T1) (↓) P < 0.001- NR	1 RCT (n=105) mean ± SEM<br>(T1) 4-days after BoNT (↓)	MODERATE ⊕⊕⊕○
**Apoptosis rates**	**Scar tissue (ear)**					
**of fibroblasts**	**HS**	**Wang, 2020 [25]**	(%)	• (n=12) healthy skin vs HS: (B) (↓) P<0.01 – NR<br>• (n=12) HS+BoNT (0.5U, 1U, 1.5U, 2U) vs HS: (T1) (↑) P < 0.05– NR	1 RCT (n=18) mean ± S.D<br>(T1) 28 days after BoNT (↑)	LOW ⊕⊕○○
	**TG**					
**HIF-1α**	**TN**	**Cho, 2022 [17]**	protein expression (pg/ml tissue)	• (n=6) TN vs (n=6) sham or (n=6) naive: (B) ip.l (↑) P<0.05; cl. (NS) - NR<br>• (n=6) TN+BoNT (3U) vs (n=6) TN+vehicle: (T1) (↓) (P<0.05) - NR	1 RCT (n=236) mean ± SEM<br>(T1) 2 days after BoNT (↓)	LOW ⊕⊕○○
	**TG (mandibular (V3) division and boundary area)**					
**Nav 1.3**	**TN**	**Yang, 2016 [31]**	expression	• (n=5 per group) TN vs sham: (B) NS – NR<br>• (n=5 per group) TN+BoNT vs TN+saline: (T1) NS - NR	1 ? (n=?) mean±SEM<br>(T1) 6-days after BoNT (NS)	VERY LOW ⊕○○○

**Nav 1.6**				• (n=5 per group) TN vs sham: (B) (↑) P < 0.05 – NR<br>• (n=5 per group) TN+BoNT vs TN+saline: (T1) NS - NR	1 ? (n=?) mean±SEM<br>(T1) 6-days after BoNT (NS)	VERY LOW ⊕○○○
**Nav 1.7**				• (n=5 per group) TN vs sham: (B) (↑) P < 0.05 – NR<br>• (n=5 per group) TN+BoNT vs TN+saline: (T1) (↓) F2,12 = 9.176, P < 0.05	1 ? (n=?) mean±SEM<br>(T1) 6-days after BoNT (↓)	VERY LOW ⊕○○○
**Nav 1.8**				• (n=5 per group) TN vs sham: (B) (↑) P < 0.05 – NR<br>• (n=5 per group) TN+BoNT vs TN+saline: (T1) NS - NR	1 ? (n=?) mean±SEM<br>(T1) 6-days after BoNT (NS)	VERY LOW ⊕○○○
**ATF3**			positive cells	• (n=5 per group) TN vs sham: (B) (↑) P < 0.05 – NR<br>• (n=5 per group) TN+BoNT vs TN+saline: (T1) NS - NR	1 ? (n=?) mean±SEM<br>(T1) 6-days after BoNT (NS)	VERY LOW ⊕○○○
**Trigeminal spinal subnucleus caudalis**						
**TRPM3**	**TN**	**Zhang, 2019 [32]**	protein expression, OD	• TN+saline vs sham: (B) (↑) P<0.05 – NR<br>• TN+BoNT (3U, 10U) vs TN+saline: (T1) (↓) P<0.05 - NR	1 RCT (n=236) mean ± SEM<br>(T1) 7 days after BoNT (↓)	LOW ⊕⊕○○
**TRPV4**				• TN+saline vs sham: (B) (↑) P<0.05 – NR<br>• TN+BoNT (3U, 10U) vs TN+saline: (T1) (↓) P<0.05 - NR	1 RCT (n=236) mean ± SEM<br>(T1) 7 days after BoNT (↓)	LOW ⊕⊕○○
**Brainstem Vc region (caudal subnucleus of the spinal trigeminal nucleus)**						
**TRPV1**	**TN**	**Wu, 2016 [23]**	protein levels, OD	• (n=6) TN+saline vs (n=6) sham+saline+saline: (B) (↑) (P <0.05) - NR<br>• (n=6) TN+saline+Peripheral BoNT (3U/10U) vs (n=6) TN+saline+saline: (T1) (↓) (P <0.05) - NR	1 ? (n=?) mean ± SD<br>(T1) 7 days after BoNT (3U, 10U) (↓)	VERY LOW ⊕○○○
**TRPV2**				• (n=6) TN+saline vs (n=6) sham+saline+saline: (B) (↑) (P <0.05) - NR<br>• (n=6) TN+saline+Peripheral BoNT (3U) / (10U) vs (n=6) TN+saline+saline: (T1) 3U - (X) (P >0.05) / 10U - (↓) (P<0.05) - NR	1 ? (n=?) mean ± SD<br>(T1) 7 days after BoNT (10U) (↓)	VERY LOW ⊕○○○
**TRPM8**				• (n=6) TN+saline vs (n=6) sham+saline+saline: (B) (↑) (P <0.05) - NR<br>• (n=6) TN+saline+Peripheral BoNT (3U/10U) vs (n=6) TN+saline+saline: (T1) (X) (P >0.05) - NR	1 ? (n=?) mean ± SD<br>(T1) 7 days after BoNT (NS)	VERY LOW ⊕○○○
**Hippocampus (brain) tissues – total protein isolates**						
**SOD**	**Anxiety & ageing**	**Yesudhas, 2021 [34]**	U/mg protein	• control+saline vs (n=6) BoNT+saline: (T1) 0.63 ± 0.08 vs 0.76 ± 0.03, (↑) P < 0.05	1 ? (n=12) mean ± SD<br>(T1) 30-days after BoNT (↑)	VERY LOW ⊕○○○
**Catalase**				• control+saline vs (n=6) BoNT+saline: (T1) 27.5 ± 3.3 vs 34.5 ± 2.4, (↑) P < 0.05	1 ? (n=12) mean ± SD<br>(T1) 30-days after BoNT (↑)	VERY LOW ⊕○○○
**GSH**				• control+saline vs (n=6) BoNT+saline: (T1) 0.29 ± 0.008 vs 0.38 ± 0.05, (↑) P < 0.05	1 ? (n=12) mean ± SD<br>(T1) 30-days after BoNT (↑)	VERY LOW ⊕○○○
**GPx**				• control+saline vs (n=6) BoNT+saline: (T1) 18.4 ± 2.3 vs 21.2 ± 1.3, (↑) P < 0.05	1 ? (n=12) mean ± SD<br>(T1) 30-days after BoNT (↑)	VERY LOW ⊕○○○

LEGEND: RCT, randomised controlled trial; **nRT**, non-randomised trial; **RoB**, risk of bias; **CIS**, chronic inflammatory state; **BoNT,** botulinum toxin; **(↑)**, statistically significantly higher/increased; **(↓)**, statistically significantly lower/decreased; **(√)**, increased but not significantly; **(-)**, decrease but not significantly; **(X)**, no remarkable changes; **(B)**, baseline; **SEM,** standard error of the mean; **NS**, no statistically significant difference; **NR**, not reported; **ip.l.**, ipsilateral; **c.l.**, contralateral; **s.c.**, subcutaneously; **i.c.**, intracisternally**; i.a**., intraarticular; **i.g**. intraganglionic; **POD**, post operative day; **Tx**, treatment; **O.D**, optical density**; (mm)**, millimetres; **SD**, standard deviation; **(r),** decay time constant; **HS**, hypertrophic scar; **MMP-13**, matrix metallopeptidase; **TG**, trigeminal ganglia; **PD**, Parkinson Disease; **TMD**,

temporomandibular disorder; **TMJ**, temporomandibular joint; **OA**, osteoarthritis; **TN**, trigeminal neuralgia; **PIH**, persistent immunogenic hypersensitivity; **TNF-α**, tumor necrotic factor-α; **IL**, interleukin; **BDNF**, brain derived neurotrophic factor; **(cl)SNAP-25**, (cleaved) synaptosomal-associated protein-25; **ChAT**, choline acetyltransferase; **PSD95**, postsynaptic density-95; **NMDAR**, N-methyl-D-aspartate receptor; **5-HT**, 5-hydroxytryptamine; **p-ERK**, phosphorylated extracellular signal-regulated kinase; **p-CREB**, cAMP response element binding protein; **Iba-1**; ionized calcium-binding adaptor molecule 1; **TLRs** - toll-like receptors; **c-Fos**, neuron activation marker; **GFPA**, glial fibrillary acidic protein; **IgE**, immunoglobulin E; **CX3CR1,** CX3 chemokine receptor 1; **IB4**, isolectin B4-binding; **HIF-1α**, hypoxia-inducible factor; **TH**, tyrosine hydroxylase, dopaminergic neuronal marker; **VGlut2,** vesicular glutamate transporter 2; **VGAT,** vesicular GABA transporter; **SOD**, superoxide dismutase; **GSH,** glutathione; **GPx,** glutathione peroxidase; **ATF3**, activating transcription factor 3; **TRPV4**, protein expression of transient receptor potential vanilloid type 4; **TRPM**, transient receptor potential melastatin; **TNCB**, 2-Chloro-1,3,5-trinitrobenzene; **TRPV,** transient receptor potential vanilloid type ; **Vc**, caudal subnucleus of the spinal trigeminal nucleus; **AEW**, acetone-diethylether-water; **TGF-β1**, transforming growth factor beta; **GAPDH,** Glyceraldehyde 3-phosphate dehydrogenase; **SNpc,** substantia nigra pars compacta; **SRS**; spatial restraint stress; **CRS**; chronic restraint stress; **CFA**, Complete Freund's Adjuvant; **NTG**, nitroglycerin.

KEY - GRADE certainty ratings: Very low, the true effect is probably markedly different from the estimated effect**. Low,** the true effect might be markedly different from the estimated effect**. Moderate,** the authors believe that the true effect is probably close to the estimated effect**. High,** the authors have a lot of confidence that the true effect is similar to the estimated effect
