# Supplementary material for: Botulinum Toxin Effects on Biochemical Biomarkers Related to Inflammation-Associated Head and Neck Chronic Conditions: A Systematic Review of Preclinical Research
Source: Toxins (Basel). 2025 Jul 29;17(8):377. doi: 10.3390/toxins17080377 (PMC12390450; doi:10.3390/toxins17080377)
Supplement: Supplementary file 1 [file toxins-17-00377-s001.zip › SR2. file S7. Table S6. Synopsis of outcomes.pdf]

File S7. Table S6. Synopsis of outcomes. Characteristics & Outcomes of the included preclinical trials

PRECLINICAL STUDIES											
**Author Year**	**Chronic Inflammatory State Study design Exposure**	**Biological sampling** (Biomarker Measurement unit)	**Values presented / Measurement time point**	**N, Species, Age, Gender (Weight)**						**P value**	**NOTES** (other assessments)
				**Sham**	**Control**	**Intervention (BoNT)**			**Placebo / Different intervention**		
**BIOMARKER:** IL-1β											
**Makawi, 2022 [13]**	**TMJ osteoarthritis (OA)** induced by monosodium iodoacetate<br><br>Sham - no injection (right)<br>OA - injection left TMJ, BoNT - injection right TMJ (4 weeks following OA induction)	**TMJ tissues** (pg/ml tissue; mRNA; protein expression)	mean (m) , SD values of ANOVA - comparison between groups	**(n=42) +3 Wister albino rats, 3-4 months, Male (180 -200g)**						* (↓) p<0.001	histological analysis, bone area % and joint space (mm) between head of condyle and temporal bone (CBCT):<br>• BoNT-treated samples showed (↓) mean bone area % than other treated groups (p<0.001).<br>•Treated sides ↑ mean joint space than the untreated sides - statistically significant only for BoNT (p<0.001)
				(n=3) sham vs control	(n=42) OA (n=2/group sacrificed)	(n=12) OA+BoNT (5U/kg)			(n=12) PRP<br>(n=12) BoNT + PRP		
						treated right side *	untreated left side				
			2 weeks post-Tx	72.2h(m), 2.6(SD)	321.3d(m), 4.4(SD)	205.3f(m), 10.6(SD)	405.3b(m), 17.2(SD)				
			4 weeks post-Tx	72.2G(m), 2.6(SD)	321.3D(m), 4.4(SD)	113.6E(m), 14.7(SD)	464.6C(m), 12.0(SD)				
**Chen, 2021 [14]**	**Trigeminal Neuralgia (TN)** modified TN model induced by IoNC - neuropathic pain and anxiety-like behaviors<br><br>BoNT s.c. unilateral peripheral (facial) injection into whisker pad on the ip.l. side of IoNC (2-weeks after IoNC)<br>* 2-weeks after BoNT-analgesic effect worn off (lasted about 9-days), a 2nd injection performed.	**Trigeminal nucleus caudalis (TNC)** (mRNA expression; fold change)	mean±SEM. Unpaired Student's t-test. Two-Way ANOVA+post-hoc Bonferroni test.	**(n=48) C57BL/6 mice, 6-8 weeks, Male (20g)**						* (↑) P=0.0001<br><br>** NS<br><br>*** (↓) P<0.0001	•**Data from 4 mice/group were used for statistical analysis**.<br>• DRS (c.l): 1f.c = 3.13cm<br>• DRS (ip.l): 1f.c = 2.5cm<br>•Bilateral mechanical pain (von Frey test), Hypersensitivity (OFT), Anxiety-like behaviour (elevated plus-maze testing), and (depressive-like behaviour) FST: (↓) IoNC-induced bilateral mechanical pain hypersensitivity (appeared after 1h, lasted 9 days) and anxiety-like behaviours (but not depression-like behavior)<br>•Pain behaviours of TN (n=6) CCI+*Tlr2*$^{-/-}$ to investigate the role of TLR2 on the development of persistent pain.
				(n=12) sham vs control	(n=18) TN+vehicle	(n=12) TN+BoNT (0.18U) vs vehicle			(n=6) TN+ *Tlr2*$^{-/-}$		
				c.l / ip.l	c.l / ip.l	c.l	ip.l				
				c.l: NR** DRS 1f.c / 3.13<br>ip.l: DRS 1f.c / 2.5	c.l: NR** DRS 0.88 f.c = 2.76 cm<br>ip.l: **t6 = 8.633*** DRS 1.74 f.c = 4.34cm						
			5 day after BoNT/ 19-day after IoNC		c.l: NR	NR DRS 0.85 f.c = 2.67cm	**t6 = 10.39***** DRS 0.916 f.c = 2.29cm				
**Muñoz-Lora, 2017 [15]**	**Persistent Immunogenic Hypersensitivity (PIH)** by	**Peri-articular tissues from TMJ and**	ANOVA: Tukey's test	**(n=?) Wistar rats, age unknown, Male (250–500g)**						* (↑) P<0.05<br><br>** (↓) P<0.05	Other assessments: behavioural nociceptive tests.<br>•DRS: 2000 pg/ml = 2.5cm
				non-immunised	PHI	PIH+ BoNT 3.5U	PIH+ BoNT 7U	PIH+ BoNT 14U	PIH+Saline (NaCl-0.9%)		

	systemic immunization - mBSA/PBS+CFA<br>**& TMJ rheumatoid arthritis** by mBSA + formalin (0.5%) intraarticular TMJ injection<br><br>Sham: mBSA i.a.TMJ injection.<br>Experimental: i.a.TMJ injection, day-42/7days after last challenge of TMJ induction	**Trigeminal ganglia (TG)**<br>(pg/mL)		NR<br>DRS 0.6cm = 480pg/ml	NR*<br>DRS 6.37cm = 5096pg/ml				NR*** vs PHI<br>DRS 5.34cm = 4272pg/ml	*** NS P>0.05	• BoNT (3.5;7;14U) ↓ PIH induced by arthritis in the TMJ of rats without differences among groups. Established the dose of the BoNT at 7 U/Kg for the next experiments.
			24h after BoNT			NR	NR**<br>DRS 2.7cm = 2160 pg/ml	NR	NR		
			14-days after BoNT			NR	NR**<br>DRS 0.3cm = 240 pg/ml	NR	NR		
**Muñoz-Lora, 2020 [16]**	**PIH** by systemic immunization - mBSA/PBS+CFA<br>**& TMJ rheumatoid arthritis** by mBSA + formalin (0.5%) intraarticular TMJ injection<br><br>▪Sham: mBSA/PBS+ mBSA i.a.TMJ injection.<br>▪Experimental - unilateral/ ip.l. to immunisation intra-TMJ injection, day-42/7days after last challenge of TMJ induction<br><br>Seven days after the last intra-TMJ injection of the immunization protocol animals were treated with BoNT or vehicle saline. Sterile saline (0.9% NaCl; 20 µl) was injected in non-immunized and immunized groups	**Trigeminal subnucleus caudalis**<br>(pg/ml tissue)	mean ± SD.<br>one-way ANOVA + post hoc Tukey's test	**(n=40) Wistar rats, age unknown, Male (300–400 g)**						* (↑) P < 0.05<br><br>** (↓) P < 0.05<br>(this effect was reversed 7 days after Tx)	**• Number of samples for each experimental group = 8.**<br>•DRS: 500 pg/ml = 1.2cm
				non-immunised + vehicle saline (0.9% NaCl)	Immunised – PHI (TMJ arthritis) +vehicle saline (0.9% NaCl)	PHI+BoNT (7U/kg)					
				DRS<br>0.88cm = 366.67pg/ml	NR*vs sham<br>DRS 3.16cm = 1316.67pg/ml						
			24h after BoNT			NR** vs vehicle<br>DRS 1.57cm = 654.17pg/ml					
			7-days after BoNT			NR* vs sham<br>DRS 2.87cm = 1195.83pg/ml					
			14-days after BoNT			NR* vs sham<br>DRS 2.66cm = 1108.33pg/ml					
**Cho, 2022 [17]**	**Trigeminal neuralgia** by compression of the trigeminal nerve root (TNR)<br><br>(1 or 3 U/kg) single BoNT injection (POD-5) or repeated BoNT (POD-12)	**Trigeminal ganglion (TG)**<br>(protein concentration; pg/ml tissue)	mean ± SEM.<br>Student's t-test and one way ANOVA + Holm-Sidak post hoc	**(n=236) Sprague-Dawley rats, age unknown, Male (250−280 g)**						* (↑) (P<0.05)<br><br>**(↓) (P<0.05)	**•Data from 6 mice/ group were used.**<br>•DRS (naive, sham, TN): 40pg/ml = 3.04cm.<br>•DRS (vehicle, BoNT): 50pg/ml = 2.96cm
				sham / naive	TN	TN + Single BoNT		TN + Repeated BoNT	TN + vehicle		
					c.l / ip.l	3U / 1U		3U / 1U			
				DRS 3.96 cm = / DRS 4.14 cm =	vs sham or naïve<br>*NR						

				52.10 pg/ml	54.47 pg/ml	DRS 6.25cm = 82.24pg/ml									•Other assessments: Mechanical Allodynia (changes in the air-puff thresholds) and up-regulate IL-1β, IL-6, and TNF-α concentrations in TG after PX-12 injection, a HIF-1α inhibitor (to check participation of the HIF-1α associated cytokine pathway in the development of TN)<br>•Single/double Tx with high BoNT (3U/kg) led to significantly prolonged antinociceptive effects. Repeated s.c. injections of low dose BoNT (1U/kg) did not affect the air-puff thresholds.<br>•PX-12, compared to vehicle led to significant anti-allodynic effects and ↓ IL-1β, IL-6, and TNF-α levels.
			Day-2 after BoNT/ POD-7					** NR DRS 3.84 cm = 64.86 pg/ml	NR	NR	NR	DRS 6.75cm = 114.02pg/ml			
**Li, 2023 [18]**	**Depression** by reserpine chronic administration **in Parkinson´s disease model.**<br><br>From the 10th week, BoNT (10U/kg⁻¹ ·d⁻¹) injected into the cheek once daily for 3 consecutive days.	**Brain-Substantia nigra pars compacta (SNpc) & hippocampus** (mRNA; protein expression)	mean±SEM. Two-way ANOVA + Bonferroni's and one-way ANOVA + Tukey's post hoc test	**(n=?) Parkinson disease model-ICR mice, 6–8 weeks old, Male (30g)**										* (↑) (mRNA and protein expression)<br><br>** (↓) P=0.0013<br><br>*** (↓) P = 0.0019	**•Protein concentrations ([]) were measured (n=3) mice/each group. mRNA expression was determined (n=5) mice/each group.**<br>•DRS (Protein [], hippocampus by ELISA): 600 pg/g = 2.8cm<br>•DRS (mRNA): 1 GAPDH = 1.16cm<br>•Other assessments: Behavioural test (body weight, Rotarod test, Pole climbing test, OFT, FST, Tail suspension test, Sucrose preference test), In vitro cell culture-CCK-8 assay (mouse BV2 microglial cell line supplemented with reserpine in the presence/absence of BoNT for 24h).<br>•BoNT significantly helped depressive-like behaviours.
				Control		Reserpine vs. control (no BoNT) * NR		Reserpine + BoNT Vs. reserpine				Control + BoNT			
				DRS Protein [] 2.9cm = 621.43pg/g		DRS Protein [] 4.1cm = 878.57pg/g									
				DRS mRNA 1.16cm = 1 GAPDH		DRS mRNA 2.17cm = 1.87 GAPDH									
			(?) follow-up					Protein [] by ELISA		mRNA by qRT–PCR		DRS Protein [] 2.5cm = 535.71 pg/g	DRS mRNA 0.94cm = 0.81 GAPD H		
								Hyppocampal							
								** F (3, 8) = 14.50 DRS 3.14cm = 672.86pg/g		*** F (3,16) = 7.872 DRS 1.3cm = 1.12 GAPDH					
**BIOMARKER:** TNF-α															
**Chen, 2021 [14]**	**Trigeminal Neuralgia** modified TN model induced by IoNC - neuropathic pain and anxiety-like behaviors	**Trigeminal nucleus caudalis (TNC)** (mRNA; protein expression)	mean±SEM. Unpaired Student's t-test. Two-Way ANOVA + post-hoc Bonferroni test.	**(n=48) C57BL/6 mice, 6-8 weeks, Male (20g)**										* (↑) P=0.0001<br><br>** NS<br><br>*** (↓) P < 0.0001	**•Data from 4 mice/ group were used for statistical analysis.**<br>• DRS (c.l): 1f.c = 3.13cm<br>• DRS (ip.l): 1f.c = 2.5cm<br>•Bilateral mechanical pain (von Frey test), Hypersensitivity (OFT), Anxiety-like behaviour (elevated
				(n=12) sham vs control		(n=18) TN+vehicle		(n=12) TN+BoNT (0.18U) vs vehicle				(n=6) TN+*Tlr2*$^{-/-}$			
				c.l	ip.l	c.l	ip.l	c.l		ip.l					
				NR** DRS 1f.c	DRS 1f.c	NR** DRS	**t6 = 8.990***								

	BoNT s.c unilateral peripheral (facial) injection into the whisker pad on the ip.l. side of IoNC (2-weeks after IoNC)<br>* 2-weeks after BoNT-analgesic effect worn off (lasted about 9-days), a 2nd injection performed.			= 3.13 cm	= 2.5 cm	1.08f.c = 3.38 cm	DRS 1.97f.c = 4.92cm				plus-maze testing), and (depressive-like behaviour) FST: (↓) IoNC-induced bilateral mechanical pain hypersensitivity (appeared after 1h, lasted 9 days) and anxiety-like behaviours (not depression-like behavior)<br>•Pain behaviours of TN (n=6) CCI+*Tlr2*$^{-/-}$ to investigate the role of TLR2 on the development of persistent pain.
			5 day after BoNT/ 19-day after IoNC			NR		NR DRS 0.97f.c = 3.03cm	**t6 = 9.878***** DRS 0.87f.c = 2.18cm		
**Muñoz-Lora, 2017 [15]**	**PIH** by systemic immunization - mBSA/PBS+CFA **& TMJ rheumatoid arthritis** by mBSA + formalin (0.5%) intraarticular TMJ injection<br><br>Sham: mBSA i.a.TMJ injection Experimental: i.a. TMJ injection, day-42/7days after last challenge of TMJ induction	**Peri-articular tissues from TMJ and Trigeminal ganglia (TG)** (pg/mL)	ANOVA: Tukey's test	**(n=?) Wistar rats, age unknown, Male (250–500g)**						* (↑) P < 0.05<br><br>** NS P > 0.05	Other assessments: behavioural nociceptive tests.<br>•DRS: 50pg/ml = 2.44cm<br>• BoNT (3.5;7;14U) ↓ PIH induced by arthritis in the TMJ of rats without differences among groups. Established the dose of the BoNT at 7 U/Kg for the next experiments.
				non-immunised	PHI	PIH+ BoNT 3.5U	PIH+ BoNT 7U	PIH+ BoNT 14U	PIH+Saline (NaCl-0.9%)		
				NR DRS 1.89cm = 38.73pg/ml	NR* DRS 6.39cm = 130.94pg/ml				NR** vs sham DRS 5.85cm = 119.88pg/ml		
			24h after BoNT			NR	NR** DRS 4.2cm = 86.06 pg/ml	NR	NR		
			14-days after BoNT			NR	NR** DRS 6.82cm = 139.75pg/ml	NR	NR		
**Muñoz-Lora, 2020 [16]**	**PIH** by systemic immunization - mBSA/PBS+CFA **& TMJ rheumatoid arthritis** by mBSA + formalin (0.5%) intraarticular TMJ injection<br><br>▪Sham: mBSA/PBS+ mBSA i.a.TMJ injection.<br>▪Experimental - unilateral/ ip.l. to immunisation intra-TMJ injection, day-42/7days after last challenge of TMJ induction	**Trigeminal subnucleus caudalis** (pg/ml tissue)	mean ± standard deviation (SD). one-way ANOVA + post hoc Tukey's test	**(n=40) Wistar rats, age unknown, Male (300–400 g)**						* (↑) P < 0.05<br><br>** NS P > 0.05<br><br>*** (↓) P < 0.05	**• Number of samples for each experimental group = 8.**<br>• DRS: 2000pg/ml = 1.62cm
				non-immunised+ vehicle saline (0.9% NaCl)	Immunised – PIH (TMJ arthritis) +vehicle saline (0.9% NaCl)	PIH+BoNT (7U/kg)					
				DRS 2.89cm = 3567.90pg/ml	NR*vs sham DRS 3.55cm = 4382.72pg/ml						
			24h after BoNT			NR** DRS 2.97cm = 3666.67pg/ml					
			7-days after BoNT			NR** DRS 3.26cm = 4024.69pg/ml					
			14-days after BoNT			NR*** vs placebo					

	Seven days after the last intra-TMJ injection of the immunization protocol animals were treated with BoNT or vehicle saline. Sterile saline (0.9% NaCl; 20 µl) was injected in non-immunized and immunized groups					DRS 2.55cm = 3148.15pg/ml			
**Cho, 2022 [17]**	**Trigeminal neuralgia** by compression of the trigeminal nerve root (TNR)<br><br>(1 or 3 U/kg) single BoNT injection (POD-5) or repeated BoNT (POD-12)	**Trigeminal ganglion (TG)** pg/ml tissue; protein concentration	mean ± SEM. Student's t-test and one way ANOVA + Holm-Sidak post hoc	**(n=236) Sprague-Dawley rats, age unknown, Male (250−280 g)**				* (↑) (P<0.05)<br><br>**(↓) (P<0.05)	**•Data from 6 mice/group were used.**<br>•DRS (naive, sham, TN): 2pg/ml = 2.53cm.<br>•DRS (vehicle, BoNT): 2pg/ml = 2.98cm.<br>•Other assessments: Mechanical Allodynia (changes in the air-puff thresholds) and up-regulate IL-1β, IL-6, and TNF-α concentrations in the TG after PX-12, a HIF-1α inhibitor.<br>•Single/double Tx with high BoNT (3 U/kg) led to significantly prolonged antinociceptive effects. Repeated s.c. injections of low dose BoNT (1 U/kg) did not affect the air-puff thresholds.<br>•PX-12, compared to vehicle led to significant anti-allodynic effects and ↓ IL-1β, IL-6, and TNF-α levels.
				sham | naïve	TN<br>c.l | ip.l	TN + Single BoNT (3U | 1U) | TN + Repeated BoNT (3U | 1U)	TN + vehicle		
				sham: DRS 1.22 cm = 0.96 pg/ml<br>naïve: DRS 1.33 cm = 1.05 pg/ml	vs sham or naïve<br>* NR<br>DRS 4.84cm = 3.83pg/ml				
			Day-2 after BoNT/ POD-7			Single 3U: ** NR DRS 3.42 cm = 2.29 pg/ml<br>Single 1U: NR<br>Repeated 3U: NR<br>Repeated 1U: NR	DRS 6.82cm = 4.58 pg/ml		
**Li, 2023 [18]**	**Depression** by reserpine chronic administration **in Parkinson´s disease model.**<br><br>From the 10th week, BoNT (10U/kg⁻¹ ·d⁻¹) injected into the cheek once daily for 3 consecutive days.	**Brain- Substantia nigra pars compacta (SNpc) & hippocampus** (mRNA, GAPDH; protein expression, pg/g)	mean±SEM. Two-way ANOVA + Bonferroni's and one-way ANOVA + Tukey's post hoc test	**(n=?) Parkinson disease model-ICR mice, 6–8 weeks old, Male (30g)**				* (↑) (mRNA and protein expression)<br><br>** (↓) P = 0.0002<br><br>*** (↓) P = 0.0003	**•Protein concentrations ([]) were measured (n=3) mice/each group. mRNA expression was determined (n=5) mice/each group.**<br>•DRS (Protein [], hippocampus by ELISA): 1000 pg/g = 0.94cm<br>•DRS (mRNA): 1 GAPDH = 1.6cm<br>•Other assessments: Behavioural test (body weight, Rotarod test, Pole climbing test, OFT, FST, Tail suspension test, Sucrose preference test), In vitro cell culture-CCK-8
				Control	Reserpine vs. control (no BoNT) * NR	Reserpine + BoNT Vs. reserpine	Control + BoNT		
				DRS Protein [] 3cm = 3191.49pg/g<br>DRS mRNA 1.6cm = 1 GAPDH	DRS Protein [] 3.84cm = 4085.11pg/g<br>DRS mRNA 3.21cm = 2.0 GAPDH				
			(?) follow-up			Protein [] by ELISA | mRNA by qRT–PCR<br>Hyppocampal	DRS Protein [] | DRS mRNA		

							** F (3, 8) = 23.78 DRS Protein [] 3.37cm = 3585.11 pg/g	*** F (3,16) = 11.79 DRS mRNA 2.3cm = 1.43 GAPDH	2.65cm = 2819.15 pg/g	1.69cm = 1.1 GAPD H		assay (mouse BV2 microglial cell line supplemented with reserpine in the presence/absence of BoNT for 24h).<br>•BoNT significantly ameliorated depressive-like behaviours.
**BIOMARKER:** CGRP												
**Shao, 2013 [19]**	**Migraine** by nitroglycerin (NTG)<br><br>Vehicle or BoNT subcutaneous (frontal and temporal) injection 2h after NTG	**Jugular plasma and medulla oblongata - containing caudal trigeminal nucleus** (pg/mL)	means ± SEM. One-way ANOVA. Binomial qualitative data, comparisons between groups (chi-square)		**(n=32) Sprague-Dawley rats, age unknown, Female (250-300g)**						+ (↑) 1.8-fold, P<0.01<br>++ (↑) 1.8-fold, P<0.05<br><br>* (↓) P<0.01<br><br>** NS P>0.05 (between doses)	•DRS (a) 20pg/ml = 1cm<br>•DRS (b) 2pg/ml = 0.86cm<br>▪**Analysis (n=8)/ group**
					(n=8) Sham (control)	(n=8) NTG + vehicle	(n=8) NTG+ BoNT 5U/kg	(n=8) NTG+ BoNT 10U/kg				
					(a) jugular plasma	+ NR						
					(b) oblongata	++ NR						
			24h after BoNT	(a)	DRS 2.6cm = 52 pg/ml	DRS 4.7cm = 94 pg/ml	* NR DRS 2.75cm = 55 pg/ml	* NR DRS 2.75cm = 55 pg/ml				
							**					
				(b)	DRS 2.78cm = 6.46 pg/ml	DRS 4.95 cm = 11.51 pg/ml	* NR DRS 3cm = 6.98 pg/ml	* NR DRS 2.7cm = 6.28 pg/ml				
							**					
**Muñoz-Lora, 2022 [22]**	**Persistent Immunogenic Hypersensitivity** by systemic immunization - mBSA/PBS+CFA<br>**& TMJ rheumatoid arthritis** by mBSA + formalin (0.5%) intraarticular TMJ injection<br><br>Sham: mBSA i.a.TMJ injection<br>Experimental: unilateral left i.a.TMJ injection, day-42/7days after last challenge of TMJ induction	**Trigeminal nucleus caudalis (TNC)** threshold area of immunoreactivity (µm²)	Mean ± SEM, one-way ANOVA		**(n=40) Sprague–Dawley rats, 6–8 weeks, Male (300–400g)**						* NS – lack of changes	Other assessments - spontaneous nociception by examining RGS, evoked pain by examining facial mechanical allodynia over the skin covering the stimulated TMJ (Frey filaments) – behavioural assessments pre-formalin (day-13) + post-formalin (day-14)<br>•**Analysis on 5 randomly selected slices per animal (n=5 animals/ group)**<br>•DRS (ip.l) 50000µm² = 2cm<br>•DRS (c.l) 50000µm² = 1.94cm
					(n=10) control non-immunised	(n=10) PIH+Saline (NaCl-0.9%)	(n=10) PIH + OnaBoNT 7U	(n=10) PIH + AboBoNT 14U				
					NR	NR*						
			14-days after BoNT	ip.l	DRS 5.12 cm = 128000µm²	DRS 5.62 cm = 1405000µm²	* $F_{3,16}$ (ip.l.) = 0.3819					
							DRS 5.47 cm = 136750µm²	DRS 6.12 cm = 153000µm²				
				c.l	DRS 4.88 cm = 125773µm²	DRS 5.56 cm = 143299µm²	* $F_{3,16}$ (c.l.) = 1.267					
							DRS 4.47 cm = 115206µm²	DRS 6.27 cm = 161598µm²				
					**(n=?) Wistar rats, age unknown, Male (250–500g)**						* (↑) P < 0.05	

**Muñoz-Lora, 2017 [15]**	**PIH** by systemic immunization - mBSA/PBS+CFA **& TMJ rheumatoid arthritis** by mBSA + formalin (0.5%) intraarticular TMJ injection<br><br>Sham: mBSA i.a.TMJ injection<br>Experimental: i.a.TMJ injection, day-42/7days after last challenge of TMJ induction	**Peri-articular tissues from TMJ and Trigeminal ganglia (TG)** (ng/mL)	ANOVA: Tukey's test	non-immunised	PIH	PIH+ BoNT 3.5U	PIH+B oNT7 U	PIH+ BoNT 14U	PIH+Saline (NaCl-0.9%)	** (↓) P < 0.05	Other assessments: behavioural nociceptive tests.<br>•DRS: 0.05ng/ml = 2.05cm<br>• BoNT (3.5;7;14U) ↓ PIH induced by arthritis in the TMJ of rats without differences among groups. Established the dose of the BoNT at 7 U/Kg for the next experiments.
				NR<br>DRS 2.66cm = 0.065ng/ml	NR*<br>DRS 9.26cm = 0.22ng/ml				NR* vs sham<br>DRS 7.57cm = 0.18ng/ml		
			24h after BoNT			NR	NR**<br>DRS 0.87cm = 0.021 ng/ml	NR	NR		
			14-days after BoNT			NR	NR**<br>DRS 0.00cm = 0 ng/ml	NR	NR		
**Lacković, 2016 [20]**	**Trigeminal pain - temporomandibular disorders (inflammatory pain)** by CFA<br><br>CFA + BoNT single injections into left TMJ (3-days prior CFA):<br>**a**. Intra-articular (i.a.) (5Ukg^{-1},20 μL)<br>**b**. Intraganglionic (i.g.) – left TG through infraorbital foramen (2U kg^{-1},2μL)<br>**c**. Facial injections at 4 sites outside TMJ - total dose of 5U kg^{-1} divided in 4 equal doses (1.25 U kg^{-1} / site):<br>(i) bilaterally in forehead above orbital arch<br>(ii) bilaterally into whisker pad.	**Dura mater, TNC, Trigeminal ganglion (TG), CSF**<br>Concentration (fmol (mg wet tissue)$^{-1}$): dura mater, TNC, TG or (fmol mL^{-1}): CSF	means ± SEM. one-way ANOVA + Newman–Keuls post hoc test	**(n=105) Wistar rats, 3–3.5 months old, Male (300–350g)**						+ (↑) P < 0.01<br><br>++ (↑) P < 0.05<br><br>+++ NS<br><br>* (↓) P < 0.01<br><br>** NS	•Other assessments: behavioural testing (mechanical allodynia) by using von Frey monofilaments, Investigation of the effect of the axonal transport inhibitor, colchicine (7-days after **a.** and **b.**), on antinociceptive activity and appearance of clSNAP-25 in dura mater following BoNT injection.<br><br>**•Analysis on (n/ group = 6)**<br>•DRS (dura): 2 fmol(mg)$^{-1}$ = 1.24cm<br>•DRS (TNC ip.l): 50 fmol(mg)$^{-1}$ = 1.27cm<br>•DRS (TG ip.l): 20 fmol(mg)$^{-1}$ = 1.65cm<br>•DRS (CSF): 50 fmol(mg)$^{-1}$ = 1.24cm
				Saline control	CFA+saline (0.9% NaCl) (Vs control)	CFA+BoNT 5Ukg^{-1} a. intraarticular (Vs CFA+saline)			CFA+ sumatriptan (175μgkg^{-1}) 24h after CFA		
			(a) Cranial dura DRS	2.24cm = 3.61fmol(mg)$^{-1}$	NR+ 3.77cm = 6.08 fmol(mg)$^{-1}$						
			(b) TNC (ipsilateral) DRS	2.46cm = 96.85 fmol(mg)$^{-1}$	NR++ 3.4cm = 133.86 fmol(mg)$^{-1}$						
			(c) TG (ipsilateral) DRS	2.6cm= 31.51 fmol(mg)$^{-1}$	NR+++ 3.15cm = 38.18 fmol(mg)$^{-1}$						
			(d) CSF DRS	1.66cm = 50 fmol(mg)$^{-1}$	NR+++ 2.23cm = 67.17 fmol(mg)$^{-1}$						
			4-days after BoNT			(a) NR DRS * 2.53cm = 4.08 fmol(mg)$^{-1}$<br>(b) NR DRS ** 2.83cm = 111.42 fmol(mg)$^{-1}$<br>(c) NR DRS ** 2.65cm = 32.12 fmol(mg)$^{-1}$<br>(d) NR DRS ** 2.08cm =62.65 fmol(mg)$^{-1}$					
**BIOMARKER:** SNAP25											
**Li, 2019 [21]**	**Depression** by spatial restraint stress	**Hippocampus, hypothalamus, prefrontal cortex,**	mean ± SEM. Student's t-test. One/Two-way ANOVA + Bonferroni post-test.	**(n=?) ICR mice, 6–8 weeks, Male (20g–25g)**						*expression of SNAP25 in the hippocampus was not altered	Other assessments: Behavioural assessments: FST, tail suspension test, sucrose preference test, body weight, rotarod test (motor function), OFT (locomotory activity).
				Control (naïve)	Spatial restraint stress (SRS)	SRS + BoNT			Control + BoNT		
				DRS 3.55cm =0.95Tubulin	NR						

	BoNT 0.18U single facial intramuscular injection (3 points at each cheek)<br>Imipramine, 10mg/kg, fluoxetine 10 mg/kg injected intraperitoneally daily.	**amígdala (Brain)**<br>(protein expression by Tubulin)	(a) 1h, (b) 1-, (c) 3-, (d) 7-days (naive) (e) 16-, (f) 18-, (g) 22-, (h) 29-days (SRS)	* NR<br>DRS 3cm =0.95Tubulin	* NR<br>DRS 2.93cm =	* NR DRS (e) 3.02cm = 0.96 Tubulin	* NR DRS (a) 3.74cm =1 Tubulin		**•Analysis on (n=6) – BoNT+naïve; (n=6) – BoNT+SRS**<br>•DRS (naïve) 0.5 Tubulin = 1.86cm<br>•DRS (SRS) 0.5 Tubulin = 1.57cm<br>•BoNT Tx helps Depressive-Like Behaviours in Naïve Mice (n=5–10/group) & mice undergoing SRS.
						* NR DRS (f) 3.04cm = 0.97 Tubulin	* NR DRS (b) 3.76cm = 1 Tubulin		
						* NR DRS (g) 3.2cm = 1.02 Tubulin	* NR DRS (c) 3.52cm = 0.95 Tubulin		
						* NR DRS (h) 3cm = 0.96 Tubulin	* NR DRS (d) 3.69cm = 0.99 Tubulin		
**Muñoz-Lora, 2022 [22]**	**PIH** by systemic immunization - mBSA/PBS+CFA<br>**& TMJ rheumatoid arthritis** by mBSA + formalin (0.5%) intraarticular TMJ injection<br><br>Sham: mBSA i.a.TMJ injection (saline on day 42)<br>Experimental: unilateral left i.a.TMJ injection, day-42/ 7days after last challenge of TMJ induction	**Trigeminal nucleus caudalis (TNC)**<br>(cSNAP-25 staining/positive fibres)	Mean ± SE, one-way ANOVA	**(n=40) Sprague–Dawley rats, 6–8 weeks, Male (300–400g)**				*appeared as fibre-like processes in the ipsilateral TNC.<br><br>** not observed	Other assessments - spontaneous nociception by examining RGS, evoked pain by examining facial mechanical allodynia over the skin covering the stimulated TMJ (Frey filaments) – behavioural assessments pre-formalin (day-13) + post-formalin (day-14)<br>**•Analysis on 10 sections per animal (n=3/Tx group)**
				(n=10)<br>control non-immunised + Saline	(n=10)<br>PIH +<br>Saline<br>(NaCl-0.9%)	(n=10)<br>PIH +<br>OnaBoNT<br>7U / (n=10)<br>PIH +<br>AboBoNT<br>14U			
				NR	NR*				
			14-days after BoNT — ip.l		NR	* NR			
			14-days after BoNT — c.l<br><br>no Tx		NR	** NR			
**Lacković, 2016 [20]**	**Trigeminal pain - temporomandibular disorders (inflammatory pain)** by CFA<br><br>CFA + BoNT single injections into left TMJ (3-days prior CFA):<br>**a**. i.a. (5Ukg^{-1},20 μL)<br>**b**. i.g. – left trigeminal ganglion through infraorbital foramen (2U kg^{-1},2μL)<br>**c**. Facial injections at 4 sites outside TMJ - total dose: 5U kg^{-1} divided in 4 equal doses (1.25 U kg^{-1} / site):<br>(i) bilaterally into the rat forehead above orbital arch	**Cranial dura**<br>(presence of clSNAP-25 - fibers containing SNAP-25 co-expressed bright granular immunoreactivity for CGRP. Scale bars = 100 μm)	means ± SEM.<br>one-way ANOVA + Newman–Keuls post hoc test	**(n=105) Wistar rats, 3–3.5 months old, Male (300–350g)**				* presence of cleaved SNAP-25	•Other assessments: behavioural testing (mechanical allodynia) by using von Frey monofilaments, Investigation of the effect of the axonal transport inhibitor, colchicine (7-days after **a.** and **b.**), on antinociceptive activity and appearance of clSNAP-25 in dura mater following BoNT injection.<br>**•Analysis on 4 animals**<br>•presence of clSNAP-25 in the injected-side lateral and parietal dura near the dural blood vessels after BoNT single injections into TMJ and whisker pad (data not shown).<br>•c.l. dura was devoid of clSNAP-25, ruling out possible systemic BoNT diffusion.
				Saline control	CFA+saline<br>(0.9% NaCl)<br>(Vs control)	CFA+BoNT<br>(Vs CFA+saline)<br>a. / b. / c.	CFA+<br>sumatriptan<br>(175μgkg^{-1}) 24h<br>after CFA		
					Cranial dura<br>NR				
			4-days after BoNT			Cranial dura (ip.l.– near blood vessels and non-vascular areas)<br>* NR / *NR / *NR			

	(ii) bilaterally into whisker pad.												
**Ni, 2023 [24]**	**Major depressive disorder** by chronic restraint stress (CRS) - induced depression and drug administration.<br><br>pre-injection into the unilateral whisker intrinsic musculature (WIM) with 3 dosages at 3 different time points prior to the end of the restraint period:<br>i) 6 weeks before to the restrain end/ 3weeks before the restrain start,<br>ii) 3weeks before to the end of restrain/<br>1-day before restrain start,<br>iii) 3days before 21-day restraint stress end.	**Brain – hindbrain sections**<br>(positive signal)<br>(%positive signal - area)<br><br>(a) Scale bar, 100 µm<br>(b) & (c) Scale bar, 50 µm	mean±SEM. one-way ANOVA + Bonferroni' or Dunnett's multiple comparisons test. Two-tailed Student's t-test		**(n=?) pathogen-free C57BL/6J mice, 8 weeks, Male (25g)**							+ positive signals detected day-10 after BoNT<br>- No signals detected.<br><br>(a)* maximum positive signal (BoNT activity dose-dependent)<br><br>(a)** detected 10 days after BoNT#, highest expression at 4 weeks##, and persisted for at least 7 weeks.##<br><br>X SNAP25 surrounded ChAT and PSD95 but did not co-localize with either ChAT or PSD95.<br><br>« P<0.0001<br><br>(1)« 3 U/kg vs. 10 U/kg: P<0.0001<br>(1)«« 3 U/kg vs. 30 U/kg: P<0.0001<br>(2)« 10 U/kg vs. 30 U/kg: P=0.0169<br><br># P=0.0384<br><br>(a)# 10 days vs. 4 Weeks: P=0.8069<br>(a)## 10 days vs. 7 Weeks: P=0.4491	-Other assessments:<br>•monosynaptic retrograde tracer CTB-488 injected alone or CTB-488-mixed BoNT to label the wFMNs:<br>Co-immunostaining for NeuN (neuronal marker) and vAChT, SerT, or vGluT2 (synaptic markers)<br>•injection virus tracer (PRV-EGFP) - to rule out interference from afferent sensory regions and investigate the circuitry upstream of wFMNs.<br>•Following virus infection, (Ca^{2+}/ (CaMKII)), (GAD67), (TH), and (TpH2) - to identify the neuron types in the vlPAG that send afferent synapses to the wFMNs; and wFMNs-projecting vlPAG - to ascertain the anatomical synaptic connectivity of vlPAG input to wFMNs.<br>•neuroanatomical tracing, and specific chemogenetic manipulation to evaluate the contribution of the wFMNs-projecting vlPAG neurons to retrograde effects.<br>•behavioural tests (FST) - despair, sucrose preference test– anhedonia, and (OFT) - locomotor ability.<br><br>**▪Analysis on . n=4 cells from 3 mice per subgroup (dosages).**<br>**•# (n=4 brain sections from 3 mice in the group of 10 days)**<br>**•## (n=5 brain sections from 3 mice in the group of 4 weeks)**<br>**•### (n=6 brain sections from 3 mice in the group of 7 weeks)**
					Naïve + saline	CRS + saline	CRS + BoNT						
							(n=)	3U/Kg (1)	10U/Kg (2)	30 U/Kg (3)			
			(a) cl. SNAP-25_{197}<br>(b) SNAP25 + ChAT<br>(c) SNAP25 + PSD95	i)	(n=10)	(n=13)	(13)						
				ii)	(n=12)	(n=15)	(15)						
				iii)	(n=10)	(n=9)	(9)						
			#10-days after BoNT<br><br>##4-weeks after BoNT<br><br>###7-weeks after BoNT	(a)<br>i) ii) iii)			IFN ipl.l.	+<br>(1)<br>«<br>(1)<br>««	+<br>(a)**<br>(2)«<br>(a)#<br>(a)##<br>(a)###	+<br>(a)<br>*	« Quantificational analysis among subgroups of three different BoNT dosages n=4 cells from 3 mice per subgroup/BoNT dosages:<br>F (2, 9)=99.68 «<br><br># Quantificational analysis of % cl. SNAP25_{197}-positive signal areas among groups of 3 time points post-BoNT (10 U/kg):<br>F (2, 12)=4.33 #		
							FN ipl.l.	-<br>NR	-<br>NR	(a)<br>+++<br>(a)<br>*<br>NR			
							Pr5	-<br>NR	-<br>NR	-<br>NR			
							Sp5	-<br>NR	-<br>NR	-<br>NR			
				(b)<br>i) ii) iii)			X<br>NR						
				(c)<br>i) ii) iii)			X<br>NR						

											(a)### 4 weeks vs. 7 weeks: P=0.0382	
**Wu, 2016 [23]**	**Trigeminal neuropathy** by IoNC<br><br>BoNT/saline subcutaneously into the whisker pad tissue (ipsilaterally to the nerve injury) 14 days after the IoNC<br><br>Colchicine or normal saline (2 µl) was injected into TG (ip.l. to the nerve injury) of anesthetized rat, 12h before BoNT/saline (IoNC + colchicine + saline/ BoNT)	**Brainstem Vc region (caudal subnucleus of the spinal trigeminal nucleus)** (by β-actin, OD)	mean ± SD. One-way ANOVA and the New-man-Keuls test	**(n=?) Sprague–Dawley rats, age unknown, Male (220–300g)**							* (↑) (P <0.05)	•Other assessments: Antinociceptive effect/pain threshold/allodynia tested by Von Frey filaments, motor coordination ability by Rota-rod test. Colchicine effects.<br>▪**Analysis n = 6/ group**<br>•DRS 1OD = 1.15cm<br>•Peripheral application of BoNT (3, 10 U/kg) (↑) pain threshold of IoNC.<br>•Antinociceptive effects reached a max. level at 8 days and remained (↑) until 20 days. The 10 U/kg had better antinociceptive effects than the 3 U/kg but NS (P>0.05)<br>•Colchicine injection into TG resulted in failure to ↑ the level of clSNAP-25 and in disappearance of BoNT 10U/kg antinociceptive effects, P > 0.05
				(n=6) IoNC + saline + saline		(n=6) IoNC + Colchicine + saline		IoNC + saline + Peripheral BoNT (vs control)		(n=6) IoNC + colchicine + BoNT		
								(n=6) 3U/Kg	(n=6) 10 U/kg			
			7-days	DRS 1.25cm = 1.09OD		DRS 1.15cm = 1OD		NR	* NR DRS 5.45cm = 4.74OD	DRS 2.6cm = 2.26OD		
**BIOMARKER:** c-Fos												
**Chen, 2021 [14]**	**Trigeminal Neuralgia** modified TN model induced by IoNC - neuropathic pain and anxiety-like behaviors<br><br>BoNT s.c. unilateral peripheral (facial) injection into the whisker pad on the ip.l. side of IoNC (2-weeks after IoNC)<br>* 2-weeks after BoNT-analgesic effect worn off (lasted about 9-days), a 2nd injection performed.	**Trigeminal nucleus caudalis (TNC)** mRNA; protein expression - fold change	mean±SEM. Unpaired Student's t-test to compare 2 groups. Two-Way ANOVA + post-hoc Bonferroni test	**(n=48) C57BL/6 mice, 6-8 weeks, Male (20g)**							* (↑) P=0.0069<br><br>** (↑) P=0.0033<br><br>*** (↓) P = 0.0001<br><br>**** (↓) P=0.0011	•Data from 4 mice/group were used for statistical analysis.<br>•DRS (c.l): 1f.c = 4.16cm<br>•DRS (ip.l): 1f.c = 3.1cm<br>•Bilateral mechanical pain (von Frey test), Hypersensitivity (OFT), Anxiety-like behaviour (elevated plus-maze testing), and (depressive-like behaviour) FST: (↓) bilateral mechanical pain hypersensitivity (appeared after 1h, lasted 9 days) and anxiety-like behaviours (not depression-like behavior)<br>•Pain behaviours of TN (n=6) CCI+*Tlr2*$^{-/-}$ to investigate the role of TLR2 on the development of persistent pain.
				(n=12) vs control		(n=18) TN+vehicle		(n=12) TN+BoNT (0.18U) vs vehicle		(n=6) TN+*Tlr2*$^{-/-}$		
				c.l	ip.l	c.l	ip.l	c.l	ip.l			
				DRS 1f.c = 4.16 cm	DRS 1f.c = 3.1 cm	**t6 = 4.022*** DRS 1.23f.c = 5.12 cm	**t6 = 4.707**** DRS 1.49f.c = 4.61 cm					
			5 day after BoNT/ 19-day after IoNC			NR		**t6 = 8.647***** DRS 0.97f.c = 4.04 cm	**t6 = 5862****** DRS 0.87f.c = 2.7 cm			
**Muñoz-Lora, 2022 [22]**	**PIH** by systemic immunization - mBSA/PBS+CFA	**Trigeminal nucleus caudalis (TNC)**	Mean ± SEM, one-way ANOVA followed by Tukey's post hoc test	**(n=40) Sprague–Dawley rats, 6–8 weeks, Male (300–400g)**							+ (↑)<br><br>* (↓)<br>p < 0.001 vs PIH + saline	Other assessments - spontaneous nociception by examining RGS, evoked pain by examining facial mechanical allodynia over the skin covering the stimulated TMJ (Frey
				(n=10) control non-immunised + Saline		(n=10) PIH+Saline (NaCl-0.9%)		(n=10) PIH + OnaBoNT 7U	(n=10) PIH + AboBoNT 14U			

	**& TMJ rheumatoid arthritis** by mBSA + formalin (0.5%) intraarticular TMJ injection<br><br>Sham: mBSA i.a.TMJ injection<br>Experimental – unilateral left i.a. TMJ injection, day-42/7days after last challenge of TMJ induction	levels of c-Fos-positive nuclei			NR	ip.l	+ NR				** (↓) p < 0.01 vs PIH + saline	filaments) – behavioural assessments pre-formalin (day-13) + post-formalin (day-14)<br>•**Analysis on c-Fos-expressing neuronal profiles (5 sections/ animal, n=5 animals/Tx group)**<br>•DRS 20 positive nuclei (и)=2.2 cm
						c.l	+ NR					
			14-days after BoNT	ip.l		DRS 5.4cm = 49(и)	F5,24 = 20.1	* DRS 2.74 cm=27.4(и)	** DRS 3.42 cm = 31(и)			
				c.l		DRS 3.8cm =34.5 (и)		** DRS 1.7 cm=15.5(и)	** DRS 1.7 cm=15.5(и)			
**Ni, 2023 [24]**	**Major depressive disorder** by chronic restraint stress (CRS) - induced depression and drug administration.<br><br>pre-injection into the unilateral (WIM) with 3 dosages at 3 different time points prior to the end of the restraint period:<br>i) 6 weeks before to the restrain end/ 3weeks before the restrain start,<br>ii) 3weeks before to the end of restrain/<br>1-day before restrain start,<br>iii) 3days before 21-day restraint stress end.	**Brain (vlPAG)**<br>(expression) - number of c-Fos-positive neurons - . (a) Scale bar, 500µm<br>(b) Scale bar, 200µm; 50 µm<br>(c) Scale bar, 500µm	mean±SEM. one-way ANOVA + Bonferroni' or Dunnett's multiple comparisons test. Two-tailed Student's t-test		**(n=?) pathogen-free C57BL/6J mice, 8 weeks, Male (25g)**						(a)+ (↑)<br>(a)++Naïve+Saline vs. CRS+Saline: P=0.0274<br><br>(b)++ Naïve+Saline vs. CRS+Saline: P=0.0002<br><br>(c)++ Naïve+Saline vs. CRS+Saline: P=0.9102<br><br>(a)* (↓)<br>(a)** P=0.0210<br>(a)*** CRS+Saline vs. CRS+BoNT: P=0.0321<br><br>(b)* (↓)<br>(b)** P=0.0002<br>(b)*** CRS+Saline vs. CRS+BoNT: P=0.0013<br><br>(c)** P=0.4772<br>(c)*** RS+Saline vs. CRS+BoNT: P=0.5721	Other assessments:<br>•monosynaptic retrograde tracer CTB-488 injected alone or CTB-488-mixed BoNT to label the wFMNs:<br>Co-immunostaining for NeuN (neuronal marker) and vAChT, SerT, or vGluT2 (synaptic markers)<br>•injection virus tracer (PRV-EGFP) - to rule out interference from afferent sensory regions + check the circuitry upstream of wFMNs.<br>•Following virus infection, (Ca2+/ CaMKII), GAD67, TH, and TpH2 - to identify the neuron types in the vlPAG that send afferent synapses to the wFMNs; and wFMNs-projecting vlPAG - to ascertain the anatomical synaptic connectivity of vlPAG input to wFMNs.<br>•neuroanatomical tracing, and specific chemogenetic manipulation to evaluate the contribution of the wFMNs-projecting vlPAG neurons to retrograde effects.<br>•behavioural tests (FST) - despair, sucrose preference test – anhedonia, and (OFT) - locomotor ability.<br>▪ n=3 mice per group<br>▪DRS (a) 20 c-fos number in ip.l vlPAG = 1.1cm<br>▪DRS (b) 20 proportion of CaMKII cells among c-fos cells= 1.48cm<br>▪DRS (c) 20 proportion of Gad67 cells among c-fos cells = 2.25cm
					Naïve + saline<br><br>ii) (n=3)	CRS + saline<br><br>ii) (n=3)		CRS + BoNT<br><br>ii) (n=3)<br>10U/Kg		CRS + saline<br><br>ii) (n=3)		
			(a) c-fos		(a)++ DRS 1.82cm = 33.1c-fos(n)	(a)+ DRS 3.24cm = 58.91 c-fos(n)		DRS 1.96cm = 35.64 c-fos(n)		(a)++		
			(b) c-fos + CaMKII		(b)++ DRS 1.34cm= 18.11 CaMKII(p)	DRS 3.3cm = 44.59 CaMKII(p)		DRS 1.85cm = 25 CaMKII(p)		(b)++		
			(c) c-fos + Gad67		(c)++<br>DRS 2.25cm = 20 Gad67(p)	DRS 2.35cm = 20.89 Gad67(p)		DRS 2.67cm = 23.73 Gad67(p)		(c)++		
			24h after day-23 to -27	(a)				(a)*		(a)** F (2, 16)=4.967<br>(a)***		
				(b)				(b)*		(b)** F (2, 16)=15.39<br>(b)***		
				(c)						(c)** F (2, 10)=0.7974<br>(c)***		

**Kim, 2015 [25]**	**Trigeminal nociception** by NMDA<br><br>Injection of BoNT, 3 days before NMDA (i.c. 5µg):<br>(1 or 3U/kg, 30µl) (**s.c**) into the vibrissa pad (peripheral effects);<br>(0.3 or 1U/kg, 10µl) was injected (**i.c**) (central effects).	**medullary dorsal horn – trigeminal neurons** (expression - number of immunoreactive neurons - Scale bar, 100 µm)	means ± SEM.		**(n=?) Sprague-Dawley rats, age unknown, Male (230-280g)**							+ (↑) in the superficial lamina I & II<br><br>* (↓) P<0.05 (vehicle vs. BoNT) - ipsilateral	Other assessments: orofacial nociceptive behavioural responses - intrathecal or intracisternal induced nociceptive behaviour.<br>▪DRS 50 number of c-fos (nC-fos)= 1.95cm
					(n=5) Sham (control)	(n=5) NMDA+ Vehicle	(n=5) s.c BoNT + NMDA		(n=5) i.c. BoNT + NMDA				
					DRS 0cm = 0 (nC-fos)	DRS 6.34cm = 162.6 nC-fos	1U/ Kg	3U /K g	3U/ Kg	1U /K g			
						+ NR	DRS 3.67cm =94.10nC-fos		DRS 3.1cm = 79.49nC-fos				
			?					* NR		* NR			

BIOMARKER: SP

**Shao, 2013 [19]**	**Migraine** by nitroglycerin (NTG)<br><br>Vehicle or BoNT subcutaneous (frontal and temporal) injection 2h after NTG	**Jugular plasma and medulla oblongata - containing caudal trigeminal nucleus** (pg/mL)	means ± SEM. One-way ANOVA. Binomial qualitative data, comparisons between groups (chi-square)		**(n=32) Sprague-Dawley rats, age unknown, Female (250-300g)**				+ (↑) 2.14-fold, P<0.01<br>++ (↑) 3.14-fold, P<0.05<br><br>* (↓) P<0.05<br><br>** (↓) P<0.01<br><br>*** NS P>0.05 (between doses)	•DRS (a) 6pg/ml = 2.54cm<br>•DRS (b) 1pg/ml = 1cm<br>**▪Analysis (n=8)/ group**
					(n=8) Sham (control)	(n=8) NTG + vehicle	(n=8) NTG+ BoNT 5U/kg	(n=8) NTG+ BoNT 10U/kg		
					(a) jugular plasma	+ NR				
					(b) oblongata	++ NR				
			24h after BoNT	(a)	DRS 2.54cm = 6 pg/ml	DRS 5.37cm =12.68 pg/ml	* NR DRS 2.73cm = 6.45 pg/ml	* NR DRS 1.57cm = 3.71 pg/ml		
							***			
				(b)	DRS 1.43cm / pg/ml	DRS 4.4cm / pg/ml	** NR DRS 1.44 pg/ml	** NR DRS 1.55 pg/ml		
							***			

**Muñoz-Lora, 2017 [15]**	**PIH** by systemic immunization - mBSA/PBS+CFA **& TMJ rheumatoid arthritis** by mBSA + formalin (0.5%) intraarticular TMJ injection<br><br>Sham: mBSA i.a.TMJ injection<br>Experimental: i.a.TMJ injection, day-42/7days after last challenge of TMJ induction	**Peri-articular tissues from TMJ and Trigeminal ganglia (TG)** (ng/mL)	ANOVA: Tukey's test	**(n=?) Wistar rats, age unknown, Male (250–500g)**						* (↑) P < 0.05<br><br>** (↓) P < 0.05	Other assessments: behavioural nociceptive tests.<br>•DRS: 0.05ng/ml = 3.09cm<br>• BoNT (3.5;7;14U) ↓ PIH induced by arthritis in the TMJ of rats without differences among groups. Established the dose of the BoNT at 7 U/Kg for the next experiments.
				non-immunised	PIH	PIH+ BoNT 3.5U	PIH+B oNT7 U	PIH+ BoNT 14U	PIH+Saline (NaCl-0.9%)		
				NR DRS 0.59cm = 0.009ng/ml	NR* DRS 6.47cm = 0.105ng/ml				NR* vs sham DRS 7.09cm = 0.115ng/ml		
			24h after BoNT			NR	NR** DRS 1.24cm = 0.020 ng/ml	NR	NR		
			14-days after BoNT			NR	NR** DRS 0.00cm = 0.00 ng/ml	NR	NR		

BIOMARKER: IBA-1

Study	Model / intervention	Tissue	Statistics	(n=12) vs control c.l	(n=12) vs control ip.l	(n=18) TN+vehicle c.l	(n=18) TN+vehicle ip.l	(n=12) TN+BoNT (0.18U) vs vehicle c.l	(n=12) TN+BoNT (0.18U) vs vehicle ip.l	(n=6) TN+ $Tlr2^{-/-}$	Significance	Notes
				(n=48) C57BL/6 mice, 6-8 weeks, Male (20g)								
**Chen, 2021 [14]**	**Trigeminal Neuralgia** modified TN model induced by IoNC - neuropathic pain and anxiety-like behaviors<br><br>BoNT s.c. unilateral peripheral (facial) injection into whisker pad on the ip.l. side of IoNC (2-weeks after IoNC)<br>* 2-weeks after BoNT-analgesic effect worn off (lasted about 9-days), a 2nd injection performed.	**Trigeminal nucleus caudalis (TNC)** (mRNA; protein expression)	mean±SEM. Unpaired Student's t-test. Two-Way ANOVA + post-hoc Bonferroni test.	**	DRS 1f.c / 2.1	**	**t4 = 2.949 *** DRS 1.36f.c / 2.85				* (↑) P=0.0420<br><br>** NS<br><br>*** (↓) P=NR<br><br>**** c.l (↓) vs ip.l P=0.0123	• **Data from 4 mice for statistical analysis.**<br>•DRS 1f.c = 2.1 cm<br>• Bilateral mechanical pain (von Frey test), Hypersensitivity (open field), Anxiety-like behaviour (elevated plus-maze testing), and (depressive-like behaviour) FST: (↓) IoNC-induced bilateral mechanical pain hypersensitivity (appeared after 1h, lasted 9 days) and anxiety-like behaviours (but not depression-like behavior)<br>• Pain behaviours of TN (n=6) CCI+$Tlr2^{-/-}$ to investigate the role of TLR2 on the development of persistent pain.
						c.l vs ip.l **** **t4 = 4.332** DRS c.l (1f.c = 2.1) vs ip.l (1.42f.c =2.98)						
			5 day after BoNT/ 19-day after IoNC			NR	NR	NR	NR***			

BIOMARKER: IL-6

Study	Model / intervention	Tissue	Statistics	(n=12) sham vs control c.l	(n=12) sham vs control ip.l	(n=18) TN+vehicle c.l	(n=18) TN+vehicle ip.l	(n=12) TN+BoNT (0.18U) vs vehicle c.l	(n=12) TN+BoNT (0.18U) vs vehicle ip.l	(n=6) TN+ $Tlr2^{-/-}$	Significance	Notes
				(n=48) C57BL/6 mice, 6-8 weeks, Male (20g)								
**Chen, 2021 [14]**	**Trigeminal Neuralgia** modified TN model induced by IoNC - neuropathic pain and anxiety-like behaviors<br><br>BoNT s.c. unilateral peripheral (facial) injection into whisker pad on the ip.l. of IoNC (2-weeks after IoNC)<br>* 2-weeks after BoNT-analgesic effect worn off (lasted about 9-days), a 2nd injection performed.	**Trigeminal nucleus caudalis (TNC)** mRNA; protein expression	mean±SEM. Unpaired Student's t-test. Two-Way ANOVA + post-hoc Bonferroni test.	NR** DRS 1f.c =3.13 cm	DRS 1f.c = 2.5 cm	NR** DRS 1.27f.c = 3.98 cm	**t6= 5.629*** DRS 1.48f.c = 3.7cm				* (↑) P=0.0013<br><br>** NS<br><br>*** (↓) P=0.0035	•**Data from 4 mice/group were used for statistical analysis.**<br>•DRS (cl) 1f.c = 3.13 cm<br>•DRS (ip.l) 1f.c = 2.5cm.<br>•Bilateral mechanical pain (von Frey test), Hypersensitivity (open field), Anxiety-like behaviour (elevated plus-maze testing), and (depressive-like behaviour) FST: (↓) IoNC-induced bilateral mechanical pain hypersensitivity (appeared after 1h, lasted 9 days) and anxiety-like behaviours (but not depression-like behavior)<br>•Pain behaviours of TN (n=6) CCI+$Tlr2^{-/-}$ to investigate the role of TLR2 on the development of persistent pain.
			5 day after BoNT/ 19-day after IoNC			NR		NR DRS 1.34f.c = 4.21cm	**t6 = 4.657***** DRS 0.78f.c = 1.94cm			

Study	Model / intervention	Tissue	Statistics	sham	naive	TN c.l	TN ip.l	TN + Single BoNT 3U	TN + Single BoNT 1U	TN + Repeated BoNT 3U	TN + Repeated BoNT 1U	TN + vehicle	Significance	Notes
				(n=236) Sprague-Dawley rats, age unknown, Male (250−280 g)										
**Cho, 2022 [17]**	**Trigeminal neuralgia** by compression of the trigeminal nerve root (TNR)<br><br>(1 or 3 U/kg) single BoNT injection (POD-5) or repeated BoNT (POD-12)	**Trigeminal ganglion (TG)** pg/ml tissue; protein concentration	mean ± SEM. Student's t-test and one way ANOVA + Holm-Sidak post hoc	DRS 1.93 cm =	DRS 1.93 cm =	vs sham or naïve *NR							* (↑) (P<0.05)<br><br>**(↓) (P<0.05)	•**Data from 6 mice/group were used.**<br>•DRS (naive, sham, TN): 20pg/ml = 1.5cm.<br>•DRS (vehicle, BoNT): 20pg/ml = 1.77cm.

				25.73 pg/ml	25.73 pg/ml	DRS 4.62cm = 61.6 pg/ml							•Other assessments: Mechanical Allodynia (changes in the air-puff thresholds) and up-regulate IL-1β, IL-6, and TNF-α concentrations in the TG after injection of PX-12, a HIF-1α inhibitor.<br>•Single/double Tx with high BoNT (3 U/kg) led to significantly prolonged antinociceptive effects. Repeated s.c. injections of low dose BoNT (1 U/kg) did not affect the air-puff thresholds.<br>•PX-12, compared to vehicle led to significant anti-allodynic effects and ↓ IL-1β, IL-6, and TNF-α levels.
			Day-2 after BoNT/ POD-7				** NR DRS 3.38 cm = 38.19 pg/ml	NR	NR	NR	DRS 6.59cm = 74.46 pg/ml		
**BIOMARKER:** CX3CR1													
**Muñoz-Lora, 2020 [16]**	**PIH** by systemic immunization - mBSA/PBS+CFA **& TMJ rheumatoid arthritis** by mBSA + formalin (0.5%) intraarticular TMJ injection<br><br>▪Sham: mBSA/PBS+ mBSA i.a.TMJ injection.<br>▪Experimental - unilateral/ ip.l. to immunisation intra-TMJ injection, day-42/7days after last challenge of TMJ induction<br><br>Seven days after the last intra-TMJ injection of the immunization protocol animals were treated with BoNT or vehicle saline. Sterile saline (0.9% NaCl; 20 µl) was injected in non-immunized and immunized groups	**Trigeminal subnucleus caudalis** (OD, protein level)	mean ± standard deviation (SD). one-way ANOVA + post hoc Tukey's test	**(n=40) Wistar rats, age unknown, Male (300–400 g)**								* NS P > 0.05	**• Number of samples for each experimental group = 8.**<br>•DRS: 0.5OD = 1.7cm
				non-immunised+ vehicle saline (0.9% NaCl)		Immunised – PIH (TMJ arthritis) +vehicle saline (0.9% NaCl)	PIH+BoNT (7U/kg)						
				DRS 3.87cm = 1.138OD		NR* DRS 2.95cm = 0.868OD							
			24h after BoNT				NR* DRS 2.77cm = 0.815OD						
			7-days after BoNT				NR* DRS 3.08cm = 0.906OD						
			14-days after BoNT				NR* DRS 2.61cm = 0.768OD						
**Li, 2023 [18]**	**Depression** by reserpine chronic administration **in Parkinson´s disease model.**	**Brain-Substantia nigra pars compacta**	mean±SEM. Two-way ANOVA + Bonferroni's and one-way ANOVA + Tukey's post hoc test	**(n=?) Parkinson disease model-ICR mice, 6–8 weeks old, Male (30g)**								* (↑) (mRNA expression)<br><br>** (↓) P = 0.0054	**•Chemokine receptor CX3CR1 mRNA expression levels in the hippocampal samples were measured (n = 5–6) mice/each group.**
				Control		Reserpine vs. control (no BoNT)	Reserpine + BoNT Vs. reserpine				Control + BoNT		
				DRS mRNA 1.97cm = 1 GAPDH		*NR DRS mRNA							

	From the 10th week, BoNT ($10U/kg^{-1} \cdot d^{-1}$) injected into the cheek once daily for 3 consecutive days.	**(SNpc) & hippocampus** (mRNA expression levels, GAPDH)				3.2cm = 1.62 GAPDH					•DRS (mRNA): 1 GAPDH = 1.97cm<br>•Other assessments: Behavioural test (body weight, Rotarod test, Pole climbing test, OFT, FST, Tail suspension test, Sucrose preference test), In vitro cell culture-CCK-8 assay (mouse BV2 microglial cell line supplemented with reserpine in the presence/absence of BoNT for 24h).<br>•BoNT significantly helped depressive-like behaviours.
			(?) follow-up				mRNA by qRT–PCR<br>Hyppocampal<br>** F (3, 20) = 5.718<br>DRS mRNA<br>2.72cm = 1.38 GAPDH		DRS mRNA<br>1.8cm = 0.91 GAPDH		
**BIOMARKER:** dural protein extravasation											
**Filipović, 2012 [28]**	**Trigeminal neuropathy** by unilateral IoNC<br><br>Saline/BoNT (3.5U/Kg) single unilateral injection 14 days after IoNC, s.c in vibrissal pad (ip.l or c.l). Sham operated - exposing infraorbital nerve without placing silk ligatures around the nerve.<br><br>•(n=8/ 3groups) animals pretreated with saline/BoNT with formalin (trigeminal inflammatory pain) - to assess bilateral dural extravasation, dural tissue from 4 animals was pooled in one sample.	**Cranial dura Plasma protein complexes** (ng of Evans blue per mg of dural tissue)	means ± SEM. ANOVA + Newman-Keuls post hoc test or Tukey's test.		**(n=200) Wistar rats, age unknow, Male (300–350g)**					+ (↑) P<0.01 bilateral dural extravasation (vs sham)<br><br>* (↓) P<0.01 bilateral (vs saline) (lasting more than 2 weeks)	Other measurements: allodynia tested by von Frey filaments; to test the role of axonal transport of BoNT in sensory neurons for its effects on bilateral neuropathic pain and dural extravasation – axonal transport blocker Colchicine (2µl) injected into TG; behavioural test - rubbing time.<br>▪**Each sample consists of combined dural tissue of 4 animals. n=5 samples per group.**<br>•DRS (dura ip.l) 10 ng/mg tissue = 2.24cm<br>•DRS (dura c.l) 10 ng/mg tissue = 2.33cm<br>•Effects of BoNT on pain and dural extravasation in IoNC model were dependent on axonal transport through sensory neurons, as evidenced by colchicine injections (5 mM, 2 µl) into the TG completely preventing BoNT effects.
					(n=20) Sham operated	(n=20) IoNC + saline	(n=20) IoNC + BoNT ip.l	(n=20) IoNC + BoNT c.l	Saline or BoNT + formalin		
					DRS	+ NR DRS			+ NR		
			Dura ipl.l to IoNC (a)		2.24cm = 10ng/mg	8.2cm =36.61ng/mg					
			Dura c.l. to IoNC (b)		2.56cm = 10.99ng/mg	7.4cm =31.76ng/mg					
			3-days after BoNT				* NR	* NR			
				(a)			DRS 2.2cm =9.82ng/mg	DRS 2.85cm = 12.72 ng/mg			
				(b)			DRS 3cm = 12.87ng/mg	DRS 3.8cm = 16.31 ng/mg			
**Lacković, 2016 [20]**	**Trigeminal pain - temporomandibular disorders (inflammatory pain)** by CFA	**Cranial dura tissue** (ng (mg tissue)$^{-1}$)	means ± SEM. t-test for dependent samples. one-way ANOVA + Newman–Keuls post hoc test		**(n=105) Wistar rats, 3–3.5 months old, Male (300–350g)**					+ (↑) P < 0.001 (ip.l. dura was double that c.l. side)<br><br>* (↓) P < 0.001	•Other assessments: behavioural testing (mechanical allodynia) by using von Frey monofilaments, Investigation of the effect of the axonal transport inhibitor, colchicine (7-days after **a.** and **b.**),
					Saline control	CFA+saline (0.9% NaCl) (Vs control)	CFA+BoNT (Vs CFA+saline)		CFA+ sumatriptan (175µgkg^{-1}) 24h after CFA		
					ip.l c.l	ip.l + NR c.l + NR	ip.l	c.l			

	CFA + BoNT injections into left TMJ (3-days prior CFA):<br>**a**. single i.a. ($5Ukg^{-1}$, 20 μL)<br>**b**. single i.g. – left TG via infraorbital foramen ($2Ukg^{-1}$, 2μL)<br>**c**. multiple facial injections at 4 sites outside TMJ - total dose: 5U kg^{-1} divided in 4 equal doses (1.25 Ukg^{-1} / site):<br>(i) bilaterally in forehead above orbital arch<br>(ii) bilaterally into whisker pad.		DRS a. b.	0.47cm = 11.19 ng (mg tissue)$^{-1}$		3cm = 71.43 ng (mg tissue)$^{-1}$	1.24cm = 29.52 ng (mg tissue)$^{-1}$			** Tx did affect DNI	on antinociceptive activity and appearance of clSNAP-25 in dura mater following BoNT injection.<br>**•Analysis of single injections (a., b.) on (n/ group = 5-9)**<br>**•Analysis of multiple injections (c.) on (n/ group = 5-8)**<br>•DRS (a. b.) 20 ng (mg tissue)$^{-1}$ = 0.84cm<br>•DRS (c.) 20 ng (mg tissue)$^{-1}$ = 1.1cm
			DRS c.	0.56 cm = 10.18 ng (mg tissue)$^{-1}$	0.93 cm = 16.91 ng (mg tissue)$^{-1}$	3.56 cm =64.73 ng (mg tissue)$^{-1}$	2.14 cm = 38.91 ng (mg tissue)$^{-1}$				
			4-days after BoNT					ip.l * NR	c.l ** NR		
			DRS a.					0.96cm =22.86 ng (mg tissue)$^{-1}$	1.01cm =24.05 ng (mg tissue)$^{-1}$		
			DRS b.					0.57 cm= 13.57 ng (mg tissue)$^{-1}$	0.62 cm =14.76 ng (mg tissue)$^{-1}$		
			DRS c.					1.15cm = 20.91 ng (mg tissue)$^{-1}$	1.31 cm = 23.81 ng (mg tissue)$^{-1}$		

BIOMARKER: TGF-β1, Collagen (I, III), α-SMA & myosin II proteins

**Wang, 2020 [25]**	**Hypertrophic scar** by lesion/6 wound/per 36 ears (n=216)<br><br>Tx groups (28-days after lesion), directly inject the drug into the scar:<br>BoNT (0.5U, 1.0U, 1.5U, 2.0U) and same dose of hormone-TAC.<br>Scar group/no Tx + (PBS)	**Scar tissue (ear)**<br>(a) protein concentration and expression by GAPDH<br><br>(b) protein concentration and expression by GAPDH<br><br>(c) OD<br><br>(d) proteins, optical density (OD)<br><br>(e) protein concentration and expression by GAPDH	Groups comparison - Student's t-test. Data expressed as mean ± S.D	**(n=18) New Zealand big-ear albino rabbits, gender/age unknown, 2.5~3.5 kg**							(a)+ (↑) P < 0.01<br>(b)+ (↑) P < 0.05<br>(c)+ (↑) P < 0.05<br>(d)+ (↑) P < 0.01<br>(e)+ (↑) P < 0.01<br><br>(a)* (↓) P < 0.01 (vs scar group)<br>(a)** (↓) P < 0.05 (vs 1.5U) – higher ↓ with higher dose.<br>(a)*** NS (vs sham)<br><br>(b)* (↓) P < 0.05 (vs scar group)<br>(b)** NS (vs 2.0U vs sham)<br><br>(c)* (-) NS (vs scar)<br>(c)** (↓) P < 0.05 (vs scar group)<br>(c)*** (↓)	•Other assessments: Thickness of dermis (mm)<br>•DRS (a) 0.3 GAPDH = 0.9cm.<br>•DRS (b) 0.2 GAPDH = 1.05cm<br>•DRS (c) 0.05 OD = 1.24cm<br>•DRS (d) 0.1 OD = 1.91cm<br>•DRS (e) 0.2 GAPDH = 0.9cm
				Healthy skin	(n=12) scar group vs sham	(n=12) BoNT right ear				(n=12) TAC left ear<br>Same doses		
						0.5U	1.0 U	1.5U	2.0 U			
			Collagen<br>(a) COL-I,<br>(b) COL-III	DRS<br>(a) 1.31cm= 0.44 GAPDH<br>(b) 2.2cm= 0.42 GAPDH	NR<br>(a)+ DRS 4.92cm= 1.64 GAPDH<br>(b)+ DRS 4.31cm= 0.82 GAPDH	DRS: 0.5U (a) 3.34 cm = 1.11 GAPDH<br>(b) 3.44 cm= 0.65 GAPDH<br>1U (a) 2.56 cm= 0.85 GAPDH<br>(b) 3.14cm= 0.60 GAPDH<br>1.5U (a) 2.04cm = 0.68 GAPDH<br>(b) 2.6cm= 0.49 GAPDH<br>2U (a) 1.33cm= 0.44 GAPDH<br>(b) 2.2cm = 0.42 GAPDH						
			(c) α-SMA	DRS 4.91cm= 0.20 OD	NR (c)+ DRS 6.43cm= 0.26 OD	DRS 5.85 cm = 0.24 OD	DRS 5.4 cm = 0.2	DRS 5.07 cm = 0.20 OD	DRS 4.15 cm =			

								2 OD		0.17 OD		P < 0.05 (vs 1.5U)<br><br>(d)* (↓)<br>P < 0.01 (vs scar group)<br>(d)* (↓) P < 0.05 (vs 1.5U) - significant changes low- and high-dose BoNT (P < 0.05).<br><br>(e)* (↓) P < 0.01 (vs PBS)<br>NS (vs sham)<br>(e)** NS between high- and low-dose of BoNT.	
			(d) myosin II		DRS 3.51cm= 0.18 OD	NR (d)+ DRS 5.73cm= 0.3 OD	DRS 4.27 cm = 0.22 OD	DRS 3.91 cm = 0.20 OD	DRS 3.6cm = 0.19 OD	DRS 3.03 cm = 0.16 OD			
			(e) TGF-β1		DRS 2.76cm= 0.61 GAPDH	NR (e)+ DRS 4.95cm= 1.1 GAPDH	DRS 3.45 cm = 0.77 GAPDH	DRS 3.4 cm = 0.76	DRS 3.31 cm = 0.74 GAPDH	DRS 2.86 cm = 0.64			
			Day-60 after BoNT	(a)<br>(b)			NR (a)* (b)*	NR (a)* (b)*	NR (a)* (b)*/**	NR (a)*/**/*** (b)*			
				(c)			NR (c)*	NR (c)**	NR (c)**	NR (c)**/***			
				(d)			NR (d)*	NR (d)*	NR (d)*	NR (d)*/**			
				(e)			NR (e)**	NR (e)**	NR (e)**	NR (e)*			
**Xiong, 2023 [30]**	**Hypertrophic scar** by lesion/wound/per ear<br><br>Tx groups 4 weeks after lesion, directly inject the drug into the scar.<br>Scar group/no Tx + (PBS)	**Scar tissue (ear)**<br>protein concentration and expression,<br>(1) gray value (GV)	mean ± standard deviation. Tukey–Kramer and analysis of variance		**(n=24) New Zealand white rabbits, 6 months, Female (3.0~3.3 kg)**							(a)* (↓) p < 0.001<br><br>(b)* (↓) p < 0.01<br><br>(c)* (↓) p < 0.001<br><br>(d)* (↓) p < 0.001	•Other assessments:<br>scar thickness, SEI (hematoxylin and eosin staining). collagen content and alignment (Masson's trichrome staining), fibroblast proliferation.<br>▪DRS GV (1) 0.5GV = 2.4cm<br>▪DRS AOD (a2) 0.2AOD = 2.4cm<br>▪DRS AOD (b2) 0.2AOD = 2.9cm<br>▪DRS AOD (c2) 0.2AOD = 2.4cm
					Healthy skin	(n=6) HS + saline (control group) vs sham	(n=6) HS + BoNT (2U) Vs control				(n=6) HS + fractional CO2 laser (repeated after 2 weeks)<br><br>(n=6) HS+BoNT + fractional CO2 laser		
			Collagen<br>(a) COL-I,<br>(b) COL-III			(a1) DRS 5.93cm = 1.23GV	(a1) DRS 3.37cm = 0.70GV						

		(2) average optical density (AOD)			(a2) DRS 5.36cm = 0.45AOD	(a2) DRS 3.7cm = 0.31AOD			•DRS AOD (d2) 0.2AOD = 1.8cm
					(b1) NR	(b1) NR			
					(b2) DRS 4.96cm = 0.34AOD	(b2) DRS 4.5cm = 0.31AOD			
			(c) α-SMA		(c1) DRS 6.5cm = 1.35GV	(c1) DRS 2.33cm = 0.48GV			
					(c2) DRS 6.35cm = 0.53AOD	(c2) DRS 3.87cm = 0.32AOD			
			(d) TGF-β1		(d1) DRS 5.86cm = 1.22GV	(d1) DRS 2.7cm = 0.56GV			
					(d2) DRS 5.13cm = 0.57AOD	(d2) DRS 2.9cm = 0.32AOD			
			5-weeks after BoNT (a)<br>(b)			(a)* NR<br>(b)* NR			
			(c)			(c)* NR			
			(d)			(d)* NR			
**BIOMARKER:** tyrosine hydroxylase (TH), VGlut2									
**Li, 2023 [18]**	**Depression** by reserpine chronic administration **in Parkinson´s disease model.**<br><br>From the 10th week, BoNT (10U/kg^{-1} ·d^{-1}) injected into the cheek once daily for 3 consecutive days.	**Brain-Substantia nigra pars compacta (SNpc) & hippocampus**<br>(a1) protein expression normalized to GAPDH<br>(a2) TH positive cells/neurons (% of control)<br>(b) puncta density (fold)	mean±SEM. Two-way ANOVA + Bonferroni's; one-way ANOVA + Tukey's post hoc	**(n=?) Parkinson disease model-ICR mice, 6–8 weeks old, Male (30g)**				* (↓) substantia nigra and striatum P<br><br>** (↓) hippocampus<br><br>***(+) reversed the decreased levels but not significantly improvement in activity (protein expression)<br>P = 0.0436<br>**** (+) P = 0.0593 (partially rescued)<br><br>***** (↑) P = 0.0013	**•TH protein expression normalized to GAPDH in the SNpc or % (n = 3) mice for each group; Immunostaining images in hippocampal VGlut 6 images (n=3) mice/each group.**<br>•DRS (Vglut2): 1-fold = 1.68cm<br>•DRS (TH a1): 0.5GAPDH = 1.41cm<br>•DRS (TH a2): 50(%) = 1.45cm<br>•Other assessments: Behavioural test (body weight, Rotarod test, Pole climbing test, OFT, FST, Tail suspension test, Sucrose preference test), In vitro cell culture-CCK-8 assay (mouse BV2 microglial cell line with reserpine in the presence/absence of BoNT for 24h).<br>•BoNT significantly helped depressive-like behaviours.
				Control	Reserpine vs. control (no BoNT)<br><br>* NR	Reserpine + BoNT Vs. reserpine	Control + BoNT		
			TH (a1)	DRS 2.84cm = 1.00GAPDH	DRS 1.84cm = 1.65 GAPDH		(a1) DRS 2.84cm = 1.00 GAPDH<br>(b) DRS 1.83cm = 1.09-fold		
			TH (a2)	DRS 2.91cm = 100.34%	DRS 2.33cm = 80.34%				
			VGlut2 (b)	DRS 1.68cm = 1-fold	** NR DRS 0.95cm = 0.56fold				
			(?) follow-up (a)			(a1) Protein levels - SNpc<br>*** F (3, 8) = 4.316<br>DRS 2.46cm =0.87 GAPDH<br>(a2) Positive cells – SNpc Relative to control<br>****F (3, 8) = 3.767<br>DRS 2.62cm = 90.34%	(a2) DRS 2.75cm = 94.83%		
			(b)			Scale bar = 5 μm<br>Hippocampal CA1 region<br>***** F (3, 20) = 7.739<br>DRS 1.86cm = 1.11-fold			
**BIOMARKER:** TRPA1									
**Wu, 2016 [23]**	**Trigeminal neuropathy** by IoNC	**Brainstem Vc region (caudal**	mean ± SD. One-way ANOVA and	**(n=?) Sprague–Dawley rats, age unknown, Male (220–300g)**				+ (↑) (P <0.05)	•Other assessments: Antinociceptive effect/pain threshold/allodynia tested by Von Frey filaments, motor
				(n=6) Sham + saline +	(n=6) IoNC + saline (vs control)	IoNC + saline + Peripheral BoNT (vs control)	(n=6)		

	BoNT/saline s.c.into whisker pad tissue (ip.l. to<br>the nerve injury) 14 days after the IoNC<br><br>Colchicine or normal saline (2 µl) was injected into the TG (ip.l. to the nerve injury) of anesthetized rat, 12h before BoNT/saline (IoNC + colchicine + saline/ BoNT)	**subnucleus of the spinal trigeminal nucleus)**<br>protein expression - OD	the New-man-Keuls test	saline (IoNC not ligated)	7D	14 D	28 D	(n=6)<br>3U/Kg	(n=6)<br>10 U/kg	IoNC + saline + saline	(day-14 and increased until day-28)<br><br>* (↓) (P<0.05) (in a dose-related manner)	coordination ability by Rota-rod test. Colchicine effects. Quantitative analysis of TRPA1 at various times after IoNC.<br>▪**Analysis (n=6)/group**<br>▪DRS (IoNC vs control saline): 1OD = 1.16cm<br>▪DRS (BoNT vs control): 1OD = 1.15cm<br>•Peripheral application of BoNT (3, 10 U/kg) (↑) pain threshold of IoNC.<br>•Antinociceptive effects reached a max. level at 8 days and remained (↑) until 20 days. The 10 U/kg had better antinociceptive effects than 3 U/kg but NS (P>0.05)<br>•Axonal transport blocker colchicine injection into TG resulted in failure to ↑ the level of clSNAP-25 and in disappearance of the antinociceptive effects of BoNT (10 U/kg) P > 0.05
				DRS 1.6cm = 1.38OD	+ NR DRS:							
					1.8 cm =1.5 OD	5.8 cm = 5 OD	6.3 cm =5.4 OD					
			7-days after BoNT	DRS 1.5 cm = 1.3OD				* NR<br>DRS 3.94 cm =3.43OD	* NR<br>DRS 2.77 cm = 2.41OD	DRS 5.86 cm = 5.09OD		
**Cao, 2017 [33]**	**Chronic dry skin itch models** by AEW - Tx twice daily for 7 days.<br><br>For mRNA and protein levels in DRG: BoNT vs saline (0.1U) single injection into the neck in 3 ways:<br>i) itch model (acute and chronic) – single intradermal injection at neck (0.03, 0.1, 0.3, 1U); **for chronic: 1 day before AEW.**<br>ii) formalin-induced pain model – s.c. single injection into hindpaw (0.1U/mice)<br>iii) compound 48/80-induced itch model – repeatedly injected into neck (0.01, 0.03, 0.1U)	**dorsal root ganglia (DRG)**<br>protein expression–relative densitometry by Tubulin	mean ± SD. unpaired Student´s t-test. One-way/Two-way ANOVA + Bonferroni' post-test.	**(n=525) CD1 (ICR) mice, 6-8 weeks, Male (weight unknown)**							+ (↑) P=0.0003 for 3 days<br><br>++ (↑) P=0.0011 for 7 days<br><br>* (↓) P=0.0018 for 3 days<br><br>** (↓) P=0.0157 for 7 days	Other assessments: behavioural test (scratching behaviour), rota-rod test (motor function)<br>•single injection of BoNT (0.1U) on acute itch mice (↓)TRPV1 and TRPA1 expression at both transcriptional and translational levels in DRG - this effect lasted at least 7-days.<br>•Repeated BoNT (3 times) on acute itch mice (↓) compound 48/80-induced scratching compared with saline, and protein expression of TRPA1 in cervical DRG.<br>•BoNT (0.1U) (↓) the development of chronic itch on days 5 and 7 – anti-pruritic activity under chronic itch conditions.<br>•**Analyses (n=6-7 mice)**<br>▪DRS (1D) 0.2Tubulin = 0.85cm<br>▪DRS (3D) 0.2Tubulin = 0.73cm<br>▪DRS (7D) 0.2Tubulin = 0.85cm
				Sham – treated with water	AEW – chronic model<br>(Vs sham)			AEW+BoNT (0.1U) i)<br>(vs saline)		AEW+Saline		
								i)				
			30min	NR	NR			NR		NR		
			1-day	DRS 1.93cm = 0.45T				DRS 2.15cm = 0.50T		DRS 2.4cm = 0.56T		
			3-days	DRS 1.66cm =0.45T	+ t(10)=5,414			* t(8)=4.571<br>DRS 1.8cm = 0.49T		DRS 2.85cm = 0.78T		
			7-days	DRS 1.7cm = 0.4T	++ t(6)=5,860			** t(7)=3.169<br>DRS 2.4cm = 0.56T		DRS 3.1cm = 0.73T		
			14-days									

BIOMARKER: MMP-13

				(n=42) +3 Wister albino rats, 3-4 months, Male (180 -200g)						
**Makawi, 2022 [13]**	**TMJ osteoarthritis (OA)** induced by monosodium iodoacetate<br><br>Sham – no injection (right)<br>OA - injection left TMJ, BoNT - injection right TMJ	**TMJ tissues** (pg/ml tissue)	Mean (m) , SD values of ANOVA - comparison between groups	(n=3) vs control	(n=42) OA (n=2/group sacrificed)	(n=12) OA+BoNT (5U/kg) treated right side *	(n=12) OA+BoNT (5U/kg) untreated left side	(n=12) PRP<br>(n=12) BoNT + PRP	* (↓) p<0.001	histological analysis, bone area % and joint space (mm) between head of condyle and temporal bone (CBCT):<br>•BoNT-treated samples showed (↓) mean bone area % than other treated groups (p<0.001).<br>•Treated sides recorded ↑ mean joint space than the untreated sides - statistically significant only for BoNT-Tx (p<0.001)
			2 weeks post-Tx	1.26E(m), 0.10(SD)	3.55B(m), 0.25(SD)	2.24C(m), 0.13(SD)	4.16A(m), 0.75(SD)			
			4 weeks post-Tx	1.26G(m), 0.10(SD)	3.55E(m), 0.25(SD)	1.61F(m), 0.06(SD)	6.17B(m), 0.05(SD)			

BIOMARKER: 5-HT, NMDAR subunits, BDNF, p-ERK, p-CREB

						(n=?) ICR mice, 6–8 weeks, Male (20g–25g)								
						Control (naïve)	Spatial restraint stress (SRS) (vs control)	SRS + BoNT ▫ 16 days	SRS + BoNT ▫ 18 days	SRS + BoNT ▫ 22 days	SRS + BoNT ▫ 29 days	Control + BoNT (vs naive) ▪<br>1h, 1-,3-,7-days		
**Li, 2019 [21]**	**Depression** by spatial restraint stress<br><br>BoNT 0.18U single facial intramuscular injection (3 points at each cheek)<br>Imipramine, 10mg/kg, fluoxetine 10 mg/kg injected intraperitoneally daily.	**Hippocampus (i), hypothalamus (ii), prefrontal córtex (iii), amígdala (iv) (Brain)**<br>(a) (ng/g) (using HPLC)<br>(b) by tubulin<br>(c) by tubulin<br>(d) by tubulin<br>(e) (1.mRNA (RT-PCR), 2.protein expression by tubulin (Western blotting analysis)<br>(f) HPLC analysis, RT-PCR, western blotting (GAPDH)<br>(g) HPLC analysis, RT-PCR, western blotting (GAPDH)	mean ± SEM. Student's t-test. One-/Two-way ANOVA+ post Bonferroni	5-HT (a)<br><br>DRS	i	▪ 3.35cm = 270.2ng/g<br>▫ DRS 2cm = 1.61.3ng/g	NR<br>▫ DRS 0.85cm = 68.5ng/g	3.27 cm = 263.7 ng/g	3.28 cm = 264.5 ng/g	2.8 cm = 225.8 ng/g	2.7 cm = 217.7 ng/g	1h NR<br>1D: 3.91cm=3.15.3ng/g<br>3D: 4cm=322.6ng/g<br>7D NR	(a)+ P = 0.0459<br>(a)++ no change<br>(a)* (↓) P = 0.0277<br>(a)** (↓) P = 0.0027<br>(a)*** no change<br>(a)« i (↑) P < 0.0001<br>(a)« ii(↑) P = 0.0007<br>(a)« iii (↑) P = 0.0056<br><br>(b)+ no change<br>(b)* (↓) P = 0.0005<br>(b)« (↑) P<0.0001<br>(c)+ (↓)<br>(c)* no change P=0.4254<br>(d)+ no change<br>(d)* (↓) P = 0.0011<br>(d)« (↑) P = 0.0026<br><br>(e)+ no change<br>(e)++ no change<br>(e)* (↓) P = 0.0057<br>(e)** (↓) P = 0.0146<br>(e)*** (↓) P = 0.0145<br>(e)**** no change<br>(e)« (↑) P<0.0001<br>(e)«« (↑) P = 0.0027<br>(e)««« (↑) P = 0.0048	Other assessments: Behavioural assessments: FST, tail suspension test, sucrose preference test, body weight, rotarod test (motor function), OFT (locomotory activity).<br>•BoNT Tx Improves Depressive-Like Behaviours in naïve mice (n=5–10/group) and in mice undergoing SRS.<br><br>**▪(a) analysis (n=6-7) BoNT+naïve or SRS**<br>**▪(b,c,d) analysis (n=6) BoNT + naïve or SRS**<br>**▪(e1) analysis (n=5-6) BoNT+naïve**<br>**▪(e2) analysis (n=6) BoNT+naïve**<br>**▪(e1) analysis (n=6) BoNT+SRS**<br>**▪(e2) analysis (n=6) BoNT+SRS**<br>**▪(f,g) analysis (n=6) BoNT+SRS**<br><br>• DRS (a) naïve: (i) 200ng/g = 2.48cm; (ii) 200ng/g = 1.24cm; (iii) 200ng/g = 2.05cm<br>• DRS (a) SRS: (i) 200ng/g = 2.48cm; (ii) 200ng/g = 1.24cm; (iii) 200ng/g = 1.23cm<br>• DRS (b) naïve: 0.5Tubulin = 1.87cm<br>• DRS (b) SRS: 1Tubulin = 3.1cm<br>• DRS (c) naïve: 0.5Tubulin = 1.98cm
					ii	▪ 3.33cm = 537.1ng/g<br>▫ DRS 3.47cm = 559.7ng/g	NR<br>▫ DRS 1.7cm = 274.2ng/g	3.6 cm = 580.6 ng/g	2.77 cm = 446.8 ng/g	3.13 cm = 504.8 ng/g	1.4 cm = 225.8 ng/g	1h NR<br>1D: 4.7cm=758.1ng/g<br>3D: 4.22cm=680.6ng/g<br>7D NR		
					iii	▪ 4.3cm = 419.5ng/g<br>▫ DRS 2.3cm = 374.0ng/g	NR<br>▫ DRS 2.24cm = 364.2ng/g	3.5 cm = 569.1 ng/g	2.35 cm = 382.1 ng/g	2.6 cm = 422.8 ng/g	2.1 cm = 341.5 ng/g	1h NR<br>1D: 4.6cm=448.8ng/g<br>3D: 4.5cm=439.0ng/g<br>7D NR		
				NMDAR subunits	NR1 (b)	▪ 3.58cm = 0.96Tubulin<br>▫DRS 2.91 cm= 0.94T	NR<br>▫DRS 1.4cm =0.45T	2.95 cm = 0.95 T	4.07 cm = 1.31 T	3.8 cm = 1.22 T	2.88 cm = 0.93 T	1h: 3.45cm = 0.92T<br>1D: 3.48cm = 0.93T<br>3D: 3.5cm = 0.94T<br>7D: 3.6cm = 0.96T		
					NR2 A (c)	▪ 3.8cm = 0.96T<br>▫DRS 3.12cm = 0.95T	NR<br>▫DRS 2.96cm = 0.90T	2.96 cm =	3.63 cm =	3.06 cm =	3cm = 0.91 T	1h: 3.14cm = 0.79T<br>1D: 3.37cm = 0.85T<br>3D: 3.77cm = 0.95T<br>7D: 4.17cm = 1.05T		

								0.90 T	1.11 T	0.93 T			(e)««««« (↑) transiently P = 0.0042<br>(e)«««««« (↑) transiently P = 0.0078<br><br>(f)* (↓) P = 0.0003 (vs control, naïve)<br>(f)« (↑) P = 0.0007<br><br>(g)* (↓) P = 0.0024 (vs control, naïve)<br>(g)« (↑) P<0.0001	• DRS (c) SRS: 0.5Tubulin = 1.64cm<br>• DRS (d) naïve: 0.5Tubulin = 1.96cm<br>• DRS (d) SRS: 0.5Tubulin = 1.6cm<br>• DRS (e1) naïve: 0.5Tubulin = 1.85cm<br>• DRS (e1) SRS: 0.5 Tubulin = 1.57cm<br>• DRS (e2) naïve: (i) 1mRNA =3.84cm; (ii) 1mRNA = 3.8cm; (iii) 1mRNA = 3.75cm<br>• DRS (e2) SRS: (i) ; (ii); (iii); (iv)<br>• DRS (f) SRS: 0.5Tubulin = 2.14cm<br>• DRS (g) SRS: 0.5 Tubulin = 2cm<br><br>•Motor coordination and locomotor activity was not altered by BoNT, indicating that BoNT has anti-depressive effects in native mice without affecting motor function.
				NR2 B (d)		▪ 3.76cm = 0.96T<br>▫DRS 3.12 cm= 0.975T	NR<br>▫DRS 2cm = 0.625T	3.52 cm = 1.11 T	3.6 cm = 1.12 5T	3.7 cm = 1.16 T	3.18 cm = 0.99 T	1h: 3.71cm = 0.95T<br>1D: 3.56cm = 0.91T<br>3D: 3.65cm = 0.93T<br>7D: 3.7cm = 0.94T		
			BDNF (e)	e1		▪ 3.59cm = 0.97Tubulin<br>▫DRS 2.95 cm= 0.94T	NR<br>▫DRS 2.38cm = 0.76T	3.18 cm = 1.01 T	3.2 cm = 1.02 T	3.47 cm = 1.10 T	3.3 cm = 1.05 T	1h: 4cm = 1.08T<br>1D: 4.13cm = 1.12T<br>3D: 4cm = 1.08T<br>7D: 4.36cm = 1.18T		
				e2	i	▪ 3.84cm = 1mRNA<br>▫DRS 2.41cm = 0.96mRNA	NR<br>▫DRS 1.86cm = 0.74 mRNA	2.94 cm = 1.18 mR NA	3.26 cm = 1.30 mR NA	3.43 cm = 1.37 mR NA	2.23 cm = 0.89 mR NA	1h: NR<br>1D: 3.96cm = 1.03 mRNA<br>3D: 4.23cm = 1.10 mRNA<br>7D: 4.13cm = 1.07 mRNA		
					ii	▪ 3.85cm = 1.01 mRNA<br>▫DRS 1.45 cm= 0.97 mRNA	NR<br>▫DRS 1.14cm = 0.76 mRNA	3.3 cm = 2.2 mR NA	1.87 cm = 1.25 mR NA	2cm = 1.33 mR NA	1.25 cm = 0.83 mR NA	1h: NR<br>1D: 3.7cm = 0.97 mRNA<br>3D: 3.6cm = 0.95 mRNA<br>7D: 3.6cm = 0.95 mRNA		
					iii	▪ 3.67cm = 0.98 mRNA<br>▫DRS 1.7cm = 0.94 mRNA	NR<br>▫DRS 1.55cm = 0.86 mRNA	3.37 cm = 1.87 mR NA	2.17 cm = 1.20 mR NA	2.37 cm = 1.32 mR NA	1.5 cm = 0.83 mR NA	1h: NR<br>1D: 3.94cm = 1.05 mRNA<br>3D: 3.98cm = 1.06 mRNA<br>7D: 3.69cm = 0.984 mRNA		
					iv	▪ NR<br>▫DRS 1.1cm = 1 mRNA	NR<br>▫DRS 0.77cm = 0.7 mRNA	2.85 cm = 2.59 mR NA	2.9 cm = 2.63 mR NA	2.9 cm = 2.63 mR NA	2.5 cm = 2.27 mR NA	1h: NR<br>1D: NR<br>3D: NR<br>7D: NR		
			p-ERK (f)			▪ NR<br>▫DRS 4.3cm = 1T	NR<br>▫DRS 2.76cm = 0.64T	4.6 cm = 1.07 T	3.82 cm = 0.89 T	3.1 cm = 0.72 T	2.9 cm = 0.68 T	NR		
			p-CREB (g)			▪ NR<br>▫DRS 3.83cm =0.96T	NR<br>▫DRS 2.75cm = 0.69T	4.45 cm = 1.11 T	3.47 cm = 0.87 T	2.56 cm = 0.64 T	2.1 cm = 0.6T	NR		
			1-day+	(a)		(a)+ ii	(a)* i (t = 3.383)<br>(a)** ii (t = 6.596)	(a)« i F(4, 10) = 21.46<br>(a)« ii F(4, 10) = 12.29				(a)+ F(2, 15) = 3.811		

			16-,18-<br>,22-, 29-<br>days*	(n=6-<br>7)	(a)++ i, iii<br>(n = 6–7)	(a)*** iii NR	(a)« iii F(4, 10) = 7.101	(a)++ NR		
			1h,<br>1-,3-,7-<br>days+<br><br>16-,18-<br>,22-, 29-<br>days*	(b)	(b)+ i	(b)* i<br>(t = 5.102)	(b)« i (n=6) F(5, 30) = 15.50	(b)+ NR		
				(c)	(c)+ i<br>(n = 6)	(c)* i (t =0.8309)		(c)+ F(4, 25) = 11.74		
				(d)	(d)+ i	(d)* i (t = 4.529)	(d)« i (n=6)<br>F(5, 30) = 4.758	(d)+ NR		
			1h,<br>1-,3-,7-<br>days+<br><br>16-,18-<br>,22-, 29-<br>days*	(e)	(e)+Protein<br>(n=6) i	(e)* i (t = 3.500)	(e)« i F(5, 30) = 10.37	(e)+ NR		
			1-,3-,7-<br>days+<br><br>16-,18-<br>,22-, 29-<br>days*		(e)++mRNA<br>(n=5-6) i, ii,<br>iii	(e)** i (t = 4.117)<br>(e)*** iv<br>(t = 4.129)<br>(e)**** iii, ii	(e)«« i<br>(day-1-7) F(4, 10) = 8.700<br>(e)««« iv<br>(day-1-14) F(4, 10) = 7.423<br>(e)«««« iii F(4, 10)= 7.727<br>(e)««««« ii F(4, 10)= 6.459	(e)++ NR		
			16-,18-<br>,22-, 29-<br>days*	(f)		(f)* i (t = 5.350)	(f)« (day-1)<br>F(5, 30) = 5.810			
				(g)		(g)* i (t = 4.034)	(g) (day-1) F(5, 30) = 8.619			

BIOMARKER: Toll-like receptors (TLR1, TLR2, TLR5, TLR4, TLR8, TLR11, MyD88), CD11b, F4/80

**Chen, 2021 [14]**	**Trigeminal Neuralgia**<br>modified TN model induced by IoNC - neuropathic pain and anxiety-like behaviors<br><br>BoNT s.c. unilateral peripheral (facial) injection into whisker pad on the ip.l. side of IoNC (2-weeks after IoNC)<br>* 2-weeks after BoNT-analgesic effect worn off (lasted about 9-days), a 2nd injection performed.	**Trigeminal nucleus caudalis (TNC)**<br>mRNA; protein expression - fold change	mean±SEM.<br>Student's t-test, Two-Way ANOVA+posthoc Bonferroni test	**(n=48) C57BL/6 mice, 6-8 weeks, Male (20g)**							(a)* (↑) P=0.0007<br>(b)* (↑) P=0.0003<br>(e)* (↓) P < 0.0001<br>(f)* (↑) P=0.0347<br>(g)* (↑) P=0.0076<br>(h)* (↑) P=0.0351<br><br>(X) NS<br><br>^ P=0.3842<br><br>(a)+ (↓) P=0.0009<br>(b)+ (↓) P=0.0005<br>(c)+ (↓) P=0.0001<br>(d)+ (↓) P=0.0012	**• Data from 4 mice/group were used for statistical analysis.**<br>•DRS (a) (c.l): 1f.c / 4.16cm<br>•DRS (a) (ip.l): 1f.c / 4.12cm<br>•DRS (b) (c.l): 1f.c / 4.16cm<br>•DRS (b) (ip.l): 1f.c / 4.13cm<br>•DRS (c) (c.l): 1f.c / 4.16cm<br>•DRS (c) (ip.l): 1f.c / 4.13cm<br>•DRS (d) (c.l): 1f.c / 4.16cm<br>•DRS (d) (ip.l): 1f.c / 4.13cm<br>•DRS (e) (c.l): 1f.c / 4.16cm<br>•DRS (e) (ip.l): 1f.c / 4.13cm<br>•DRS (f) (c.l): 1f.c / 2.97cm<br>•DRS (f) (ip.l): 1f.c / 1.1cm<br>•DRS (g) (c.l): 1f.c / 4.16cm
				(n=12) sham vs control		(n=18) TN+vehicle		(n=12) TN+BoNT (0.18U) vs vehicle		(n=6) TN+ *Tlr2*$^{-/-}$		
				c.l	ip.l	c.l	ip.l	c.l	ip.l			
			TLR2 (a)	(X) NR DRS 1f.c / 4.16	DRS 1f.c / 4.12	(X) NR DRS 1f.c / 4.16	(a)* **t6 = 6.307** DRS 1.36f.c / 5.6					
			TLR5 (b)	(X) NR DRS 1f.c / 4.16	DRS 1f.c / 4.13	(X) NR DRS 0.92f.c / 3.83	(b)* **t6 = 7.584** DRS 1.31f.c / 5.45					

			TLR4 (c)		(X)<br>NR<br>DRS<br>1f.c /<br>4.16	DRS<br>1f.c /<br>4.13	(X)<br>NR<br>DRS<br>0.89f.c<br>/ 4.12	(X) NR<br>DRS<br>1.09f.c /<br>4.5				(i)+ (↓) P=0.0146<br>(h)+ (↓) P = 0.003<br>(g)+ (↓) P=0.0025<br>(f)+ (↓) P=0.0378<br><br>- (↓) P=0.0004<br>-- (↓) P=0.0356<br>--- (↓) P=0.0006<br>---- P=0.5831	•DRS (g) (ip.l): 1f.c / 3.1cm<br>•DRS (h) (c.l): 1f.c / 4.16cm<br>•DRS (h) (ip.l): 1f.c / 3.3cm<br>•DRS (i) (c.l): 1f.c / 4.16cm<br>•DRS (i) (ip.l): 1f.c / 4.12cm<br><br>• Bilateral mechanical pain (von Frey test), Hypersensitivity OFT, Anxiety-like behaviour (elevated plus-maze testing), and (depressive-like behaviour) FST: (↓) IoNC-induced bilateral mechanical pain hypersensitivity (appeared after 1h, lasted 9 days) and anxiety-like behaviours (but not depression-like behavior)<br>•Pain behaviours of TN (n=6) CCI+*Tlr2*$^{-/-}$ to investigate the role of TLR2 on the development of persistent pain.
			TLR8 (d)		(X)<br>NR<br>DRS<br>1f.c /<br>4.16	DRS<br>1f.c /<br>4.13	(X)<br>NR<br>DRS<br>1.08f.c<br>/ 4.49	(X) NR<br>DRS<br>1.04f.c /<br>4.29					
			TLR11 (e)		(X)<br>NR<br>DRS<br>1f.c /<br>4.16	DRS<br>1f.c /<br>4.13	(X)<br>NR<br>DRS<br>1.09f.c<br>/ 4.54	(e)* **t6<br>= 10.8**<br>DRS<br>0.63f.c /<br>2.58					
			MyD88 (f)		^ (X)<br>DRS<br>1f.c /<br>2.97	DRS<br>1f.c /<br>1.1	^ (X)<br>**T6 =<br>0.9386**<br>DRS<br>1.12f.c<br>/ 3.33	(f)* **t6 =<br>2.718**<br>DRS<br>2.54f.c /<br>2.79					
			CD11b (g)		NR<br>DRS<br>1f.c /<br>4.16	DRS<br>1f.c /<br>3.1	NR<br>DRS<br>0.98f.c<br>/ 4.1	(g)*<br>**t6 =<br>0.3945**<br>DRS<br>1.26f.c /<br>3.92					
			F4/80 (h)		NR<br>DRS<br>1f.c /<br>4.16	DRS<br>1f.c /<br>3.1	NR<br>DRS<br>0.96f.c<br>/ 4	(h)*<br>**T6 =<br>2.965**<br>DRS<br>1.21f.c /<br>3.77					
			TLR1 (i)		(X)<br>NR<br>DRS<br>1f.c /<br>4.16	(X)<br>NR<br>DRS<br>1f.c /<br>4.12	(X)<br>NR<br>DRS<br>1.18f.c<br>/ 4.69	(X) NR<br>DRS<br>1.13f.c /<br>4.88					
			5 day<br>after<br>BoNT/<br>19-day<br>after<br>IoNC	(a)			NR		NR<br>DRS<br>0.89f.c /<br>3.69	(a)+<br>**t6 = 6.058**<br>DRS<br>0.80f.c / 3.3			
				(b)			NR		NR	(b)+<br>**t6 = 6.897**			

									DRS<br>0.77f.c /<br>3.19	DRS<br>1.01f.c /<br>4.19			
				(c)					-<br>**t6 = 6.963**<br>DRS<br>0.65f.c /<br>2.69	(c)+<br>**t6 = 8.721**<br>DRS<br>0.71f.c /<br>2.96			
				(d)					--<br>**t6 = 2.7**<br>DRS<br>0.85f.c /<br>3.56	(d)+<br>**t6 = 5.766**<br>DRS<br>0.78f.c /<br>3.24			
				(e)			NR	NR	NR<br>DRS<br>1.10f.c /<br>4.59	NR<br>DRS<br>0.78f.c /<br>3.24			
				(f)					(X) ----<br>**t6 = 0.5799**<br>DRS<br>1.01f.c / 3	(f)+<br>**t6 = 2.654**<br>DRS<br>1.03f.c /<br>1.13			
				(g)					NR<br>DRS<br>0.91f.c /<br>3.81	(g)+ **t6 =<br>4.994**<br>DRS<br>0.95f.c /<br>2.95			
				(h)					NR<br>DRS<br>0.77f.c /<br>3.19	(h)+<br>**t6 = 4.799**<br>DRS<br>0.80f.c /<br>2.49			
				(i)					---<br>**t6 = 6.614**<br>DRS<br>0.83f.c /<br>3.44	(i)+<br>**T6 = 3.393**<br>DRS<br>0.97f.c / 4			
**BIOMARKER:** IL-4, Mast Cell count, Total IgE													
**Han, 2017 [27]**	**Atopic Dermatitis** by NC/Nga + contact sensitizer (2-Chloro-1,3,5-trinitrobenzene - TNCB)	**Rostral dorsal Skin**<br>(a) (a1) mRNA; (a2) protein expression (ng/mL)	mean ± SEM. One -Way ANOVA + Tukey. Bonferroni correction		**(n=42) NC/Nga mice, 6weeks, female, weigh unknown**							(a1)+ p=0.000 (vs TNCB)<br>(a2)+ p=0.000 (vs TNCB)<br>(a1)++ (↑) p=0.000 (on day-14, vs control)	Other assessments: skin thickness, transepidermal water loss (TEWL), skin severity scores, histological/ laboratory tests.<br><br>•BoNT failed to supress TNCB-induced IgE serum levels.
					(n=6) untreated control		(n=6) TNCB (vs control)+		TNCB + BoNT		(n=6) TNCB + vehicle [0.9% saline]<br>(n=6) TNCB + 0.03% tacrolimus		
									(n=9) 30 U/kg	(n=9) 60 U/kg			

	BoNT - single intradermal injections on the rostral back on the day of TNCB sensitization. Test area limited to 1.5x1.5 cm)	(b) count (5 high power fields)<br><br>**Serum – retro orbital plexus**<br>(c) total (ng/mL)	IL-4 (a)	(a1)	(a1)+<br>0.90 ± 0.36	(a1)++<br>174±44.9					(a2)++ (↑) p=0.000 (on day-14, vs control)<br>(b)+ p=0.000 (vs TNCB)<br>(b)++ p=0.000<br>(c)+ p=0.000 (vs TNCB)<br>(c) ++ (↑) p=0.000 (on day-14, vs control)<br><br>(a1)* (↓) p=0.004 (vs control), p=0.000 (vs TNCB)<br>(a1)** (↓) p=0.028 (vs control), p=0.000 (vs TNCB)<br>(a1)*** p=0.000 (vs control), p=1.000 (vs TNCB)<br><br>(a2)* (↓) p=0.44 (vs control), p=0.000 (vs TNCB)<br>(a2)** (↓) p=0.148 (vs control), p=0.000 (vs TNCB)<br>(a2)*** p=0.000 (vs. control), p=1.000 (vs TNCB)<br><br>(b)* (↓) p=0.004 (vs control), p=0.000 (vs TNCB)<br>(b)** (↓) p=1.000 (vs control), p=0.000 (vs TNCB)<br>(b)*** p=0.000 (vs control) p=0.366 (vs TNCB)<br><br>(c)* NS p=0.011 (vs control), p=0.765 (vs TNCB)	
				(a2)	(a2)+<br>1.00±0.84	(a2)++<br>69.3±13.4						
			Mast cell (b)		(b)+<br>13.4±3.65	(b)++<br>85.3 ± 7.55						
			IgE (c)		(c)+<br>2.93±1.76	(c)++ 103±27.4						
			day-14 after 1st challenge	(a)			(a1)*<br>53.0±17.6	(a1)**<br>44.3±7.38	(a1) ***<br>161 ± 27.4			
							(a2)*<br>20.2±4.84	(a2)**<br>17.4±5.72	(a2) ***<br>67.6 ± 16.6			
				(b)			(b)* 28.1 ± 4.70	(b)**<br>17.7±2.69	(b)***7 6.5 ± 8.64			
				(c)			(c)* 68.7 ± 13.5	(c)**<br>66.5±29.7	(c)***<br>112 ± 46.5			

											(c)** NS p=0.15 (vs control) p=0.573 (vs TNCB)<br>(c)*** p=0.000 (vs control), p=1.000 (vs TNCB)	
**BIOMARKER:** GFAP												
**Muñoz-Lora, 2022 [22]**	**PIH** by systemic immunization - mBSA/PBS+CFA<br>**& TMJ rheumatoid arthritis** by mBSA + formalin (0.5%) intraarticular TMJ injection<br><br>Sham: mBSA i.a.TMJ injection<br>Experimental: unilateral left i.a.TMJ injection, day-42/7days after last challenge of TMJ induction)	**Trigeminal nucleus caudalis (TNC)** (surface area (µm²) immunoreactivity and mean gray intensity)	Mean ± SE, one-way ANOVA followed by Tukey's multiple comparisons test		**(n=40) Sprague–Dawley rats, 6–8 weeks, Male (300–400g)**						+ (↑) P < 0.001 (vs sham)<br>++ (↑) P < 0.05 (vs sham)<br><br>* (↓) P<0.001 (vs placebo)<br>** (↓) P<0.05 (vs placebo)	Other assessments -spontaneous nociception by examining RGS, evoked pain by examining facial mechanical allodynia over the skin covering the stimulated TMJ (Frey filaments) – behavioural assessments pre-formalin (day-13) + post-formalin (day-14)<br>**•analysis was performed on 5 randomly selected slices per animal (n=5 animals/ group)**<br>•DRS (surface area): 50000= 2cm<br>•DRS (mean gray value): 20 GV = 2cm
					(n=10) control non-immunised + Saline	(n=10) PIH+Saline (NaCl-0.9%)	(n=10) PIH + OnaBoNT 7U	(n=10) PIH + AboBoNT 14U				
						Area (A) / gray value (GV)	A / GV	A / GV				
			ip.l		(A) DRS 0.76 cm =19000µm²<br>(GV) DRS 4.2 cm= 42 GV	+ NR DRS 4.06cm =101500 µm² / ++ NR DRS 5.64cm = 56.4 GV	DRS 0.55 cm= 13750 µm² / DRS 3.09 cm = 30.9 GV	DRS 1.22 cm = 30500 µm² / DRS 4.2 cm = 42 GV				
			c.l			NR / NR						
			14-days after BoNT	ip.l		($F_{3,16}$ (GFAP area) = 73.14; $F_{3,16}$ (GFAP gray value) = 23.21	* NR / * NR	* NR / ** NR				
				c.l			NR / NR	NR / NR				
**BIOMARKER:** glutamate												
**Muñoz-Lora, 2017 [15]**	**PIH** by systemic immunization - mBSA/PBS+CFA<br>**& TMJ rheumatoid arthritis** by mBSA + formalin (0.5%) intraarticular TMJ injection<br><br>Sham: mBSA i.a.TMJ injection<br>Experimental: intra-TMJ injection, day-42/7days after last challenge of TMJ induction	**Peri-articular tissues from TMJ and Trigeminal ganglia (TG)** (nmol)	ANOVA: Tukey's test		**(n=?) Wistar rats, age unknown, Male (250–500g)**						* NS P > 0.05	Other assessments: behavioural nociceptive tests.<br>•DRS: 2nmol = 1.91cm<br>• BoNT (3.5;7;14U) ↓ PIH induced by arthritis in the TMJ of rats without differences among groups. Established the dose of the BoNT at 7 U/Kg for the next experiments.
					non-immunised	PIH	PIH+ BoNT 3.5U / PIH+BoNT7 U	PIH+ BoNT 14U	PIH+Saline (NaCl-0.9%)			
					NR<br>DRS 2.46cm = 2.57nmol	NR*<br>DRS 4.76cm = 4.98nmol			NR* vs sham<br>DRS 4.05cm = 4.24nmol			
			24h after BoNT				NR / NR* DRS 3.44cm = 3.60 nmol	NR	NR			
			14-days after BoNT				NR / NR* DRS 3.35cm = 3.51 nmol	NR	NR			

**BIOMARKER:** P2X7, Cathepsin S/Fractalkine											
**Muñoz-Lora, 2020 [16]**	**PIH** by systemic immunization - mBSA/PBS+CFA **& TMJ rheumatoid arthritis** by mBSA + formalin (0.5%) intraarticular TMJ injection<br><br>▪Sham: mBSA/PBS+ mBSA i.a.TMJ injection.<br>▪Experimental - unilateral/ ip.l. to immunisation intra-TMJ injection, day-42/7days after last challenge of TMJ induction<br><br>Seven days after the last intra-TMJ injection of the immunization protocol animals were treated with BoNT or vehicle saline. Sterile saline (0.9% NaCl; 20 µl) was injected in non-immunized and immunized groups	**Trigeminal subnucleus caudalis** (pg/mL) (a,b) (OD, protein level) (c)	mean ± SD. One-way ANOVA + post hoc Tukey's test		**(n=40) Wistar rats, age unknown, Male (300–400 g)**					* (↑) P < 0.05<br>** NS P > 0.05<br><br>*** (↓) P < 0.05	• **Number of samples for each experimental group = 8.**<br>• DRS (FKN): 500pg/ml = 1.58cm<br>• DRS (CatS): 500pg/ml = 1.75cm<br>• DRS (P2X7): 1OD = 2.43cm
					non-immunised+ vehicle saline (0.9% NaCl)	Immunised – PHI (TMJ arthritis) +vehicle saline (0.9% NaCl)	PHI+BoNT (7U/kg)				
			FKN (a)		DRS 1.84cm = 582.28pg/ml	NR* DRS 3.31cm = 1047.47pg/ml					
			CatS (b)		DRS 0.85cm = 242.86pg/ml	NR* DRS 3.14cm = 897.14pg/ml					
			P2X7 (c)		DRS 2.47cm = 1.016OD	NR* DRS 3.68cm = 1.514OD					
			24h after BoNT	(a)			NR** vs sham DRS 3.35cm = 1060.13pg/ml				
				(b)			NR** vs sham DRS 2.82cm = 805.71pg/ml				
				(c)			NR*** vs placebo DRS 3.12cm = 1.284OD				
			7-days after BoNT	(a)			NR*** vs placebo DRS 2.12cm = 670.89pg/ml				
				(b)			NR**vs sham DRS 3.27cm = 934.28pg/ml				
				(c)			NR*** vs placebo DRS 3.03cm = 1.247OD				
			14-days after BoNT	(a)			NR*** vs placebo DRS 1.83cm = 579.11pg/ml				
				(b)			NR*** vs placebo DRS 1.84cm = 194.28pg/ml				
				(c)			NR*** vs placebo DRS 3.07cm = 1.263OD				
**BIOMARKER:** IB4 (+) and IB4 (-) neurons / FM4-64											
**Kitamura, 2009 [29]**	**Trigeminal neuropathy** by IoNC	**Somata of trigeminal ganglion**	means ± SEM. Differences in group means by		**(n=?) Sprague–Dawley rats, age unknown, Male (200–250g)**					+ (↑) faster onset of KCL-evoked vesicular release.	Other assessments: sensory testing, neuropathy behaviours (head withdrawal thresholds), in vitro pre-treatment with BoNT, dissociation
					Sham - saline injected (c.l.)	IoNC (ip.l.) (Vs sham-c.l.)	IoNC + BoNT (ip.l.) (vs IoNC + saline)	IoNC + saline (ip.l.)			

	Day-3 after unilateral IoNC: single peripheral injection of saline or BoNT (100pg in 0.1ml of sterile saline) in the centre of the whisker pad/ ip.l. side facial skin<br>All rats were injected intradermally with saline on the c.l side (sham).	**(TRG) sensory neurons**<br>(isolated from the side of the injury)<br>onset and rate of transmitter release – time (s)<br>(KCl-evoked vesicular release of FM4-64 -membrane-uptake marker)	RM ANOVA on ranks or t-test.						« (↑) larger maximal release.<br><br>(a)+ (r) of FM4-64 signal during KCl application.<br>(a)++ (↓) (r) without affecting onset.<br><br>(b)+ (r) of FM4-64 signal during KCl application.<br>(b)++ (↓) (r) without affecting onset.<br><br>(a)* (↓) KCL-evoked vesicular release<br>(a)** (↓) slower onset of KCL-induced FM4-64 release<br><br>(b)* (↓) KCL-evoked vesicular release<br>(b)** (↓) slower onset of KCL-induced FM4-64 release	of TRG neurons, FM4-64 dye staining, confocal imaging.<br>•peripheral injection BoNT in neuropathic rats ↓ neuropathic pain behaviours by ↓ exaggerated neurotransmitter vesicular release from TRG sensory neurons (in both IB4 (+) and IB4 (−) neurons)<br>•IB4 (+) and IB4 (-) neurons ip.l. to IoNC exhibited a profoundly faster onset of KCl-evoked vesicular release of FM4-64 compared to neurons isolated from c.l. TRG.
			IB4 + (a)		(a)+ (n=11)<br>26.4±8.1 s	+ (n=8)<br>(a)++ (3.1±1.0 s)	(n=9)	(n=8)		
			IB4 – (b)		(b)+ (n=10)<br>11.1±2.6 s	+ « (n=8)<br>(b)++ (4.2±0.9 s)	(n=9)	(n=8)		
			11-days	(a)			(a)*<br>NR			
							(a)**<br>NR			
				(b)			(b)*<br>NR			
							(b)**<br>NR			
**BIOMARKER:** Inflammatory cells – lymphocyte, monocyte, neutrophile, plasma cells										
**Lacković, 2016 [20]**	**Trigeminal pain - TMDs (inflammatory pain)** by CFA<br><br>CFA + BoNT injections into left TMJ (3-days prior CFA):<br>**a**. single i.a. (5Ukg^{-1},20 µL)<br>**b**. single i.g. – left TG via infraorbital foramen<br>(2U kg^{-1},2µL)<br>**c**. multiple facial injections at 4 sites outside TMJ - total dose: 5U kg^{-1} divided in 4 equal doses (1.25 U kg^{-1} / site):	**Cranial dura tissue (ipsilateral)**<br>(by Giemsa-staining - number of Giemsa positive profiles - Scale bars = 100 µm)	means ± SEM. t-test for dependent samples. one-way ANOVA + Newman–Keuls post hoc test		**(n=105) Wistar rats, 3–3.5 months old, Male (300–350g)**				+ (↑) P < 0.001<br><br>* (↓) P < 0.001<br><br>** (↓) P < 0.05	•Other assessments: behavioural testing (mechanical allodynia) by using von Frey monofilaments, Investigation of the effect of the axonal transport inhibitor, colchicine (7-days after **a.** and **b.**), on antinociceptive activity and appearance of clSNAP-25 in dura mater following BoNT injection.<br>•**Mean of 4–5 visual fields per single animal. Analysis on (n/ group = 5)**<br>•DRS 200 Gpp (number of Giemsa-stained cell profiles) = 1.25cm
					Saline control	CFA+saline (0.9% NaCl) (Vs control)	CFA+BoNT 5Ukg^{-1} a. intraarticular (Vs CFA+saline)	CFA+ sumatriptan (175µgkg^{-1}) 24h after CFA		
					DRS 0.45cm = 72 Gpp	+ NR DRS 4.16cm = 665.6 Gpp				
			4-days after BoNT				* NR<br>** NR (vs saline control)<br><br>DRS 1.4cm = 224 Gpp			

	(i) bilaterally in forehead above orbital arch<br>(ii) bilaterally into whisker pad.								•Lack of polymorphonuclear neutrophils in dura suggests the presence of sterile inflammation.
**BIOMARKER:** fibroblasts									
**Wang, 2020 [25]**	**Hypertrophic scar** by lesion/6 wound/per ear (n=216)<br><br>Tx groups (28-days after lesion), directly inject the drug into the scar:<br>BoNT (0.5U, 1.0U, 1.5U, 2.0U) and same dose of hormone-TAC.<br>Scar group/no Tx + (PBS)	**Scar tissue (ear)**<br>(%) Apoptosis rates	mean ± S.D. Groups comparison - Student's t-test.	**(n=18) New Zealand big-ear albino rabbits, gender/age unknown, 2.5~3.5 kg**				* (↑) P<0.05 (vs scar)<br>** (↓) P < 0.01<br>*** higher (↑) with higher doses	•Other assessments: Thickness of dermis (mm)<br>•DRS 10% = 2.5cm
				Healthy skin	(n=12) scar group	(n=12) BoNT right ear<br>0.5U | 1.0 U | 1.5U | 2.0 U	(n=12) TAC left ear<br>Same doses		
				NR ** DRS 1.1cm = 4.4%	NR DRS 2.53cm= 10.12%	DRS: (0.5U) 2.9cm=11.6%<br>(1U) 3.3cm=13.2%<br>(1.5U) 3.43cm =13.72%<br>(2U) 4.54cm = 18.16%			
			Day-28 after BoNT	NR**		NR * | NR * | NR * | NR */***			
**BIOMARKER:** HIF-1α									
**Cho, 2022 [17]**	**Trigeminal neuralgia** by compression of the trigeminal nerve root (TNR)<br><br>(1 or 3 U/kg) single BoNT injection (POD-5) or repeated BoNT (POD-12)	**Trigeminal ganglion (TG)**<br>pg/ml tissue; protein expression	mean ± SEM. Student's t-test and one way ANOVA + Holm-Sidak post hoc	**(n=236) Sprague-Dawley rats, age unknown, Male (250−280 g)**				* NS<br><br>** (↑) (P<0.05)<br><br>*** (↓) (P<0.05)	**•Data from 6 mice/group were used.**<br>•DRS (naive, sham, TN): 0.5pg/ml = 1.35cm.<br>•DRS (vehicle, BoNT): 0.5pg/ml = 1.33cm.<br>•Other assessments: Mechanical Allodynia (changes in the air-puff thresholds) and up-regulate IL-1β, IL-6, and TNF-α concentrations in the TG after injection of PX-12, a HIF-1α inhibitor.<br>•Single/double Tx with high BoNT (3U/kg) led to significantly prolonged antinociceptive effects. Repeated s.c. injections of low dose BoNT (1 U/kg) did not affect the air-puff thresholds.<br>•PX-12, compared to vehicle led to significant anti-allodynic effects and ↓ IL-1β, IL-6, and TNF-α levels.
				sham | naive	TN<br>vs sham or naïve<br>c.l | ip.l	TN + Single BoNT: 3U | 1U<br>TN + Repeated BoNT: 3U | 1U	TN + vehicle		
				sham: DRS 1.37 cm = 0.51 pg/ml<br>naive: DRS 1.1cm = 0.41 pg/ml	c.l: * NR | ip.l: **NR<br>DRS 2.65 cm = 0.98 pg/ml				
			Day-2 after BoNT/ POD-7			TN + Single BoNT: 3U: *** NR DRS 1.81 cm = 0.68 pg/ml | 1U: NR<br>TN + Repeated BoNT: 3U: NR | 1U: NR	DRS 2.84 cm = 1.07 pg/ml		
**BIOMARKER:** CD68+IBA-1, fractalkine/CX3CL1, C3, C1q, C3aR, PSD95, VGlut2 & PSD95 + dendritic spines from CA1 pyramidal neurons + C3 and C1q, VGAT, VGAT+ Gephyrin									
**Li, 2023 [18]**	**Depression** by reserpine chronic administration **in**	**Brain-Substantia**	mean±SEM. Two-way ANOVA + Bonferroni's &	**(n=?) Parkinson disease model-ICR mice, 6–8 weeks old, Male (30g)**				(a) + (↓)<br>*P = 0.0239<br>**P = 0.0279	**•Dopamine concentration (n=3–6 mice/each group); % of CD68+ lysosome volume in Iba1+**
				Control	Reserpine vs. control (no BoNT)	Reserpine + BoNT Vs. reserpine	Control + BoNT		

	**Parkinson´s disease model.**<br><br>From the 10th week, BoNT (10U/kg^{-1} ·d^{-1}) injected into the cheek once daily for 3 consecutive days.	**nigra pars compacta (SNpc) & hippocampus**<br>(a) ng/g (protein []) by HPLC; pg/g by ELISA<br>(b) occupancy %<br>(c) mRNA expression normalised to GAPDH<br>(d1) mRNA by qRT–PCR normalised to GAPDH<br>(d2) protein normalized to Tubulin (expression),<br>(d3) deposits by immunohistochemistry scale bar=5µm (activation)<br>(e1) mRNA by qRT–PCR normalised to GAPDH,<br>(e2) protein normalized to Tubulin (expression),<br>(e3) deposits by immunohistochemistry - scale bar =5µm (activation)<br>(f) mRNA expression by qRT–PCR normalised to GAPDH<br>(g) immunostaining	unpaired student´s t test or one-way ANOVA+Tukey’s post hoc test					(a) ++ (↓) P = 0.0112<br><br>(b)+ (↑) in hippocampus<br><br>(c)+ (X) no remarkable changes<br><br>(d)+ hippocampus<br>* (↑)<br>** (↑)<br>(d)++ (↑)<br><br>(e)+ hippocampus<br>* (↑)<br>**NS<br>(e)++ (↑)<br><br>(f)+ (↑)<br><br>(h)+ (↓) in hippocampus<br><br>(i)+ NS<br>(j)+ NS<br>(k)+ NS<br>(l)+ (↓)<br>(m)+ (↑)<br><br>(b)* (↓) P<0.0001<br><br>(c)* (X) (mRNA expression) P = 0.1235<br><br>(d)* (↓) P < 0.0001<br>(d)** (↓) P = 0.0010<br>(d)*** (↓) P = 0.0059<br><br>(e)* (↓) P = 0.0001<br>(e)** NS P = 0.1498<br>(e)*** (↓) P < 0.0001	**microglia volume (n=6 images from 3 mice/each group; activation of classical complement pathway (C3, C1q) fluorescence intensity (n=6 images from 3 mice/each group; expression of complement proteins in the classical complement pathway (C3,C1q,C3aR) protein expression (n=6 images from 3 mice/each group, mRNA expression by qRT–PCR (n=5–6 mice/each group; CD68+ lysosome volume in Iba1+ microglia volume (n=6) images from 3 mice/each group; fractalkine CX3CL1 mRNA expression levels in the hippocampal samples were measured (n=5–6) mice/each group; individual and colocalized excitatory pre- and postsynaptic markers (VGlut, PSD95) and inhibitory pre- and postsynaptic markers (VGAT, Gepyrin) (n=6 images from 3 mice/each group; dendrite spine density of pyramidal neurons in the hippocampal CA1 regions (n=12 images from 3 mice/each group; engulfed VGlut2+synaptic volume + Iba1+ microglial volume (n=6) images from 3 mice/each group.**<br><br>•DRS (b) 2 (%) = 1.29cm<br>•DRS (c) 1 GAPDH = 2.5cm<br>•DRS (d) (expression): (d1) protein 0.5 Tubulin = 1.03cm; (d2) mRNA 1 GAPDH = 1.26cm.<br>•DRS (d3) (activation): fluorescence intensity ratio 0.5 (FIR) = 1.12cm<br>•DRS (e) (expression): (e1) protein 0.5 Tubulin = 0.78cm; (e2) mRNA 0.5 GAPDH = 1.02cm.
			Dopamine (a)		(a) + by HPLC<br>*SNpc: t = 3.005, df = 6<br>** striatum: t = 2.884, df = 6<br>(a)++ by ELISA (striatum)<br>t = 3.614, df = 6				
			CD68+IBA-1 (b)	DRS 1.11cm = 1.72%	(b)+ NR DRS 4.27cm = 6.62%	DRS 1.57cm = 2.43%	DRS 0.77cm = 1.19%		
			fractalkine/ CX3CL1 (c)	DRS 2.5cm = 1 GAPDH	(c)+ (X)<br>NR DRS 2.93cm = 1.17 GAPDH	DRS 2.8cm = 1.12 GAPDH	DRS 2.8cm = 1.12 GAPDH		
			C3 (d)	(d1) DRS 2.04cm = 0.99 Tubulin<br><br>(d2) DRS 1.26cm = 1 GAPDH	(d)+ (expression) NR<br>* (d1) DRS 3.45cm = 1.67 Tubulin<br>** (d2) DRS 2.6cm = 2.06 GAPDH	(d1) DRS 2.41cm = 1.17 Tubulin<br><br>(d2) DRS 1.65cm = 1.31 GAPDH	(d1) DRS 1.98cm = 0.96 Tubulin<br><br>(d2) DRS 1.59cm = 1.26 GAPDH		
				(d3) DRS 2.25cm = 1.00 FIR	(d)++ (activation) hippocampal CA1 region<br>(d3) DRS 3.47cm = 1.55 FIR	(d3) DRS 1.96cm = 0.87 FIR	(d3) DRS 2.59cm = 1.16 FIR		
			C1q (e)	(e1) DRS 1.56cm = 1 Tubulin<br><br>(e2) DRS 2.02cm = 0.99 GAPDH	(e)+ (expression) NR<br>* (e1) DRS 3cm = 1.92 Tubulin<br>** (e2) DRS 2.31cm = 1.13 GAPDH	(e1) DRS 1.66cm = 1.06 Tubulin<br><br>(e2) DRS 2.31cm = 1.13 GAPDH	(e1) DRS 1.65cm = 1.06 Tubulin<br><br>(e2) DRS 1.79cm = 0.88 GAPDH		
				(e3) DRS 1.16cm = 1 FIR	(e)++ (activation) hippocampal CA1 region<br>(e3) DRS 3.08cm = 2.65 FIR	(e3) DRS 1.5cm = 1.29 FIR	(e3) DRS 1.66cm = 1.43 FIR		
			C3aR (f)	DRS 1.55cm = 1.09 GAPDH	(f)+ NR DRS 2.47cm = 1.734 GAPDH	DRS 1.56cm = 1.10 GAPDH	DRS 1.55cm = 1.09 GAPDH		

		synaptic/puncta density (fold)<br>(h) (fold) Scale bar = 5 µm<br>(i) (fold) Scale bar = 5 µm<br>(j) (fold) immunostaining synaptic density<br>(k) (fold) immunostaining synaptic density<br>(l) spine numbers on apical dendrites-Golgi-stained Scale bar = 5 µm<br>(m) Immunostaining Scale bar = 10 µm – occupancy (%)	PSD95 (g)		DRS 1.68cm = 1 fold	NR DRS 1.03cm = 0.61 fold	DRS 1.68cm = 1 fold		DRS 1.83cm = 1.09 fold	(f)* (↓) P = 0.0204<br>(g)* P=0.0014<br>(h)* (↑) P=0.0004<br>(i)* NS P=0.3384<br>(j)* NS P=0.1440<br>(k)* NS P=0.8476<br>(l)* (↑) P<0.0001<br>(m)* (↓) P<0.0001	•DRS (e3) (activation): fluorescence intensity ratio 1 (FIR) = 1.16cm<br>•DRS (f) 1 GAPDH = 1.42cm.<br>•DRS (g) 1-fold = 1.68cm<br>•DRS (h) 1-fold = 1.68cm<br>•DRS (i) 1-fold = 1.07cm<br>•DRS (j) 1-fold = 1.07cm<br>•DRS (k) 1-fold = 1.07cm<br>•DRS (l) 10 spine density (Sds)/10µm = 3.1cm.<br>•DRS (m) 2 occupancy (%) = 1.1cm<br><br>•Other assessments: Behavioural test (body weight, Rotarod test, Pole climbing test, OFT, FST, Tail suspension test, Sucrose preference test), In vitro cell culture-CCK-8 assay (mouse BV2 microglial cell line supplemented with reserpine in the presence/absence of BoNT for 24h).<br>•BoNT significantly helped depressive-like behaviours.
			VGlut & PSD95 (h)		DRS 1.68cm = 1 fold	(h)+ NR DRS 1.05cm = 0.62fold	DRS 1.83cm = 1.09 fold		DRS 1.91cm = 1.14 fold		
			VGAT (i)		DRS 1.07cm = 1 fold	(i)+ NR DRS 1.27cm =1.19 fold	DRS 1.23cm = 1.15 fold		DRS 0.9cm = 0.84 fold		
			VGAT+ Gephyrin (j)		DRS 1.07cm = 1 fold	(j)+ NR DRS 1.64cm =1.53 fold	DRS 1.2cm = 1.12 fold		DRS 0.74cm = 0.69 fold		
			Gephyrin (k)		DRS 1.07cm = 1 fold	(k)+ NR DRS 1.25cm = 1.17fold	DRS 1.07cm = 1 fold		DRS 1.28cm = 1.20 fold		
			dendritic spines of CA1 pyramidal neurons (l)		DRS 3.2cm = 10.32 Sds	(l)+ NR DRS 1.84cm = 5.93 Sds	DRS 2.86cm = 9.22 Sds		DRS 3.13cm = 10.10 Sds		
			engulfed VGlut2+ synaptic volume + Iba1+ microglial volume (m)		DRS 1.1cm = 2%	(m)+ NR DRS 3.1cm = 5.64%	DRS 1 cm = 1.82 %		DRS 1.2cm = 2.18%		
			(?) follow-up	(a)			NR				
				(b)			Hippocampus				
							. (b)* F (3, 20) = 27.82				
				(c)			Hyppocampal				
							(c)* F (3, 16) = 2.236				
				(d)			Hyppocampal				
							(d)* protein	(d)** mRNA			
							F (3, 8) = 34.09	F (3, 20) = 8.045			
							(d)*** hippocampal CA1 region				
							F (3, 20) = 5.604				
				(e)			Hyppocampal				
							(e)* protein	(e)** mRNA			
							F (3, 8) = 29.37	F (3, 20) = 1.978			
							(e) *** hippocampal CA1 region				
							F (3, 20) = 22.60				
				(f)			(f)* F (3, 16) = 4.338				
				(g)			hippocampal CA1				
							(g)* F (3, 20) = 7.603				
				(h)			hippocampal CA1				
							(h)* F (3, 20) = 9.593				
				(i)			hippocampal CA1				

							(i)* F (3, 20) = 1.191				
				(j)			hippocampal CA1				
							(j)* F (3, 20) = 2.016				
				(k)			(k)* F (3, 20) = 0.2680				
				(l)			hippocampal CA1 regions				
							F (3, 44) = 27.26				
				(m)			hippocampal CA1 regions				
							F (3, 20) = 25.95				
**BIOMARKER:** Nav isoform: 1.3, 1.6, 1.7, 1.8, ATF3											
**Yang, 2016 [31]**	**Trigeminal neuropathic pain** by malpositioned dental implants following extraction of 2nd molar- inducing injury to the inferior alveolar nerve.<br><br>POD-3, saline or BoNT (3U/kg) s.c. injection into the most sensitive area of facial region	**nerve-injured trigeminal ganglion – TG (mandibular (V3) division and boundary area)** expression - GAPDH<br><br>(e) positive cells counted in 5 sections from each rat	One-way ANOVA + Holm-Sidak post hoc analysis. Mean ± SEM at each time point tested		**(n=?) Sprague-Dawley rat, age unknown, Male (220 and 240g)**					(a)+ NS<br>(b)+ (↑) P < 0.05<br>(c)+ (↑) P < 0.05<br>(d)+ (↑) P < 0.05<br>(e)+ (↑) P < 0.05<br><br>(a)* NS<br>(b)* NS<br>(c)* (↓) P < 0.05, POD-9<br>(d)* NS<br>(e)* NS	Other assessments: Mechanical allodynia, air-puff threshold, aggressiveness, and biting.<br>**•for immune-histochemistry, analysis on (n=5 / group)**<br>▪DRS (a) 0.4 GAPDH = 2.52cm<br>▪DRS (b) 0.4 GAPDH = 1.8cm<br>▪DRS (c1) 0.4 GAPDH = 1.21cm<br>▪DRS (c2) 8 fluorescence intensity (F.i)/immunoreactivity =2.32cm<br>▪DRS (d) 0.4 GAPDH = 1.35cm<br>▪DRS (e) 40 number of positive neurons = 1.78cm
					Naïve/Sham (extraction without implant placement)	TN+ vehicle (saline)	TN + BoNT		TN + vehicle (saline)		
			Nav 1.3 (a)		DRS 5.04cm = 0.8GAPDH	(a)+ NR	DRS 4.58cm=0.73GAPDH		DRS 4.65cm= 0.74GAPDH		
			Nav 1.6 (b)		DRS 2.55cm = 0.57GAPDH	(b)+ NR	DRS 3.96cm= 0.88GAPDH		DRS 4.7cm= 1.04 GAPDH		
			Nav 1.7 (c)		DRS 2.42cm = 0.8 GAPDH	(c)+ NR	DRS 1.93cm= 0.64 GAPDH		DRS 5.08cm = 1.68 GAPDH		
					DRS 2.32cm = 8 F.i.		DRS 2.87cm= 9.90 F.i.		DRS 4.5cm = 1.33 F.i.		
			Nav 1.8 (d)		NR	(d)+ NR	DRS 4.34cm= 97.5 GAPDH		DRS 4.75cm= 106.8 GAPDH		
			ATF3 (e)			(e)+ NR					
			6-days after BoNT	(a)			(a)* NR				
				(b)			(b)* NR				
				(c)			(c)* F2,12 = 9.176				
				(d)			(d)* NR				
				(e)			(e)* NR				
**BIOMARKER:** TRPV4, TRPM3											
**Zhang, 2019 [32]**	**Trigeminal neuropathy** by IoNC<br><br>S.c. injection into the whisker pad tissue on the operation side, 14 days after IoNC	**Trigeminal spinal subnucleus caudalis** protein expression, optical densities – (1) OD (folds), (2) mean OD/area	mean±SEM. Due to heterogeneity of variance, Kruskal-Wallis+ Wilcoxon rank sum tests.		**(n=?) Sprague–Dawley rats, age unknown, Male (200–250g)**					(a)+ (↑) P<0.05<br>(b)+ (↑) P<0.05<br><br>(a)* (↓) P<0.05<br>(b)* (↓) P<0.05<br>«3U vs 10U: NS (P>0.05)	Other assessments: pain threshold (Von Frey hairs)<br>• Compared with IoNC, the pain thresholds of 3U and 10U were (↑) 4 days after BoNT (P<0.05)<br>▪DRS (a1) 1 ODf = 1.45cm<br>▪DRS (a2) 0.1 ODa = 1.9cm<br>▪DRS (b1) 1 ODf = 1.35cm<br>▪DRS (b2) 0.1 ODa = 1.95cm
					Sham + saline (identical operation, but the nerve was not ligated)	IoNC + saline (vs sham)	IoNC + BoNT (vs IoNC + saline)		IoNC+ saline		
							3U	10U			
			TRPV4 (a)		(a1) DRS 1.45cm= 1ODf	(a)+ NR	(a1) DRS 0.95cm = 0.65ODf	(a1) DRS 0.88cm = 0.61ODf	(a1) DRS 2.3cm= 1.59ODf		

					(a2) DRS 3.14cm = 0.16ODa				(a2) DRS 2.25cm =0.12ODa	(a2) DRS 2cm= 0.10ODa	(a2) DRS 3.9cm= 0.20ODa		
			TRPM3 (b)		(b1) DRS 1.35cm= 1ODf	(b)+ NR			(b1) DRS 0.4cm = 0.30ODf	(b1) DRS 0.4cm = 0.30ODf	(b1) DRS 3.1cm= 2.30ODf		
					(b2) DRS 3.76cm= 0.19ODa				(b2)DRS 1.94cm= 0.10ODa	(b2)DRS 1.85cm= 0.09ODa	(b2) DRS 4.75cm= 0.24ODa		
			7-days after BoNT	(a)					(a)* NR	(a)* NR«			
				(b)					(b)* NR	(b)* NR«			
**BIOMARKER:** TRPV1, TRPV2, TRPM8													
**Wu, 2016 [23]**	**Trigeminal neuropathy** by IoNC<br><br>BoNT/saline subcutaneously into the whisker pad tissue (ip.l. to the nerve injury) 14 days after the IoNC<br><br>Colchicine or normal saline (2 µl) was injected into the TG (ip.l. to the nerve injury) of anesthetized rat, 12h before BoNT/saline (IoNC + colchicine + saline/ BoNT)	**Brainstem Vc region (caudal subnucleus of the spinal trigeminal nucleus)** (protein expression)	mean ± SD. One-way ANOVA and the New-man-Keuls test		**(n=?) Sprague–Dawley rats, age unknown, Male (220–300g)**							(a)+ (↑) (P <0.05) (day-14; increased until day-28)<br>(b)+ (↑) (P <0.05) (day-14; increased until day-28)<br>(c)+ (↑) (P <0.05 (day-7: max; day-14 remained increased until day-28)<br><br>(a)* (↓) (P<0.05) (dose-related manner)<br>(b)* no change (P>0.05)<br>(b)** (↓) (P<0.05)<br>(c)* no change (P>0.05)	•Other assessments: Antinociceptive effect/pain threshold/ allodynia tested by Von Frey filaments, motor coordination ability by Rota-rod test. Colchicine effects. Quantitative analysis of TRPA1 at various times after ION-CCI.<br>▪**Analysis (n=6)/group**<br>•DRS (IoNC vs control saline): 1OD = 1.16cm<br>•DRS (BoNT vs control): 1OD = 1.15cm<br>•Peripheral application of BoNT (3, 10 U/kg) (↑) pain threshold of IoNC.<br>•Antinociceptive effects reached a max. level at 8 days and remained (↑) until 20 days. The 10 U/kg had better antinociceptive effects than the 3 U/kg but NS (P>0.05)<br>•Axonal transport blocker colchicine injection into TG resulted in failure to ↑ the level of clSNAP-25 and in disappearance of the antinociceptive effects of BoNT (10 U/kg) P > 0.05
					(n=6) Sham + saline + saline (IoNC not ligated)	(n=6) IoNC + saline (vs control)			IoNC + saline + Peripheral BoNT (vs control)		(n=6) IoNC + saline + saline		
						7D	14 D	28 D	(n=6) 3U/Kg	(n=6) 10 U/kg			
			TRPV1 (a)		DRS 1.6cm = 1.38OD	(a)+ NR DRS							
						1.91 cm = 1.63 OD	4.65 cm = 4 OD	4.42 cm = 3.81 OD					
			TRPV2 (b)			(b)+ NR DRS							
						5cm = 4.31 OD	5.36 cm = 4.62 OD	6.64 cm = 5.72 OD					
			TRPM8 (c)			(c)+ NR DRS							
						3.4 cm = 2.93 OD	4.11 cm = 3.54 OD	4.23 cm = 3.65 OD					
			7-days after BoNT	(a)	DRS 1.5cm = 1.30OD				(a)* NR DRS 4.25cm =3.69OD	(a)* NR DRS 2.74cm = 2.38OD	DRS 5.4cm = 4.69OD		
				(b)					(b)* NS NR DRS 3.06cm = 2.66OD	(b)** NR DRS 2.04cm = 1.77OD	DRS 3.16cm = 2.75OD		

				(c)			(c)* NR DRS 5.26cm = 4.57OD	(c)* NR DRS 5.4cm = 4.69OD	DRS 5.56cm = 4.83OD		
**BIOMARKER:** SOD, Catalase, GSH, GPx											
**Yesudhas, 2021 [34]**	**Anxiety and ageing**<br><br>(1U/Kg body weight) single and mild dose intramuscular injection in the thigh:<br>G1. BoNT (n=6)<br>G2. Control-Sterile saline (n=6)	**Hippocampus (brain) tissues – total protein isolates**<br>(U/mg protein)	mean ± SD. One-way ANOVA + Tukey's post hoc. Student t-test.		**(n=12) ageing model-BALB/c mice, 7-8 months-old, Male (unknown weight)**					* (↑) P < 0.05	Other assessments:<br>behavioural tests - OFT (seconds), elevated plus maze (seconds), and light-dark box test (seconds).<br>▪BoNT exhibited better performance in all behavioural tests.<br>▪DRS (a) 0.2 activity U/mg protein extracts = 1.1cm<br>▪DRS (b) 20 activity U/mg protein extracts = 2.8cm<br>▪DRS (c) 0.1 activity U/mg protein extracts = 1.1cm<br>▪DRS (d) 15 activity U/mg protein extracts = 3.35cm
					Ageing model (control)	(n=6) control + saline	(n=6) BoNT (vs control + saline)				
			SOD (a)			DRS 3.57cm=0.65U/mg	DRS 4.34cm=0.79U/mg				
			Catalase (b)			DRS 3.87cm= 27.64 U/mg	DRS 4.88cm= 34.86U/mg				
			GSH (c)			DRS 3.3cm= 0.3 U/mg	DRS 4.3cm= 0.39 U/mg				
			GPx (d)			DRS 4.15cm= 18.58 U/mg	DRS 4.8cm= 21.49 U/mg				
			30-days	(a)		0.63 ± 0.08	0.76 ± 0.03*				
				(b)		27.5 ± 3.3	34.5 ± 2.4*				
				(c)		0.29 ± 0.008	0.38 ± 0.05*				
				(d)		18.4 ± 2.3	21.2 ± 1.3*				

LEGEND:

*** Effect not reported.**

CIS, chronic inflammatory state; **BoNT,** botulinum toxin; **(↑)**, statistically significantly higher/increased; **(↓),** statistically significantly lower/decreased; **SEM,** standard error of the mean; **NS**, no statistically significant difference; **NR**, not reported; **DRS**, digital ruler software; **ip.l.**, ipsilateral; **c.l.**, contralateral; **s.c.**, subcutaneously; **i.c.**, intracisternally**; i.a.**, intraarticular; **i.g**. intraganglionic; **f.c,** fold change; **POD**, post operative day; **Tx**, treatment; **O.D,** optical density**; (mm)**, millimetres; **SD**, standard deviation; **ANOVA**; analysis of variance; **(r),** decay time constant; **CBCT**, Cone Beam computer Tomography; **ELISA**, enzyme linked immunosorbent assay; **qRT–PCR**; Real-Time Quantitative Reverse Transcription PCR**; RGS**, facial grimacing related to pain; **FST**, forced swimming test; **SEI**, scar elevation index; **TEWL,** transepidermal water loss; **HS**, hypertrophic scar; **PRP**, platelet rich plasma; **TNR**; trigeminal nerve root; **CCI**, chronic constriction injury; **NeuN**, neuronal nuclei; **WIM**, whisker intrinsic musculature; **OFT**, open field test; **MMP-13**, matrix metallopeptidase; **TG**, trigeminal ganglia; ; **TNC**, trigeminal nucleus caudalis; **TMD**, temporomandibular disorder; **TMJ**, temporomandibular joint; **OA**, osteoarthritis; **TN**, trigeminal neuralgia; **PIH**, persistent immunogenic hypersensitivity; **TNF-α**, tumor necrotic factor-α; **IL**, interleukin; **ION-CCI/IoNC**, infraorbital nerve constriction; **SNpc**, substantia nigra pars compacta; **vlPAG**, ventrolateral periaqueductal gray; **mBSA**, methylated bovine serum albumin; **CFA**, Complete Freund's Adjuvant; **PBS**, phosphate-buffered saline; **NTG**, nitroglycerin; **SP**, substance P; **CGRP**, calcitonin gene related peptide; **BDNF**, brain derived neurotrophic factor; **(cl)SNAP-25**, (cleaved) synaptosomal-associated protein-25; **ChAT**, choline acetyltransferase; **PSD95**, postsynaptic density-95; **NMDAR**, N-methyl-D-aspartate receptor; **5-HT**, 5-hydroxytryptamine; **SRS**; spatial restraint stress; **Vc**, caudal subnucleus of the spinal trigeminal nucleus**; p-ERK**, phosphorylated extracellular signal-regulated kinase; **p-CREB**, cAMP response element binding protein; **Iba-1**; ionized calcium-binding adaptor molecule 1; **TLRs** - toll-like receptors; **c-Fos**, neuron activation marker; **GFPA**, glial fibrillary acidic protein; **DNI**, dural neurogenic inflammation; **IgE**, immunoglobulin E; **CX3CR1,** CX3 chemokine receptor 1; **IB4**, isolectin B4-binding; **COL-I**, collagen 1-related proteins; **COL-II**, collagen 2-related proteins; **α-SMA**, α- smooth muscle actin; **HIF-1α**, hypoxia-inducible factor; **TH**, tyrosine hydroxylase, dopaminergic neuronal marker; **VGlut2,** vesicular glutamate transporter 2; **VGAT,** vesicular GABA transporter; **SOD**, superoxide dismutase; **GSH,** glutathione; **GPx,** glutathione peroxidase; **DRG**; dorsal root ganglia; **ATF3**, activating transcription factor 3; **TRPV4**, protein expression of transient receptor potential vanilloid type 4; **TRPM**, transient receptor potential melastatin; **TNCB**, 2-Chloro-1,3,5-trinitrobenzene; **TRPV,** transient receptor potential vanilloid type ; **TRPA1**, transient receptor potential ankyrin 1; **Vc**, caudal subnucleus of the spinal trigeminal nucleus; **AEW**, acetone-diethylether-water; **TGF-β1**, transforming growth factor beta; **vAChT**, acetylcholine vesicular transporter; **SerT**, serotonin transporter; **CTB-488**, monosynaptic retrograde tracer; **PRV-EGFP**, retrograde polytranssynaptic pseudorabies virus (PRV) tracer, EGFP-conjugated; **wFMNs**, whisker-innervating facial motoneurons; **CaMKII**, excitatory Ca^{2+}/calmodulin-dependent protein kinase type II; **GAD67**, inhibitory glutamate decarboxylase 1; **vlPAG,** ventrolateral periaqueductal grey; **TpH2**, tryptophan hydroxylase 2, serotoninergic neuronal marker; **GAPDH,** Glyceraldehyde 3-phosphate dehydrogenase
