# Supplementary material for: Botulinum Toxin Effects on Biochemical Biomarkers Related to Inflammation-Associated Head and Neck Chronic Conditions: A Systematic Review of Preclinical Research
Source: Toxins (Basel). 2025 Jul 29;17(8):377. doi: 10.3390/toxins17080377 (PMC12390450; doi:10.3390/toxins17080377)
Supplement: Supplementary file 1 [file toxins-17-00377-s001.zip › SR2. file S8. GRADE Justification.pdf]

File S8: Biomarkers in Preclinical Research on Botulinum Toxin effects on Chronic Inflammatory State. GRADE consistency assessment

Biomarker (CIS)	Biological Sampling	Author Year	Unit measure	BoNT Key effect Follow-up after BoNT (Outcome) Summary	Justification / Criteria (*): 1. Risk of bias (**) 2. Imprecision 3. Inconsistency 4. Indirectness 5. Publication bias — Downgrade (RCT) — Upgrade (nRT) — 1. Large effect 2. Dose-response gradient 3. Opposing plausible residual confounding and bias	Overall GRADE
**6 STUDIES**						
					**TNC**	
**IL-1β**	**TN**	**Chen, 2021 [14]**	(mRNA expression)	1 RCT (n=48)<br>(T1) 5 days (↓)	Start: High quality (RCT)<br>Downgraded: Moderate quality - (1) High RoB D8; Unclear RoB D3 and D7.<br>Downgraded: Low quality – (4) Population - several inferior animal and disease models were used in the included studies. There are no current animal models of TN that adequately replicate the clinical disorder. Most used IoNC. Compression of TNR has been linked to the main theory for the pathophysiology of TN.	LOW ⊕⊕○○
	**PHI & TMJ arthritis**	**Muñoz-Lora, 2020 [16]**	(pg/mL tissue)	1 RCT (n=40)<br>(T1) 24h (↓)<br>(T2) 7 days (NS)<br>(T3) 14 days (NS)	Start: High quality (RCT)<br>Downgraded: Moderate quality - (1) High RoB D6; Unclear RoB D3 and D5.<br>Downgraded: Low quality – (4) Population - several inferior animal and disease models were used in the included studies. Model is useful to assess immunomodulatory mechanisms. However, there are other mechanisms involved in the pathogenesis of the model that may also be related to BoNT activity.	
					**TMJ tissues**	
	**TMJ OA**	**Makawi, 2022 [13]**	(pg/ml tissue; mRNA; protein expression)	1RCT (n=42)<br>(T1) 2weeks (↓)<br>(T2) 4 weeks (↓)	Start: High quality (RCT)<br>Downgraded: Moderate quality - (1) High RoB D6, Unclear RoB D3, D5 and D7.<br>Downgraded: Low quality – (4) Population - several inferior animal and disease models were used in the included studies. There is still not a clear "gold standard" for choosing the best animal model for OA.	LOW ⊕⊕○○
					**Peri-articular tissues from TMJ and TG**	
	**PIH & TMJ arthritis**	**Muñoz-Lora, 2017 [15]**	(pg/mL)	1 ? (n=?)<br>(T1) 24h (↓)<br>(T2) 14 days (↓)	Start: Very Low quality (nRCT)	VERY LOW ⊕○○○
					**TG**	
	**TN**	**Cho, 2022 [17]**	(pg/ml tissue)	1 RCT (n=236)<br>(T1) 2 days (↓)	Start: High quality (RCT)<br>Downgraded: Moderate quality - (1) Unclear RoB D3 and D7.<br>Downgraded: Low quality – (4) Population - several inferior animal and disease models were used in the included studies. There are no current animal models of TN that adequately replicate the clinical disorder. Most used IoNC. Compression of TNR has been linked to the main theory for the pathophysiology of TN.	LOW ⊕⊕○○
					**Brain - SNpc & hippocampus**	
	**Depression - PD**	**Li, 2023 [18]**	(mRNA; protein expression)	1 ? (n=?)<br>(T1) ? (↓)	Start: Very Low quality (nRCT)	VERY LOW ⊕○○○

5 STUDIES						
	**TNC**					
**TNF-α**	**TN**	**Chen, 2021 [14]**	(mRNA; protein expression)	1 RCT (n=48)<br>(T1) 5 days (↓)	Start: High quality (RCT)<br>Downgraded: Moderate quality - (1) High RoB D8; Unclear RoB D3 and D7.<br>Downgraded: Low quality – (4) Population - several inferior animal and disease models were used in the included studies. There are no current animal models of TN that adequately replicate the clinical disorder. Most used IoNC. Compression of TNR has been linked to the main theory for the pathophysiology of TN.	LOW ⊕⊕○○
	**PHI & TMJ arthritis**	**Muñoz-Lora, 2020 [16]**	(pg/mL tissue)	1 RCT (n=40)<br>(T1) 24h (NS)<br>(T2) 7 days (NS)<br>(T3) 14 days (↓)	Start: High quality (RCT)<br>Downgraded: Moderate quality - (1) High RoB D6; Unclear RoB D3 and D5.<br>Downgraded: Low quality – (4) Population - several inferior animal and disease models were used in the included studies. Model is useful to assess immunomodulatory mechanisms. However, there are other mechanisms involved in the pathogenesis of the model that may also be related to BoNT activity.	LOW ⊕⊕○○
	**Peri-articular tissues from TMJ and TG**					
	**PIH & TMJ arthritis**	**Muñoz-Lora, 2017 [15]**	(pg/mL)	1 ? (n=?)<br>(T1) 24h (NS)<br>(T2) 14 days (NS)	Start: Very Low quality (nRCT)	VERY LOW ⊕○○○
	**TG**					
	**TN**	**Cho, 2022 [17]**	(pg/ml tissue)	1 RCT (n=236)<br>(T1) 2 days (↓)	Start: High quality (RCT)<br>Downgraded: Moderate quality - (1) Unclear RoB D3 and D7.<br>Downgraded: Low quality – (4) Population - several inferior animal and disease models were used in the included studies. There are no current animal models of TN that adequately replicate the clinical disorder. Most used IoNC. Compression of TNR has been linked to the main theory for the pathophysiology of TN.	LOW ⊕⊕○○
	**Brain - SNpc & hippocampus**					
	**Depression - PD**	**Li, 2023 [18]**	mRNA; protein expression	1 ? (n=?)<br>(T1) ? (↓)	Start: Very Low quality (nRCT)	VERY LOW ⊕○○○
	**Hippocampus, hypothalamus, prefrontal cortex, amígdala (Brain)**					
**SNAP25**	**Depression**	**Li, 2019 [21]**	protein expression	1 nRT (n=?)<br>(T1) 16-,18-,22-, 29-days (NS)	Start: Very Low quality (nRCT)	VERY LOW ⊕○○○
	**TNC**					
	**PIH & TMJ arthritis**	**Muñoz-Lora, 2022 [22]**	(cSNAP-25 staining/ positive fibres)	1 RCT (n=40)<br>(T1) 14 days (+)<br>OnaBoNT (7U), ipl.	Start: High quality (RCT)<br>Downgraded: Moderate quality - (1) High RoB D10, Unclear RoB D3, D5 and D7.<br>Downgraded: Low quality – (4) Population - different inferior animal and disease models were used in the included studies<br>(5) Funded by pharmaceutical industry and not disclosing the conflicts of interest.	LOW ⊕⊕○○
	**Cranial dura**					
	**TMDs trigeminal inflammatory pain**	**Lacković, 2016 [20]**	(presence clSNAP25 - fibers	1 RCT (n=105)<br>(T1) 4-days (+) ipl.	Start: High quality (RCT)<br>Downgraded: Moderate quality - (1) High RoB D8; Unclear RoB D3 and D7.<br>Downgraded: Low quality – (4) Population - different inferior animal and disease models were used in the	LOW ⊕⊕○○

			containing SNAP-25 co-expressed for CGRP. Scale bars = 100 µm)		included studies.	
				**Brain – hindbrain sections**		
	**MDD**	**Ni, 2023 [24]**	(positive signal) (% positive signal area)	1 RCT (n=?) (T1) 10-days (+) IFN ipl. (T2) 4-weeks (+) IFN ipl. (T3) 7-weeks (+) IFN ipl.	Start: High quality (RCT)<br>Downgraded: Moderate quality - (1) Unclear RoB D3 and D7.<br>Downgraded: Low quality – (4) Population - different inferior animal and disease models were used in the included studies. Results obtained from rodents are not generalizable to human feelings and expression.	LOW ⊕⊕◯◯
				**Brainstem Vc region (caudal subnucleus of the spinal trigeminal nucleus)**		
	**TN**	**Wu, 2016 [23]**	(by β-actin)	1 ? (n=?) (T1) 7 days (↑)	Start: Very Low quality (nRCT)	VERY LOW ⊕◯◯◯
**4 STUDIES**						
				**TNC**		
**c-Fos**	**TN**	**Chen, 2021 [14]**	(mRNA; protein expression)	1 RCT (n=48) (T1) 5 days (↓)	Start: High quality (RCT)<br>Downgraded: Moderate quality - (1) High RoB D8; Unclear RoB D3 and D7.<br>Downgraded: Low quality – (4) Population - different inferior animal and disease models were used in the included studies. There are no current animal models of TN that adequately replicate the clinical disorder. Most used IoNC. Compression of TNR has been linked to the main theory for the pathophysiology of TN.	LOW ⊕⊕◯◯
	**PHI & TMJ arthritis**	**Muñoz-Lora, 2022 [22]**	levels of c-Fos-positive nuclei	1 RCT (n=40) (T1) 14 days (↓) – ipl & cl. OnaBoNT (7U,14U)	Start: High quality (RCT)<br>Downgraded: Moderate quality - (1) High RoB D10, Unclear RoB D3, D5 and D7.<br>Downgraded: Low quality – (4) Population – different inferior animal and disease models were used in the included studies<br>(5) Funded by pharmaceutical industry and not disclosing the conflicts of interest	LOW ⊕⊕◯◯
				**Brain – hindbrain sections**		
	**MDD**	**Ni, 2023 [24]**	(positive signal) (% positive signal area)	1 RCT (n=?) (T1) 24h after day-23 to -27 (↓)	Start: High quality (RCT)<br>Downgraded: Moderate quality - (1) Unclear RoB D3 and D7.<br>Downgraded: Low quality – (4) Population - different inferior animal and disease models were used in the included studies. Results obtained from rodents are not generalizable to human feelings and expression.	LOW ⊕⊕◯◯
				**Medullary dorsal horn**		
	**TN**	**Kim, 2015 [26]**	(expression - number of neurons - Scale bar,	1 ? (n=?) (T1) ? (↓)	Start: Very Low quality (nRCT)	VERY LOW ⊕◯◯◯

			100 µm)			
	Jugular plasma and medulla oblongata - containing caudal trigeminal nucleus					
**CGRP**	**Migraine**	**Shao, 2013 [19]**	(pg/mL)	1 ? (n=32)<br>(T1) 24h (↓)	Start: Very Low quality (nRCT)	VERY LOW ⊕○○○
	TNC					
	**PIH & TMJ arthritis**	**Muñoz-Lora, 2022 [22]**	(pg/mL)<br>area (μm^2)	1 RCT (n=40)<br>(T1) 14 days – BoNT (7U/14U) (NS)	Start: High quality (RCT)<br>Downgraded: Moderate quality - (1) High RoB D10, Unclear RoB D3, D5 and D7.<br>Downgraded: Low quality – (4) Population - different inferior animal and disease models were used in the included studies<br>(5) Funded by pharmaceutical industry and not disclosing the conflicts of interest	LOW ⊕⊕○○
	Peri-articular tissues (TMJ) & TG					
	**PIH & TMJ arthritis**	**Muñoz-Lora, 2017 [15]**	(ng/mL)	1 ? (n=?) ?<br>(T1) 24h (↓)<br>(T2) 14 days (↓)	Start: Very Low quality (nRCT)	VERY LOW ⊕○○○
	Dura mater, TNC, TG, CSF					
	**Trigeminal pain - TMDs (inflammatory pain)**	**Lacković, 2016 [20]**	Concentration (fmol mg^{-1} or fmol mL^{-1})	1 RCT (n=105)<br>(T1) 4-days (↓)<br>cranial dura	Start: High quality (RCT)<br>Downgraded: Moderate quality - (1) High RoB D8; Unclear RoB D3 and D7.<br>Downgraded: Low quality – (4) Population – different inferior animal and disease models were used in the included studies	LOW ⊕⊕○○
**2 STUDIES**						
	Jugular plasma and medulla oblongata - containing caudal trigeminal nucleus					
**SP**	**Migraine**	**Shao, 2013 [19]**	(pg/mL)	1 ? (n=32)<br>(T1) 24h (↓)	Start: Very Low quality (nRCT)	VERY LOW ⊕○○○
	Peri-articular tissues (TMJ) & TG					
	**PIH & TMJ arthritis**	**Muñoz-Lora, 2017 [15]**	(ng/mL)	1 ? (n=?) ?<br>(T1) 24h (↓)<br>(T2) 14 days (↓)	Start: Very Low quality (nRCT)	VERY LOW ⊕○○○
**IL-6**	TG					
	**TN**	**Cho, 2022 [17]**	pg/mL tissue	1 RCT (n=236)<br>(T1) 2 days (↓)	Start: High quality (RCT)<br>Downgraded: Moderate quality - (1) Unclear RoB D3 and D7.<br>Downgraded: Low quality – (4) Population - several inferior animal and disease models were used in the included studies. There are no current animal models of TN that adequately replicate the clinical disorder. Most used IoNC. Compression of TNR has been linked to the main theory for the pathophysiology of TN.	LOW ⊕⊕○○
	TNC					
	**TN**	**Chen, 2021 [14]**	(mRNA; protein expression)	1 RCT (n=48)<br>(T1) 5 days (↓)	Start: High quality (RCT)<br>Downgraded: Moderate quality - (1) High RoB D8; Unclear RoB D3 and D7.<br>Downgraded: Low quality – (4) Population - several inferior animal and disease models were used in the included studies. There are no current animal models of TN that adequately replicate the clinical disorder.	LOW ⊕⊕○○

					Most used IoNC. Compression of TNR has been linked to the main theory for the pathophysiology of TN.	
	**TNC**					
**CX3CR1**	**PIH & TMJ arthritis**	**Muñoz-Lora, 2020 [16]**	(OD, protein level)	1 RCT (n=40)<br>(T1) 24h (NS)<br>(T2) 7 days (NS)<br>(T3) 14 days (NS)	Start: High quality (RCT)<br>Downgraded: Moderate quality - (1) High RoB D6; Unclear RoB D3 and D5.<br>Downgraded: Low quality – (4) Inferior animal models were used. Disease-model is useful to assess immunomodulatory mechanisms. However, there are other mechanisms involved in the pathogenesis of the model that may also be related to BoNT activity.	LOW ⊕⊕○○
	**Brain - SNpc & hippocampus**					
	**Depression - PD**	**Li, 2023 [18]**	(mRNA expression levels)	1 ? (n=?)<br>(T1) ? (↓)	Start: Very Low quality (nRCT)	VERY LOW ⊕○○○
	**Cranial dura Plasma protein complexes**					
**Dural Protein Extravasation**	**TN**	**Filipović, 2012 [28]**	(ng of Evans blue per mg of dural tissue)	1 nRT (n=200)<br>(T1) 3 days (↓) ipl. & cl.	Start: Very Low quality (nRCT)	VERY LOW ⊕○○○
	**Cranial dura tissue, brainstem (TNC)**					
	**Trigeminal pain - TMDs (inflammatory pain)**	**Lacković, 2016 [20]**	ng (mg tissue)$^{-1}$	1 RCT (n=105)<br>(T1) 4-days (↓) ipl.	Start: High quality (RCT)<br>Downgraded: Moderate quality - (1) High RoB D8; Unclear RoB D3 and D7.	MODERATE ⊕⊕⊕○
	**Scar tissue (ear)**					
**Collagen**	**HS**	**Xiong, 2023 [30]**	protein concentration and expression	1 RCT (n=24)<br>(T1) 5 weeks (↓)	Start: High quality (RCT)<br>Downgraded: Moderate quality - (1) Unclear RoB D3 and D5.<br>Downgraded: Low quality – (4) The rabbit ear model cannot completely represent human HS.	LOW ⊕⊕○○
		**Wang, 2020 [25]**		1 RCT (n=18)<br>(T1) 28 days (↓)	Start: High quality (RCT)<br>Downgraded: Moderate quality - (1) High RoB D10, Unclear RoB D3, D5 and D7.<br>Downgraded: Low quality – (4) The rabbit ear model cannot completely represent human HS.	LOW ⊕⊕○○
**α-SMAS & Myosin II Proteins**		**Xiong, 2023 [30]**	optical density	1 RCT (n=24)<br>(T1) 5 weeks (↓)	Start: High quality (RCT)<br>Downgraded: Moderate quality - (1) Unclear RoB D3 and D5.<br>Downgraded: Low quality – (4) The rabbit ear model cannot completely represent human HS.	LOW ⊕⊕○○
		**Wang, 2020 [25]**		1 RCT (n=18)<br>(T1) 28 days (↓)	Start: High quality (RCT)<br>Downgraded: Moderate quality - (1) High RoB D10, Unclear RoB D3, D5 and D7.<br>Downgraded: Low quality – (4) The rabbit ear model cannot completely represent human HS.	LOW ⊕⊕○○
**TGF-β1**		**Xiong, 2023 [30]**	protein [] & expression;	1 RCT (n=24)<br>(T1) 5 weeks (↓)	Start: High quality (RCT)<br>Downgraded: Moderate quality - (1) Unclear RoB D3 and D5.<br>Downgraded: Low quality – (4) The rabbit ear model cannot completely represent human HS.	LOW ⊕⊕○○
		**Wang, 2020 [25]**		1 RCT (n=18)<br>(T1) 28 days (↓)	Start: High quality (RCT)<br>Downgraded: Moderate quality - (1) High RoB D10, Unclear RoB D3, D5 and D7.<br>Downgraded: Low quality – (4) Population - several inferior animal and disease models were used in the	LOW ⊕⊕○○

					included studies. The rabbit ear model cannot completely represent human HS.	
**TRPA-1**	**Brainstem Vc region (caudal subnucleus of the spinal trigeminal nucleus)**					
	**TN**	**Wu, 2016 [23]**	protein expression	1 ? (n=?)<br>(T1) 7 days (↓)	Start: Very Low quality (nRCT)	VERY LOW<br>⊕○○○
	**DRG**					
	**TN**	**Cao, 2017 [33]**	protein expression	1 ? (n=525)<br>(T1) 3 days (↓)<br>(T2) 7 days (↓)	Start: Very Low quality (nRCT)	VERY LOW<br>⊕○○○
**1 STUDY**						
	**TNC**					
**IBA-1**	**TN**	**Chen, 2021 [14]**	mRNA; protein expression	1 RCT (n=48)<br>(T1) 5 days (↓)	Start: High quality (RCT)<br>Downgraded: Moderate quality - (1) High RoB D8; Unclear RoB D3 and D7.<br>Downgraded: Low quality – (4) There are no current animal models of TN that adequately replicate the clinical disorder. Most used IoNC. Compression of TNR has been linked to the main theory for the pathophysiology of TN.	LOW<br>⊕⊕○○
	**Brain- SNpc & hippocampus**					
**fractalkine/ CX3CL1**	**Depression - PD**	**Li, 2023 [18]**	mRNA expression	1 ? (n=?)<br>(T1) ? (X)	Start: Very Low quality (nRCT)	VERY LOW<br>⊕○○○
**VGAT**			Scale bar = 5 µm	1 ? (n=?)<br>(T1) ? (NS)	Start: Very Low quality (nRCT)	VERY LOW<br>⊕○○○
**VGAT/ Gephyrin**			synaptic density	1 ? (n=?)<br>(T1) ? (NS)	Start: Very Low quality (nRCT)	VERY LOW<br>⊕○○○
**Gephyrin**				1 ? (n=?)<br>(T1) ? (NS)	Start: Very Low quality (nRCT)	VERY LOW<br>⊕○○○
**PSD95**				1 ? (n=?)<br>(T1) ? (?)	Start: Very Low quality (nRCT)	VERY LOW<br>⊕○○○
**VGlut2/ PSD95**			Scale bar = 5 µm	1 ? (n=?)<br>(T1) ? (↑)	Start: Very Low quality (nRCT)	VERY LOW<br>⊕○○○
**VGlut2/ IBA-1**			Scale bar = 10 µm - %	1 ? (n=?)<br>(T1) ? (↓)	Start: Very Low quality (nRCT)	VERY LOW<br>⊕○○○
**TH**			protein levels	1 ? (n=?)<br>(T1) ? (√) NS	Start: Very Low quality (nRCT)	VERY LOW<br>⊕○○○

**VGlut2**			mRNA; protein expression	1 ? (n=?)<br>(T1) ? (↑)	Start: Very Low quality (nRCT)	VERY LOW ⊕○○○
**C3**				1 ? (n=?)<br>(T1) ? (↓)	Start: Very Low quality (nRCT)	VERY LOW ⊕○○○
**C1q**				1 ? (n=?)<br>(T1) ? (↓) protein, (NS) mRNA	Start: Very Low quality (nRCT)	VERY LOW ⊕○○○
**C3aR**				1 ? (n=?)<br>(T1) ? (↓)	Start: Very Low quality (nRCT)	VERY LOW ⊕○○○
**CD68/IBA-1**			%	1 ? (n=?)<br>(T1) ? (↓)	Start: Very Low quality (nRCT)	VERY LOW ⊕○○○
				**TMJ tissues**		
**MMP-13**	**TMJ OA**	**Makawi, 2022 [13]**	protein expresson, pg/ml tissue	1RCT (n=42)<br>(T1) 2weeks (↓)<br>(T2) 4 weeks (↓)	Start: High quality (RCT)<br>Downgraded: Moderate quality - (1) High RoB D6, Unclear RoB D3, D5 and D7.<br>Downgraded: Low quality – (4) There is still not a clear "gold standard" for choosing the best animal model for OA.	LOW ⊕⊕○○
				**Hippocampus, hypothalamus, prefrontal córtex, amígdala (Brain)**		
**BDNF**	**Depression**	**Li, 2019 [21]**	mRNA; protein expression	1 nRT (n=?)<br>(B) 1h, 1-,3-,7-days<br>(T1) 16-,18-,22-, 29-days (↑)	Start: Very Low quality (nRCT)	VERY LOW ⊕○○○
**NR1**			by tubulin	1 nRT (n=?)<br>(B) 1h, 1-,3-,7-days<br>(T1) 16-,18-,22-, 29-days (↑)	Start: Very Low quality (nRCT)	VERY LOW ⊕○○○
**NR2A**				1 nRT (n=?)<br>(B) 1h, 1-,3-,7-days<br>(T1)16-,18-,22-,29-days (NR)	Start: Very Low quality (nRCT)	VERY LOW ⊕○○○
**NR2B**				1 nRT (n=?)<br>(B) 1h, 1-,3-,7-days<br>(T1) 16-,18-,22-, 29-days (↑)	Start: Very Low quality (nRCT)	VERY LOW ⊕○○○
**5-HT**			ng/g	1 nRT (n=?)<br>(B) 1day<br>(T1) 16-,18-,22-, 29-	Start: Very Low quality (nRCT)	VERY LOW ⊕○○○

				days (↑)		
**p-ERK**			HPLC analysis, RT-PCR, western blotting (GAPDH)	1 nRT (n=?)<br>(B) 1-, 3-,7-days<br>(T1) 16-,18-,22-, 29-days (↑)	Start: Very Low quality (nRCT)	VERY LOW ⊕○○○
**p-CREB**				1 nRT (n=?)<br>(B) 1-, 3-,7-days<br>(T1) 16-,18-,22-, 29-days (↑)	Start: Very Low quality (nRCT)	VERY LOW ⊕○○○
**TNC**						
**TLR1**	**TN**	**Chen, 2021 [14]**	mRNA; protein expression	1 RCT (n=48)<br>(T1) 5 days (↓) ipl./cl.	Start: High quality (RCT)<br>Downgraded: Moderate quality - (1) High RoB D8; Unclear RoB D3 and D7.<br>Downgraded: Low quality – (4) There are no current animal models of TN that adequately replicate the clinical disorder. Most used IoNC. Compression of TNR has been linked to the main theory for the pathophysiology of TN.	LOW ⊕⊕○○
**TLR2**				1 RCT (n=48),<br>(T1) 5 days (↓) ipl.	Start: High quality (RCT)<br>Downgraded: Moderate quality - (1) High RoB D8; Unclear RoB D3 and D7.<br>Downgraded: Low quality – (4) There are no current animal models of TN that adequately replicate the clinical disorder. Most used IoNC. Compression of TNR has been linked to the main theory for the pathophysiology of TN.	LOW ⊕⊕○○
**TLR4**				1 RCT (n=48)<br>(T1) 5 days (↓) ipl./cl.	Start: High quality (RCT)<br>Downgraded: Moderate quality - (1) High RoB D8; Unclear RoB D3 and D7.<br>Downgraded: Low quality – (4) There are no current animal models of TN that adequately replicate the clinical disorder. Most used IoNC. Compression of TNR has been linked to the main theory for the pathophysiology of TN.	LOW ⊕⊕○○
**TLR5**				1 RCT (n=48)<br>(T1) 5 days (↓) ipl.	Start: High quality (RCT)<br>Downgraded: Moderate quality - (1) High RoB D8; Unclear RoB D3 and D7.<br>Downgraded: Low quality – (4) There are no current animal models of TN that adequately replicate the clinical disorder. Most used IoNC. Compression of TNR has been linked to the main theory for the pathophysiology of TN.	LOW ⊕⊕○○
**TLR8**				1 RCT (n=48)<br>(T1) 5 days (↓) ipl./cl.	Start: High quality (RCT)<br>Downgraded: Moderate quality - (1) High RoB D8; Unclear RoB D3 and D7.<br>Downgraded: Low quality – (4) There are no current animal models of TN that adequately replicate the clinical disorder. Most used IoNC. Compression of TNR has been linked to the main theory for the pathophysiology of TN.	LOW ⊕⊕○○
**TLR11**				1 RCT (n=48)<br>(T1) 5 days (NR)	Start: High quality (RCT)<br>Downgraded: Moderate quality - (1) High RoB D8; Unclear RoB D3 and D7.<br>Downgraded: Low quality – (4) There are no current animal models of TN that adequately replicate the clinical disorder. Most used IoNC. Compression of TNR has been linked to the main theory for the pathophysiology of TN.	LOW ⊕⊕○○

**MyD88**				1 RCT (n=48)<br>(T1) 5 days (↓) ipl.	Start: High quality (RCT)<br>Downgraded: Moderate quality - (1) High RoB D8; Unclear RoB D3 and D7.<br>Downgraded: Low quality – (4) There are no current animal models of TN that adequately replicate the clinical disorder. Most used IoNC. Compression of TNR has been linked to the main theory for the pathophysiology of TN.	LOW<br>⊕⊕◯◯
**CD11b**				1 RCT (n=48)<br>(T1) 5 days (↓) ipl.	Start: High quality (RCT)<br>Downgraded: Moderate quality - (1) High RoB D8; Unclear RoB D3 and D7.<br>Downgraded: Low quality – (4) There are no current animal models of TN that adequately replicate the clinical disorder. Most used IoNC. Compression of TNR has been linked to the main theory for the pathophysiology of TN.	LOW<br>⊕⊕◯◯
**F4/80**				1 RCT (n=48)<br>(T1) 5 days (↓) ipl.	Start: High quality (RCT)<br>Downgraded: Moderate quality - (1) High RoB D8; Unclear RoB D3 and D7.<br>Downgraded: Low quality – (4) There are no current animal models of TN that adequately replicate the clinical disorder. Most used IoNC. Compression of TNR has been linked to the main theory for the pathophysiology of TN.	LOW<br>⊕⊕◯◯
	**Rostral dorsal Skin**					
**IL-4**	**Atopic Dermatitis**	**Han, 2017 [27]**	mRNA; protein expression (ng/mL)	1 RCT (n=48)<br>(T1) 14 days after 1st challenge (↓)	Start: High quality (RCT)<br>Downgraded: Moderate quality - (1) High RoB D10; Unclear RoB D3.<br>Downgraded: Low quality – (4) NC/Nga mice have been associated to low incidence of AD (model was modified)	LOW<br>⊕⊕◯◯
**Mast cell**			Count 5 high power fields	1 RCT (n=48)<br>(T1) 14 days after 1st challenge (↓)	Start: High quality (RCT)<br>Downgraded: Moderate quality - (1) High RoB D10; Unclear RoB D3.<br>Downgraded: Low quality – (4) NC/Nga mice have been associated to low incidence of AD (model was modified)	LOW<br>⊕⊕◯◯
			**Serum - retro orbital plexus**			
**IgE**			ng/mL	1 RCT (n=48)<br>(T1) 14 days after 1st challenge	Start: High quality (RCT)<br>Downgraded: Moderate quality - (1) High RoB D10; Unclear RoB D3.<br>Downgraded: Low quality – (4) NC/Nga mice have been associated to low incidence of AD (model was modified)	LOW<br>⊕⊕◯◯
	**TNC**					
**GFAP**	**PIH & TMJ arthritis**	**Muñoz-Lora, 2022 [22]**	surface area (μm²); mean grey intensity	1 RCT (n=40)<br>(T1) 14 days - BoNT (7U/14U) (↓) ipl	Start: High quality (RCT)<br>Downgraded: Moderate quality - (1) High RoB D10, Unclear RoB D3, D5 and D7.<br>Downgraded: Low quality - (5) Funded by pharmaceutical industry and not disclosing the conflicts of interest	LOW<br>⊕⊕◯◯
	**Peri-articular tissues (TMJ) & TG**					
**Glutamate**	**PIH & TMJ arthritis**	**Muñoz-Lora, 2017 [15]**	(nmol)	1 ? (n=?) ?<br>(T1) 24h (NS)<br>(T2) 14 days (NS)	Start: Very Low quality (nRCT)	VERY LOW<br>⊕◯◯◯
**P2X7**	**TNC**					

	**PIH & TMJ arthritis**	**Muñoz-Lora, 2020 [16]**	OD, protein level	1 RCT (n=40)<br>(T1) 24h (↓)<br>(T2) 7 days (↓)<br>(T3) 14 days (↓)	Start: High quality (RCT)<br>Downgraded: Moderate quality - (1) High RoB D6; Unclear RoB D3 and D5.<br>Downgraded: Low quality – (4) Inferior animal models were used. Disease-model is useful to assess immunomodulatory mechanisms. However, there are other mechanisms involved in the pathogenesis of the model that may also be related to BoNT activity.	LOW ⊕⊕○○
**cathepsin S/**			pg/mL	1 RCT (n=40)<br>(T1) 24h (NS)<br>(T2) 7 days (NS)<br>(T3) 14 days (↓)	Start: High quality (RCT)<br>Downgraded: Moderate quality - (1) High RoB D6; Unclear RoB D3 and D5.<br>Downgraded: Low quality – (4) Inferior animal models were used. Disease-model is useful to assess immunomodulatory mechanisms. However, there are other mechanisms involved in the pathogenesis of the model that may also be related to BoNT activity.	LOW ⊕⊕○○
**fractalkine**				1 RCT (n=40)<br>(T1) 24h (NS)<br>(T2) 7 days (↓)<br>(T3) 14 days (↓)	Start: High quality (RCT)<br>Downgraded: Moderate quality - (1) High RoB D6; Unclear RoB D3 and D5.<br>Downgraded: Low quality – (4) Inferior animal models were used. Disease-model is useful to assess immunomodulatory mechanisms. However, there are other mechanisms involved in the pathogenesis of the model that may also be related to BoNT activity.	LOW ⊕⊕○○
**Neurons IB4 (+) IB4 (-)**	**TRG sensory neurons**					
	**TN**	**Kitamura 2009 [29]**	KCl-evoked vesicular release FM4-64	1 nRT (n=?)<br>(T1) 11-days (↓) ipl.	Start: Very Low quality (nRCT)	VERY LOW ⊕○○○
**Inflammatory cells** lymphocyte, monocyte, neutrophile, plasma cells	**Cranial dura tissue**					
	**Trigeminal pain - TMDs (inflammatory pain)**	**Lacković, 2016 [20]**	number of Giemsa positive profiles - Scale bars = 100 μm	1 RCT (n=105)<br>(T1) 4-days (↓)	Start: High quality (RCT)<br>Downgraded: Moderate quality - (1) High RoB D8; Unclear RoB D3 and D7.	MODERATE ⊕⊕⊕○
**Fibroblasts**	**Scar tissue (ear)**					
	**HS**	**Wang, 2020 [25]**	(%) apoptosis rate	1 RCT (n=18)<br>(T1) 28 days (↑)	Start: High quality (RCT)<br>Downgraded: Moderate quality - (1) High RoB D10, Unclear RoB D3, D5 and D7.<br>Downgraded: Low quality – (4) The rabbit ear model cannot completely represent human HS.	LOW ⊕⊕○○
	**TG**					
**HIF-1α**	**TN**	**Cho, 2022 [17]**	protein expression (pg/ml tissue)	1 RCT (n=236)<br>(T1) 2 days (↓)	Start: High quality (RCT)<br>Downgraded: Moderate quality - (1) Unclear RoB D3 and D7.<br>Downgraded: Low quality – (4) There are no current animal models of TN that adequately replicate the clinical disorder. Most used IoNC. Compression of TNR has been linked to the main theory for the pathophysiology of TN.	LOW ⊕⊕○○
	**TG (mandibular (V3) division and boundary area)**					

**Nav 1.3**	**TN**	**Yang, 2016 [31]**	expression	1 ? (n=?)<br>(T1) 6-days (NS)	Start: Very Low quality (nRCT)	VERY LOW<br>⊕○○○
**Nav 1.6**				1 ? (n=?)<br>(T1) 6-days (NS)	Start: Very Low quality (nRCT)	VERY LOW<br>⊕○○○
**Nav 1.7**				1 ? (n=?)<br>(T1) 6-days (↓)	Start: Very Low quality (nRCT)	VERY LOW<br>⊕○○○
**Nav 1.8**				1 ? (n=?)<br>(T1) 6-days (NS)	Start: Very Low quality (nRCT)	VERY LOW<br>⊕○○○
**ATF3**			positive cells	1 ? (n=?)<br>(T1) 6-days (NS)	Start: Very Low quality (nRCT)	VERY LOW<br>⊕○○○
**Trigeminal spinal subnucleus caudalis**						
**TRPM3**	**TN**	**Zhang, 2019 [32]**	protein expression, OD	1 RCT (n=236)<br>(T1) 7 days (↓)	Start: High quality (RCT)<br>Downgraded: Moderate quality - (1) Unclear RoB D3 and D7.<br>Downgraded: Low quality – (4) There are no current animal models of TN that adequately replicate the clinical disorder. Most used IoNC. Compression of TNR has been linked to the main theory for the pathophysiology of TN.	LOW<br>⊕⊕○○
**TRPV4**				1 RCT (n=236)<br>(T1) 7 days (↓)	Start: High quality (RCT)<br>Downgraded: Moderate quality - (1) Unclear RoB D3 and D7.<br>Downgraded: Low quality – (4) There are no current animal models of TN that adequately replicate the clinical disorder. Most used IoNC. Compression of TNR has been linked to the main theory for the pathophysiology of TN.	LOW<br>⊕⊕○○
**Brainstem Vc region (caudal subnucleus of the spinal trigeminal nucleus)**						
**TRPV1**	**TN**	**Wu, 2016 [23]**	protein levels, OD	1 ? (n=?)<br>(T1) 7 days – (↓)<br>BoNT (3U, 10U)	Start: Very Low quality (nRCT)	VERY LOW<br>⊕○○○
**TRPV2**				1 ? (n=?)<br>(T1) 7 days – (↓)<br>BoNT (10U)	Start: Very Low quality (nRCT)	VERY LOW<br>⊕○○○
**TRPM8**				1 ? (n=?)<br>(T1) 7 days – (NS)	Start: Very Low quality (nRCT)	VERY LOW<br>⊕○○○
**Hippocampus (brain) tissues – total protein isolates**						
**SOD**	**Anxiety & ageing**	**Yesudhas 2021 [34]**	U/mg protein	1 ? (n=12)<br>(T1) 30-days (↑)	Start: Very Low quality (nRCT)	VERY LOW<br>⊕○○○

**Catalase**				1 ? (n=12)<br>(T1) 30-days (↑)	Start: Very Low quality (nRCT)	VERY LOW<br>⊕○○○
**GSH**				1 ? (n=12)<br>(T1) 30-days (↑)	Start: Very Low quality (nRCT)	VERY LOW<br>⊕○○○
**GPx**				1 ? (n=12)<br>(T1) 30-days (↑)	Start: Very Low quality (nRCT)	VERY LOW<br>⊕○○○

LEGEND: **RCT**, randomised controlled trial; **nRT**, non-randomised trial; **RoB**, risk of bias; **CIS**, chronic inflammatory state; **BoNT,** botulinum toxin; **(↑)**, statistically significantly higher/increased; **(↓),** statistically significantly lower/decreased; **(√)**, increased but not significantly; **(-)**, decrease but not significantly; **(X)**, no remarkable changes; **(B)**, baseline; **SEM,** standard error of the mean; **NS**, no statistically significant difference; **NR**, not reported; **ip.l.**, ipsilateral; **c.l.**, contralateral; **s.c.**, subcutaneously; **i.c.**, intracisternally**; i.a.**, intraarticular; **i.g**. intraganglionic; **POD**, post operative day; **Tx**, treatment; **O.D,** optical density**; (mm)**, millimetres; **SD**, standard deviation; **ANOVA**; analysis of variance; **(r),** decay time constant; **CBCT**, Cone Beam computer Tomography; **ELISA**, enzyme linked immunosorbent assay; **qRT–PCR**; Real-Time Quantitative Reverse Transcription PCR**; RGS**, facial grimacing related to pain; **FST**, forced swimming test; **SEI**, scar elevation index; **TEWL,** transepidermal water loss; **HS**, hypertrophic scar; **PRP**, platelet rich plasma; **TNR**; trigeminal nerve root; **CCI**, chronic constriction injury; **NeuN**, neuronal nuclei; **WIM**, whisker intrinsic musculature; **OFT**, open field test; **MMP-13**, matrix metallopeptidase; **TG**, trigeminal ganglia; **PD**, Parkinson Disease; **TNC**, trigeminal nucleus caudalis; **TMD**, temporomandibular disorder; **TMJ**, temporomandibular joint; **OA**, osteoarthritis; **TN**, trigeminal neuralgia; **PIH**, persistent immunogenic hypersensitivity; **TNF-α**, tumor necrotic factor-α; **IL**, interleukin; **ION-CCI/IoNC**, infraorbital nerve constriction; **SNpc**, substantia nigra pars compacta; **vlPAG**, ventrolateral periaqueductal gray; **mBSA**, methylated bovine serum albumin; **CFA**, Complete Freund's Adjuvant; **PBS**, phosphate-buffered saline; **NTG**, nitroglycerin; **SP**, substance P; **CGRP**, calcitonin gene related peptide; **BDNF**, brain derived neurotrophic factor; **(cl)SNAP-25**, (cleaved) synaptosomal-associated protein-25; **ChAT**, choline acetyltransferase; **PSD95**, postsynaptic density-95; **NMDAR**, N-methyl-D-aspartate receptor; **5-HT**, 5-hydroxytryptamine; **SRS**; spatial restraint stress; **Vc**, caudal subnucleus of the spinal trigeminal nucleus**; p-ERK**, phosphorylated extracellular signal-regulated kinase; **p-CREB**, cAMP response element binding protein; **Iba-1**; ionized calcium-binding adaptor molecule 1; **TLRs** - toll-like receptors; **c-Fos**, neuron activation marker; **GFPA**, glial fibrillary acidic protein; **DNI**, dural neurogenic inflammation; **IgE**, immunoglobulin E; **CX3CR1,** CX3 chemokine receptor 1; **IB4**, isolectin B4-binding; **COL-I**, collagen 1-related proteins; **COL-II**, collagen 2-related proteins; **α-SMA**, α- smooth muscle actin; **HIF-1α**, hypoxia-inducible factor; **TH**, tyrosine hydroxylase, dopaminergic neuronal marker; **VGlut2,** vesicular glutamate transporter 2; **VGAT,** vesicular GABA transporter; **SOD**, superoxide dismutase; **GSH,** glutathione; **GPx,** glutathione peroxidase; **DRG**; dorsal root ganglia; **ATF3**, activating transcription factor 3; **TRPV4**, protein expression of transient receptor potential vanilloid type 4; **TRPM**, transient receptor potential melastatin; **TNCB**, 2-Chloro-1,3,5-trinitrobenzene; **TRPV,** transient receptor potential vanilloid type ; **TRPA1**, transient receptor potential ankyrin 1; **Vc**, caudal subnucleus of the spinal trigeminal nucleus; **AEW**, acetone-diethylether-water; **TGF-β1**, transforming growth factor beta; **vAChT**, acetylcholine vesicular transporter; **SerT**, serotonin transporter; **CTB-488**, monosynaptic retrograde tracer; **PRV-EGFP**, retrograde polytranssynaptic pseudorabies virus (PRV) tracer, EGFP-conjugated; **wFMNs**, whisker-innervating facial motoneurons; **CaMKII**, excitatory Ca^{2+}/calmodulin-dependent protein kinase type II; **GAD67**, inhibitory glutamate decarboxylase 1; **vlPAG,** ventrolateral periaqueductal grey; **TpH2**, tryptophan hydroxylase 2, serotoninergic neuronal marker; **GAPDH,** Glyceraldehyde 3-phosphate dehydrogenase.

KEY:

(*) based on Wei, D., Tang, K., Wang, Q., Estill, J., Yao, L., Wang, X., Chen, Y., & Yang, K. (2016). The use of GRADE approach in systematic reviews of animal studies. *Journal of evidence-based medicine*, *9*(2), 98–104. https://doi.org/10.1111/jebm.12198

(**) for the GRADE domain 1 (risk of bias), we decreased the level of certainty one level for studies rated high risk of bias at least in one domain, or unclear if lack of concealment of randomization (D3) and/or lack of blinding (D5, D7). We downgraded two levels the studies rated high risk of bias due to lack of concealment of randomization or lack of blinding.

GRADE certainty ratings

Very low The true effect is probably markedly different from the estimated effect
Low The true effect might be markedly different from the estimated effect
Moderate The authors believe that the true effect is probably close to the estimated effect
High The authors have a lot of confidence that the true effect is similar to the estimated effect
